# Molecular Mimicry between Respiratory Syncytial Virus F Antigen and the Human Proteome

**DOI:** 10.1055/s-0043-1761489

**Published:** 2023-01-30

**Authors:** Darja Kanduc

**Affiliations:** 1 Department of Biosciences, Biotechnologies, and Biopharmaceutics, University of Bari, Bari, Italy

**Keywords:** molecular mimicry, RSV F, anti-RSV vaccines

## Abstract

This study examined respiratory syncytial virus (RSV) F glycoprotein (gp) antigen for molecular mimicry with the human proteome. It was found that the viral antigen presents an impressive number of pentapeptides (namely, 525 out of 570) in common with the human proteome, with viral sequences widely and repeatedly distributed among 3,762 human proteins implicated in crucial fundamental cellular functions. The data can have implications for anti-RSV vaccines. Indeed, the high level of molecular mimicry can lead to cross-reactivity and autoimmunity, and invites to follow safer vaccinal protocols based on pentapeptide sequences uniquely present in the viral antigen.

## Introduction

Molecular mimicry, that is the sharing of sequences between pathogen and human proteins, is recently under intense investigation 1 2 3 to explain the thousands of adverse events associated with the anti-severe acute respiratory syndrome coronavirus 2 (SARS-CoV-2) vaccination campaign. 4 Currently, as a few examples among the many, it has been documented that

- Antineuronal antibodies against brainstem antigens are associated with coronavirus disease 2019 (COVID-19) 5
- COVID-19 induces autoantibodies against angiotensin II that correlate with blood pressure dysregulation and disease severity 6
- Molecular mimicry is a prerequisite of autoimmunity following COVID-19. 7

In general, the SARS-COV-2 data support and validate previous studies 8 9 10 11 12 according to which molecular mimicry and the consequent cross-reactivity represent the most likely pathogenic mechanism that leads to autoimmunity following vaccinations against infectious agents. Here, as a continuation of such studies, matching analyses have been extended to respiratory syncytial virus (RSV) F antigen and findings are described in Table 1 .

**Table 1:** Numerical description of the pentapeptide sharing between RSV F gp and the human proteome a b

Number of human proteins involved in the sharing	3,762
Viral pentapeptides occurring in the human proteome (including multiple occurrences)	4,996
Number of pentapeptides composing the viral antigen	5,70
Number of pentapeptides shared with the human proteome	5,25

Abbreviation: RSV, respiratory syncytial virus.

a Pentapeptides were used as probes since a pentapeptide acts as a minimal determinant in humoral and cellular immune recognition. 13

b The methodology for defining the pentapeptide sharing has been described in 11 12 and utilized UniProt Peptide Search programs (www.uniprot.org). ^14–16^ Analyses were performed on the primary sequence of RSV F gp, 574 amino acid long, Uniprot accession (AC) entry P03420.

Analysis of Table 1 highlights three main points:

1. First, the number of human proteins involved in the peptide sharing with the viral antigen is highest, that is 3,762. Moreover, many viral pentapeptides recur repeatedly among the 3,762 human proteins so that, including multiple occurrences, the total number of viral pentapeptides occurring in the human proteome is actually 4,996.
2. Second, mathematically the massive dimensions of such viral versus human peptide sharing are unexpected. In fact, assuming that all amino acids occur with the same frequency, the probability of 1 pentapeptide occurring in two proteins is 1 out of 20 5 (or 1 in 3,200,000 or 0.0000003125). That is, it is close to zero.
3. Third, only 45 out of the 570 viral pentapeptides are not shared with the human proteome, thus being unique to RSV F. They are in the order: VTFCF; TFCFA; YQSTC; RTGWY; TGWYT; GWYTS; NKCNG; KCNGT; YKNAV; MNYTL; KNYID; NKQSC; KQSCS; QQKNN; QKNNR; YMLTN; MPITN; GVIDT; DTPCW; TPCWK; CWKLH; WKLHT; PLCTT; LCTTN; GSNIC; TDRGW; DRGWY; RGWYC; GWYCD; YCDNA; QAETC; AETCK; VFCDT; EINLC; PKYDC; YDCKI; DCKIM; IVSCY; CYGKT; GCDYV; YVNKQ; HNVNA; TNIMI; YCKAR; NNIAF. Of note, a few unique viral pentapeptides overlap each other by four residues thus forming longer peptide stretches, that is, TDRGWYCD. Intriguingly, analysis of the Immune Epitope DataBase (IEDB, www.iedb.org ) reveals that the octamer TDRGWYCD is present in the immunoreactive RSV F-derived epitope RTDRGWYCDNAGSVS (IEDB ID: 956694, with the octamer given underlined). 17

Obvious space reasons prevent listing/discussing the 3,762 human proteins involved in the sharing and, as well, the multiple occurrences of the viral pentapeptides. Hence, data are given in Supplemental Table S1 , which is an essential part of this report to illustrate and infer the pathological potential of the peptide sharing. Indeed, for example, inspection of Supplemental Table S1 (online only) shows that 29 coiled-coil domain-containing (CCDC) proteins are involved in the peptide sharing. CCDC proteins are implicated in numerous physiological and pathological processes like gametogenesis, embryonic development, hematopoiesis, angiogenesis, and ciliary development. 18 Then, it is evident that cross-reactivity with CCDC proteins alone would lead in itself to unrepairable damages to humans.

## Conclusions

The massive peptide commonality between RSV F antigen gp and the human proteome indicates and confirms a strict phenetic relationship between viruses and the origin of eukaryotic cells according to the endosymbiotic theory. 19 Such common evolutionary link can have a heavy immunological impact on and puts warns against using RSV F antigen or its oligopeptides in vaccinal compositions. Indeed, the highest extent of the peptide sharing between the viral antigen and human proteins and the consequent cross-reactive potential could cause collateral effects of unimaginable proportions both in number and in severity. In this regard, this report describes the 45 pentapeptide sequences unique to the RSV F antigen and absent in the human proteome, thus providing a molecular platform that can be used to formulate vaccines exempt of the cross-reactivity burden. De facto, using as an antigen the octamer TDRGWYCD described above, offers the possibility of formulating effective and safe immunotherapies for specifically hitting RSV.
