# Supplementary material for: Molecular Mimicry between Respiratory Syncytial Virus F Antigen and the Human Proteome
Source: Glob Med Genet. 2023 Jan 30;10(1):19–21. doi: 10.1055/s-0043-1761489 (PMC9886499; doi:10.1055/s-0043-1761489)
Supplement: Supplementary file 1 — Supplementary Material [file 10-1055-s-0043-1761489-s2200075.pdf]

Supplemental Table S1 Pentapeptide matches between RSV F gp antigen and human proteins

Viral Peptide	Protein AC	Protein ID	Protein Name
ILKAN	Q9P0J1	PDP1	[Pyruvate dehydrogenase [acetyl-transferring]]-phosphatase 1, mitochondrial precursor
AVTEL	Q07973	CP24A	1,25-dihydroxyvitamin D(3) 24-hydroxylase, mitochondrial precursor
VTELQ	Q07973	CP24A	1,25-dihydroxyvitamin D(3) 24-hydroxylase, mitochondrial precursor
TELQL	Q07973	CP24A	1,25-dihydroxyvitamin D(3) 24-hydroxylase, mitochondrial precursor
QKKLM	Q07973	CP24A	1,25-dihydroxyvitamin D(3) 24-hydroxylase, mitochondrial precursor
RLLEI	Q04446	GLGB	1,4-alpha-glucan-branching enzyme
TLPSE	Q15029	U5S1	116 kDa U5 small nuclear ribonucleoprotein component
STPVT	Q15029	U5S1	116 kDa U5 small nuclear ribonucleoprotein component
AVTEL	Q04917	1433F	14-3-3 protein eta
RSTPV	Q9BPX1	DHB14	17-beta-hydroxysteroid dehydrogenase 14
SGVAV	Q9UJK0	TSR3	18S rRNA aminocarboxypropyltransferase
LPLYG	Q9NUQ2	PLCE	1-acyl-sn-glycerol-3-phosphate acyltransferase epsilon
TKEGS	Q9BRC7	PLCD4	1-phosphatidylinositol 4,5-bisphosphate phosphodiesterase delta-4
DIFNP	Q9BRC7	PLCD4	1-phosphatidylinositol 4,5-bisphosphate phosphodiesterase delta-4

TSLGA	Q9P212	PLCE1	1-phosphatidylinositol 4,5-bisphosphate phosphodiesterase epsilon-1
DIFNP	Q4KWH8	PLCH1	1-phosphatidylinositol 4,5-bisphosphate phosphodiesterase eta-1
DIFNP	O75038	PLCH2	1-phosphatidylinositol 4,5-bisphosphate phosphodiesterase eta-2
KNNRL	P19174	PLCG1	1-phosphatidylinositol 4,5-bisphosphate phosphodiesterase gamma-1
EFSVN	P16885	PLCG2	1-phosphatidylinositol 4,5-bisphosphate phosphodiesterase gamma-2
PSEIN	P16885	PLCG2	1-phosphatidylinositol 4,5-bisphosphate phosphodiesterase gamma-2
SNIKE	Q86YW0	PLCZ1	1-phosphatidylinositol 4,5-bisphosphate phosphodiesterase zeta-1
TELQL	Q6L8Q7	PDE12	2',5'-phosphodiesterase 12 precursor
KEEVL	Q15646	OASL	2'-5'-oligoadenylate synthase-like protein
ELQLL	P62195	PRS8	26S proteasome regulatory subunit 8
LLSTN	P82664	RT10	28S ribosomal protein S10, mitochondrial
QLSGI	O60783	RT14	28S ribosomal protein S14, mitochondrial
LHTSP	Q96EL2	RT24	28S ribosomal protein S24, mitochondrial precursor
GAIVS	P51398	RT29	28S ribosomal protein S29, mitochondrial precursor
SELLS	Q92665	RT31	28S ribosomal protein S31, mitochondrial precursor
LILKA	P82932	RT06	28S ribosomal protein S6, mitochondrial
LIKQE	Q9ULD0	OGDHL	2-oxoglutarate dehydrogenase-like, mitochondrial precursor
IKENK	Q9H6S0	YTDC2	3'-5' RNA helicase YTHDC2
KVKLI	P52815	RM12	39S ribosomal protein L12, mitochondrial precursor

VKLIK	P52815	RM12	39S ribosomal protein L12, mitochondrial precursor
VRQQS	P49406	RM19	39S ribosomal protein L19, mitochondrial precursor
GEVNK	Q9NYK5	RM39	39S ribosomal protein L39, mitochondrial
QELDK	P21217	FUT3	3-galactosyl-N-acetylglucosaminide 4-alpha-L-fucosyltransferase FUT3
KLHTS	P04035	HMDH	3-hydroxy-3-methylglutaryl-coenzyme A reductase
ASGVA	P42765	THIM	3-ketoacyl-CoA thiolase, mitochondrial
IKENK	O15530	PDPK1	3-phosphoinositide-dependent protein kinase 1
SKKRK	P23396	RS3	40S ribosomal protein S3
LVFPS	P62081	RS7	40S ribosomal protein S7
QELDK	Q11128	FUT5	4-galactosyl-N-acetylglucosaminide 3-alpha-L-fucosyltransferase FUT5
QELDK	P51993	FUT6	4-galactosyl-N-acetylglucosaminide 3-alpha-L-fucosyltransferase FUT6
DELLH	Q9NVE7	PANK4	4'-phosphopantetheine phosphatase
REFSV	P17643	TYRP1	5,6-dihydroxyindole-2-carboxylic acid oxidase precursor
CSAVS	O43422	P52K	52 kDa repressor of the inhibitor of the protein kinase
ISQVN	Q8IZH2	XRN1	5'-3' exoribonuclease 1
STPVT	Q8IZH2	XRN1	5'-3' exoribonuclease 1
KLMSN	Q9H0D6	XRN2	5'-3' exoribonuclease 2
GNTLY	P54619	AAKG1	5'-AMP-activated protein kinase subunit gamma-1
VVSLS	Q9UGI9	AAKG3	5'-AMP-activated protein kinase subunit gamma-3
VITSL	P08908	5HT1A	5-hydroxytryptamine receptor 1A

LGFLL	A5X5Y0	5HT3E	5-hydroxytryptamine receptor 3E precursor
GMDTV	O14841	OPLA	5-oxoprolinase
DKYKN	P10809	CH60	60 kDa heat shock protein, mitochondrial precursor
NDQKK	P46778	RL21	60S ribosomal protein L21
TASNK	P49207	RL34	60S ribosomal protein L34
KRKRR	P62945	RL41	60S ribosomal protein L41
ASGVA	O60733	PLPL9	85/88 kDa calcium-independent phospholipase A2
GFLLG	Q76LX8	ATS13	A disintegrin and metalloproteinase with thrombospondin motifs 13 precursor
LYCKA	Q8TE60	ATS18	A disintegrin and metalloproteinase with thrombospondin motifs 18 precursor
NIETV	Q8TE59	ATS19	A disintegrin and metalloproteinase with thrombospondin motifs 19 precursor
NEKIN	Q8TE59	ATS19	A disintegrin and metalloproteinase with thrombospondin motifs 19 precursor
KAVVS	P59510	ATS20	A disintegrin and metalloproteinase with thrombospondin motifs 20 precursor
AVVSL	P59510	ATS20	A disintegrin and metalloproteinase with thrombospondin motifs 20 precursor
TPVST	O15072	ATS3	A disintegrin and metalloproteinase with thrombospondin motifs 3 precursor
LLPIV	O75173	ATS4	A disintegrin and metalloproteinase with thrombospondin motifs 4 precursor

SCSIS	Q9P2N4	ATS9	A disintegrin and metalloproteinase with thrombospondin motifs 9 precursor
LLILK	Q86TW2	ADCK1	AarF domain-containing protein kinase 1 precursor
AGKST	O94911	ABCA8	ABC-type organic anion transporter ABCA8
STPPT	Q9NYB9	ABI2	Abl interactor 2
VGLLL	Q04844	ACHE	Acetylcholine receptor subunit epsilon precursor
LKANA	Q13085	ACACA	Acetyl-CoA carboxylase 1
LKANA	O00763	ACACB	Acetyl-CoA carboxylase 2 precursor
LSLIN	Q92688	AN32B	Acidic leucine-rich nuclear phosphoprotein 32 family member B
AVTFC	Q9NY37	ASIC5	Acid-sensing ion channel 5
VSKVL	Q9NY37	ASIC5	Acid-sensing ion channel 5
SKVLH	Q9NY37	ASIC5	Acid-sensing ion channel 5
KVLHL	Q9NY37	ASIC5	Acid-sensing ion channel 5
EEFYQ	O94929	ABLM3	Actin-binding LIM protein 3
TLPSE	Q86TU7	SETD3	Actin-histidine N-methyltransferase
GNTLY	Q9H568	ACTL8	Actin-like protein 8
NKAVV	Q8TDG2	ACTT1	Actin-related protein T1
KKLMS	Q15650	TRIP4	Activating signal cointegrator 1
EEVLA	Q8N9N2	ASCC1	Activating signal cointegrator 1 complex subunit 1
TPVST	Q6VMQ6	MCAF1	Activating transcription factor 7-interacting protein 1
LVFPS	Q12979	ABR	Active breakpoint cluster region-related protein
VNAGK	Q8NER5	ACV1C	Activin receptor type-1C precursor
KQLLP	Q9H2P0	ADNP	Activity-dependent neuroprotector homeobox protein

NSELL	Q6IQ32	ADNP2	Activity-dependent neuroprotector homeobox protein 2
NSLTL	Q6IQ32	ADNP2	Activity-dependent neuroprotector homeobox protein 2
NTLYY	Q6IQ32	ADNP2	Activity-dependent neuroprotector homeobox protein 2
TVSVG	Q7LC44	ARC	Activity-regulated cytoskeleton-associated protein
TNVTL	Q92604	LGAT1	Acyl-CoA:lysophosphatidylglycerol acyltransferase 1
VIDTP	Q9NUZ1	ACOXL	Acyl-coenzyme A oxidase-like protein
IDTPC	Q9NUZ1	ACOXL	Acyl-coenzyme A oxidase-like protein
GLLLY	Q08AH1	ACSM1	Acyl-coenzyme A synthetase ACSM1, mitochondrial precursor
PLVFP	Q9Y305	ACOT9	Acyl-coenzyme A thioesterase 9, mitochondrial precursor
TIELS	Q53H12	AGK	Acylglycerol kinase, mitochondrial
SLSNG	P28039	AOAH	Acyloxyacyl hydrolase precursor
TNKAV	Q8N6G6	ATL1	ADAMTS-like protein 1 precursor
SVLTS	Q8N6G6	ATL1	ADAMTS-like protein 1 precursor
EEVLA	Q8N6G6	ATL1	ADAMTS-like protein 1 precursor
TLPSE	Q8N6G6	ATL1	ADAMTS-like protein 1 precursor
PVTLS	Q8N6G6	ATL1	ADAMTS-like protein 1 precursor
AVTFC	Q9H1N7	S35B3	Adenosine 3'-phospho 5'-phosphosulfate transporter 2
LLILK	P0DMS8	AA3R	Adenosine receptor A3
LILKA	P0DMS8	AA3R	Adenosine receptor A3
SVLTS	Q08462	ADCY2	Adenylate cyclase type 2
LPRFM	O60266	ADCY3	Adenylate cyclase type 3
LSLIA	O60266	ADCY3	Adenylate cyclase type 3
GVSVL	Q8NFM4	ADCY4	Adenylate cyclase type 4

SLYVK	Q8NFM4	ADCY4	Adenylate cyclase type 4
VGLLL	O95622	ADCY5	Adenylate cyclase type 5
SLYVK	P51828	ADCY7	Adenylate cyclase type 7
SVLTS	O60503	ADCY9	Adenylate cyclase type 9
EEVLA	O95396	MOCS3	Adenylyltransferase and sulfurtransferase MOCS3
SLGAI	Q8IZF2	AGRF5	Adhesion G protein-coupled receptor F5 precursor
KQEGK	Q8IZF2	AGRF5	Adhesion G protein-coupled receptor F5 precursor
DVSSS	O94910	AGRL1	Adhesion G protein-coupled receptor L1 precursor
VSSSV	O94910	AGRL1	Adhesion G protein-coupled receptor L1 precursor
DVSSS	O95490	AGRL2	Adhesion G protein-coupled receptor L2 precursor
VSSSV	O95490	AGRL2	Adhesion G protein-coupled receptor L2 precursor
ILLSL	Q9HAR2	AGRL3	Adhesion G protein-coupled receptor L3 precursor
SNRVF	Q6QNK2	AGRD1	Adhesion G-protein coupled receptor D1 precursor
VGSAI	Q7Z7M1	AGRD2	Adhesion G-protein coupled receptor D2
SVITI	Q5T601	AGRF1	Adhesion G-protein coupled receptor F1 precursor
SELLS	Q5T601	AGRF1	Adhesion G-protein coupled receptor F1 precursor
TTILT	Q8IZF6	AGRG4	Adhesion G-protein coupled receptor G4 precursor
SLTLP	Q8IZF6	AGRG4	Adhesion G-protein coupled receptor G4 precursor
STPVT	Q8IZF6	AGRG4	Adhesion G-protein coupled receptor G4 precursor
QSLAF	Q8IZF4	AGRG5	Adhesion G-protein coupled receptor G5 precursor
TLSKK	Q8WXG9	AGRV1	Adhesion G-protein coupled receptor V1 precursor
SLGAI	Q8WXG9	AGRV1	Adhesion G-protein coupled receptor V1 precursor
AGKST	Q8WXG9	AGRV1	Adhesion G-protein coupled receptor V1 precursor

VGLLL	Q0VDE8	ADIG	Adipogenin
NITEE	P28907	CD38	ADP-ribosyl cyclase/cyclic ADP-ribose hydrolase 1
AGSVS	Q8N6H7	ARFG2	ADP-ribosylation factor GTPase-activating protein 2
AGKST	Q8N8L6	ARL10	ADP-ribosylation factor-like protein 10
KENKC	Q5H913	AR13A	ADP-ribosylation factor-like protein 13A
AGKST	Q8N4G2	ARL14	ADP-ribosylation factor-like protein 14
STPVT	P51825	AFF1	AF4/FMR2 family member 1
LLSLI	P51816	AFF2	AF4/FMR2 family member 2
VSNKG	Q9UHB7	AFF4	AF4/FMR2 family member 4
TLSKK	P55196	AFAD	Afadin
SLTLP	P43652	AFAM	Afamin precursor
LEGEV	P16112	PGCA	Aggrecan core protein precursor
NAVTE	O00468	AGRIN	Agrin precursor
GLLLY	O00468	AGRIN	Agrin precursor
IELSN	Q9Y2D5	AKAP2	A-kinase anchor protein 2
SISNI	Q13023	AKAP6	A-kinase anchor protein 6
NNRLL	Q99996	AKAP9	A-kinase anchor protein 9
AVGLL	Q99996	AKAP9	A-kinase anchor protein 9
ITEEF	Q2M3C7	SPKAP	A-kinase anchor protein SPHKAP
TEEFY	Q2M3C7	SPKAP	A-kinase anchor protein SPHKAP
TVSVG	Q9BYV1	AGT2	Alanine--glyoxylate aminotransferase 2, mitochondrial precursor
VNEKI	Q9BTE6	AASD1	Alanyl-tRNA editing protein Aarsd1
TPVTL	P51648	AL3A2	Aldehyde dehydrogenase family 3 member A2

LPIVN	P43353	AL3B1	Aldehyde dehydrogenase family 3 member B1 precursor
AFIRK	P43353	AL3B1	Aldehyde dehydrogenase family 3 member B1 precursor
TPVTL	P43353	AL3B1	Aldehyde dehydrogenase family 3 member B1 precursor
LPIVN	P48448	AL3B2	Aldehyde dehydrogenase family 3 member B2 precursor
TPVTL	P48448	AL3B2	Aldehyde dehydrogenase family 3 member B2 precursor
TPVTL	P30838	AL3A1	Aldehyde dehydrogenase, dimeric NADP-preferring
GVSVL	Q9UM73	ALK	ALK tyrosine kinase receptor precursor
TSKTD	Q9UM73	ALK	ALK tyrosine kinase receptor precursor
AVGLL	Q9UM73	ALK	ALK tyrosine kinase receptor precursor
ELSNI	Q86YH6	DLP1	All trans-polyprenyl-diphosphate synthase PDSS2
ALRTG	Q13424	SNTA1	Alpha-1-syntrophin
SLGAI	P18089	ADA2B	Alpha-2B adrenergic receptor
NNRLL	P01023	A2MG	Alpha-2-macroglobulin precursor
RRELP	P12814	ACTN1	Alpha-actinin-1
RRELP	P35609	ACTN2	Alpha-actinin-2
RRELP	Q08043	ACTN3	Alpha-actinin-3
RRELP	O43707	ACTN4	Alpha-actinin-4
SKDQL	P61163	ACTZ	Alpha-centractin
TTILT	Q12800	TFCP2	Alpha-globin transcription factor CP2
LLILK	Q16706	MA2A1	Alpha-mannosidase 2
SEINL	Q16706	MA2A1	Alpha-mannosidase 2
VLDLK	Q9UHK6	AMACR	Alpha-methylacyl-CoA racemase
KSDEL	Q9UHK6	AMACR	Alpha-methylacyl-CoA racemase

LTAVT	Q86TB3	ALPK2	Alpha-protein kinase 2
LTNSE	Q86TB3	ALPK2	Alpha-protein kinase 2
ELQLL	Q16586	SGCA	Alpha-sarcoglycan precursor
KQELD	Q60I27	AL2CL	ALS2 C-terminal-like protein
WYCDN	P51168	SCNNB	Amiloride-sensitive sodium channel subunit beta
ELLHN	P51172	SCNND	Amiloride-sensitive sodium channel subunit delta
GVSVL	P21397	AOFA	Amine oxidase [flavin-containing] A
KRKRR	Q8N6M6	AMPO	Aminopeptidase O
ILTAV	P15514	AREG	Amphiregulin precursor
NRLLE	Q86SJ2	AMGO2	Amphoterin-induced protein 2 precursor
KEEVL	Q06481	APLP2	Amyloid beta precursor like protein 2 precursor
FCDTM	Q86XD8	ZFAN4	AN1-type zinc finger protein 4
SDELL	Q6FI81	CPIN1	Anamorsin
ILKAN	Q9UJX5	APC4	Anaphase-promoting complex subunit 4
SVLTS	Q9UJX5	APC4	Anaphase-promoting complex subunit 4
IKEEV	Q9UJX5	APC4	Anaphase-promoting complex subunit 4
KEEVL	Q9UJX5	APC4	Anaphase-promoting complex subunit 4
VQLPL	Q9UJX5	APC4	Anaphase-promoting complex subunit 4
VNAGV	Q9UJX4	APC5	Anaphase-promoting complex subunit 5
LPSEI	Q9UJX3	APC7	Anaphase-promoting complex subunit 7
AGKST	P10275	ANDR	Androgen receptor
LSALR	Q9Y2J4	AMOL2	Angiomotin-like protein 2
ELQLL	O15123	ANGP2	Angiopoietin-2 precursor

LSTNK	O15123	ANGP2	Angiopoietin-2 precursor
LSTNK	Q9Y264	ANGP4	Angiopoietin-4 precursor
NITEE	Q9BYF1	ACE2	Angiotensin-converting enzyme 2 precursor
RARRE	Q9NQW6	ANLN	Anillin
SVGNT	Q9NQW6	ANLN	Anillin
ARSTP	Q9NQW6	ANLN	Anillin
LLSLI	P48751	B3A3	Anion exchange protein 3
SLSNG	Q8TE54	S26A7	Anion exchange transporter
GKSTT	Q9P0K7	RAI14	Ankycorbin
TRTDR	Q8N957	ANKF1	Ankyrin repeat and fibronectin type-III domain-containing protein 1
SLGAI	Q9P2G1	AKIB1	Ankyrin repeat and IBR domain-containing protein 1
LEGEV	Q8IWZ3	ANKH1	Ankyrin repeat and KH domain-containing protein 1
ALRTG	Q8NAG6	ANKL1	Ankyrin repeat and LEM domain-containing protein 1
ILLSL	Q8NFD2	ANKK1	Ankyrin repeat and protein kinase domain-containing protein 1
AVTEL	Q92625	ANS1A	Ankyrin repeat and SAM domain-containing protein 1A
ASNKN	Q92625	ANS1A	Ankyrin repeat and SAM domain-containing protein 1A
RSTPV	Q9Y576	ASB1	Ankyrin repeat and SOCS box protein 1
VVQLP	Q8WXI3	ASB10	Ankyrin repeat and SOCS box protein 10
LSALR	Q9H672	ASB7	Ankyrin repeat and SOCS box protein 7
ELLSL	Q9NXR5	ANR10	Ankyrin repeat domain-containing protein 10
RKRRF	Q6UB99	ANR11	Ankyrin repeat domain-containing protein 11
ASISQ	O75179	ANR17	Ankyrin repeat domain-containing protein 17
ETVIE	Q8N7Z5	ANR31	Ankyrin repeat domain-containing protein 31

VNEKI	Q8N7Z5	ANR31	Ankyrin repeat domain-containing protein 31
EEVLA	Q8N283	ANR35	Ankyrin repeat domain-containing protein 35
SISNI	A6QL64	AN36A	Ankyrin repeat domain-containing protein 36A
IVILL	A6QL64	AN36A	Ankyrin repeat domain-containing protein 36A
IVILL	Q8N2N9	AN36B	Ankyrin repeat domain-containing protein 36B
SISNI	Q5JPF3	AN36C	Ankyrin repeat domain-containing protein 36C
IVILL	Q5JPF3	AN36C	Ankyrin repeat domain-containing protein 36C
VGLLL	E5RJM6	ANR65	Ankyrin repeat domain-containing protein 65
NSELL	Q2M3V2	SWAHA	Ankyrin repeat domain-containing protein SOWAHA precursor
SELLS	Q2M3V2	SWAHA	Ankyrin repeat domain-containing protein SOWAHA precursor
IKQEL	Q01484	ANK2	Ankyrin-2
KVLDL	P04083	ANXA1	Annexin A1
GKSLY	P50995	ANX11	Annexin A11
GKSLY	P07355	ANXA2	Annexin A2
GKSLY	P09525	ANXA4	Annexin A4
GKSLY	O76027	ANXA9	Annexin A9
LILKA	Q9NQ90	ANO2	Anoctamin-2
SSSVI	Q9NQ90	ANO2	Anoctamin-2
SKKRK	Q6IWH7	ANO7	Anoctamin-7
SSSVI	Q9H6X2	ANTR1	Anthrax toxin receptor 1 precursor
LGAIV	Q9H6X2	ANTR1	Anthrax toxin receptor 1 precursor
IIVIL	P58335	ANTR2	Anthrax toxin receptor 2 precursor
LTSKV	A6NF34	ANTRL	Anthrax toxin receptor-like precursor

LHLEG	Q03518	TAP1	Antigen peptide transporter 1
LGAIV	Q96A70	AZIN2	Antizyme inhibitor 2
ILTAV	O75843	AP1G2	AP-1 complex subunit gamma-like 2
EEVLA	O75843	AP1G2	AP-1 complex subunit gamma-like 2
VLDLK	Q9BXS5	AP1M1	AP-1 complex subunit mu-1
AVSKG	O95782	AP2A1	AP-2 complex subunit alpha-1
AVSKG	O94973	AP2A2	AP-2 complex subunit alpha-2
IETVI	O94973	AP2A2	AP-2 complex subunit alpha-2
LSTNK	Q9UPP5	K1107	AP2-interacting clathrin-endocytosis protein
SSSVI	O00203	AP3B1	AP-3 complex subunit beta-1
IKQEL	O14617	AP3D1	AP-3 complex subunit delta-1
LLILK	Q96N21	AP4AT	AP-4 complex accessory subunit Tepsin
EFSVN	Q9UPM8	AP4E1	AP-4 complex subunit epsilon-1
VVSLS	O00189	AP4M1	AP-4 complex subunit mu-1
VSKVL	Q8N7J2	AMER2	APC membrane recruitment protein 2
EGKSL	Q8N944	AMER3	APC membrane recruitment protein 3
VSKVL	P04114	APOB	Apolipoprotein B-100 precursor
LSNGV	P04114	APOB	Apolipoprotein B-100 precursor
SSSVI	P04114	APOB	Apolipoprotein B-100 precursor
SSVIT	P04114	APOB	Apolipoprotein B-100 precursor
RRFLG	Q96NN9	AIFM3	Apoptosis-inducing factor 3
AVSKV	Q13625	ASPP2	Apoptosis-stimulating of p53 protein 2
RSTPV	Q9UKV3	ACINU	Apoptotic chromatin condensation inducer in the nucleus

SIIKE	O14727	APAF	Apoptotic protease-activating factor 1
ELQLL	Q8NBQ7	AQP11	Aquaporin-11
SALRT	Q8IXF9	AQ12A	Aquaporin-12A
SALRT	A6NM10	AQ12B	Aquaporin-12B
LILKA	O94778	AQP8	Aquaporin-8
NTKEG	P53367	ARFP1	Arfaptin-1
SAVSK	P52594	AGFG1	Arf-GAP domain and FG repeat-containing protein 1
AVSKV	P52594	AGFG1	Arf-GAP domain and FG repeat-containing protein 1
STNKA	P52594	AGFG1	Arf-GAP domain and FG repeat-containing protein 1
STPVT	Q96P48	ARAP1	Arf-GAP with Rho-GAP domain, ANK repeat and PH domain-containing protein 1
IELSN	Q8WZ64	ARAP2	Arf-GAP with Rho-GAP domain, ANK repeat and PH domain-containing protein 2
KVKLI	Q8WZ64	ARAP2	Arf-GAP with Rho-GAP domain, ANK repeat and PH domain-containing protein 2
TLSKK	Q9ULH1	ASAP1	Arf-GAP with SH3 domain, ANK repeat and PH domain-containing protein 1
LSKKR	Q9ULH1	ASAP1	Arf-GAP with SH3 domain, ANK repeat and PH domain-containing protein 1
SAIAS	Q9NVT9	ARMC1	Armadillo repeat-containing protein 1
DLKNY	Q8NEN0	ARMC2	Armadillo repeat-containing protein 2
NIETV	Q8NEN0	ARMC2	Armadillo repeat-containing protein 2
VGLLL	Q8NEN0	ARMC2	Armadillo repeat-containing protein 2

DAKVK	Q5H9R4	ARMX4	Armadillo repeat-containing X-linked protein 4
VTTPV	Q5T2E6	ARMD3	Armadillo-like helical domain-containing protein 3
TTPVS	Q5T2E6	ARMD3	Armadillo-like helical domain-containing protein 3
AIASG	P27540	ARNT	Aryl hydrocarbon receptor nuclear translocator
TLSKD	P35869	AHR	Aryl hydrocarbon receptor precursor
DAKVK	A9YTQ3	AHRR	Aryl hydrocarbon receptor repressor
ELLHN	P15848	ARSB	Arylsulfatase B precursor
HTSPL	P54793	ARSF	Arylsulfatase F precursor
STYML	Q6UWY0	ARSK	Arylsulfatase K precursor
ITSLG	P07307	ASGR2	Asialoglycoprotein receptor 2
TSLGA	P07307	ASGR2	Asialoglycoprotein receptor 2
VLDLK	P08243	ASNS	Asparagine synthetase [glutamine-hydrolyzing]
KRKRR	O75129	ASTN2	Astrotactin-2 precursor
TIELS	Q9H3M9	ATX3L	Ataxin-3-like protein
KRKRR	Q5TGY3	AHDC1	AT-hook DNA-binding motif-containing protein 1
VGLLL	Q6DD88	ATLA3	Atlastin-3
SKKRK	O43313	ATMIN	ATM interactor
PIINF	O43313	ATMIN	ATM interactor
NAKKT	Q5VTU8	AT5EL	ATP synthase subunit epsilon-like protein, mitochondrial
LDKYK	Q99766	ATP5S	ATP synthase subunit s, mitochondrial precursor
IIKEE	Q6PL18	ATAD2	ATPase family AAA domain-containing protein 2
KKRKR	Q96QE3	ATAD5	ATPase family AAA domain-containing protein 5
KRRFL	Q96QE3	ATAD5	ATPase family AAA domain-containing protein 5

GYLSA	Q9Y6X9	MORC2	ATPase MORC2
RGIIK	Q9Y6X9	MORC2	ATPase MORC2
NSELL	Q9H8K7	PAAT	ATPase PAAT
AGKST	Q8WWZ4	ABCAA	ATP-binding cassette sub-family A member 10
LSLIA	Q8WWZ4	ABCAA	ATP-binding cassette sub-family A member 10
AGKST	Q86UQ4	ABCAD	ATP-binding cassette sub-family A member 13
GKSTT	Q86UQ4	ABCAD	ATP-binding cassette sub-family A member 13
ILKAN	Q9BZC7	ABCA2	ATP-binding cassette sub-family A member 2
NYTLN	Q9BZC7	ABCA2	ATP-binding cassette sub-family A member 2
AGKST	Q8IUA7	ABCA9	ATP-binding cassette sub-family A member 9
GKSTT	Q8IUA7	ABCA9	ATP-binding cassette sub-family A member 9
TLYYV	Q2M3G0	ABCB5	ATP-binding cassette sub-family B member 5
AGKST	Q9NP58	ABCB6	ATP-binding cassette sub-family B member 6
LYCKA	Q5T3U5	MRP7	ATP-binding cassette sub-family C member 10
QLLPI	Q96J66	MRP8	ATP-binding cassette sub-family C member 11
ELQLL	O15438	MRP3	ATP-binding cassette sub-family C member 3
PVTLS	O15439	MRP4	ATP-binding cassette sub-family C member 4
LILKA	O15440	MRP5	ATP-binding cassette sub-family C member 5
ASGVA	P33897	ABCD1	ATP-binding cassette sub-family D member 1
AGKST	Q9UG63	ABCF2	ATP-binding cassette sub-family F member 2
TREFS	Q9NUQ8	ABCF3	ATP-binding cassette sub-family F member 3
AGKST	Q9NUQ8	ABCF3	ATP-binding cassette sub-family F member 3
AGKST	P45844	ABCG1	ATP-binding cassette sub-family G member 1

AGKST	Q9H172	ABCG4	ATP-binding cassette sub-family G member 4
GKSTT	P53396	ACLY	ATP-citrate synthase
VVSLS	P08237	PFKAM	ATP-dependent 6-phosphofructokinase, muscle type
SIIKE	O76031	CLPX	ATP-dependent Clp protease ATP-binding subunit clpX-like, mitochondrial precursor
FASGQ	Q9H611	PIF1	ATP-dependent DNA helicase PIF1
GIIKT	Q9NUU7	DD19A	ATP-dependent RNA helicase DDX19A
KQELD	Q9GZR7	DDX24	ATP-dependent RNA helicase DDX24
TLSKD	O00148	DX39A	ATP-dependent RNA helicase DDX39A
LLHNV	Q8TDD1	DDX54	ATP-dependent RNA helicase DDX54
CLTRT	Q8NDG6	TDRD9	ATP-dependent RNA helicase TDRD9
EEVLA	P08183	MDR1	ATP-dependent translocase ABCB1
FYDPL	P08183	MDR1	ATP-dependent translocase ABCB1
GKSTT	P08183	MDR1	ATP-dependent translocase ABCB1
VLDLK	P48048	KCNJ1	ATP-sensitive inward rectifier potassium channel 1
IIVIL	Q14654	KCJ11	ATP-sensitive inward rectifier potassium channel 11
VVSLS	Q99712	KCJ15	ATP-sensitive inward rectifier potassium channel 15
GLLLY	P16066	ANPRA	Atrial natriuretic peptide receptor 1 precursor
SLIND	P20594	ANPRB	Atrial natriuretic peptide receptor 2 precursor
VILLS	Q9Y5Q5	CORIN	Atrial natriuretic peptide-converting enzyme
AKKTN	P29374	ARI4A	AT-rich interactive domain-containing protein 4A
ELQLL	Q14865	ARI5B	AT-rich interactive domain-containing protein 5B
LSALR	Q8WXE1	ATRIP	ATR-interacting protein

AVVSL	Q8WXE1	ATRIP	ATR-interacting protein
ELLSL	Q8WXE1	ATRIP	ATR-interacting protein
KTKCT	O75882	ATRN	Attractin precursor
SLSNG	Q5VV63	ATRN1	Attractin-like protein 1 precursor
KTKCT	Q5VV63	ATRN1	Attractin-like protein 1 precursor
LGFLL	O00590	ACKR2	Atypical chemokine receptor 2
LGVGS	O75143	ATG13	Autophagy-related protein 13
GVGSA	O75143	ATG13	Autophagy-related protein 13
VSSSV	Q96BY7	ATG2B	Autophagy-related protein 2 homolog B
LIAVG	Q96BY7	ATG2B	Autophagy-related protein 2 homolog B
IAVGL	Q96BY7	ATG2B	Autophagy-related protein 2 homolog B
FLGFL	Q7Z3C6	ATG9A	Autophagy-related protein 9A
LSLIA	Q7Z3C6	ATG9A	Autophagy-related protein 9A
SDEFD	O15169	AXIN1	Axin-1
LSNIK	Q5T1B0	AXDN1	Axonemal dynein light chain domain-containing protein 1
LGFLL	P46663	BKRB1	B1 bradykinin receptor
LTLPS	Q13075	BIRC1	Baculoviral IAP repeat-containing protein 1
RGIIK	Q13490	BIRC2	Baculoviral IAP repeat-containing protein 2
TLSKK	Q9NR09	BIRC6	Baculoviral IAP repeat-containing protein 6
TNSEL	Q9NR09	BIRC6	Baculoviral IAP repeat-containing protein 6
VQLPL	Q9NR09	BIRC6	Baculoviral IAP repeat-containing protein 6
VTTPV	Q9H4G0	E41L1	Band 4.1-like protein 1
AIVSC	Q8TAM1	BBS10	Bardet-Biedl syndrome 10 protein

RLLEI	Q6ZT62	BGIN	Bargin
VGLLL	P50895	BCAM	Basal cell adhesion molecule precursor
SLGAI	Q68DE3	USF3	Basic helix-loop-helix domain-containing protein USF3
SLIAV	Q68DE3	USF3	Basic helix-loop-helix domain-containing protein USF3
KVLDL	Q86UB2	BIVM	Basic immunoglobulin-like variable motif-containing protein
SSVIT	Q9C0K0	BC11B	B-cell lymphoma/leukemia 11B
SVITS	Q9C0K0	BC11B	B-cell lymphoma/leukemia 11B
NITEE	Q6W2J9	BCOR	BCL-6 corepressor
QQSYS	Q6W2J9	BCOR	BCL-6 corepressor
ALLST	Q5H9F3	BCORL	BCL-6 corepressor-like protein 1
KKTNV	Q14457	BECN1	Beclin-1
KQELD	Q5T5X7	BEND3	BEN domain-containing protein 3
RRFLG	P08588	ADRB1	Beta-1 adrenergic receptor
TLPSE	Q9P109	GCNT4	Beta-1,3-galactosyl-O-glycosyl-glycoprotein beta-1,6-N-acetylglucosaminyltransferase 4
ELLSL	O96024	B3GT4	Beta-1,3-galactosyltransferase 4
LLSLI	A8MXE2	B3GT9	Beta-1,3-galactosyltransferase 9
MNSLT	O60512	B4GT3	Beta-1,4-galactosyltransferase 3
NEKIN	Q96KN2	CNDP1	Beta-Ala-His dipeptidase precursor
SKDQL	P42025	ACTY	Beta-centractin
KTNVT	P52757	CHIO	Beta-chimaerin
VKLIK	Q9H1M3	DB129	Beta-defensin 129 precursor
ELQLL	Q8IW92	GLBL2	Beta-galactosidase-1-like protein 2 precursor

QSTPP	Q8N3L3	TXLNB	Beta-taxilin
LSALR	A1A5D9	BICL2	BICD family-like cargo adapter 2
GKTKC	P52848	NDST1	Bifunctional heparan sulfate N-deacetylase/N-sulfotransferase 1
GKTKC	O95803	NDST3	Bifunctional heparan sulfate N-deacetylase/N-sulfotransferase 3
QELDK	Q9H3R1	NDST4	Bifunctional heparan sulfate N-deacetylase/N-sulfotransferase 4
TSKTD	Q9H3R1	NDST4	Bifunctional heparan sulfate N-deacetylase/N-sulfotransferase 4
GKTKC	Q9H3R1	NDST4	Bifunctional heparan sulfate N-deacetylase/N-sulfotransferase 4
VQLPL	P13995	MTDC	Bifunctional methylenetetrahydrofolate dehydrogenase/cyclohydrolase, mitochondrial precursor
AGKST	Q96T60	PNKP	Bifunctional polynucleotide phosphatase/kinase
GFLLG	O95342	ABCBB	Bile salt export pump
AGKST	O95342	ABCBB	Bile salt export pump
SAVSK	Q8NFC6	BD1L1	Biorientation of chromosomes in cell division protein 1-like 1
LSKKR	Q8NFC6	BD1L1	Biorientation of chromosomes in cell division protein 1-like 1
SKKRK	Q8NFC6	BD1L1	Biorientation of chromosomes in cell division protein 1-like 1
EEVLA	Q13867	BLMH	Bleomycin hydrolase
IKEEV	P11836	CD20	B-lymphocyte antigen CD20
LTNSE	O60477	BRNP1	BMP/retinoic acid-inducible neural-specific protein 1 precursor
SNKNR	O60477	BRNP1	BMP/retinoic acid-inducible neural-specific protein 1 precursor
IIVIL	Q10589	BST2	Bone marrow stromal antigen 2 precursor
NRARR	Q9NWK9	BCD1	Box C/D snoRNA protein 1
LTAVT	Q96DR5	BPIA2	BPI fold-containing family A member 2 precursor
NVTLS	Q8NFQ6	BPIFC	BPI fold-containing family C protein precursor

KKLMS	Q6UXY1	BI2L2	Brain-specific angiogenesis inhibitor 1-associated protein 2-like protein 2
GFLLG	Q6UWZ7	ABRX1	BRCA1-A complex subunit Abraxas 1
KRKRR	Q96RL1	UIMC1	BRCA1-A complex subunit RAP80
VGLLL	Q9P287	BCCIP	BRCA2 and CDKN1A-interacting protein
MLTNS	P11274	BCR	Breakpoint cluster region protein
LSALR	P38398	BRCA1	Breast cancer type 1 susceptibility protein
KRKRR	P38398	BRCA1	Breast cancer type 1 susceptibility protein
SDELL	P38398	BRCA1	Breast cancer type 1 susceptibility protein
IKEEV	P51587	BRCA2	Breast cancer type 2 susceptibility protein
KEEVL	P51587	BRCA2	Breast cancer type 2 susceptibility protein
EEVLA	P51587	BRCA2	Breast cancer type 2 susceptibility protein
TASNK	P51587	BRCA2	Breast cancer type 2 susceptibility protein
LTAVT	Q9Y6D6	BIG1	Brefeldin A-inhibited guanine nucleotide-exchange protein 1
LTAVT	Q9Y6D5	BIG2	Brefeldin A-inhibited guanine nucleotide-exchange protein 2
SALRT	Q9Y6D5	BIG2	Brefeldin A-inhibited guanine nucleotide-exchange protein 2
VSVLT	Q5TH69	BIG3	Brefeldin A-inhibited guanine nucleotide-exchange protein 3
GFLLG	Q15018	ABRX2	BRISC complex subunit Abraxas 2
GIIKT	Q9NRL2	BAZ1A	Bromodomain adjacent to zinc finger domain protein 1A
EEFYQ	Q9UIF9	BAZ2A	Bromodomain adjacent to zinc finger domain protein 2A
ALLST	Q9UIF9	BAZ2A	Bromodomain adjacent to zinc finger domain protein 2A
LTLPS	Q9UIF9	BAZ2A	Bromodomain adjacent to zinc finger domain protein 2A
KVLHL	Q9UIF8	BAZ2B	Bromodomain adjacent to zinc finger domain protein 2B

LLGVG	Q6RI45	BRWD3	Bromodomain and WD repeat-containing protein 3
ELLSL	Q6RI45	BRWD3	Bromodomain and WD repeat-containing protein 3
LLSLI	Q6RI45	BRWD3	Bromodomain and WD repeat-containing protein 3
VIDTP	Q6RI45	BRWD3	Bromodomain and WD repeat-containing protein 3
KKRKR	Q9H8M2	BRD9	Bromodomain-containing protein 9
TSKTD	Q9BWV1	BOC	Brother of CDO precursor
NSELL	Q9H0C5	BTBD1	BTB/POZ domain-containing protein 1
SELLS	Q9H0C5	BTBD1	BTB/POZ domain-containing protein 1
KSALL	Q96Q07	BTBD9	BTB/POZ domain-containing protein 9
KEEVL	Q96Q07	BTBD9	BTB/POZ domain-containing protein 9
SVNAG	Q719H9	KCTD1	BTB/POZ domain-containing protein KCTD1
SVNAG	Q96SI1	KCD15	BTB/POZ domain-containing protein KCTD15
LQLLM	O00481	BT3A1	Butyrophilin subfamily 3 member A1 precursor
SCSIS	A8MVZ5	BTNLA	Butyrophilin-like protein 10 precursor
STPPT	Q6UXE8	BTNL3	Butyrophilin-like protein 3 precursor
NVTLS	Q6UXE8	BTNL3	Butyrophilin-like protein 3 precursor
LMQST	P11586	C1TC	C-1-tetrahydrofolate synthase, cytoplasmic
VQLPL	P11586	C1TC	C-1-tetrahydrofolate synthase, cytoplasmic
GKSTT	P11586	C1TC	C-1-tetrahydrofolate synthase, cytoplasmic
SELLS	Q4AC94	C2CD3	C2 domain-containing protein 3
EEVLA	O75844	FACE1	CAAX prenyl protease 1 homolog
AVSKG	Q9NYQ6	CELR1	Cadherin EGF LAG seven-pass G-type receptor 1 precursor
MLTNS	Q9NYQ6	CELR1	Cadherin EGF LAG seven-pass G-type receptor 1 precursor

TDVSS	Q9NYQ6	CELR1	Cadherin EGF LAG seven-pass G-type receptor 1 precursor
GLLLY	Q9NYQ6	CELR1	Cadherin EGF LAG seven-pass G-type receptor 1 precursor
YSIMS	Q9HCU4	CELR2	Cadherin EGF LAG seven-pass G-type receptor 2 precursor
GLLLY	Q9HCU4	CELR2	Cadherin EGF LAG seven-pass G-type receptor 2 precursor
ELQLL	Q9NYQ7	CELR3	Cadherin EGF LAG seven-pass G-type receptor 3 precursor
ILLSL	Q9NYQ7	CELR3	Cadherin EGF LAG seven-pass G-type receptor 3 precursor
LLPIV	P55287	CAD11	Cadherin-11 precursor
IVILL	P55287	CAD11	Cadherin-11 precursor
LTSKV	P55289	CAD12	Cadherin-12 precursor
IVILL	P55289	CAD12	Cadherin-12 precursor
NRLLE	Q9UJ99	CAD22	Cadherin-22 precursor
TSVIT	Q9H251	CAD23	Cadherin-23 precursor
TDAKV	Q9H251	CAD23	Cadherin-23 precursor
DPLVF	Q9H251	CAD23	Cadherin-23 precursor
AGSVS	P55283	CADH4	Cadherin-4 precursor
GIIKT	P55285	CADH6	Cadherin-6 precursor
IVILL	P55285	CADH6	Cadherin-6 precursor
GIIKT	Q9ULB4	CADH9	Cadherin-9 precursor
TELQL	A4D0V7	CPED1	Cadherin-like and PC-esterase domain-containing protein 1 precursor
KGYLS	O43745	CHP2	Calcineurin B homologous protein 2
VGLLL	Q9UM00	TMCO1	Calcium load-activated calcium channel
SALLS	Q96D31	CRCM1	Calcium release-activated calcium channel protein 1

LTLPS	Q9NWR8	MCUB	Calcium uniporter regulatory subunit MCUb, mitochondrial precursor
KQLLP	Q8N5S9	KKCC1	Calcium/calmodulin-dependent protein kinase kinase 1
SNIKE	Q9UQC9	CLCA2	Calcium-activated chloride channel regulator 2 precursor
YTLNN	Q9UQC9	CLCA2	Calcium-activated chloride channel regulator 2 precursor
LTSKV	Q14CN2	CLCA4	Calcium-activated chloride channel regulator 4 precursor
DQKKL	Q13137	CACO2	Calcium-binding and coiled-coil domain-containing protein 2
IASGV	P98194	AT2C1	Calcium-transporting ATPase type 2C member 1
VLTSK	P98194	AT2C1	Calcium-transporting ATPase type 2C member 1
VVSLS	O75185	AT2C2	Calcium-transporting ATPase type 2C member 2
VSVLT	O75185	AT2C2	Calcium-transporting ATPase type 2C member 2
VITIE	Q8WWF8	CAPSL	Calcyphosin-like protein
EEVLA	Q7Z624	CMKMT	Calmodulin-lysine N-methyltransferase
EVLAY	Q7Z624	CMKMT	Calmodulin-lysine N-methyltransferase
LIKQE	Q5T5Y3	CAMP1	Calmodulin-regulated spectrin-associated protein 1
ARREL	Q5T5Y3	CAMP1	Calmodulin-regulated spectrin-associated protein 1
RARRE	Q08AD1	CAMP2	Calmodulin-regulated spectrin-associated protein 2
SELLS	Q9HC96	CAN10	Calpain-10
LLSLI	Q6ZSI9	CAN12	Calpain-12
VVSLS	O14958	CASQ2	Calsequestrin-2 precursor
VTELQ	Q9H4D0	CSTN2	Calsyntenin-2 precursor
LGVGS	Q9H4D0	CSTN2	Calsyntenin-2 precursor
NNAKK	P13861	KAP2	cAMP-dependent protein kinase type II-alpha regulatory subunit

NNAKK	P31323	KAP3	cAMP-dependent protein kinase type II-beta regulatory subunit
TYMLT	P27815	PDE4A	cAMP-specific 3',5'-cyclic phosphodiesterase 4A
TNSEL	P27815	PDE4A	cAMP-specific 3',5'-cyclic phosphodiesterase 4A
TNSEL	Q07343	PDE4B	cAMP-specific 3',5'-cyclic phosphodiesterase 4B
TNSEL	Q08493	PDE4C	cAMP-specific 3',5'-cyclic phosphodiesterase 4C
TNSEL	Q08499	PDE4D	cAMP-specific 3',5'-cyclic phosphodiesterase 4D
SIIKE	Q5HYN5	CT451	Cancer/testis antigen family 45 member A1
SIIKE	P0DMU9	CT45A	Cancer/testis antigen family 45 member A10
SIIKE	Q5DJT8	CT452	Cancer/testis antigen family 45 member A2
SIIKE	Q8NHU0	CT453	Cancer/testis antigen family 45 member A3
SIIKE	P0DMU8	CT455	Cancer/testis antigen family 45 member A5
SIIKE	P0DMU7	CT456	Cancer/testis antigen family 45 member A6
SIIKE	P0DMV0	CT457	Cancer/testis antigen family 45 member A7
SIIKE	P0DMV1	CT458	Cancer/testis antigen family 45 member A8
SIIKE	P0DMV2	CT459	Cancer/testis antigen family 45 member A9
TLSKK	Q8TC20	CAGE1	Cancer-associated gene 1 protein
EEVLA	Q8TC20	CAGE1	Cancer-associated gene 1 protein
TSPLC	P30622	CLIP1	CAP-Gly domain-containing linker protein 1
SPLCT	P30622	CLIP1	CAP-Gly domain-containing linker protein 1
FASGQ	Q96DZ5	CLIP3	CAP-Gly domain-containing linker protein 3
FASGQ	Q8N3C7	CLIP4	CAP-Gly domain-containing linker protein 4
LDLKN	Q8IYT2	CMTR2	Cap-specific mRNA (nucleoside-2'-O-)-methyltransferase 2
VSKVL	P31327	CPSM	Carbamoyl-phosphate synthase [ammonia], mitochondrial precursor

TLNNA	Q7LFX5	CHSTF	Carbohydrate sulfotransferase 15
ALRTG	Q9H2A9	CHST8	Carbohydrate sulfotransferase 8
TASNK	O43570	CAH12	Carbonic anhydrase 12 precursor
LLEIT	O75052	CAPON	Carboxyl-terminal PDZ ligand of neuronal nitric oxide synthase protein
IKTFS	Q96DG6	CMBL	Carboxymethylenebutenolidase homolog
ELLSL	P22792	CPN2	Carboxypeptidase N subunit 2 precursor
CSAVS	Q7Z692	CEA19	Carcinoembryonic antigen-related cell adhesion molecule 19 precursor
CTASN	Q6UY09	CEA20	Carcinoembryonic antigen-related cell adhesion molecule 20 precursor
DASIS	Q8N3K9	CMYA5	Cardiomyopathy-associated protein 5
CNVDI	Q92523	CPT1B	Carnitine O-palmitoyltransferase 1, muscle isoform
LSGIN	Q92523	CPT1B	Carnitine O-palmitoyltransferase 1, muscle isoform
TAVTF	Q8IUL8	CILP2	Cartilage intermediate layer protein 2 precursor
DKQLL	Q9H078	CLPB	Caseinolytic peptidase B protein homolog precursor
QKKLM	Q8WXD9	CSKI1	Caskin-1
QKKLM	Q8WXE0	CSKI2	Caskin-2
SIMSI	Q9BXL7	CAR11	Caspase recruitment domain-containing protein 11
AVGLL	Q9BXL6	CAR14	Caspase recruitment domain-containing protein 14
LVFPS	Q14790	CASP8	Caspase-8 precursor
VFPSD	Q14790	CASP8	Caspase-8 precursor
CSISN	P35221	CTNA1	Catenin alpha-1

CSISN	P26232	CTNA2	Catenin alpha-2
DTVSV	P07339	CATD	Cathepsin D precursor
KNRGI	P43235	CATK	Cathepsin K precursor
QSLAF	Q8NEC5	CTSR1	Cation channel sperm-associated protein 1
TLSKK	Q96P56	CTSR2	Cation channel sperm-associated protein 2
LSKKR	Q96P56	CTSR2	Cation channel sperm-associated protein 2
SKVLH	Q9H7T0	CTSRB	Cation channel sperm-associated protein subunit beta
SLAFI	Q9H7T0	CTSRB	Cation channel sperm-associated protein subunit beta
LGVGS	P52569	CTR2	Cationic amino acid transporter 2
LGVGS	Q8WY07	CTR3	Cationic amino acid transporter 3
LLGVG	O43246	CTR4	Cationic amino acid transporter 4
LLEIT	Q6NZI2	CAVN1	Caveolae-associated protein 1
AGSVS	Q96RK1	CITE4	Cbp/p300-interacting transactivator 4
TSVIT	P41597	CCR2	C-C chemokine receptor type 2
IVILL	P41597	CCR2	C-C chemokine receptor type 2
GIIKT	P51677	CCR3	C-C chemokine receptor type 3
VITSL	P51679	CCR4	C-C chemokine receptor type 4
TSVIT	P51681	CCR5	C-C chemokine receptor type 5
LIAVG	O00622	CCN1	CCN family member 1 precursor
SALRT	A5YKK6	CNOT1	CCR4-NOT transcription complex subunit 1
AGSVS	A5YKK6	CNOT1	CCR4-NOT transcription complex subunit 1
DKQLL	Q96LI5	CNO6L	CCR4-NOT transcription complex subunit 6-like
LSKKR	Q92600	CNOT9	CCR4-NOT transcription complex subunit 9

QSNRV	Q92600	CNOT9	CCR4-NOT transcription complex subunit 9
LTLPS	Q6YHK3	CD109	CD109 antigen precursor
VGLLL	Q13740	CD166	CD166 antigen precursor
TLPSE	O95400	CD2B2	CD2 antigen cytoplasmic tail-binding protein 2
ITTII	Q15762	CD226	CD226 antigen precursor
LIAVG	P08962	CD63	CD63 antigen
LIAVG	P60033	CD81	CD81 antigen
YVKGE	Q8TCZ2	C99L2	CD99 antigen-like protein 2 precursor
VSSSV	Q9H3Q1	BORG4	Cdc42 effector protein 4
VITIE	Q86Y37	CACL1	CDK2-associated and cullin domain-containing protein 1
VSSSV	Q9NXV6	CARF	CDKN2A-interacting protein
TTILT	Q9BY67	CADM1	Cell adhesion molecule 1 precursor
VSSSV	Q8N126	CADM3	Cell adhesion molecule 3 precursor
GAIVS	Q4KMG0	CDON	Cell adhesion molecule-related/down-regulated by oncogenes precursor
SKKRK	Q8N163	CCAR2	Cell cycle and apoptosis regulator protein 2
IKEEV	Q8N163	CCAR2	Cell cycle and apoptosis regulator protein 2
SDEFD	Q6WBX8	RAD9B	Cell cycle checkpoint control protein RAD9B
CSISN	O75943	RAD17	Cell cycle checkpoint protein RAD17
SISNI	O75943	RAD17	Cell cycle checkpoint protein RAD17
FLLGV	Q9NV96	CC50A	Cell cycle control protein 50A
YLSAL	Q9UHD4	CIDEB	Cell death activator CIDE-B
KKRKR	Q99459	CDC5L	Cell division cycle 5-like protein

NAGKS	Q86Y33	CD20B	Cell division cycle protein 20 homolog B
TELQL	Q9UJX2	CDC23	Cell division cycle protein 23 homolog
NIKEN	Q96GN5	CDA7L	Cell division cycle-associated 7-like protein
IKENK	Q96GN5	CDA7L	Cell division cycle-associated 7-like protein
KKRKR	Q9NX58	LYAR	Cell growth-regulating nucleolar protein
KRKRR	Q9NX58	LYAR	Cell growth-regulating nucleolar protein
CTASN	P43121	MUC18	Cell surface glycoprotein MUC18 precursor
FPSDE	Q9UHN6	CEIP2	Cell surface hyaluronidase
NAKKT	Q9NXG0	CNTLN	Centlein
IKENK	Q6IQ19	CCSAP	Centriole, cilia and spindle-associated protein
LIKQE	Q7Z7A1	CNTRL	Centriolin
TDVSS	Q03188	CENPC	Centromere protein C
ELQLL	P49454	CENPF	Centromere protein F precursor
NSLTL	P49454	CENPF	Centromere protein F precursor
EKINQ	P49454	CENPF	Centromere protein F precursor
SKDQL	P49454	CENPF	Centromere protein F precursor
SALLS	Q92674	CENPI	Centromere protein I
VLTSK	Q9HC77	CENPJ	Centromere protein J
ELQLL	Q02224	CENPE	Centromere-associated protein E precursor
KRKRR	Q02224	CENPE	Centromere-associated protein E precursor
EFQQK	Q02224	CENPE	Centromere-associated protein E precursor
VILLS	Q02224	CENPE	Centromere-associated protein E precursor
GQNIT	Q8N8E3	CE112	Centrosomal protein of 112 kDa

SEINL	Q8N960	CE120	Centrosomal protein of 120 kDa
LSALR	Q66GS9	CP135	Centrosomal protein of 135 kDa
KEEVL	Q66GS9	CP135	Centrosomal protein of 135 kDa
SDELL	Q66GS9	CP135	Centrosomal protein of 135 kDa
DKQLL	O94986	CE152	Centrosomal protein of 152 kDa
SQVNE	Q5TB80	CE162	Centrosomal protein of 162 kDa
ELLHN	Q9Y4F5	C170B	Centrosomal protein of 170 kDa protein B
NVTLS	Q8TEP8	CE192	Centrosomal protein of 192 kDa
IRKSD	Q8TEP8	CE192	Centrosomal protein of 192 kDa
KEEVL	O15078	CE290	Centrosomal protein of 290 kDa
QVNEK	O15078	CE290	Centrosomal protein of 290 kDa
ENKCN	Q86XR8	CEP57	Centrosomal protein of 57 kDa
AGKST	Q86XR8	CEP57	Centrosomal protein of 57 kDa
SCSIS	Q76N32	CEP68	Centrosomal protein of 68 kDa
EITRE	Q96ST8	CEP89	Centrosomal protein of 89 kDa
AVVSL	Q1MSJ5	CSPP1	Centrosome and spindle pole-associated protein 1
IVRQQ	Q5VT06	CE350	Centrosome-associated protein 350
LSALR	Q9BV73	CP250	Centrosome-associated protein CEP250
HLEGE	Q9BV73	CP250	Centrosome-associated protein CEP250
ELLSL	Q9BV73	CP250	Centrosome-associated protein CEP250
EEVLA	Q9BV73	CP250	Centrosome-associated protein CEP250
SSSVI	Q96MC4	C295L	CEP295 N-terminal-like protein
FPQAE	Q6ZMG9	CERS6	Ceramide synthase 6

KEEVL	Q5TA50	CPTP	Ceramide-1-phosphate transfer protein
VGLLL	Q13370	PDE3B	cGMP-inhibited 3',5'-cyclic phosphodiesterase B
AGKST	Q8WWZ7	ABCA5	Cholesterol transporter ABCA5
SIIKE	P35790	CHKA	Choline kinase alpha
IIKEE	P35790	CHKA	Choline kinase alpha
VITSL	Q53GD3	CTL4	Choline transporter-like protein 4
ASISQ	Q53GD3	CTL4	Choline transporter-like protein 4
GKSLY	Q8NCS7	CTL5	Choline transporter-like protein 5
VNAGV	Q8TDX6	CGAT1	Chondroitin sulfate N-acetylgalactosaminyltransferase 1
FLLGV	Q9ULG1	INO80	Chromatin-remodeling ATPase INO80
LLSLI	O14647	CHD2	Chromodomain-helicase-DNA-binding protein 2
ILLSL	O14647	CHD2	Chromodomain-helicase-DNA-binding protein 2
KQELD	Q12873	CHD3	Chromodomain-helicase-DNA-binding protein 3
KKRKR	Q12873	CHD3	Chromodomain-helicase-DNA-binding protein 3
KQELD	Q14839	CHD4	Chromodomain-helicase-DNA-binding protein 4
KENKC	Q8TDI0	CHD5	Chromodomain-helicase-DNA-binding protein 5
KQELD	Q8TDI0	CHD5	Chromodomain-helicase-DNA-binding protein 5
KEGSN	Q8TDI0	CHD5	Chromodomain-helicase-DNA-binding protein 5
VIDTP	Q9P2D1	CHD7	Chromodomain-helicase-DNA-binding protein 7
KKRKR	Q9HCK8	CHD8	Chromodomain-helicase-DNA-binding protein 8
VIDTP	Q9HCK8	CHD8	Chromodomain-helicase-DNA-binding protein 8
PVTLS	Q9HCK8	CHD8	Chromodomain-helicase-DNA-binding protein 8
VIDTP	Q3L8U1	CHD9	Chromodomain-helicase-DNA-binding protein 9

QVNEK	Q2VIQ3	KIF4B	Chromosome-associated kinesin KIF4B
NITEE	P23946	CMA1	Chymase precursor
TLSKK	Q8IYR0	CF206	Cilia- and flagella-associated protein 206
FPQAE	Q8IYR0	CF206	Cilia- and flagella-associated protein 206
TLSKK	Q4G0U5	PCDP1	Cilia- and flagella-associated protein 221
VKLIK	Q8NDM7	CFA43	Cilia- and flagella-associated protein 43
KLIKQ	Q8NDM7	CFA43	Cilia- and flagella-associated protein 43
SKKRK	Q8NDM7	CFA43	Cilia- and flagella-associated protein 43
EKINQ	Q8NDM7	CFA43	Cilia- and flagella-associated protein 43
LILKA	Q96MT7	CFA44	Cilia- and flagella-associated protein 44
TLSKK	Q96MT7	CFA44	Cilia- and flagella-associated protein 44
TSLGA	Q8IYW2	CFA46	Cilia- and flagella-associated protein 46
TTIII	Q6ZTR5	CFA47	Cilia- and flagella-associated protein 47
SGINN	Q6ZTR5	CFA47	Cilia- and flagella-associated protein 47
VTTPV	Q96N23	CFA54	Cilia- and flagella-associated protein 54
LSLIN	Q96N23	CFA54	Cilia- and flagella-associated protein 54
SVLTS	Q8NHU2	CFA61	Cilia- and flagella-associated protein 61
ITTII	A5D8W1	CFA69	Cilia- and flagella-associated protein 69
LEGEV	Q9P2B7	CFA97	Cilia- and flagella-associated protein 97
ASNKN	Q6PI97	HOATZ	Cilia- and flagella-associated protein HOATZ
SNKNR	Q6PI97	HOATZ	Cilia- and flagella-associated protein HOATZ
ASGVA	H7BZ55	CRCC2	Ciliary rootlet coiled-coil protein 2
KDQLS	Q9P2M7	CING	Cingulin

SKVLD	Q0VF96	CGNL1	Cingulin-like protein 1
KDQLS	Q0VF96	CGNL1	Cingulin-like protein 1
FFPQA	Q9ULV3	CIZ1	Cip1-interacting zinc finger protein
TELQL	O14578	CTRO	Citron Rho-interacting kinase
LHLEG	O14578	CTRO	Citron Rho-interacting kinase
NRLLE	O14578	CTRO	Citron Rho-interacting kinase
AKVKL	Q9UPT6	JIP3	C-Jun-amino-terminal kinase-interacting protein 3
DQLSG	Q9UPT6	JIP3	C-Jun-amino-terminal kinase-interacting protein 3
VRQQS	Q8IZR5	CKLF4	CKLF-like MARVEL transmembrane domain-containing protein 4
QELDK	P58418	CLRN1	Clarin-1
LQLLM	Q8NHS4	CLHC1	Clathrin heavy chain linker domain-containing protein 1
ILLSL	Q8NHS1	CLDN2	Claudin domain-containing protein 2
GAIVS	O95832	CLD1	Claudin-1
ITTIL	O95500	CLD14	Claudin-14
ILTAV	O95500	CLD14	Claudin-14
LGFLL	O95500	CLD14	Claudin-14
LSLIA	P56750	CLD17	Claudin-17
ILLSL	Q8IUQ0	CLVS1	Clavesin-1
ILLSL	Q5SYC1	CLVS2	Clavesin-2
LLEIT	Q9C0C6	CIPC	CLOCK-interacting pacemaker
RELPR	O75153	CLU	Clustered mitochondria protein homolog
VGLLL	P10909	CLUS	Clusterin precursor
LTLPS	Q8IY22	CMIP	C-Maf-inducing protein

LGFLL	Q11203	SIAT6	CMP-N-acetylneuraminate-beta-1,4-galactoside alpha-2,3-sialyltransferase
KVLDL	Q16842	SIA4B	CMP-N-acetylneuraminate-beta-galactosamide-alpha-2,3-sialyltransferase 2
SVLTS	Q7Z401	MYCPP	C-myc promoter-binding protein
LGFLL	P00748	FA12	Coagulation factor XII precursor
GFLLG	P35606	COPB2	Coatomer subunit beta'
FLLGV	P35606	COPB2	Coatomer subunit beta'
INNIA	P27352	IF	Cobalamin binding intrinsic factor precursor
TSLGA	Q8IWY9	CDAN1	Codanin-1
IAVGL	Q8IWY9	CDAN1	Codanin-1
ASGVA	P23528	COF1	Cofilin-1
SGVAV	P23528	COF1	Cofilin-1
GVAVS	P23528	COF1	Cofilin-1
SELLS	Q8WVM7	STAG1	Cohesin subunit SA-1
NRLLE	Q8N3U4	STAG2	Cohesin subunit SA-2
SELLS	Q8N3U4	STAG2	Cohesin subunit SA-2
DKQLL	Q9UJ98	STAG3	Cohesin subunit SA-3
DVSSS	Q9UJ98	STAG3	Cohesin subunit SA-3
KAVVS	Q8TD31	CCHCR	Coiled-coil alpha-helical rod protein 1
AVVSL	Q8TD31	CCHCR	Coiled-coil alpha-helical rod protein 1
ITEEF	Q9P2K1	C2D2A	Coiled-coil and C2 domain-containing protein 2A
NQSLA	Q8TC90	CCER1	Coiled-coil domain-containing glutamate-rich protein 1

VGLLL	Q8WV48	CC107	Coiled-coil domain-containing protein 107 precursor
KRKRR	Q6ZUS5	CC121	Coiled-coil domain-containing protein 121
ELQLL	Q96JN2	CC136	Coiled-coil domain-containing protein 136
ARSTP	Q49A88	CCD14	Coiled-coil domain-containing protein 14
KYKNA	Q6ZUS6	CC149	Coiled-coil domain-containing protein 149
TSLGA	Q5M9N0	CD158	Coiled-coil domain-containing protein 158
KRRFL	P0CW27	CC166	Coiled-coil domain-containing protein 166
ILKAN	Q8NDH2	CC168	Coiled-coil domain-containing protein 168
KQLLP	Q8NDH2	CC168	Coiled-coil domain-containing protein 168
NVNAG	Q8NDH2	CC168	Coiled-coil domain-containing protein 168
KKLMS	Q8IYT3	CC170	Coiled-coil domain-containing protein 170
ELSNI	P0C221	CC175	Coiled-coil domain-containing protein 175
RARRE	Q9NQR7	CC177	Coiled-coil domain-containing protein 177
VTTPV	Q5BJE1	CC178	Coiled-coil domain-containing protein 178
SSSVI	Q5BJE1	CC178	Coiled-coil domain-containing protein 178
IKENK	Q7Z3E2	CC186	Coiled-coil domain-containing protein 186
SELLS	Q7Z3E2	CC186	Coiled-coil domain-containing protein 186
SLIND	Q7Z3E2	CC186	Coiled-coil domain-containing protein 186
SALRT	A0A096LP49	CC187	Coiled-coil domain-containing protein 187
AVGLL	A0A096LP49	CC187	Coiled-coil domain-containing protein 187
ANAIT	P0DO97	CC192	Coiled-coil domain-containing protein 192
INQSL	Q5VVM6	CCD30	Coiled-coil domain-containing protein 30
LTRTD	Q4G0X9	CCD40	Coiled-coil domain-containing protein 40

RELPR	Q2TAC2	CCD57	Coiled-coil domain-containing protein 57
ELQLL	Q6P9F0	CCD62	Coiled-coil domain-containing protein 62
TNSEL	Q6P9F0	CCD62	Coiled-coil domain-containing protein 62
PTNNR	Q96M83	CCDC7	Coiled-coil domain-containing protein 7
RGIIK	Q96M83	CCDC7	Coiled-coil domain-containing protein 7
QKKLM	Q6NSX1	CCD70	Coiled-coil domain-containing protein 70 precursor
KNNRL	Q6ZRK6	CCD73	Coiled-coil domain-containing protein 73
NEKIN	Q6ZRK6	CCD73	Coiled-coil domain-containing protein 73
GVAVS	Q9BR77	CCD77	Coiled-coil domain-containing protein 77
SVITI	Q76M96	CCD80	Coiled-coil domain-containing protein 80 precursor
LLGVG	Q76M96	CCD80	Coiled-coil domain-containing protein 80 precursor
ELQLL	A6NC98	CC88B	Coiled-coil domain-containing protein 88B
ASGVA	A6NC98	CC88B	Coiled-coil domain-containing protein 88B
KKLMS	Q53HC0	CCD92	Coiled-coil domain-containing protein 92
KVLDL	P38432	COIL	Coilin
SGVAV	P02461	CO3A1	Collagen alpha-1(III) chain precursor
INLCN	Q99715	COCA1	Collagen alpha-1(XII) chain precursor
VKGEP	Q14993	COJA1	Collagen alpha-1(XIX) chain precursor
ASGVA	P39059	COFA1	Collagen alpha-1(XV) chain precursor
LPIVN	P39059	COFA1	Collagen alpha-1(XV) chain precursor
MELLI	Q9UMD9	COHA1	Collagen alpha-1(XVII) chain
ELLSL	Q9UMD9	COHA1	Collagen alpha-1(XVII) chain
STPPT	Q96A83	COQA1	Collagen alpha-1(XXVI) chain precursor

VKGEP	Q8IZC6	CORA1	Collagen alpha-1(XXVII) chain precursor
VKGEP	P08123	CO1A2	Collagen alpha-2(I) chain precursor
VKGEP	P05997	CO5A2	Collagen alpha-2(V) chain precursor
EEVLA	P25940	CO5A3	Collagen alpha-3(V) chain precursor
VTELQ	A8TX70	CO6A5	Collagen alpha-5(VI) chain precursor
KGEPI	Q14031	CO4A6	Collagen alpha-6(IV) chain precursor
KSDEL	A6NMZ7	CO6A6	Collagen alpha-6(VI) chain precursor
TNSEL	Q5KU26	COL12	Collectin-12
VSSSV	P23508	CRCM	Colorectal mutant cancer protein
VLHLE	Q5VWW1	C1QL3	Complement C1q-like protein 3 precursor
GKSLY	P01024	CO3	Complement C3 precursor
KSLYV	P01024	CO3	Complement C3 precursor
ELLHN	P01024	CO3	Complement C3 precursor
NVTLS	P0C0L4	CO4A	Complement C4-A precursor
AVSKV	P0C0L4	CO4A	Complement C4-A precursor
VSKVL	P0C0L4	CO4A	Complement C4-A precursor
NVTLS	P0C0L5	CO4B	Complement C4-B precursor
AVSKV	P0C0L5	CO4B	Complement C4-B precursor
VSKVL	P0C0L5	CO4B	Complement C4-B precursor
NYTLN	Q07021	C1QBP	Complement component 1 Q subcomponent-binding protein, mitochondrial precursor
YTLNN	P07358	CO8B	Complement component C8 beta chain precursor
LTSKV	P08174	DAF	Complement decay-accelerating factor precursor

RSTPV	P08174	DAF	Complement decay-accelerating factor precursor
KVKLI	Q9BPX3	CND3	Condensin complex subunit 3
SELLS	P42695	CNDD3	Condensin-2 complex subunit D3
IIVIL	Q86XI2	CNDG2	Condensin-2 complex subunit G2
LPIVN	P51160	PDE6C	Cone cGMP-specific 3',5'-cyclic phosphodiesterase subunit alpha' precursor
PIVNK	P51160	PDE6C	Cone cGMP-specific 3',5'-cyclic phosphodiesterase subunit alpha' precursor
GIIKT	Q9UP83	COG5	Conserved oligomeric Golgi complex subunit 5
LSNGV	P83436	COG7	Conserved oligomeric Golgi complex subunit 7
MNSLT	Q96MW5	COG8	Conserved oligomeric Golgi complex subunit 8
NSLTL	Q96MW5	COG8	Conserved oligomeric Golgi complex subunit 8
GYLSA	Q9NX05	F120C	Constitutive coactivator of PPAR-gamma-like protein 2
YLSAL	Q9NX05	F120C	Constitutive coactivator of PPAR-gamma-like protein 2
GNTLY	O94779	CNTN5	Contactin-5 precursor
DLKNY	Q13098	CSN1	COP9 signalosome complex subunit 1
TIELS	O75131	CPNE3	Copine-3
TNTKE	Q96A23	CPNE4	Copine-4
AGKST	O95741	CPNE6	Copine-6
NAGKS	Q9UBL6	CPNE7	Copine-7
AGKST	Q9UBL6	CPNE7	Copine-7
KQELD	Q04656	ATP7A	Copper-transporting ATPase 1
SLTLP	Q04656	ATP7A	Copper-transporting ATPase 1

VSLSN	P35670	ATP7B	Copper-transporting ATPase 2
EITRE	Q53SF7	COBL1	Cordon-bleu protein-like 1
TDVSS	Q53SF7	COBL1	Cordon-bleu protein-like 1
LILKA	O75367	H2AY	Core histone macro-H2A.1
SKKRK	Q86X95	CIR1	Corepressor interacting with RBPJ 1
LSGIN	Q86X95	CIR1	Corepressor interacting with RBPJ 1
KKLMS	Q92828	COR2A	Coronin-2A
SVITS	Q92828	COR2A	Coronin-2A
VSSSV	P10589	COT1	COUP transcription factor 1
SSSVI	P10589	COT1	COUP transcription factor 1
VSSSV	P24468	COT2	COUP transcription factor 2
SSSVI	P24468	COT2	COUP transcription factor 2
TKEGS	Q8N123	CPXCR	CPX chromosomal region candidate gene 1 protein
TSLGA	Q92793	CBP	CREB-binding protein
EGEVN	P46109	CRKL	Crk-like protein
FSVNA	Q49AN0	CRY2	Cryptochrome-2
SVNAG	Q49AN0	CRY2	Cryptochrome-2
VSSSV	Q5T3F8	CSCL2	CSC1-like protein 2
PVTLS	Q6ZS10	CL17A	C-type lectin domain family 17, member A
ALLST	Q96PZ7	CSMD1	CUB and sushi domain-containing protein 1 precursor
NSLTL	Q96PZ7	CSMD1	CUB and sushi domain-containing protein 1 precursor
SSSVI	Q96PZ7	CSMD1	CUB and sushi domain-containing protein 1 precursor
SSSVI	Q7Z407	CSMD3	CUB and sushi domain-containing protein 3

SLAFI	Q7Z407	CSMD3	CUB and sushi domain-containing protein 3
ETCKV	Q86UP6	CUZD1	CUB and zona pellucida-like domain-containing protein 1 precursor
SNICL	O60494	CUBN	Cubilin precursor
LSGIN	O60494	CUBN	Cubilin precursor
SVLTS	Q92879	CELF1	CUGBP Elav-like family member 1
MNSLT	O95319	CELF2	CUGBP Elav-like family member 2
SLTLP	Q8N6W0	CELF5	CUGBP Elav-like family member 5
LTNSE	Q13616	CUL1	Cullin-1
ILLSL	Q14999	CUL7	Cullin-7
SKVLD	Q8IWT3	CUL9	Cullin-9
FLGFL	P49238	CX3C1	CX3C chemokine receptor 1
LGFLL	P49238	CX3C1	CX3C chemokine receptor 1
FLLGV	P32302	CXCR5	C-X-C chemokine receptor type 5
KKRKR	Q9H2H0	CXXC4	CXXC-type zinc finger protein 4
KKRKR	Q7LFL8	CXXC5	CXXC-type zinc finger protein 5
SNGVS	P15336	ATF2	Cyclic AMP-dependent transcription factor ATF-2
TSVIT	P17544	ATF7	Cyclic AMP-dependent transcription factor ATF-7
KVLHL	Q68CJ9	CR3L3	Cyclic AMP-responsive element-binding protein 3-like protein 3
VLHLE	Q68CJ9	CR3L3	Cyclic AMP-responsive element-binding protein 3-like protein 3
SSSVI	Q02930	CREB5	Cyclic AMP-responsive element-binding protein 5
SSVIT	Q02930	CREB5	Cyclic AMP-responsive element-binding protein 5
IIKEE	Q8N884	CGAS	Cyclic GMP-AMP synthase
FPSDE	Q96M20	CNBD2	Cyclic nucleotide-binding domain-containing protein 2

STPVT	Q9NQW8	CNGB3	Cyclic nucleotide-gated cation channel beta-3
YVVQL	Q8IZL9	CDK20	Cyclin-dependent kinase 20
NAGSV	P46527	CDN1B	Cyclin-dependent kinase inhibitor 1B
AGKST	O76039	CDKL5	Cyclin-dependent kinase-like 5
KCTAS	Q16589	CCNG2	Cyclin-G2
ALLST	O14976	GAK	Cyclin-G-associated kinase
HLEGE	Q96S94	CCNL2	Cyclin-L2
LEGEV	Q96S94	CCNL2	Cyclin-L2
SCYGK	P21291	CSRP1	Cysteine and glycine-rich protein 1
SCYGK	Q16527	CSRP2	Cysteine and glycine-rich protein 2
RARRE	Q96S65	CSRN1	Cysteine/serine-rich nuclear protein 1
VKGEP	Q9H175	CSRN2	Cysteine/serine-rich nuclear protein 2
RVFCD	Q8WYN3	CSRN3	Cysteine/serine-rich nuclear protein 3
TTIII	Q9NS75	CLTR2	Cysteinyl leukotriene receptor 2
SLGAI	P13569	CFTR	Cystic fibrosis transmembrane conductance regulator
VNAGV	Q9BWV3	CDAC1	Cytidine and dCMP deaminase domain-containing protein 1
AIASG	P13498	CY24A	Cytochrome b-245 light chain
KNAVT	P47985	UCRI	Cytochrome b-c1 complex subunit Rieske, mitochondrial precursor
VLDLK	P47985	UCRI	Cytochrome b-c1 complex subunit Rieske, mitochondrial precursor
QEGKS	Q86WW8	COA5	Cytochrome c oxidase assembly factor 5
TPVST	Q5JTJ3	COA6	Cytochrome c oxidase assembly factor 6 homolog
ITTII	P00395	COX1	Cytochrome c oxidase subunit 1
GVAVS	P12074	CX6A1	Cytochrome c oxidase subunit 6A1, mitochondrial precursor

ELLSL	P04798	CP1A1	Cytochrome P450 1A1
TTILT	P33261	CP2CJ	Cytochrome P450 2C19
STPVT	Q02928	CP4AB	Cytochrome P450 4A11 precursor
STPVT	Q5TCH4	CP4AM	Cytochrome P450 4A22 precursor
SIIKE	P22680	CP7A1	Cytochrome P450 7A1
TTILT	P31785	IL2RG	Cytokine receptor common subunit gamma precursor
LLGVG	P31785	IL2RG	Cytokine receptor common subunit gamma precursor
TKEGS	Q7Z7G1	CLNK	Cytokine-dependent hematopoietic cell linker
KEGSN	Q7Z7G1	CLNK	Cytokine-dependent hematopoietic cell linker
SLAFI	Q14204	DYHC1	Cytoplasmic dynein 1 heavy chain 1
LEGEV	Q8NCM8	DYHC2	Cytoplasmic dynein 2 heavy chain 1
LKNYI	Q8NCM8	DYHC2	Cytoplasmic dynein 2 heavy chain 1
QSCSI	Q8NCM8	DYHC2	Cytoplasmic dynein 2 heavy chain 1
EEVLA	Q8NCM8	DYHC2	Cytoplasmic dynein 2 heavy chain 1
ICLTR	Q8NCM8	DYHC2	Cytoplasmic dynein 2 heavy chain 1
PSDEF	Q8NCM8	DYHC2	Cytoplasmic dynein 2 heavy chain 1
AGKST	Q8NCM8	DYHC2	Cytoplasmic dynein 2 heavy chain 1
IMTSK	Q8NE35	CPEB3	Cytoplasmic polyadenylation element-binding protein 3
SLSNG	O43639	NCK2	Cytoplasmic protein NCK2
NTKEG	Q14008	CKAP5	Cytoskeleton-associated protein 5
FASGQ	P28838	AMPL	Cytosol aminopeptidase
ASGQN	P28838	AMPL	Cytosol aminopeptidase
LQLLM	Q9UPW5	CBPC1	Cytosolic carboxypeptidase 1

LMSNN	Q9UPW5	CBPC1	Cytosolic carboxypeptidase 1
LLPIV	Q8NEM8	CBPC3	Cytosolic carboxypeptidase 3
LSGIN	Q8NEM8	CBPC3	Cytosolic carboxypeptidase 3
SGINN	Q8NEM8	CBPC3	Cytosolic carboxypeptidase 3
RRFLG	Q5VU57	CBPC6	Cytosolic carboxypeptidase 6
RELPR	Q8NFI3	ENASE	Cytosolic endo-beta-N-acetylglucosaminidase
VSLSN	P47712	PA24A	Cytosolic phospholipase A2
REFSV	Q5M775	CYTSB	Cytospin-B
SSSVI	P21728	DRD1	D(1A) dopamine receptor
CSISN	P35462	DRD3	D(3) dopamine receptor
QELDK	Q9NYF0	DACT1	Dapper homolog 1
SKVLH	Q9HCK1	ZDBF2	DBF4-type zinc finger-containing protein 2
LSKKR	Q9UKG1	DP13A	DCC-interacting protein 13-alpha
KARST	Q5VW00	DC122	DDB1- and CUL4-associated factor 12-like protein 2
TVSVG	Q66K64	DCA15	DDB1- and CUL4-associated factor 15
LSALR	Q96JK2	DCAF5	DDB1- and CUL4-associated factor 5
STPPT	Q96JK2	DCAF5	DDB1- and CUL4-associated factor 5
AGSVS	A6NGE4	DC8L1	DDB1- and CUL4-associated factor 8-like protein 1
VITIE	Q6ZMT9	DTHD1	Death domain-containing protein 1
LHLEG	Q6ZMT9	DTHD1	Death domain-containing protein 1
ETVIE	Q96BY6	DOC10	Dedicator of cytokinesis protein 10
CNVDI	Q5JSL3	DOC11	Dedicator of cytokinesis protein 11
VGLLL	Q5JSL3	DOC11	Dedicator of cytokinesis protein 11

LTLPS	Q92608	DOCK2	Dedicator of cytokinesis protein 2
IINFY	Q92608	DOCK2	Dedicator of cytokinesis protein 2
LLLYC	Q8IZD9	DOCK3	Dedicator of cytokinesis protein 3
DQLSG	Q9H7D0	DOCK5	Dedicator of cytokinesis protein 5
ASISQ	Q96HP0	DOCK6	Dedicator of cytokinesis protein 6
MNSLT	Q96N67	DOCK7	Dedicator of cytokinesis protein 7
GSVSF	Q8NF50	DOCK8	Dedicator of cytokinesis protein 8
LSKDQ	Q8NF50	DOCK8	Dedicator of cytokinesis protein 8
TILTA	Q9BZ29	DOCK9	Dedicator of cytokinesis protein 9
VGLLL	Q9BZ29	DOCK9	Dedicator of cytokinesis protein 9
LSALR	Q01523	DEF5	Defensin-5 precursor
SALRT	Q01523	DEF5	Defensin-5 precursor
TILTA	Q01524	DEF6	Defensin-6 precursor
ILTAV	Q01524	DEF6	Defensin-6 precursor
KEEVL	Q96LJ7	DHRS1	Dehydrogenase/reductase SDR family member 1
GIIKT	Q13268	DHRS2	Dehydrogenase/reductase SDR family member 2, mitochondrial precursor
VSKGY	Q9NQZ3	DAZ1	Deleted in azoospermia protein 1
VSKGY	Q13117	DAZ2	Deleted in azoospermia protein 2
VSKGY	Q9NR90	DAZ3	Deleted in azoospermia protein 3
VSKGY	Q86SG3	DAZ4	Deleted in azoospermia protein 4
VSKGY	Q92904	DAZL	Deleted in azoospermia-like
ASGVA	Q13011	ECH1	Delta(3,5)-Delta(2,4)-dienoyl-CoA isomerase, mitochondrial

			precursor
AIASG	P54886	P5CS	Delta-1-pyrroline-5-carboxylate synthase
AVGLL	P41143	OPRD	Delta-type opioid receptor
HTSPL	Q8TEH3	DEN1A	DENN domain-containing protein 1A
SKVLD	P78524	DEN2B	DENN domain-containing protein 2B
SELLS	P78524	DEN2B	DENN domain-containing protein 2B
AVGLL	O75064	DEN4B	DENN domain-containing protein 4B
VGLLL	O75064	DEN4B	DENN domain-containing protein 4B
VQLPL	Q5VZ89	DEN4C	DENN domain-containing protein 4C
AGKST	P27707	DCK	Deoxycytidine kinase
TIELS	Q5TB30	DEP1A	DEP domain-containing protein 1A
LQLLM	Q5TB30	DEP1A	DEP domain-containing protein 1A
LQLLM	Q8WUY9	DEP1B	DEP domain-containing protein 1B
VITSL	Q8IZU8	DSEL	Dermatan-sulfate epimerase-like protein precursor
IAVGL	Q8IZU8	DSEL	Dermatan-sulfate epimerase-like protein precursor
STPVT	P32926	DSG3	Desmoglein-3 precursor
SDELL	Q7L5Y6	DET1	DET1 homolog
QNITE	Q96JH7	VCIP1	Deubiquitinating protein VCPIP1
LEGEV	Q7Z7J5	DPPA2	Developmental pluripotency-associated protein 2
TSLGA	Q7Z3D6	GLUCM	D-glutamate cyclase, mitochondrial precursor
LDLKN	Q9Y4D2	DGLA	Diacylglycerol lipase-alpha
KNAVT	Q96PD7	DGAT2	Diacylglycerol O-acyltransferase 2
ITSLG	Q14194	DPYL1	Dihydropyrimidinase-related protein 1

ITSLG	Q16555	DPYL2	Dihydropyrimidinase-related protein 2
IIVII	Q16832	DDR2	Discoidin domain-containing receptor 2 precursor
GFLLG	Q14689	DIP2A	Disco-interacting protein 2 homolog A
GFLLG	Q9P265	DIP2B	Disco-interacting protein 2 homolog B
VKGEP	Q9P265	DIP2B	Disco-interacting protein 2 homolog B
GFLLG	Q9Y2E4	DIP2C	Disco-interacting protein 2 homolog C
SIIKE	Q99965	ADAM2	Disintegrin and metalloproteinase domain-containing protein 2 precursor
SKTDV	Q99965	ADAM2	Disintegrin and metalloproteinase domain-containing protein 2 precursor
KTDVS	Q99965	ADAM2	Disintegrin and metalloproteinase domain-containing protein 2 precursor
SLSNG	O75077	ADA23	Disintegrin and metalloproteinase domain-containing protein 23 precursor
DASIS	Q9UKQ2	ADA28	Disintegrin and metalloproteinase domain-containing protein 28 precursor
AGSVS	Q9UKF2	ADA30	Disintegrin and metalloproteinase domain-containing protein 30 precursor
SNIET	Q9H2U9	ADAM7	Disintegrin and metalloproteinase domain-containing protein 7 precursor
NGTDA	Q12959	DLG1	Disks large homolog 1
EITRE	Q12959	DLG1	Disks large homolog 1
SGQNI	Q8TDM6	DLG5	Disks large homolog 5

QVNEK	Q8TDM6	DLG5	Disks large homolog 5
KDQLS	Q969H9	DIRC1	Disrupted in renal carcinoma protein 1
FPSDE	Q8NDZ4	DIK2A	Divergent protein kinase domain 2A precursor
ILTAV	Q9Y485	DMXL1	DmX-like protein 1
SKGYL	Q5FWF4	ZRAB3	DNA annealing helicase and endonuclease ZRANB3
LLPIV	Q5FWF4	ZRAB3	DNA annealing helicase and endonuclease ZRANB3
EFDAS	Q6PJP8	DCR1A	DNA cross-link repair 1A protein
YLSAL	Q8IXT1	DDIAS	DNA damage-induced apoptosis suppressor protein
QSYSI	Q8IXT1	DDIAS	DNA damage-induced apoptosis suppressor protein
EVNKI	Q99708	CTIP	DNA endonuclease RBBP8
TSLGA	Q2NKX8	ERC6L	DNA excision repair protein ERCC-6-like
GLLLY	Q2NKX8	ERC6L	DNA excision repair protein ERCC-6-like
TPVTL	O00273	DFFA	DNA fragmentation factor subunit alpha
LLHNV	O76075	DFFB	DNA fragmentation factor subunit beta
ETCKV	Q8NG08	HELB	DNA helicase B
VTLSK	Q9UJA3	MCM8	DNA helicase MCM8
SALLS	Q9UJA3	MCM8	DNA helicase MCM8
TLSKD	Q9UJA3	MCM8	DNA helicase MCM8
LLILK	Q9NXL9	MCM9	DNA helicase MCM9
GLLLY	P04053	TDT	DNA nucleotidylexotransferase
RARRE	P09884	DPOLA	DNA polymerase alpha catalytic subunit
QKKLM	Q07864	DPOE1	DNA polymerase epsilon catalytic subunit A
VNKQS	Q9UBT6	POLK	DNA polymerase kappa

VSSSV	P54098	DPOG1	DNA polymerase subunit gamma-1
ELLIL	O75417	DPOLQ	DNA polymerase theta
LLILK	O75417	DPOLQ	DNA polymerase theta
NVDIF	O75417	DPOLQ	DNA polymerase theta
FIRKS	P49642	PRI1	DNA primase small subunit
VAVSK	Q9Y620	RA54B	DNA repair and recombination protein RAD54B
KSTTN	Q92878	RAD50	DNA repair protein RAD50
STPVT	P43351	RAD52	DNA repair protein RAD52 homolog
GLLLY	P51530	DNA2	DNA replication ATP-dependent helicase/nuclease DNA2
LSKDQ	P49736	MCM2	DNA replication licensing factor MCM2
KRKRR	P25205	MCM3	DNA replication licensing factor MCM3
VNKIK	P11388	TOP2A	DNA topoisomerase 2-alpha
PITND	Q02880	TOP2B	DNA topoisomerase 2-beta
FLGFL	Q9H5L6	THAP9	DNA transposase THAP9
LGFLL	Q9H5L6	THAP9	DNA transposase THAP9
TLNNA	Q8HWS3	RFX6	DNA-binding protein RFX6
NKQEG	Q2KHR2	RFX7	DNA-binding protein RFX7
VGLLL	O14593	RFXK	DNA-binding protein RFXANK
MSNNV	Q01826	SATB1	DNA-binding protein SATB1
VVQLP	Q9UPW6	SATB2	DNA-binding protein SATB2
AGVTT	P38935	SMBP2	DNA-binding protein SMUBP-2
ELLSL	P78527	PRKDC	DNA-dependent protein kinase catalytic subunit
AVGLL	O95602	RPA1	DNA-directed RNA polymerase I subunit RPA1

AGVTT	Q9H9Y6	RPA2	DNA-directed RNA polymerase I subunit RPA2
DTVSV	Q9H9Y6	RPA2	DNA-directed RNA polymerase I subunit RPA2
KKRKR	O15446	RPA34	DNA-directed RNA polymerase I subunit RPA34
LIKQE	P05423	RPC4	DNA-directed RNA polymerase III subunit RPC4
VQLPL	P05423	RPC4	DNA-directed RNA polymerase III subunit RPC4
SALLS	O75575	RPC9	DNA-directed RNA polymerase III subunit RPC9
YYVNK	Q8TBM8	DJB14	DnaJ homolog subfamily B member 14
FLGFL	Q6Y2X3	DJC14	DnaJ homolog subfamily C member 14
SLIAV	Q9Y5T4	DJC15	DnaJ homolog subfamily C member 15
LIAVG	Q9Y5T4	DJC15	DnaJ homolog subfamily C member 15
IAVGL	Q9Y5T4	DJC15	DnaJ homolog subfamily C member 15
LLSLI	Q9Y2G8	DJC16	DnaJ homolog subfamily C member 16 precursor
AFIRK	Q5F1R6	DJC21	DnaJ homolog subfamily C member 21
RARRE	Q96LL9	DJC30	DnaJ homolog subfamily C member 30, mitochondrial precursor
SKKRK	Q8N7S2	DNJ5G	DnaJ homolog subfamily C member 5G
VLDLK	Q8N7S2	DNJ5G	DnaJ homolog subfamily C member 5G
SVLTS	O75937	DNJC8	DnaJ homolog subfamily C member 8
VLTSK	O75937	DNJC8	DnaJ homolog subfamily C member 8
LSALR	Q9P104	DOK5	Docking protein 5
AKVKL	Q6PKX4	DOK6	Docking protein 6
VSKGY	O60762	DPM1	Dolichol-phosphate mannosyltransferase subunit 1
IAFSN	Q8TCJ2	STT3B	Dolichyl-diphosphooligosaccharide--protein glycosyltransferase subunit STT3B

ILLSL	Q14183	DOC2A	Double C2-like domain-containing protein alpha
KQLLP	M0R2J8	DCDC1	Doublecortin domain-containing protein 1
SCSIS	M0R2J8	DCDC1	Doublecortin domain-containing protein 1
ELQLL	Q96SC8	DMTA2	Doublesex- and mab-3-related transcription factor A2
GYLSA	Q96MA1	DMRTB	Doublesex- and mab-3-related transcription factor B1
YLSAL	Q96MA1	DMRTB	Doublesex- and mab-3-related transcription factor B1
TPVTL	Q5HYR2	DMRTC	Doublesex- and mab-3-related transcription factor C1
DIFNP	P49959	MRE11	Double-strand break repair protein MRE11
TSKTD	P49959	MRE11	Double-strand break repair protein MRE11
NNRLL	O60216	RAD21	Double-strand-break repair protein rad21 homolog
NEKIN	O60216	RAD21	Double-strand-break repair protein rad21 homolog
LSKKR	Q9NUL3	STAU2	Double-stranded RNA-binding protein Staufen homolog 2
ISNIE	P78563	RED1	Double-stranded RNA-specific editase 1
SIIKE	A4D126	ISPD	D-ribitol-5-phosphate cytidylyltransferase
SNGCD	Q9UN19	DAPP1	Dual adapter for phosphotyrosine and 3-phosphotyrosine and 3-phosphoinositide
SALRT	Q1HG44	DOXA2	Dual oxidase maturation factor 2
AVVSL	Q1HG44	DOXA2	Dual oxidase maturation factor 2
LSALR	P49761	CLK3	Dual specificity protein kinase CLK3
KRKRR	Q9HAZ1	CLK4	Dual specificity protein kinase CLK4
LSNGV	Q9H596	DUS21	Dual specificity protein phosphatase 21
IAVGL	Q96S53	TESK2	Dual specificity testis-specific protein kinase 2
SALRT	Q9Y463	DYR1B	Dual specificity tyrosine-phosphorylation-regulated kinase 1B

ASGVA	Q7RTS9	DYM	Dymeclin
NSELL	Q9UQ16	DYN3	Dynamin-3
TPVST	Q6XZF7	DNMBP	Dynamin-binding protein
NVTLS	Q96MX6	DAA10	Dynein axonemal assembly factor 10
AGSVS	Q96MX6	DAA10	Dynein axonemal assembly factor 10
NAGSV	Q5TEA3	DAAF9	Dynein axonemal assembly factor 9
SAVSK	Q9P2D7	DYH1	Dynein axonemal heavy chain 1
LSKKR	Q9P2D7	DYH1	Dynein axonemal heavy chain 1
SKKRK	Q9P2D7	DYH1	Dynein axonemal heavy chain 1
LLGVG	Q9P2D7	DYH1	Dynein axonemal heavy chain 1
NAGVT	Q9P2D7	DYH1	Dynein axonemal heavy chain 1
AGVTT	Q9P2D7	DYH1	Dynein axonemal heavy chain 1
FFPQA	Q9P2D7	DYH1	Dynein axonemal heavy chain 1
KINQS	Q9P2D7	DYH1	Dynein axonemal heavy chain 1
TILTA	Q8IVF4	DYH10	Dynein axonemal heavy chain 10
LDLKN	Q8IVF4	DYH10	Dynein axonemal heavy chain 10
IINFY	Q8IVF4	DYH10	Dynein axonemal heavy chain 10
LLEIT	Q6ZR08	DYH12	Dynein axonemal heavy chain 12
ELLIL	Q0VDD8	DYH14	Dynein axonemal heavy chain 14
IKSAL	Q0VDD8	DYH14	Dynein axonemal heavy chain 14
FFPQA	Q0VDD8	DYH14	Dynein axonemal heavy chain 14
ALLST	Q9UFH2	DYH17	Dynein axonemal heavy chain 17
VITSL	Q9UFH2	DYH17	Dynein axonemal heavy chain 17

FIRKS	Q9UFH2	DYH17	Dynein axonemal heavy chain 17
IIVIL	Q9UFH2	DYH17	Dynein axonemal heavy chain 17
SNNVQ	Q9P225	DYH2	Dynein axonemal heavy chain 2
SELLS	Q8TE73	DYH5	Dynein axonemal heavy chain 5
TLPSE	Q8TE73	DYH5	Dynein axonemal heavy chain 5
VGLLL	Q9C0G6	DYH6	Dynein axonemal heavy chain 6
RELPR	Q96JB1	DYH8	Dynein axonemal heavy chain 8
LSLIN	Q96JB1	DYH8	Dynein axonemal heavy chain 8
SLIND	Q96JB1	DYH8	Dynein axonemal heavy chain 8
AVTEL	Q6TDU7	DNAI7	Dynein axonemal intermediate chain 7
LILKA	Q96M86	DNHD1	Dynein heavy chain domain-containing protein 1
LGAIV	Q96M86	DNHD1	Dynein heavy chain domain-containing protein 1
KARST	Q9NP97	DLRB1	Dynein light chain roadblock-type 1
INDMP	Q8NEE6	DRC6	Dynein regulatory complex subunit 6
KVKLI	Q03001	DYST	Dystonin
SALLS	Q03001	DYST	Dystonin
TSKVL	Q03001	DYST	Dystonin
KKLMS	Q03001	DYST	Dystonin
EEVLA	Q03001	DYST	Dystonin
LSLIA	Q03001	DYST	Dystonin
AKVKL	P11532	DMD	Dystrophin
IDKQL	P11532	DMD	Dystrophin
TPVTL	P11532	DMD	Dystrophin

LILKA	Q96L91	EP400	E1A-binding protein p400
SVGNT	Q96L91	EP400	E1A-binding protein p400
TPVTL	Q96L91	EP400	E1A-binding protein p400
KEEVL	Q56P03	EAPP	E2F-associated phosphoprotein
DELLH	Q9UII4	HERC5	E3 ISG15--protein ligase HERC5
PVTLS	P11161	EGR2	E3 SUMO-protein ligase EGR2
IRKSD	Q96MF7	NSE2	E3 SUMO-protein ligase NSE2
SVITS	Q9Y6X2	PIAS3	E3 SUMO-protein ligase PIAS3
VITSL	Q9Y6X2	PIAS3	E3 SUMO-protein ligase PIAS3
FLLGV	P49792	RBP2	E3 SUMO-protein ligase RanBP2
KAVVS	P49792	RBP2	E3 SUMO-protein ligase RanBP2
GMDTV	P49792	RBP2	E3 SUMO-protein ligase RanBP2
CSAVS	O43734	CIKS	E3 ubiquitin ligase TRAF3IP2
DAKVK	Q8NHY2	COP1	E3 ubiquitin-protein ligase COP1
AKVKL	Q8NHY2	COP1	E3 ubiquitin-protein ligase COP1
NFYDP	Q86Y01	DTX1	E3 ubiquitin-protein ligase DTX1
IASGV	Q9Y2E6	DTX4	E3 ubiquitin-protein ligase DTX4
LTLPS	Q7Z6J8	UBE3D	E3 ubiquitin-protein ligase E3D
SELLS	Q8IYU2	HACE1	E3 ubiquitin-protein ligase HACE1
IKEEV	Q76N89	HECW1	E3 ubiquitin-protein ligase HECW1
VILLS	Q76N89	HECW1	E3 ubiquitin-protein ligase HECW1
ILLSL	Q76N89	HECW1	E3 ubiquitin-protein ligase HECW1
IKEEV	Q9P2P5	HECW2	E3 ubiquitin-protein ligase HECW2

LSALR	O95714	HERC2	E3 ubiquitin-protein ligase HERC2
VSSSV	O95714	HERC2	E3 ubiquitin-protein ligase HERC2
ILLSL	O95714	HERC2	E3 ubiquitin-protein ligase HERC2
NRLLE	Q7Z6Z7	HUWE1	E3 ubiquitin-protein ligase HUWE1
LLSLI	Q7Z6Z7	HUWE1	E3 ubiquitin-protein ligase HUWE1
EEVLA	Q7Z6Z7	HUWE1	E3 ubiquitin-protein ligase HUWE1
SLSNG	Q96J02	ITCH	E3 ubiquitin-protein ligase Itchy homolog
SALLS	O94822	LTN1	E3 ubiquitin-protein ligase listerin
KVLDL	Q6UWE0	LRSM1	E3 ubiquitin-protein ligase LRSAM1
IASGV	O60337	MARH6	E3 ubiquitin-protein ligase MARCHF6
AVSKV	Q86YT6	MIB1	E3 ubiquitin-protein ligase MIB1
PVTLS	Q96AX9	MIB2	E3 ubiquitin-protein ligase MIB2
LLEIT	O75592	MYCB2	E3 ubiquitin-protein ligase MYCBP2
SSSVI	O75592	MYCB2	E3 ubiquitin-protein ligase MYCBP2
GVAVS	Q6VVB1	NHLC1	E3 ubiquitin-protein ligase NHLRC1
YVKGE	Q8NG27	PJA1	E3 ubiquitin-protein ligase Praja-1
IKENK	Q9NS91	RAD18	E3 ubiquitin-protein ligase RAD18
SAVSK	Q7Z6E9	RBBP6	E3 ubiquitin-protein ligase RBBP6
LSALR	Q6PCD5	RFWD3	E3 ubiquitin-protein ligase RFWD3
SVSFF	Q8TEB7	RN128	E3 ubiquitin-protein ligase RNF128 precursor
LQLLM	Q8WU17	RN139	E3 ubiquitin-protein ligase RNF139
PLVFP	Q9H6Y7	RN167	E3 ubiquitin-protein ligase RNF167 precursor
SLGAI	Q6ZMZ0	RN19B	E3 ubiquitin-protein ligase RNF19B

LSALR	Q63HN8	RN213	E3 ubiquitin-protein ligase RNF213
SALRT	Q63HN8	RN213	E3 ubiquitin-protein ligase RNF213
GKSLY	Q63HN8	RN213	E3 ubiquitin-protein ligase RNF213
KSLYV	Q63HN8	RN213	E3 ubiquitin-protein ligase RNF213
SLYVK	Q63HN8	RN213	E3 ubiquitin-protein ligase RNF213
KEEVL	O76064	RNF8	E3 ubiquitin-protein ligase RNF8
AVVSL	Q8TEC5	SH3R2	E3 ubiquitin-protein ligase SH3RF2
VQLPL	Q8TEJ3	SH3R3	E3 ubiquitin-protein ligase SH3RF3
DVSSS	Q8TEJ3	SH3R3	E3 ubiquitin-protein ligase SH3RF3
KSDEL	Q8TEJ3	SH3R3	E3 ubiquitin-protein ligase SH3RF3
MELLI	Q149N8	SHPRH	E3 ubiquitin-protein ligase SHPRH
ILTAV	Q149N8	SHPRH	E3 ubiquitin-protein ligase SHPRH
LSGIN	Q149N8	SHPRH	E3 ubiquitin-protein ligase SHPRH
SVITI	Q9NS56	TOPRS	E3 ubiquitin-protein ligase Topors
SSVIT	Q9NS56	TOPRS	E3 ubiquitin-protein ligase Topors
EGEVN	Q8IYM9	TRI22	E3 ubiquitin-protein ligase TRIM22
ELLSL	P36406	TRI23	E3 ubiquitin-protein ligase TRIM23
AVGLL	Q13049	TRI32	E3 ubiquitin-protein ligase TRIM32
AIASG	Q9UPN9	TRI33	E3 ubiquitin-protein ligase TRIM33
TIELS	O94972	TRI37	E3 ubiquitin-protein ligase TRIM37
VTTPV	O94972	TRI37	E3 ubiquitin-protein ligase TRIM37
TPVST	Q86XT4	TRI50	E3 ubiquitin-protein ligase TRIM50
VVSLS	Q9BRZ2	TRI56	E3 ubiquitin-protein ligase TRIM56

VSLSN	Q9BRZ2	TRI56	E3 ubiquitin-protein ligase TRIM56
SLSNG	Q9BRZ2	TRI56	E3 ubiquitin-protein ligase TRIM56
SEINL	P53804	TTC3	E3 ubiquitin-protein ligase TTC3
IIKTF	Q8IWV8	UBR2	E3 ubiquitin-protein ligase UBR2
VILLS	Q6ZT12	UBR3	E3 ubiquitin-protein ligase UBR3
ILLSL	Q6ZT12	UBR3	E3 ubiquitin-protein ligase UBR3
NDMPI	Q5T4S7	UBR4	E3 ubiquitin-protein ligase UBR4
SIIKE	Q5T4S7	UBR4	E3 ubiquitin-protein ligase UBR4
YGKTK	Q5T4S7	UBR4	E3 ubiquitin-protein ligase UBR4
IVNKQ	Q9ULT6	ZNRF3	E3 ubiquitin-protein ligase ZNRF3 precursor
SLGAI	Q8WWF5	ZNRF4	E3 ubiquitin-protein ligase ZNRF4 precursor
LGAIV	Q8WWF5	ZNRF4	E3 ubiquitin-protein ligase ZNRF4 precursor
VTELQ	Q15075	EEA1	Early endosome antigen 1
NITEE	O00423	EMAL1	Echinoderm microtubule-associated protein-like 1
VLTSK	Q6ZMW3	EMAL6	Echinoderm microtubule-associated protein-like 6
NRLLE	O60447	EVI5	Ecotropic viral integration site 5 protein homolog
VGLLL	O60447	EVI5	Ecotropic viral integration site 5 protein homolog
CSAVS	O75356	ENTP5	Ectonucleoside triphosphate diphosphohydrolase 5 precursor
LEGEV	O75356	ENTP5	Ectonucleoside triphosphate diphosphohydrolase 5 precursor
KGEPI	P22413	ENPP1	Ectonucleotide pyrophosphatase/phosphodiesterase family member 1
VILLS	Q6UWV6	ENPP7	Ectonucleotide pyrophosphatase/phosphodiesterase family member 7 precursor

ASGQN	Q9HCE0	EPG5	Ectopic P granules protein 5 homolog
LLMQS	Q9HCE0	EPG5	Ectopic P granules protein 5 homolog
EGEVN	Q8N6R0	EFNMT	eEF1A lysine and N-terminal methyltransferase
LSNIK	Q5JPI9	EFMT2	EEF1A lysine methyltransferase 2
KGYLS	Q9BUY7	EFC11	EF-hand calcium-binding domain-containing protein 11
SPLCT	Q8IY85	EFC13	EF-hand calcium-binding domain-containing protein 13
SKDQL	A8K855	EFCB7	EF-hand calcium-binding domain-containing protein 7
KGMDT	Q8N7U6	EFHB	EF-hand domain-containing family member B
TMNSL	O95967	FBLN4	EGF-containing fibulin-like extracellular matrix protein 2 precursor
LQLLM	Q8N3D4	EH1L1	EH domain-binding protein 1-like protein 1
ALLST	Q8N3D4	EH1L1	EH domain-binding protein 1-like protein 1
VIDTP	Q9NZN3	EHD3	EH domain-containing protein 3
KSALL	Q92616	GCN1	eIF-2-alpha kinase activator GCN1
IKEEV	Q92616	GCN1	eIF-2-alpha kinase activator GCN1
ELQLL	Q9P2K8	E2AK4	eIF-2-alpha kinase GCN2
LSLIA	Q14576	ELAV3	ELAV-like protein 3
KKRKR	Q9BY07	S4A5	Electrogenic sodium bicarbonate cotransporter 4
PSEIN	Q2Y0W8	S4A8	Electroneutral sodium bicarbonate exchanger 1
RLLEI	Q8IUD2	RB6I2	ELKS/Rab6-interacting/CAST family member 1
GKSTT	P68104	EF1A1	Elongation factor 1-alpha 1
GKSTT	Q05639	EF1A2	Elongation factor 1-alpha 2
TSKVL	Q7Z2Z2	EFL1	Elongation factor-like GTPase 1
GAIVS	Q9HB03	ELOV3	Elongation of very long chain fatty acids protein 3

LIAVG	Q9HB03	ELOV3	Elongation of very long chain fatty acids protein 3
ALRTG	Q9H5J4	ELOV6	Elongation of very long chain fatty acids protein 6
SVSFF	Q0PNE2	ELP6	Elongator complex protein 6
KAVVS	Q8IYF1	ELOA2	Elongin-A2
AYVVQ	P10266	POK10	Endogenous retrovirus group K member 10 Pol protein
AYVVQ	Q9UQG0	POK11	Endogenous retrovirus group K member 11 Pol protein
AYVVQ	P63132	PO113	Endogenous retrovirus group K member 113 Pol protein
STNKA	P61578	REC16	Endogenous retrovirus group K member 16 Rec protein
AYVVQ	Q9QC07	POK18	Endogenous retrovirus group K member 18 Pol protein
AYVVQ	Q9WJR5	POK19	Endogenous retrovirus group K member 19 Pol protein
SLTLP	P61566	ENK24	Endogenous retrovirus group K member 24 Env polyprotein
AYVVQ	P63136	POK25	Endogenous retrovirus group K member 25 Pol protein
AYVVQ	Q9BXR3	POK6	Endogenous retrovirus group K member 6 Pol protein
AYVVQ	P63135	POK7	Endogenous retrovirus group K member 7 Pol protein
SLTLP	Q902F8	ENK8	Endogenous retrovirus group K member 8 Env polyprotein precursor
AYVVQ	P63133	POK8	Endogenous retrovirus group K member 8 Pol protein
SLTLP	Q9UKH3	ENK9	Endogenous retrovirus group K member 9 Env polyprotein precursor
LLSLI	P61550	ENVT1	Endogenous retrovirus group S71 member 1 Env polyprotein precursor
AVGLL	B6SEH8	ERVV1	Endogenous retrovirus group V member 1 Env polyprotein precursor

AVGLL	B6SEH9	ERVV2	Endogenous retrovirus group V member 2 Env polyprotein precursor
ITNDQ	P11021	BIP	Endoplasmic reticulum chaperone BiP precursor
LLSLI	Q14494	NF2L1	Endoplasmic reticulum membrane sensor NFE2L1
ILLSL	Q14494	NF2L1	Endoplasmic reticulum membrane sensor NFE2L1
TSKVL	Q5T6L9	EMARD	Endoplasmic reticulum membrane-associated RNA degradation protein
LGFLL	P30040	ERP29	Endoplasmic reticulum resident protein 29 precursor
VITSL	Q9HD20	AT131	Endoplasmic reticulum transmembrane helix translocase
KAVVS	P14625	ENPL	Endoplasmin precursor
LSGIN	Q6UXG2	ELAP1	Endosome/lysosome-associated apoptosis and autophagy regulator 1 precursor
NVTLS	Q96AP7	ESAM	Endothelial cell-selective adhesion molecule precursor
LGFLL	Q19T08	ECSCR	Endothelial cell-specific chemotaxis regulator precursor
LHNVN	P23769	GATA2	Endothelial transcription factor GATA-2
SALLS	P42892	ECE1	Endothelin-converting enzyme 1
SIIKE	Q96JJ3	ELMO2	Engulfment and cell motility protein 2
LLSLI	Q6P2E9	EDC4	Enhancer of mRNA-decapping protein 4
NGVSV	Q14244	MAP7	Ensconsin
LHLEG	P21709	EPHA1	Ephrin type-A receptor 1 precursor
NDMPI	P29317	EPHA2	Ephrin type-A receptor 2 precursor
NIKEN	P54756	EPHA5	Ephrin type-A receptor 5 precursor
EVLAY	P29322	EPHA8	Ephrin type-A receptor 8 precursor

SLIAV	P29323	EPHB2	Ephrin type-B receptor 2 precursor
LRTGW	P54760	EPHB4	Ephrin type-B receptor 4 precursor
LGAIV	Q8TE68	ES8L1	Epidermal growth factor receptor kinase substrate 8-like protein 1
AVVSL	P00533	EGFR	Epidermal growth factor receptor precursor
ITSLG	P00533	EGFR	Epidermal growth factor receptor precursor
LSKDQ	P42566	EPS15	Epidermal growth factor receptor substrate 15
LSKDQ	Q9UBC2	EP15R	Epidermal growth factor receptor substrate 15-like 1
LSLIA	Q8WX39	LCN9	Epididymal-specific lipocalin-9 precursor
VGLLL	Q6UW88	EPGN	Epigen precursor
SELLS	P58107	EPIPL	Epiplakin
TVSVG	P58107	EPIPL	Epiplakin
FMNYT	Q6NXG1	ESRP1	Epithelial splicing regulatory protein 1
VNKQE	Q9NQ60	EQTN	Equatorin precursor
AIASG	Q99808	S29A1	Equilibrative nucleoside transporter 1
IVILL	P33947	ERD22	ER lumen protein-retaining receptor 2
VGLLL	Q5UCC4	EMC10	ER membrane protein complex subunit 10 precursor
LLILK	Q9BV81	EMC6	ER membrane protein complex subunit 6
LILKA	Q9BV81	EMC6	ER membrane protein complex subunit 6
RLLEI	O15083	ERC2	ERC protein 2
AGSVS	O94905	ERLN2	Erlin-2
RARRE	Q4G0M1	ERFE	Erythroferrone precursor
SCSIS	P16581	LYAM2	E-selectin precursor
NSELL	B1AK53	ESPN	Espin

SNKGM	P03372	ESR1	Estrogen receptor
SNKGM	Q92731	ESR2	Estrogen receptor beta
SVNAG	Q15723	ELF2	ETS-related transcription factor Elf-2
AGKST	P15170	ERF3A	Eukaryotic peptide chain release factor GTP-binding subunit ERF3A
AGKST	Q8IYD1	ERF3B	Eukaryotic peptide chain release factor GTP-binding subunit ERF3B
VVQLP	Q9BY44	EIF2A	Eukaryotic translation initiation factor 2A
SDEFD	Q9NZJ5	E2AK3	Eukaryotic translation initiation factor 2-alpha kinase 3 precursor
LINDM	Q14152	EIF3A	Eukaryotic translation initiation factor 3 subunit A
LQLLM	O15372	EIF3H	Eukaryotic translation initiation factor 3 subunit H
ELLSL	O15372	EIF3H	Eukaryotic translation initiation factor 3 subunit H
VTLSK	Q04637	IF4G1	Eukaryotic translation initiation factor 4 gamma 1
STPVT	O43432	IF4G3	Eukaryotic translation initiation factor 4 gamma 3
GVTTP	O60516	4EBP3	Eukaryotic translation initiation factor 4E-binding protein 3
LLGVG	Q96CN4	EVI5L	EVI5-like protein
NRLLE	Q96CN4	EVI5L	EVI5-like protein
VGLLL	Q96CN4	EVI5L	EVI5-like protein
PRFMN	Q9BQ95	ECSIT	Evolutionarily conserved signaling intermediate in Toll pathway, mitochondrial precursor
SISQV	Q9BQ95	ECSIT	Evolutionarily conserved signaling intermediate in Toll pathway, mitochondrial precursor
IIIVI	P43003	EAA1	Excitatory amino acid transporter 1

IIVII	P43003	EAA1	Excitatory amino acid transporter 1
ILTAV	P43004	EAA2	Excitatory amino acid transporter 2
AVVSL	P43004	EAA2	Excitatory amino acid transporter 2
LTLPS	P48664	EAA4	Excitatory amino acid transporter 4
YLSAL	Q86VI1	EX3L1	Exocyst complex component 3-like protein
VLHLE	Q96A65	EXOC4	Exocyst complex component 4
YDPLV	Q96A65	EXOC4	Exocyst complex component 4
VLDLK	Q9Y2D4	EXC6B	Exocyst complex component 6B
LDLKN	Q9Y2D4	EXC6B	Exocyst complex component 6B
EFSVN	Q8NEV8	EXPH5	Exophilin-5
LILKA	Q06265	EXOS9	Exosome complex component RRP45
STPPT	Q9C0E2	XPO4	Exportin-4
NKGMD	O43592	XPOT	Exportin-T
LSLIA	O43592	XPOT	Exportin-T
VLHLE	A0FGR8	ESYT2	Extended synaptotagmin-2
AGSVS	Q86XX4	FRAS1	Extracellular matrix organizing protein FRAS1 precursor
LPIVN	O94769	ECM2	Extracellular matrix protein 2 precursor
SVSFF	Q8IWU5	SULF2	Extracellular sulfatase Sulf-2 precursor
KVLHL	Q504Y2	PKDCC	Extracellular tyrosine-protein kinase PKDCC precursor
SVITS	O95677	EYA4	Eyes absent homolog 4
SALLS	Q86X51	EZHIP	EZH inhibitory protein
VVSLS	Q0VG06	FP100	Fanconi anemia core complex-associated protein 100
LSALR	Q00597	FANCC	Fanconi anemia group C protein

ELQLL	Q9HB96	FANCE	Fanconi anemia group E protein
SSSVI	Q8IYD8	FANCM	Fanconi anemia group M protein
NGCDY	Q8IYD8	FANCM	Fanconi anemia group M protein
SDELL	Q8IYD8	FANCM	Fanconi anemia group M protein
ITEEF	Q96I24	FUBP3	Far upstream element-binding protein 3
KSALL	Q8TES7	FBF1	Fas-binding factor 1
RRELP	Q96IV6	FXDC2	Fatty acid hydroxylase domain-containing protein 2
GKSTT	P15090	FABP4	Fatty acid-binding protein, adipocyte
ASGVA	O00519	FAAH1	Fatty-acid amide hydrolase 1
LSGIN	Q0JRZ9	FCHO2	F-BAR domain only protein 2
AVSKV	Q8NFZ0	FBH1	F-box DNA helicase 1
SKKRK	Q8TCJ0	FBX25	F-box only protein 25
SDELL	Q8N4B4	FBX39	F-box only protein 39
SISQV	Q4G163	FBX43	F-box only protein 43
SDELL	Q9UKT7	FBXL3	F-box/LRR-repeat protein 3
DVSSS	Q9UJT9	FBXL7	F-box/LRR-repeat protein 7
TCSAV	Q96CD0	FBXL8	F-box/LRR-repeat protein 8
EEVLA	Q96CD0	FBXL8	F-box/LRR-repeat protein 8
NKAVV	Q9BZK7	TBL1R	F-box-like/WD repeat-containing protein TBL1XR1
LLLYC	Q96RD9	FCRL5	Fc receptor-like protein 5 precursor
LLEIT	A9Z1Z3	FR1L4	Fer-1-like protein 4
SALLS	Q68DX3	FRPD2	FERM and PDZ domain-containing protein 2
LAYVV	Q5JV73	FRPD3	FERM and PDZ domain-containing protein 3

AYVVQ	Q5JV73	FRPD3	FERM and PDZ domain-containing protein 3
LTSKV	Q6ZUT3	FRMD7	FERM domain-containing protein 7
NVTLS	Q9Y4F1	FARP1	FERM, ARHGEF and pleckstrin domain-containing protein 1
QLPLY	Q9Y4F1	FARP1	FERM, ARHGEF and pleckstrin domain-containing protein 1
LPLYG	Q9Y4F1	FARP1	FERM, ARHGEF and pleckstrin domain-containing protein 1
NPKYD	Q9BQL6	FERM1	Fermitin family homolog 1
NPKYD	Q96AC1	FERM2	Fermitin family homolog 2
SVITS	Q9BRP7	FDXA1	Ferredoxin-fold anticodon-binding domain-containing protein 1
SKGYL	Q6ZNA5	FRRS1	Ferric-chelate reductase 1
KGYLS	Q6ZNA5	FRRS1	Ferric-chelate reductase 1
VKLIK	P02792	FRIL	Ferritin light chain
SALLS	Q9UGM5	FETUB	Fetuin-B precursor
SELLS	Q8TBJ5	FEZF2	Fez family zinc finger protein 2
GVSVL	Q9C0D6	FHDC1	FH2 domain-containing protein 1
RLLEI	P35555	FBN1	Fibrillin-1 precursor
QLSGI	P35555	FBN1	Fibrillin-1 precursor
TNTKE	P02671	FIBA	Fibrinogen alpha chain precursor
TTNIM	P02671	FIBA	Fibrinogen alpha chain precursor
AIASG	Q92915	FGF14	Fibroblast growth factor 14
IAVGL	P31371	FGF9	Fibroblast growth factor 9 precursor
FSNGC	O43559	FRS3	Fibroblast growth factor receptor substrate 3
SISNI	Q86WI1	PKHL1	Fibrocystin-L precursor
NAGSV	Q86WI1	PKHL1	Fibrocystin-L precursor

TVSVG	Q86WI1	PKHL1	Fibrocystin-L precursor
PIVNK	P02751	FINC	Fibronectin precursor
STPVT	P02751	FINC	Fibronectin precursor
GFLLG	Q4ZHG4	FNDC1	Fibronectin type III domain-containing protein 1 precursor
LDLKN	Q4ZHG4	FNDC1	Fibronectin type III domain-containing protein 1 precursor
LLEIT	Q53EP0	FND3B	Fibronectin type III domain-containing protein 3B
NSLTL	Q9Y2H6	FND3A	Fibronectin type-III domain-containing protein 3A
SVSFF	Q9HCM7	FBSL	Fibrosin-1-like protein
VSFFP	Q9HCM7	FBSL	Fibrosin-1-like protein
IKQEL	Q5CZC0	FSIP2	Fibrous sheath-interacting protein 2
KQELD	Q5CZC0	FSIP2	Fibrous sheath-interacting protein 2
QELDK	Q5CZC0	FSIP2	Fibrous sheath-interacting protein 2
KIKSA	Q5CZC0	FSIP2	Fibrous sheath-interacting protein 2
TSKVL	Q5CZC0	FSIP2	Fibrous sheath-interacting protein 2
IMSII	Q5CZC0	FSIP2	Fibrous sheath-interacting protein 2
EEVLA	Q5CZC0	FSIP2	Fibrous sheath-interacting protein 2
SDELL	Q5CZC0	FSIP2	Fibrous sheath-interacting protein 2
DVSSS	O00602	FCN1	Ficolin-1 precursor
IKEEV	Q5HY92	FIGN	Fidgetin
KEEVL	Q5HY92	FIGN	Fidgetin
SQVNE	Q5HY92	FIGN	Fidgetin
TLSKK	P20930	FILA	Filaggrin
SKTDV	Q4L180	FIL1L	Filamin A-interacting protein 1-like

SSSVI	Q4L180	FIL1L	Filamin A-interacting protein 1-like
SSVIT	Q4L180	FIL1L	Filamin A-interacting protein 1-like
GKSTT	Q4L180	FIL1L	Filamin A-interacting protein 1-like
AYVVQ	P21333	FLNA	Filamin-A
FIRKS	Q7Z7B0	FLIP1	Filamin-A-interacting protein 1
GVAVS	Q12934	BFSP1	Filensin
AVSKV	Q5T1M5	FKB15	FK506-binding protein 15
SISQV	O75955	FLOT1	Flotillin-1
ISQVN	O75955	FLOT1	Flotillin-1
VGLLL	P49771	FLT3L	Fms-related tyrosine kinase 3 ligand precursor
EVLAY	Q5VW36	FOCAD	Focadhesin
SNRVF	P23945	FSHR	Follicle-stimulating hormone receptor precursor
LSALR	Q12950	FOXD4	Forkhead box protein D4
LSALR	Q6VB84	FX4L3	Forkhead box protein D4-like 3
LSALR	Q8WXT5	FX4L4	Forkhead box protein D4-like 4
LSALR	Q5VV16	FX4L5	Forkhead box protein D4-like 5
LSALR	Q3SYB3	FX4L6	Forkhead box protein D4-like 6
AKKTN	Q12946	FOXF1	Forkhead box protein F1
DVSSS	Q12951	FOXI1	Forkhead box protein I1
KRKRR	Q6ZQN5	FOXI2	Forkhead box protein I2
KRKRR	A8MTJ6	FOXI3	Forkhead box protein I3
VSSSV	A8MTJ6	FOXI3	Forkhead box protein I3
TLSKD	Q9P0K8	FOXJ2	Forkhead box protein J2

DKQLL	Q9UPW0	FOXJ3	Forkhead box protein J3
TPVTL	P85037	FOXK1	Forkhead box protein K1
VTLSK	Q9H334	FOXP1	Forkhead box protein P1
LDLKN	B1AJZ9	FHAD1	Forkhead-associated domain-containing protein 1
SVITI	E5RQL4	FONG	Formiminotransferase N-terminal subdomain-containing protein precursor
GKSLY	Q9NZ56	FMN2	Formin-2
AGSVS	O95466	FMNL1	Formin-like protein 1
NAITT	P15408	FOSL2	Fos-related antigen 2
AVGLL	P78423	X3CL1	Fractalkine precursor
TNSEL	P51114	FXR1	Fragile X mental retardation syndrome-related protein 1
TNSEL	P51116	FXR2	Fragile X mental retardation syndrome-related protein 2
MSNNV	Q5H8C1	FREM1	FRAS1-related extracellular matrix protein 1 precursor
AVGLL	Q5H8C1	FREM1	FRAS1-related extracellular matrix protein 1 precursor
VIDTP	Q5SZK8	FREM2	FRAS1-related extracellular matrix protein 2 precursor
SVITS	Q5SZK8	FREM2	FRAS1-related extracellular matrix protein 2 precursor
NSLTL	P0C091	FREM3	FRAS1-related extracellular matrix protein 3 precursor
PLYGV	O15552	FFAR2	Free fatty acid receptor 2
LYGVI	O15552	FFAR2	Free fatty acid receptor 2
IVIIV	O15552	FFAR2	Free fatty acid receptor 2
SVITS	Q9ULW2	FZD10	Frizzled-10 precursor
GFLLG	O60353	FZD6	Frizzled-6 precursor
AGSVS	O60353	FZD6	Frizzled-6 precursor

KSDEL	O60353	FZD6	Frizzled-6 precursor
SVITS	O14772	FPGT	Fucose-1-phosphate guanylyltransferase
VITSL	O14772	FPGT	Fucose-1-phosphate guanylyltransferase
GFLLG	Q8IVP5	FUND1	FUN14 domain-containing protein 1
GFLLG	Q9BWH2	FUND2	FUN14 domain-containing protein 2
AVTEL	Q9BQS8	FYCO1	FYVE and coiled-coil domain-containing protein 1
EFQQK	Q9BQS8	FYCO1	FYVE and coiled-coil domain-containing protein 1
TSLGA	P98174	FGD1	FYVE, RhoGEF and PH domain-containing protein 1
PVTLS	Q7Z6J4	FGD2	FYVE, RhoGEF and PH domain-containing protein 2
DMPIT	Q5JSP0	FGD3	FYVE, RhoGEF and PH domain-containing protein 3
KSTTN	Q5VSY0	GKAP1	G kinase-anchoring protein 1
NAGKS	Q9NW75	GPTC2	G patch domain-containing protein 2
SKKRK	Q9UKJ3	GPTC8	G patch domain-containing protein 8
KKRKR	Q9UKJ3	GPTC8	G patch domain-containing protein 8
AVVSL	Q92806	KCNJ9	G protein-activated inward rectifier potassium channel 3
GKSTT	Q7L622	G2E3	G2/M phase-specific E3 ubiquitin-protein ligase
LSNGV	Q96C23	GALM	Galactose mutarotase
RARRE	Q96RP7	G3ST4	Galactose-3-O-sulfotransferase 4
LLGVG	P47211	GALR1	Galanin receptor type 1
VGNTL	O43603	GALR2	Galanin receptor type 2
AVVSL	O60755	GALR3	Galanin receptor type 3
ITTIL	O00182	LEG9	Galectin-9
YVKGE	Q9UEY8	ADDG	Gamma-adducin

VSSSV	Q16445	GBRA6	Gamma-aminobutyric acid receptor subunit alpha-6 precursor
STPVT	Q16445	GBRA6	Gamma-aminobutyric acid receptor subunit alpha-6 precursor
LYYVN	P47870	GBRB2	Gamma-aminobutyric acid receptor subunit beta-2 precursor
LYYVN	P28472	GBRB3	Gamma-aminobutyric acid receptor subunit beta-3 precursor
VITIE	Q9HBI0	PARVG	Gamma-parvin
ELLSL	Q9BSJ2	GCP2	Gamma-tubulin complex component 2
ELQLL	Q96RT7	GCP6	Gamma-tubulin complex component 6
STPPT	Q96RT7	GCP6	Gamma-tubulin complex component 6
DASIS	Q96RT7	GCP6	Gamma-tubulin complex component 6
NVTLS	P17900	SAP3	Ganglioside GM2 activator precursor
QKKLM	Q96MZ0	GD1L1	Ganglioside-induced differentiation-associated protein 1-like 1
LSKKR	Q9NTQ9	CXB4	Gap junction beta-4 protein
LLSLI	Q9NTQ9	CXB4	Gap junction beta-4 protein
SALLS	Q8N144	CXD3	Gap junction delta-3 protein
AVGLL	Q8N144	CXD3	Gap junction delta-3 protein
RSTPV	Q8NFK1	CXG3	Gap junction gamma-3 protein
NAVTE	Q99501	GA2L1	GAS2-like protein 1
EGKSL	Q8TAX9	GSDMB	Gasdermin-B
DPLVF	O60443	GSDME	Gasdermin-E
SLAFI	P07098	LIPG	Gastric triacylglycerol lipase precursor
VGLLL	P07098	LIPG	Gastric triacylglycerol lipase precursor
DVSSS	O75140	DEPD5	GATOR complex protein DEPDC5
TSVIT	O00451	GFRA2	GDNF family receptor alpha-2 precursor

KVKLI	Q2TAA5	ALG11	GDP-Man:Man(3)GlcNAc(2)-PP-Dol alpha-1,2-mannosyltransferase
DKQLL	Q8TEQ6	GEMI5	Gem-associated protein 5
TKCTA	Q9P107	GMIP	GEM-interacting protein
FLGFL	P18074	ERCC2	General transcription and DNA repair factor IIH helicase subunit XPD
AIASG	Q92759	TF2H4	General transcription factor IIH subunit 4
LSALR	Q14687	GSE1	Genetic suppressor element 1
TVIEF	Q6UXU4	GSG1L	Germ cell-specific gene 1-like protein
TVSVG	O60318	GANP	Germinal-center associated nuclear protein
LGFLL	Q6UWM5	GPRL1	GLIPR1-like protein 1 precursor
ALLST	O95838	GLP2R	Glucagon-like peptide 2 receptor
AGKST	Q9Y692	GMEB1	Glucocorticoid modulatory element-binding protein 1
AGKST	Q9UKD1	GMEB2	Glucocorticoid modulatory element-binding protein 2
LLGVG	Q8TDV5	GP119	Glucose-dependent insulinotropic receptor
VTELQ	P14314	GLU2B	Glucosidase 2 subunit beta precursor
KYKNA	A0PJZ3	GXLT2	Glucoside xylosyltransferase 2
GVSVL	A0PJZ3	GXLT2	Glucoside xylosyltransferase 2
EGKSL	Q04609	FOLH1	Glutamate carboxypeptidase 2
GKSLY	Q04609	FOLH1	Glutamate carboxypeptidase 2
LLSLI	P42262	GRIA2	Glutamate receptor 2 precursor
ELSNI	P39086	GRIK1	Glutamate receptor ionotropic, kainate 1 precursor
PRFMN	P39086	GRIK1	Glutamate receptor ionotropic, kainate 1 precursor

VITSL	Q14957	NMDE3	Glutamate receptor ionotropic, NMDA 2C precursor
RRELP	Q8TCU5	NMD3A	Glutamate receptor ionotropic, NMDA 3A precursor
SALLS	O60391	NMD3B	Glutamate receptor ionotropic, NMDA 3B precursor
KIKSA	Q9Y3R0	GRIP1	Glutamate receptor-interacting protein 1
SVITI	Q9C0E4	GRIP2	Glutamate receptor-interacting protein 2
RGIIK	P48507	GSH0	Glutamate--cysteine ligase regulatory subunit
IKENK	Q5RHP9	ERIC3	Glutamate-rich protein 3
GVAVS	Q5RHP9	ERIC3	Glutamate-rich protein 3
VVSLS	Q2KHR3	QSER1	Glutamine and serine-rich protein 1
LDLKN	Q2KHR3	QSER1	Glutamine and serine-rich protein 1
EEVLA	Q6IA69	NADE	Glutamine-dependent NAD(+) synthetase
GYLSA	Q9H0J4	QRIC2	Glutamine-rich protein 2
TELQL	Q07075	AMPE	Glutamyl aminopeptidase
YTLNN	Q07075	AMPE	Glutamyl aminopeptidase
LYVKG	O76003	GLRX3	Glutaredoxin-3
YVKGE	O76003	GLRX3	Glutaredoxin-3
GIIKT	P32189	GLPK	Glycerol kinase
GIIKT	Q14409	GLPK3	Glycerol kinase 3
SALLS	Q9HCL2	GPAT1	Glycerol-3-phosphate acyltransferase 1, mitochondrial precursor
KEEVL	Q9HCL2	GPAT1	Glycerol-3-phosphate acyltransferase 1, mitochondrial precursor
EEVLA	Q9HCL2	GPAT1	Glycerol-3-phosphate acyltransferase 1, mitochondrial precursor
LKANA	P21695	GPDA	Glycerol-3-phosphate dehydrogenase [NAD(+)], cytoplasmic
GSAIA	P21695	GPDA	Glycerol-3-phosphate dehydrogenase [NAD(+)], cytoplasmic

KNAVT	Q6W3E5	GDPD4	Glycerophosphodiester phosphodiesterase domain-containing protein 4
LGFLL	Q6W3E5	GDPD4	Glycerophosphodiester phosphodiesterase domain-containing protein 4
EEVLA	P41250	GARS	Glycine--tRNA ligase precursor
DYVSN	P35573	GDE	Glycogen debranching enzyme
GMDTV	O75063	XYLK	Glycosaminoglycan xylosylkinase
LLGVG	Q8WWB7	GLMP	Glycosylated lysosomal membrane protein precursor
LGFLL	O43292	GPAA1	Glycosylphosphatidylinositol anchor attachment 1 protein
AVGLL	O43292	GPAA1	Glycosylphosphatidylinositol anchor attachment 1 protein
VGLLL	O43292	GPAA1	Glycosylphosphatidylinositol anchor attachment 1 protein
TSKVL	P49915	GUAA	GMP synthase [glutamine-hydrolyzing]
SKDQL	Q08378	GOGA3	Golgin subfamily A member 3
SIIKE	Q13439	GOGA4	Golgin subfamily A member 4
EFDAS	Q13439	GOGA4	Golgin subfamily A member 4
AKKTN	Q9NYA3	GOG6A	Golgin subfamily A member 6A
AKKTN	A6NDN3	GOG6B	Golgin subfamily A member 6B
AKKTN	A6NDK9	GOG6C	Golgin subfamily A member 6C
AKKTN	P0CG33	GOG6D	Golgin subfamily A member 6D
TLSKK	Q14789	GOGB1	Golgin subfamily B member 1
VSVLT	O95427	PIGN	GPI ethanolamine phosphate transferase 1
ILLSL	O95427	PIGN	GPI ethanolamine phosphate transferase 1
VSVLT	Q5H8A4	PIGG	GPI ethanolamine phosphate transferase 2

VGLLL	Q8TEQ8	PIGO	GPI ethanolamine phosphate transferase 3
ILLSL	Q96S52	PIGS	GPI transamidase component PIG-S
ASGVA	Q8IZ08	GP135	G-protein coupled receptor 135
LGFLL	O15218	GP182	G-protein coupled receptor 182
IVILL	Q99680	GPR22	G-protein coupled receptor 22
LSLIA	P46093	GPR4	G-protein coupled receptor 4
QSLAF	Q96P69	GPR78	G-protein coupled receptor 78
IIVIL	Q5T6X5	GPC6A	G-protein coupled receptor family C group 6 member A precursor
NNRAR	Q5JY77	GASP1	G-protein coupled receptor-associated sorting protein 1
QLSGI	Q5JY77	GASP1	G-protein coupled receptor-associated sorting protein 1
ISNIE	Q9NZI5	GRHL1	Grainyhead-like protein 1 homolog
KRRFL	O15063	GRRE1	Granule associated Rac and RHOG effector protein 1
FSVNA	Q6Y7W6	GGYF2	GRB10-interacting GYF protein 2
SISQV	Q8WWW8	GAB3	GRB2-associated-binding protein 3
SDELL	Q9C091	GRB1L	GREB1-like protein
GLLLY	Q9C091	GRB1L	GREB1-like protein
KDQLS	Q8IWJ2	GCC2	GRIP and coiled-coil domain-containing protein 2
NQSLA	Q4V328	GRAP1	GRIP1-associated protein 1
LLILK	Q14449	GRB14	Growth factor receptor-bound protein 14
LGFLL	O95390	GDF11	Growth/differentiation factor 11 precursor
KQLLP	O95390	GDF11	Growth/differentiation factor 11 precursor
STPPT	P43026	GDF5	Growth/differentiation factor 5 precursor
NAGKS	O75616	ERAL1	GTPase Era, mitochondrial precursor

AGKST	O75616	ERAL1	GTPase Era, mitochondrial precursor
DYVSN	Q8WWP7	GIMA1	GTPase IMAP family member 1
VIDTP	Q6P9H5	GIMA6	GTPase IMAP family member 6
VIDTP	Q8ND71	GIMA8	GTPase IMAP family member 8
AGKST	O00178	GTPB1	GTP-binding protein 1
NAGKS	A4D1E9	GTPBA	GTP-binding protein 10
SGVAV	O15068	MCF2L	Guanine nucleotide exchange factor DBS
ARREL	P50148	GNAQ	Guanine nucleotide-binding protein G(q) subunit alpha
IDKQL	Q5JWF2	GNAS1	Guanine nucleotide-binding protein G(s) subunit alpha isoforms XLas
ARREL	P29992	GNA11	Guanine nucleotide-binding protein subunit alpha-11
ARREL	O95837	GNA14	Guanine nucleotide-binding protein subunit alpha-14
VSFFP	Q9HAV0	GBB4	Guanine nucleotide-binding protein subunit beta-4
KEEVL	Q9NVN8	GNL3L	Guanine nucleotide-binding protein-like 3-like protein
SDELL	Q9NVN8	GNL3L	Guanine nucleotide-binding protein-like 3-like protein
GIIKT	Q02153	GCYB1	Guanylate cyclase soluble subunit beta-1
IELSN	O75343	GCYB2	Guanylate cyclase soluble subunit beta-2
AGKST	Q16774	KGUA	Guanylate kinase
VSFFP	P32455	GBP1	Guanylate-binding protein 1 precursor
VSFFP	P32456	GBP2	Guanylate-binding protein 2 precursor
VSFFP	Q9H0R5	GBP3	Guanylate-binding protein 3
LLGVG	Q96PP9	GBP4	Guanylate-binding protein 4
LLHNV	Q96PP8	GBP5	Guanylate-binding protein 5 precursor

VSFFP	Q6ZN66	GBP6	Guanylate-binding protein 6
VSFFP	Q8N8V2	GBP7	Guanylate-binding protein 7
TLPSE	Q9NXP7	GIN1	Gypsy retrotransposon integrase-like protein 1
LLGVG	P51795	CLCN5	H(+)/Cl(-) exchange transporter 5
STPVT	P51798	CLCN7	H(+)/Cl(-) exchange transporter 7
LLGVG	O60832	DKC1	H/ACA ribonucleoprotein complex subunit DKC1
QKKLM	O60832	DKC1	H/ACA ribonucleoprotein complex subunit DKC1
YTLNN	P00738	HPT	Haptoglobin precursor
LSKKR	Q7Z4H7	HAUS6	HAUS augmin-like complex subunit 6
AGKST	Q9Y450	HBS1L	HBS1-like protein
EEFYQ	Q53T59	H1BP3	HCLS1-binding protein 3
SALLS	Q7Z4Q2	HEAT3	HEAT repeat-containing protein 3
ELLIL	Q86XA9	HTR5A	HEAT repeat-containing protein 5A
IASGV	Q86XA9	HTR5A	HEAT repeat-containing protein 5A
LTLPS	Q86XA9	HTR5A	HEAT repeat-containing protein 5A
TLPSE	Q86XA9	HTR5A	HEAT repeat-containing protein 5A
VAVSK	Q9P2D3	HTR5B	HEAT repeat-containing protein 5B
LGAIV	Q6AI08	HEAT6	HEAT repeat-containing protein 6
GAIVS	Q6AI08	HEAT6	HEAT repeat-containing protein 6
KKLMS	P34932	HSP74	Heat shock 70 kDa protein 4
KKLMS	O95757	HS74L	Heat shock 70 kDa protein 4L
VQSNR	Q00613	HSF1	Heat shock factor protein 1
ALLST	Q4G112	HSF5	Heat shock factor protein 5

REFSV	Q92598	HS105	Heat shock protein 105 kDa
KKLMS	Q92598	HS105	Heat shock protein 105 kDa
RARRE	Q6ZRS2	SRCAP	Helicase SRCAP
LEGEV	Q6ZRS2	SRCAP	Helicase SRCAP
GFLLG	Q9BYK8	HELZ2	Helicase with zinc finger domain 2
GVAVS	Q9BYK8	HELZ2	Helicase with zinc finger domain 2
FPQAE	Q9BYK8	HELZ2	Helicase with zinc finger domain 2
TSVIT	P28906	CD34	Hematopoietic progenitor cell antigen CD34 precursor
SVITS	P28906	CD34	Hematopoietic progenitor cell antigen CD34 precursor
VSLSN	Q96RW7	HMCN1	Hemicentin-1 precursor
IDTPC	Q96RW7	HMCN1	Hemicentin-1 precursor
CTASN	Q8NDA2	HMCN2	Hemicentin-2 precursor
VLTSK	P69905	HBA	Hemoglobin subunit alpha
TTIII	Q6ZVN8	RGMC	Hemojuvelin precursor
TLSKK	Q9Y278	HS3S2	Heparan sulfate glucosamine 3-O-sulfotransferase 2
VLHLE	Q9Y278	HS3S2	Heparan sulfate glucosamine 3-O-sulfotransferase 2
TLSKK	Q9Y661	HS3S4	Heparan sulfate glucosamine 3-O-sulfotransferase 4
VIIVI	Q68CP4	HGNAT	Heparan-alpha-glucosaminide N-acetyltransferase
TSPLC	P11150	LIPC	Hepatic triacylglycerol lipase precursor
KSALL	Q14CZ8	HECAM	Hepatocyte cell adhesion molecule precursor
SALLS	Q14CZ8	HECAM	Hepatocyte cell adhesion molecule precursor
LSALR	O14964	HGS	Hepatocyte growth factor-regulated tyrosine kinase substrate
SALLS	P35680	HNF1B	Hepatocyte nuclear factor 1-beta

QLLPI	Q969F9	HPS3	Hermansky-Pudlak syndrome 3 protein
ELLSL	Q969F9	HPS3	Hermansky-Pudlak syndrome 3 protein
LSALR	Q9UPZ3	HPS5	Hermansky-Pudlak syndrome 5 protein
AIASG	Q9UPZ3	HPS5	Hermansky-Pudlak syndrome 5 protein
LHTSP	Q9UPZ3	HPS5	Hermansky-Pudlak syndrome 5 protein
IKQEL	O60812	HNRC1	Heterogeneous nuclear ribonucleoprotein C-like 1
IKQEL	B2RXH8	HNRC2	Heterogeneous nuclear ribonucleoprotein C-like 2
IKQEL	B7ZW38	HNRC3	Heterogeneous nuclear ribonucleoprotein C-like 3
IKQEL	P0DMR1	HNRC4	Heterogeneous nuclear ribonucleoprotein C-like 4
IKENK	P52789	HXK2	Hexokinase-2
KSALL	Q96JK4	HIPL1	HHIP-like protein 1 precursor
LDLKN	Q6UWX4	HIPL2	HHIP-like protein 2 precursor
LLSLI	Q13946	PDE7A	High affinity cAMP-specific 3',5'-cyclic phosphodiesterase 7A
ILLSL	Q13946	PDE7A	High affinity cAMP-specific 3',5'-cyclic phosphodiesterase 7A
LSLIA	Q13946	PDE7A	High affinity cAMP-specific 3',5'-cyclic phosphodiesterase 7A
AVGLL	Q13946	PDE7A	High affinity cAMP-specific 3',5'-cyclic phosphodiesterase 7A
LGVGS	P30825	SL7A1	High affinity cationic amino acid transporter 1
SLIAV	P30825	SL7A1	High affinity cationic amino acid transporter 1
IINFY	P35367	HRH1	Histamine H1 receptor
SIIKE	P42357	HUTH	Histidine ammonia-lyase
ITSLG	P42357	HUTH	Histidine ammonia-lyase
KVLDL	O95568	MET18	Histidine protein methyltransferase 1 homolog
LGVGS	P12081	HARS1	Histidine--tRNA ligase, cytoplasmic

ELLSL	P12081	HARS1	Histidine--tRNA ligase, cytoplasmic
VIDTP	P12081	HARS1	Histidine--tRNA ligase, cytoplasmic
ELLSL	P49590	SYHM	Histidine--tRNA ligase, mitochondrial precursor
KGMDT	P49590	SYHM	Histidine--tRNA ligase, mitochondrial precursor
VSLSN	Q92794	KAT6A	Histone acetyltransferase KAT6A
VSNKG	Q92794	KAT6A	Histone acetyltransferase KAT6A
SAIAS	Q9UQL6	HDAC5	Histone deacetylase 5
SALLS	Q9UQL6	HDAC5	Histone deacetylase 5
AGSVS	Q9H0E3	SP130	Histone deacetylase complex subunit SAP130
LILKA	O14607	UTY	Histone demethylase UTY
LTLPS	O14607	UTY	Histone demethylase UTY
SKKRK	Q92522	H1X	Histone H1.10
KKRKR	Q96A08	H2B1A	Histone H2B type 1-A
KKRKR	P33778	H2B1B	Histone H2B type 1-B
KKRKR	P62807	H2B1C	Histone H2B type 1-C/E/F/G/I
KKRKR	P58876	H2B1D	Histone H2B type 1-D
KKRKR	Q93079	H2B1H	Histone H2B type 1-H
KKRKR	P06899	H2B1J	Histone H2B type 1-J
KKRKR	O60814	H2B1K	Histone H2B type 1-K
KKRKR	Q99880	H2B1L	Histone H2B type 1-L
KKRKR	Q99879	H2B1M	Histone H2B type 1-M
KKRKR	Q99877	H2B1N	Histone H2B type 1-N
KKRKR	P23527	H2B1O	Histone H2B type 1-O

KKRKR	Q16778	H2B2E	Histone H2B type 2-E
KKRKR	Q5QNW6	H2B2F	Histone H2B type 2-F
KKRKR	Q8N257	H2B3B	Histone H2B type 3-B
LSALR	Q86X55	CARM1	Histone-arginine methyltransferase CARM1
LDLKN	Q86X55	CARM1	Histone-arginine methyltransferase CARM1
EGEVN	Q09028	RBBP4	Histone-binding protein RBBP4
EGEVN	Q16576	RBBP7	Histone-binding protein RBBP7
VQIVR	Q03164	KMT2A	Histone-lysine N-methyltransferase 2A
PLYGV	Q03164	KMT2A	Histone-lysine N-methyltransferase 2A
VNKQE	Q03164	KMT2A	Histone-lysine N-methyltransferase 2A
SVLTS	Q9UMN6	KMT2B	Histone-lysine N-methyltransferase 2B
VTTPV	Q9UMN6	KMT2B	Histone-lysine N-methyltransferase 2B
LPSEI	Q9UMN6	KMT2B	Histone-lysine N-methyltransferase 2B
SKKRK	Q8NEZ4	KMT2C	Histone-lysine N-methyltransferase 2C
KKRKR	Q8NEZ4	KMT2C	Histone-lysine N-methyltransferase 2C
KAVVS	Q8NEZ4	KMT2C	Histone-lysine N-methyltransferase 2C
QLLPI	Q8NEZ4	KMT2C	Histone-lysine N-methyltransferase 2C
DVSSS	Q8NEZ4	KMT2C	Histone-lysine N-methyltransferase 2C
SKKRK	O14686	KMT2D	Histone-lysine N-methyltransferase 2D
KKRKR	O14686	KMT2D	Histone-lysine N-methyltransferase 2D
TSLGA	O14686	KMT2D	Histone-lysine N-methyltransferase 2D
KKLMS	Q9NR48	ASH1L	Histone-lysine N-methyltransferase ASH1L
EEVLA	Q9NR48	ASH1L	Histone-lysine N-methyltransferase ASH1L

SLTLP	Q96KQ7	EHMT2	Histone-lysine N-methyltransferase EHMT2
SAIAS	Q92800	EZH1	Histone-lysine N-methyltransferase EZH1
SKTDV	O96028	NSD2	Histone-lysine N-methyltransferase NSD2
YIDKQ	Q9BZ95	NSD3	Histone-lysine N-methyltransferase NSD3
VITIE	O15047	SET1A	Histone-lysine N-methyltransferase SETD1A
KRKRR	O15047	SET1A	Histone-lysine N-methyltransferase SETD1A
KRKRR	Q9BYW2	SETD2	Histone-lysine N-methyltransferase SETD2
TSKTD	Q9BYW2	SETD2	Histone-lysine N-methyltransferase SETD2
SKKRK	Q96T68	SETB2	Histone-lysine N-methyltransferase SETDB2
VSKVL	Q96L73	NSD1	Histone-lysine N-methyltransferase, H3 lysine-36 specific
ARSTP	Q8TEK3	DOT1L	Histone-lysine N-methyltransferase, H3 lysine-79 specific
AVTEL	P04440	DPB1	HLA class II histocompatibility antigen, DP beta 1 chain precursor
AVTEL	P05538	DQB2	HLA class II histocompatibility antigen, DQ beta 2 chain precursor
AVTEL	P79483	DRB3	HLA class II histocompatibility antigen, DR beta 3 chain precursor
AVTEL	P13762	DRB4	HLA class II histocompatibility antigen, DR beta 4 chain precursor
AVTEL	Q30154	DRB5	HLA class II histocompatibility antigen, DR beta 5 chain precursor
AVTEL	P01911	DRB1	HLA class II histocompatibility antigen, DRB1 beta chain precursor
SKDQL	Q12766	HMGX3	HMG domain-containing protein 3
GEVNK	P53701	CCHL	Holocytochrome c-type synthase
TELQL	Q9HBU1	BARX1	Homeobox protein BarH-like 1
TELQL	Q9UMQ3	BARX2	Homeobox protein BarH-like 2
AGSVS	P39880	CUX1	Homeobox protein cut-like 1
TELQL	P56915	GSC	Homeobox protein goosecoid

ELQLL	P56915	GSC	Homeobox protein goosecoid
QLPLY	P31267	HXA6	Homeobox protein Hox-A6
QLPLY	P17509	HXB6	Homeobox protein Hox-B6
ELSNI	Q9H9S0	NANOG	Homeobox protein NANOG
ELSNI	Q6NSW7	NANP8	Homeobox protein NANOGP8
KKRKR	O95096	NKX22	Homeobox protein Nkx-2.2
KRKRR	O95096	NKX22	Homeobox protein Nkx-2.2
SLTLP	O60393	NOBOX	Homeobox protein NOBOX
IASGV	P55347	PKNX1	Homeobox protein PKNOX1
ASGVA	P55347	PKNX1	Homeobox protein PKNOX1
KRKRR	Q9GZN2	TGIF2	Homeobox protein TGIF2
LSALR	Q86Z02	HIPK1	Homeodomain-interacting protein kinase 1
SVITI	Q86Z02	HIPK1	Homeodomain-interacting protein kinase 1
SVITI	Q9H2X6	HIPK2	Homeodomain-interacting protein kinase 2
SVITI	Q9H422	HIPK3	Homeodomain-interacting protein kinase 3
TLSKD	Q16543	CDC37	Hsp90 co-chaperone Cdc37
CLTRT	P42858	HD	Huntingtin
DVSSS	P42858	HD	Huntingtin
LSALR	O00291	HIP1	Huntingtin-interacting protein 1
DAKVK	Q14520	HABP2	Hyaluronan-binding protein 2 precursor
AKVKL	Q14520	HABP2	Hyaluronan-binding protein 2 precursor
KVKLI	Q14520	HABP2	Hyaluronan-binding protein 2 precursor
VLAYV	O43820	HYAL3	Hyaluronidase-3 precursor

ELLSL	Q9BYI3	HYCCI	Hyccin
EEVLA	Q4G0P3	HYDIN	Hydrocephalus-inducing protein homolog
GIIKT	Q4G0P3	HYDIN	Hydrocephalus-inducing protein homolog
IINFY	Q4G0P3	HYDIN	Hydrocephalus-inducing protein homolog
KDQLS	Q4G0P3	HYDIN	Hydrocephalus-inducing protein homolog
RLLEI	Q6PII5	HAGHL	Hydroxyacylglutathione hydrolase-like protein
YLSAL	Q01581	HMCS1	Hydroxymethylglutaryl-CoA synthase, cytoplasmic
LGFLL	Q12908	NTCP2	Ileal sodium/bile acid cotransporter
SNIKE	A1A4Y4	IRGM	Immunity-related GTPase family M protein
FIRKS	P0DOX4	IGE	Immunoglobulin epsilon heavy chain
VSVLT	P0DOX5	IGG1	Immunoglobulin gamma-1 heavy chain
STPPT	P01876	IGHA1	Immunoglobulin heavy constant alpha 1
FIRKS	P01854	IGHE	Immunoglobulin heavy constant epsilon
VSVLT	P01857	IGHG1	Immunoglobulin heavy constant gamma 1
VSVLT	P01859	IGHG2	Immunoglobulin heavy constant gamma 2
VSNKG	P01859	IGHG2	Immunoglobulin heavy constant gamma 2
VSVLT	P01860	IGHG3	Immunoglobulin heavy constant gamma 3
VSVLT	P01861	IGHG4	Immunoglobulin heavy constant gamma 4
VSNKG	P01861	IGHG4	Immunoglobulin heavy constant gamma 4
QQSYS	P01597	KV139	Immunoglobulin kappa variable 1-39 precursor
QQSYS	P04432	KVD39	Immunoglobulin kappa variable 1D-39 precursor
SLSNG	Q8TDY8	IGDC4	Immunoglobulin superfamily DCC subclass member 4 precursor
SVITS	Q6WRI0	IGS10	Immunoglobulin superfamily member 10 precursor

DASIS	Q6WRI0	IGS10	Immunoglobulin superfamily member 10 precursor
LLSTN	Q93033	IGSF2	Immunoglobulin superfamily member 2 precursor
AKKTN	Q71H61	ILDR2	Immunoglobulin-like domain-containing receptor 2 precursor
GVAVS	A9QM74	IMA8	Importin subunit alpha-8
LGFLL	O94829	IPO13	Importin-13
FPSDE	O94829	IPO13	Importin-13
SALLS	Q8IZJ6	TDH	Inactive L-threonine 3-dehydrogenase, mitochondrial precursor
LEGEV	Q15111	PLCL1	Inactive phospholipase C-like protein 1
QSTCS	Q8IWB6	TEX14	Inactive serine/threonine-protein kinase TEX14
KAVVS	Q8IWB6	TEX14	Inactive serine/threonine-protein kinase TEX14
VLDLK	Q8IWB6	TEX14	Inactive serine/threonine-protein kinase TEX14
LSLIN	Q96N87	S6A18	Inactive sodium-dependent neutral amino acid transporter B(0)AT3
SLIND	Q96N87	S6A18	Inactive sodium-dependent neutral amino acid transporter B(0)AT3
TPVST	Q70EL1	UBP54	Inactive ubiquitin carboxyl-terminal hydrolase 54
ASGVA	Q8NI35	INADL	InaD-like protein
SVSFF	Q9Y6W8	ICOS	Inducible T-cell costimulator precursor
LTLPS	P58166	INHBE	Inhibin beta E chain precursor
NEKIN	Q9P2D0	IBTK	Inhibitor of Bruton tyrosine kinase
ALLST	Q9NXR8	ING3	Inhibitor of growth protein 3
LSNIK	O15111	IKKA	Inhibitor of nuclear factor kappa-B kinase subunit alpha
SNIKE	O15111	IKKA	Inhibitor of nuclear factor kappa-B kinase subunit alpha
QNITE	Q14164	IKKE	Inhibitor of nuclear factor kappa-B kinase subunit epsilon
TREFS	Q9NQS7	INCE	Inner centromere protein

STPPT	Q53TQ3	IN80D	INO80 complex subunit D
LPIVN	P20839	IMDH1	Inosine-5'-monophosphate dehydrogenase 1
LPIVN	P12268	IMDH2	Inosine-5'-monophosphate dehydrogenase 2
STCSA	Q9Y6F6	IRAG1	Inositol 1,4,5-triphosphate receptor associated 1
ILTAV	Q14643	ITPR1	Inositol 1,4,5-trisphosphate receptor type 1
ILTAV	Q14571	ITPR2	Inositol 1,4,5-trisphosphate receptor type 2
KNRGI	Q14571	ITPR2	Inositol 1,4,5-trisphosphate receptor type 2
VLDLK	Q92551	IP6K1	Inositol hexakisphosphate kinase 1
VLDLK	Q9UHH9	IP6K2	Inositol hexakisphosphate kinase 2
VLDLK	Q96PC2	IP6K3	Inositol hexakisphosphate kinase 3
SKKRK	O15327	INP4B	Inositol polyphosphate 4-phosphatase type II
KDQLS	Q9BT40	INP5K	Inositol polyphosphate 5-phosphatase K
LGFLL	Q01968	OCRL	Inositol polyphosphate 5-phosphatase OCRL
GFLLG	Q01968	OCRL	Inositol polyphosphate 5-phosphatase OCRL
VVQLP	Q9H8X2	IPPK	Inositol-pentakisphosphate 2-kinase
VQLPL	Q9H8X2	IPPK	Inositol-pentakisphosphate 2-kinase
FYDPL	Q6UW32	IGFL1	Insulin growth factor-like family member 1 precursor
STPPT	P01344	IGF2	Insulin-like growth factor II precursor
LHLEG	P35858	ALS	Insulin-like growth factor-binding protein complex acid labile subunit precursor
TREFS	Q9Y581	INSL6	Insulin-like peptide INSL6 precursor
INNIA	Q86V85	GP180	Integral membrane protein GPR180 precursor
SALLS	Q8N201	INT1	Integrator complex subunit 1

RTDRG	Q8N201	INT1	Integrator complex subunit 1
KVLDL	Q9NVR2	INT10	Integrator complex subunit 10
VSSSV	Q96CB8	INT12	Integrator complex subunit 12
LLPIV	Q9H0H0	INT2	Integrator complex subunit 2
KKLMS	Q96HW7	INT4	Integrator complex subunit 4
VVSLS	Q6P9B9	INT5	Integrator complex subunit 5
SELLS	Q75QN2	INT8	Integrator complex subunit 8
KKRKR	Q9NV88	INT9	Integrator complex subunit 9
ISQVN	P56199	ITA1	Integrin alpha-1 precursor
VILLS	P56199	ITA1	Integrin alpha-1 precursor
GSAIA	P17301	ITA2	Integrin alpha-2 precursor
MELLI	P26006	ITA3	Integrin alpha-3 precursor
GSAIA	P26006	ITA3	Integrin alpha-3 precursor
GVSVL	P08648	ITA5	Integrin alpha-5 precursor
SSSVI	Q13797	ITA9	Integrin alpha-9 precursor
VTELQ	P38570	ITAE	Integrin alpha-E precursor
GSAIA	P08514	ITA2B	Integrin alpha-IIb precursor
VSVGN	P08514	ITA2B	Integrin alpha-IIb precursor
GSAIA	P06756	ITAV	Integrin alpha-V precursor
SNICL	P20702	ITAX	Integrin alpha-X precursor
LKANA	P05556	ITB1	Integrin beta-1 precursor
SVLTS	O14713	ITBP1	Integrin beta-1-binding protein 1
VITIE	P16144	ITB4	Integrin beta-4 precursor

TTIII	P16144	ITB4	Integrin beta-4 precursor
TNVTL	P26010	ITB7	Integrin beta-7 precursor
NKIKS	P26012	ITB8	Integrin beta-8 precursor
NVQIV	Q06033	ITIH3	Inter-alpha-trypsin inhibitor heavy chain H3 precursor
LTAVT	Q14624	ITIH4	Inter-alpha-trypsin inhibitor heavy chain H4 precursor
TAVTF	Q14624	ITIH4	Inter-alpha-trypsin inhibitor heavy chain H4 precursor
AGVTT	Q6UXX5	ITIH6	Inter-alpha-trypsin inhibitor heavy chain H6 precursor
GVTTP	Q6UXX5	ITIH6	Inter-alpha-trypsin inhibitor heavy chain H6 precursor
DVSSS	Q6UXX5	ITIH6	Inter-alpha-trypsin inhibitor heavy chain H6 precursor
ILLSL	Q6UXX5	ITIH6	Inter-alpha-trypsin inhibitor heavy chain H6 precursor
GVGSA	Q14773	ICAM4	Intercellular adhesion molecule 4 precursor
GSAIA	P40305	IFI27	Interferon alpha-inducible protein 27, mitochondrial precursor
LSNGV	P14316	IRF2	Interferon regulatory factor 2
SNGVS	P14316	IRF2	Interferon regulatory factor 2
CNVDI	P20592	MX2	Interferon-induced GTP-binding protein Mx2
KNNRL	Q9BYX4	IFIH1	Interferon-induced helicase C domain-containing protein 1
LSALR	Q8TCB0	IFI44	Interferon-induced protein 44
SALRT	Q8TCB0	IFI44	Interferon-induced protein 44
VNAGV	Q8TCB0	IFI44	Interferon-induced protein 44
LSALR	Q53G44	IF44L	Interferon-induced protein 44-like
VRQQS	Q7Z2Y8	GVIN1	Interferon-induced very large GTPase 1
NICLT	Q7Z2Y8	GVIN1	Interferon-induced very large GTPase 1
ILKAN	O75569	PRKRA	Interferon-inducible double-stranded RNA-dependent protein kinase

			activator A
LKANA	O75569	PRKRA	Interferon-inducible double-stranded RNA-dependent protein kinase activator A
TPVST	P14778	IL1R1	Interleukin-1 receptor type 1 precursor
KIKSA	Q5VVH5	IKBP1	Interleukin-1 receptor-associated kinase 1-binding protein 1
FASGQ	Q01638	ILRL1	Interleukin-1 receptor-like 1 precursor
LTAVT	Q13651	I10R1	Interleukin-10 receptor subunit alpha precursor
SNKGM	Q13651	I10R1	Interleukin-10 receptor subunit alpha precursor
LQLLM	P20809	IL11	Interleukin-11 precursor
GVSVL	P42701	I12R1	Interleukin-12 receptor subunit beta-1 precursor
AVTEL	P78552	I13R1	Interleukin-13 receptor subunit alpha-1 precursor
VTELQ	P78552	I13R1	Interleukin-13 receptor subunit alpha-1 precursor
FLGFL	Q969J5	I22R2	Interleukin-22 receptor subunit alpha-2 precursor
NSELL	Q9H293	IL25	Interleukin-25 precursor
ALLST	P05113	IL5	Interleukin-5 precursor
CTTNT	Q01344	IL5RA	Interleukin-5 receptor subunit alpha precursor
NRLLE	Q5TF58	IFFO2	Intermediate filament family orphan 2
TTPVS	Q17R60	IMPG1	Interphotoreceptor matrix proteoglycan 1 precursor
NSELL	Q17R60	IMPG1	Interphotoreceptor matrix proteoglycan 1 precursor
LTSKV	Q9BZV3	IMPG2	Interphotoreceptor matrix proteoglycan 2 precursor
SLTLP	Q9BZV3	IMPG2	Interphotoreceptor matrix proteoglycan 2 precursor
SQVNE	Q15811	ITSN1	Intersectin-1
SQVNE	Q9NZM3	ITSN2	Intersectin-2

AITTI	P03956	MMP1	Interstitial collagenase precursor
KKLMS	A0AVF1	IFT56	Intraflagellar transport protein 56
EKINQ	Q96LB3	IFT74	Intraflagellar transport protein 74 homolog
VSLSN	Q9P2H3	IFT80	Intraflagellar transport protein 80 homolog
PVTLS	P07476	INVO	Involucrin
LPSEI	O60928	KCJ13	Inward rectifier potassium channel 13
DELLH	Q15051	IQCB1	IQ calmodulin-binding motif-containing protein 1
SNIKE	Q4KMZ1	IQCC	IQ domain-containing protein C
SALLS	Q6IPM2	IQCE	IQ domain-containing protein E
NKAVV	Q9H0B3	IQCN	IQ domain-containing protein N
RARRE	A8MXQ7	IQAK1	IQ motif and ankyrin repeat domain-containing protein 1
ARREL	A8MXQ7	IQAK1	IQ motif and ankyrin repeat domain-containing protein 1
LDLKN	Q9UPP2	IQEC3	IQ motif and SEC7 domain-containing protein 3
SLTLP	P48200	IREB2	Iron-responsive element-binding protein 2
VNKIK	Q9BUE6	ISCA1	Iron-sulfur cluster assembly 1 homolog, mitochondrial precursor
RLLEI	Q86U28	ISCA2	Iron-sulfur cluster assembly 2 homolog, mitochondrial precursor
LLEIT	Q86U28	ISCA2	Iron-sulfur cluster assembly 2 homolog, mitochondrial precursor
GSAIA	Q9H1K1	ISCU	Iron-sulfur cluster assembly enzyme ISCU, mitochondrial precursor
SAIAS	Q9H1K1	ISCU	Iron-sulfur cluster assembly enzyme ISCU, mitochondrial precursor
GKSTT	Q8TB37	NUBPL	Iron-sulfur protein NUBPL precursor
KEEVL	Q6UXV1	IZUM2	Izumo sperm-egg fusion protein 2 precursor
IEFQQ	Q96N16	JKIP1	Janus kinase and microtubule-interacting protein 1
LSALR	Q5VZ66	JKIP3	Janus kinase and microtubule-interacting protein 3

RRELP	Q5VZ66	JKIP3	Janus kinase and microtubule-interacting protein 3
SLAFI	Q5VZ66	JKIP3	Janus kinase and microtubule-interacting protein 3
KVKLI	Q9Y624	JAM1	Junctional adhesion molecule A precursor
SNKNR	Q9P266	JCAD	Junctional protein associated with coronary artery disease
NKNRG	Q9P266	JCAD	Junctional protein associated with coronary artery disease
VKGEP	Q9P266	JCAD	Junctional protein associated with coronary artery disease
SNIET	O60229	KALRN	Kalirin
LSLIA	Q92876	KLK6	Kallikrein-6 precursor
DVSSS	Q7Z3B3	KANL1	KAT8 regulatory NSL complex subunit 1
SLGAI	Q9P2N6	KANL3	KAT8 regulatory NSL complex subunit 3
GLLLY	Q8IYT4	KATL2	Katanin p60 ATPase-containing subunit A-like 2
EEFYQ	Q9NR64	KLHL1	Kelch-like protein 1
LAFIR	Q9NR64	KLHL1	Kelch-like protein 1
ALLST	Q9P2N7	KLH13	Kelch-like protein 13
NVTLS	Q9P2G3	KLH14	Kelch-like protein 14
TIELS	Q96M94	KLH15	Kelch-like protein 15
TRTDR	Q6TDP4	KLH17	Kelch-like protein 17
MELLI	Q9Y2M5	KLH20	Kelch-like protein 20
GVSVL	Q9Y2M5	KLH20	Kelch-like protein 20
FYDPL	Q8NBE8	KLH23	Kelch-like protein 23
ELLSL	Q0D2K2	KLH30	Kelch-like protein 30
LPSEI	Q9H511	KLH31	Kelch-like protein 31
SGVAV	Q96NJ5	KLH32	Kelch-like protein 32

SGVAV	Q8N4N3	KLH36	Kelch-like protein 36
IFNPK	O60662	KLH41	Kelch-like protein 41
SNNVQ	Q8IXQ5	KLHL7	Kelch-like protein 7
ALLST	Q9P2J3	KLHL9	Kelch-like protein 9
ELLSL	Q14525	KT33B	Keratin, type I cuticular Ha3-II
LTRTD	Q99456	K1C12	Keratin, type I cytoskeletal 12
AGSVS	P13646	K1C13	Keratin, type I cytoskeletal 13
TNSEL	P02533	K1C14	Keratin, type I cytoskeletal 14
TNSEL	Q04695	K1C17	Keratin, type I cytoskeletal 17
ELDKY	P05783	K1C18	Keratin, type I cytoskeletal 18
LGVGS	Q14533	KRT81	Keratin, type II cuticular Hb1
AVSKG	Q9NSB4	KRT82	Keratin, type II cuticular Hb2
INQSL	P04264	K2C1	Keratin, type II cytoskeletal 1
INQSL	Q7Z794	K2C1B	Keratin, type II cytoskeletal 1b
INQSL	P12035	K2C3	Keratin, type II cytoskeletal 3
INQSL	P19013	K2C4	Keratin, type II cytoskeletal 4
INQSL	P08729	K2C7	Keratin, type II cytoskeletal 7
PVSTY	Q3LI76	KR151	Keratin-associated protein 15-1
LSGIN	Q9BYR8	KRA31	Keratin-associated protein 3-1
SCSIS	Q9BYQ6	KR411	Keratin-associated protein 4-11
SCSIS	Q9BYQ5	KRA46	Keratin-associated protein 4-6
SCSIS	Q9BYQ9	KRA48	Keratin-associated protein 4-8
SCSIS	Q9BYQ8	KRA49	Keratin-associated protein 4-9

ETVIE	Q8N1A0	KT222	Keratin-like protein KRT222
NKIKS	O60938	KERA	Keratocan precursor
AVVSL	Q9Y664	KPTN	KICSTOR complex protein kaptin
ELQLL	Q5T011	SZT2	KICSTOR complex protein SZT2
AVVSL	Q96MD2	KICS2	KICSTOR subunit 2
RARRE	Q6ZWJ8	KCP	Kielin/chordin-like protein precursor
NVTLS	P43626	KI2L1	Killer cell immunoglobulin-like receptor 2DL1 precursor
NVTLS	Q99706	KI2L4	Killer cell immunoglobulin-like receptor 2DL4 precursor
NVTLS	Q8N109	KI2LA	Killer cell immunoglobulin-like receptor 2DL5A precursor
NVTLS	Q8NHK3	KI2LB	Killer cell immunoglobulin-like receptor 2DL5B precursor
NVTLS	Q14954	KI2S1	Killer cell immunoglobulin-like receptor 2DS1 precursor
NVTLS	P43632	KI2S4	Killer cell immunoglobulin-like receptor 2DS4 precursor
NVTLS	P43630	KI3L2	Killer cell immunoglobulin-like receptor 3DL2 precursor
NVTLS	Q8N743	KI3L3	Killer cell immunoglobulin-like receptor 3DL3 precursor
ALLST	Q76NI1	KNDC1	Kinase non-catalytic C-lobe domain-containing protein 1
ELLIL	Q92845	KIFA3	Kinesin-associated protein 3
INQSL	P52732	KIF11	Kinesin-like protein KIF11
KRKRR	Q9H1H9	KI13A	Kinesin-like protein KIF13A
KEGSN	Q9H1H9	KI13A	Kinesin-like protein KIF13A
EGSNI	Q9H1H9	KI13A	Kinesin-like protein KIF13A
GAIVS	Q9H1H9	KI13A	Kinesin-like protein KIF13A
EFSVN	Q9NQT8	KI13B	Kinesin-like protein KIF13B
KEGSN	Q9NQT8	KI13B	Kinesin-like protein KIF13B

EGSNI	Q9NQT8	KI13B	Kinesin-like protein KIF13B
NKAVV	Q9NS87	KIF15	Kinesin-like protein KIF15
SALLS	Q9P2E2	KIF17	Kinesin-like protein KIF17
AKVKL	Q2TAC6	KIF19	Kinesin-like protein KIF19
TCSAV	Q12756	KIF1A	Kinesin-like protein KIF1A
SKKRK	O43896	KIF1C	Kinesin-like protein KIF1C
SAIAS	O95235	KI20A	Kinesin-like protein KIF20A
KSALL	Q96Q89	KI20B	Kinesin-like protein KIF20B
PSEIN	Q96Q89	KI20B	Kinesin-like protein KIF20B
KVLDL	Q14807	KIF22	Kinesin-like protein KIF22
INQSL	Q02241	KIF23	Kinesin-like protein KIF23
INQSL	Q5T7B8	KIF24	Kinesin-like protein KIF24
GVTTP	Q9ULI4	KI26A	Kinesin-like protein KIF26A
DEFDA	Q9ULI4	KI26A	Kinesin-like protein KIF26A
VITIE	O15066	KIF3B	Kinesin-like protein KIF3B
LSKDQ	O15066	KIF3B	Kinesin-like protein KIF3B
AKVKL	Q6ZMV9	KIF6	Kinesin-like protein KIF6
LQLLM	Q6ZMV9	KIF6	Kinesin-like protein KIF6
LLILK	Q9HAQ2	KIF9	Kinesin-like protein KIF9
NKIKS	Q8NG31	KNL1	Kinetochore scaffold 1
SLIAV	P50748	KNTC1	Kinetochore-associated protein 1
STPVT	Q14678	KANK1	KN motif and ankyrin repeat domain-containing protein 1
SELLS	O00522	KRIT1	Krev interaction trapped protein 1

LLSTN	O14901	KLF11	Krueppel-like factor 11
VKGEP	Q9Y4X4	KLF12	Krueppel-like factor 12
IKQEL	Q13887	KLF5	Krueppel-like factor 5
SVLTS	O95600	KLF8	Krueppel-like factor 8
NAGSV	O95600	KLF8	Krueppel-like factor 8
LGFLL	Q6UDR6	SPIT4	Kunitz-type protease inhibitor 4 precursor
GSVSF	Q9NS61	KCIP2	Kv channel-interacting protein 2
TPVTL	P42167	LAP2B	Lamina-associated polypeptide 2, isoforms beta/gamma
NAGVT	P20700	LMNB1	Lamin-B1 precursor
AGSVS	P25391	LAMA1	Laminin subunit alpha-1 precursor
GLLLY	P25391	LAMA1	Laminin subunit alpha-1 precursor
TPVTL	P25391	LAMA1	Laminin subunit alpha-1 precursor
EGKSL	Q16787	LAMA3	Laminin subunit alpha-3 precursor
LEGEV	P07942	LAMB1	Laminin subunit beta-1 precursor
VVSLS	P07942	LAMB1	Laminin subunit beta-1 precursor
NAGVT	Q13753	LAMC2	Laminin subunit gamma-2 precursor
ILLSL	Q96RQ9	OXLA	L-amino-acid oxidase precursor
QSTPP	Q92615	LAR4B	La-related protein 4B
VLHLE	Q92615	LAR4B	La-related protein 4B
NAGSV	Q9UHI5	LAT2	Large neutral amino acids transporter small subunit 2
LGFLL	O75387	LAT3	Large neutral amino acids transporter small subunit 3
SLTLP	O75387	LAT3	Large neutral amino acids transporter small subunit 3
SLTLP	Q8N370	LAT4	Large neutral amino acids transporter small subunit 4

VGLLL	Q8N370	LAT4	Large neutral amino acids transporter small subunit 4
QLLMQ	P46379	BAG6	Large proline-rich protein BAG6
ARREL	Q8N3Y3	LARG2	LARGE xylosyl- and glucuronyltransferase 2
LGFLL	Q8N3Y3	LARG2	LARGE xylosyl- and glucuronyltransferase 2
ELLSL	Q8N3Y3	LARG2	LARGE xylosyl- and glucuronyltransferase 2
LPLYG	Q8NBF6	AVL9	Late secretory pathway protein AVL9 homolog
IFNPK	Q9HCC9	LST2	Lateral signaling target protein 2 homolog
DNAGS	A0A0U1RRK4	LBHD2	LBH domain-containing protein 2
IKTFS	Q0VAK6	LMOD3	Leiomodin-3
SLTLP	A0A1B0GTW7	LMLN2	Leishmanolysin-like peptidase 2 precursor
LTLPS	A0A1B0GTW7	LMLN2	Leishmanolysin-like peptidase 2 precursor
LTAVT	Q8NC56	LEMD2	LEM domain-containing protein 2
ASGVA	Q15334	L2GL1	Lethal(2) giant larvae protein homolog 1
KKRKR	Q9Y468	LMBL1	Lethal(3)malignant brain tumor-like protein 1
KRKRR	Q9Y468	LMBL1	Lethal(3)malignant brain tumor-like protein 1
KKRKR	Q969R5	LMBL2	Lethal(3)malignant brain tumor-like protein 2
SEINL	Q96JM7	LMBL3	Lethal(3)malignant brain tumor-like protein 3
LLSTN	Q6P1Q0	LTMD1	LETM1 domain-containing protein 1
LLHNV	Q6P1Q0	LTMD1	LETM1 domain-containing protein 1
KLHTS	Q2VYF4	LETM2	LETM1 domain-containing protein LETM2, mitochondrial precursor
LHLEG	P02750	A2GL	Leucine-rich alpha-2-glycoprotein precursor
ICLTR	O95970	LGI1	Leucine-rich glioma-inactivated protein 1 precursor

INNIA	P42704	LPPRC	Leucine-rich PPR motif-containing protein, mitochondrial precursor
LPSEI	A4D1F6	LRRD1	Leucine-rich repeat and death domain-containing protein 1
SALLS	Q9P244	LRFN1	Leucine-rich repeat and fibronectin type III domain-containing protein 1 precursor
IVILL	Q96FE5	LIGO1	Leucine-rich repeat and immunoglobulin-like domain-containing nogo receptor-interacting protein 1 precursor
IVILL	Q7L985	LIGO2	Leucine-rich repeat and immunoglobulin-like domain-containing nogo receptor-interacting protein 2 precursor
SKKRK	Q96JM4	LRIQ1	Leucine-rich repeat and IQ domain-containing protein 1
TELQL	Q8N967	LRTM2	Leucine-rich repeat and transmembrane domain-containing protein 2 precursor
EEVLA	Q32MZ4	LRRF1	Leucine-rich repeat flightless-interacting protein 1
TPVST	Q96L50	LLR1	Leucine-rich repeat protein 1
SIMSI	Q9UQ13	SHOC2	Leucine-rich repeat protein SHOC-2
NSLTL	Q9UQ13	SHOC2	Leucine-rich repeat protein SHOC-2
RLLEI	Q38SD2	LRRK1	Leucine-rich repeat serine/threonine-protein kinase 1
KLIKQ	Q5S007	LRRK2	Leucine-rich repeat serine/threonine-protein kinase 2
LSKKR	Q5S007	LRRK2	Leucine-rich repeat serine/threonine-protein kinase 2
SKKRK	Q5S007	LRRK2	Leucine-rich repeat serine/threonine-protein kinase 2
NRLLE	Q5S007	LRRK2	Leucine-rich repeat serine/threonine-protein kinase 2
RLLEI	Q5S007	LRRK2	Leucine-rich repeat serine/threonine-protein kinase 2
GLLLY	Q5S007	LRRK2	Leucine-rich repeat serine/threonine-protein kinase 2
GKSTT	Q86VH5	LRRT3	Leucine-rich repeat transmembrane neuronal protein 3 precursor

VLDLK	A6NDA9	LRIT2	Leucine-rich repeat, immunoglobulin-like domain and transmembrane domain-containing protein 2 precursor
SLTLP	A6NHZ5	LR14B	Leucine-rich repeat-containing protein 14B
IVILL	Q8N6Y2	LRC17	Leucine-rich repeat-containing protein 17 precursor
NYIDK	Q8N456	LRC18	Leucine-rich repeat-containing protein 18
LHLEG	Q8TCA0	LRC20	Leucine-rich repeat-containing protein 20
TLPSE	A6NM36	LRC30	Leucine-rich repeat-containing protein 30
SLTLP	A6NMS7	L37A1	Leucine-rich repeat-containing protein 37A precursor
SLTLP	A6NM11	L37A2	Leucine-rich repeat-containing protein 37A2 precursor
SLTLP	O60309	L37A3	Leucine-rich repeat-containing protein 37A3 precursor
SGVAV	Q5VT99	LRC38	Leucine-rich repeat-containing protein 38 precursor
GVAVS	Q5VT99	LRC38	Leucine-rich repeat-containing protein 38 precursor
TLPSE	Q9H9A6	LRC40	Leucine-rich repeat-containing protein 40
VLAYV	Q96CN5	LRC45	Leucine-rich repeat-containing protein 45
SVGNT	Q9HCJ2	LRC4C	Leucine-rich repeat-containing protein 4C precursor
GEVNK	Q96E66	LRC51	Leucine-rich repeat-containing protein 51
ILLSL	A6NM62	LRC53	Leucine-rich repeat-containing protein 53
KNNRL	Q96CX6	LRC58	Leucine-rich repeat-containing protein 58
VTELQ	Q96AG4	LRC59	Leucine-rich repeat-containing protein 59
SGINN	Q7Z2Q7	LRR70	Leucine-rich repeat-containing protein 70 precursor
NVDIF	Q8NAA5	LR75A	Leucine-rich repeat-containing protein 75A
SDELL	Q2VPJ9	LR75B	Leucine-rich repeat-containing protein 75B
DELLH	Q2VPJ9	LR75B	Leucine-rich repeat-containing protein 75B

VLHLE	Q96JA1	LRIG1	Leucine-rich repeats and immunoglobulin-like domains protein 1 precursor
NKIKS	Q96JA1	LRIG1	Leucine-rich repeats and immunoglobulin-like domains protein 1 precursor
IVIIV	O94898	LRIG2	Leucine-rich repeats and immunoglobulin-like domains protein 2 precursor
LDLKN	Q6UXM1	LRIG3	Leucine-rich repeats and immunoglobulin-like domains protein 3 precursor
GIIKT	Q9P2J5	SYLC	Leucine--tRNA ligase, cytoplasmic
ETVIE	P42702	LIFR	Leukemia inhibitory factor receptor precursor
PVTLS	Q8N423	LIRB2	Leukocyte immunoglobulin-like receptor subfamily B member 2 precursor
IKENK	P19397	CD53	Leukocyte surface antigen CD53
KENKC	P19397	CD53	Leukocyte surface antigen CD53
TSKTD	P29376	LTK	Leukocyte tyrosine kinase receptor precursor
FLGFL	P09960	LKHA4	Leukotriene A-4 hydrolase
VLHLE	Q8N0W3	FCSK	L-fucose kinase
QKKLM	Q8N0W3	FCSK	L-fucose kinase
SLTLP	Q8N0W3	FCSK	L-fucose kinase
QLLPI	Q5T3J3	LRIF1	Ligand-dependent nuclear receptor-interacting factor 1
RGIIK	P53667	LIMK1	LIM domain kinase 1
NKIKS	O75112	LDB3	LIM domain-binding protein 3
KIKSA	O75112	LDB3	LIM domain-binding protein 3

GKSTT	O75112	LDB3	LIM domain-binding protein 3
ELLSL	Q8TE12	LMX1A	LIM homeobox transcription factor 1-alpha
SVLTS	P50458	LHX2	LIM/homeobox protein Lhx2
TKCTA	Q969G2	LHX4	LIM/homeobox protein Lhx4
STPVT	Q68G74	LHX8	LIM/homeobox protein Lhx8
RKSDE	Q8WVP7	LMBR1	Limb region 1 protein homolog
VLDLK	Q5T7N2	LITD1	LINE-1 type transposase domain-containing protein 1
KEEVL	Q5T7N2	LITD1	LINE-1 type transposase domain-containing protein 1
IKTFS	Q5T7N2	LITD1	LINE-1 type transposase domain-containing protein 1
ELLSL	Q9BU23	LMF2	Lipase maturation factor 2
LGFLL	Q5VXI9	LIPN	Lipase member N precursor
STPVT	Q93052	LPP	Lipoma-preferred partner
NRVFC	P50851	LRBA	Lipopolysaccharide-responsive and beige-like anchor protein
GTDAK	Q8IVV2	LOXH1	Lipoxygenase homology domain-containing protein 1
TDAKV	Q8IVV2	LOXH1	Lipoxygenase homology domain-containing protein 1
VSVLT	Q86W92	LIPB1	Liprin-beta-1
KSALL	Q8ND30	LIPB2	Liprin-beta-2
VSVLT	Q8ND30	LIPB2	Liprin-beta-2
VSVLT	P05451	REG1A	Lithostathine-1-alpha precursor
VSVLT	P48304	REG1B	Lithostathine-1-beta precursor
QNITE	Q9Y2F5	ICE1	Little elongation complex subunit 1
SGVAV	Q9Y2F5	ICE1	Little elongation complex subunit 1
DVSSS	Q9Y2F5	ICE1	Little elongation complex subunit 1

AIVSC	Q9Y2F5	ICE1	Little elongation complex subunit 1
SKKRK	Q659A1	ICE2	Little elongation complex subunit 2
SLSNG	Q6P1M3	L2GL2	LLGL scribble cell polarity complex component 2
VVQLP	Q6P1M3	L2GL2	LLGL scribble cell polarity complex component 2
FMNYT	Q9Y2P4	S27A6	Long-chain fatty acid transport protein 6
LLMQS	Q9UKU0	ACSL6	Long-chain-fatty-acid--CoA ligase 6
KRKRR	Q9NZR2	LRP1B	Low-density lipoprotein receptor-related protein 1B precursor
QSTCS	P98164	LRP2	Low-density lipoprotein receptor-related protein 2 precursor
LGFLL	P98164	LRP2	Low-density lipoprotein receptor-related protein 2 precursor
KSDEL	O75581	LRP6	Low-density lipoprotein receptor-related protein 6 precursor
AGKST	Q8IV42	PSTK	L-seryl-tRNA(Sec) kinase
ELLIL	Q8IYD9	LAS2	Lung adenoma susceptibility protein 2 precursor
LLILK	Q8IYD9	LAS2	Lung adenoma susceptibility protein 2 precursor
LILKA	Q8IYD9	LAS2	Lung adenoma susceptibility protein 2 precursor
LEGEV	P05455	LA	Lupus La protein
LCNVD	Q6UXB3	LYPD2	Ly6/PLAUR domain-containing protein 2 precursor
TPVST	O60449	LY75	Lymphocyte antigen 75 precursor
IKEEV	O60449	LY75	Lymphocyte antigen 75 precursor
DASIS	Q9Y6Y9	LY96	Lymphocyte antigen 96 precursor
SLSNG	Q8IWV1	LAX1	Lymphocyte transmembrane adapter 1
KKRKR	Q9UJU2	LEF1	Lymphoid enhancer-binding factor 1
TIELS	Q9NRZ9	HELLS	Lymphoid-specific helicase
WYTSV	Q06643	TNFC	Lymphotoxin-beta

SVLTS	Q9Y2K7	KDM2A	Lysine-specific demethylase 2A
KTFSN	Q7LBC6	KDM3B	Lysine-specific demethylase 3B
CKIMT	O94953	KDM4B	Lysine-specific demethylase 4B
CKIMT	Q9H3R0	KDM4C	Lysine-specific demethylase 4C
KKRKR	P29375	KDM5A	Lysine-specific demethylase 5A
NSELL	P41229	KDM5C	Lysine-specific demethylase 5C
NSELL	Q9BY66	KDM5D	Lysine-specific demethylase 5D
LILKA	O15550	KDM6A	Lysine-specific demethylase 6A
SKKRK	O75151	PHF2	Lysine-specific demethylase PHF2
NRLLE	O60341	KDM1A	Lysine-specific histone demethylase 1A
LGFLL	Q9H1C0	LPAR5	Lysophosphatidic acid receptor 5
PVTLS	P43657	LPAR6	Lysophosphatidic acid receptor 6
TASNK	Q7L5N7	PCAT2	Lysophosphatidylcholine acyltransferase 2
TSLGA	Q6P1A2	MBOA5	Lysophospholipid acyltransferase 5
LSALR	Q8N661	TM86B	Lysoplasmalogenase
ALRTG	Q8N2M4	TM86A	Lysoplasmalogenase-like protein TMEM86A
LGFLL	Q8N2M4	TM86A	Lysoplasmalogenase-like protein TMEM86A
LSNGV	P10253	LYAG	Lysosomal alpha-glucosidase precursor
GNTLY	Q6ZP29	LAAT1	Lysosomal amino acid transporter 1 homolog
LHLEG	Q99698	LYST	Lysosomal-trafficking regulator
LSNGV	Q99698	LYST	Lysosomal-trafficking regulator
SNGVS	Q99698	LYST	Lysosomal-trafficking regulator
AVGLL	Q99698	LYST	Lysosomal-trafficking regulator

VFPSD	Q14108	SCRB2	Lysosome membrane protein 2
NVTLS	P0DSE2	TRBR1	M1-specific T cell receptor beta chain precursor
VIIVI	P22897	MRC1	Macrophage mannose receptor 1 precursor
IIVIL	P22897	MRC1	Macrophage mannose receptor 1 precursor
IVILL	P22897	MRC1	Macrophage mannose receptor 1 precursor
AVGLL	A6NGR9	MROH6	Maestro heat-like repeat-containing protein family member 6
LLILK	Q5TGP6	MROH9	Maestro heat-like repeat-containing protein family member 9
TVSVG	Q7RTP0	NIPA1	Magnesium transporter NIPA1
LLSLI	Q8N8Q9	NIPA2	Magnesium transporter NIPA2
NGVSV	Q96JB8	MPP4	MAGUK p55 subfamily member 4
GLLLY	Q14728	MFS10	Major facilitator superfamily domain-containing protein 10
SALLS	Q96ES6	MFSD3	Major facilitator superfamily domain-containing protein 3
GAIVS	Q8IWD5	MFS6L	Major facilitator superfamily domain-containing protein 6-like
FLLGV	Q9UPX6	MNAR1	Major intrinsically disordered Notch2-binding receptor 1
VITIE	Q14764	MVP	Major vault protein
STYML	Q14764	MVP	Major vault protein
TYMLT	Q14764	MVP	Major vault protein
AKVKL	P40925	MDHC	Malate dehydrogenase, cytoplasmic
ARSTP	Q9Y4C4	MFHA1	Malignant fibrous histiocytoma-amplified sequence 1
TTPVS	O43451	MGA	Maltase-glucoamylase, intestinal
LGVGS	Q5VYJ5	MALR1	MAM and LDL-receptor class A domain-containing protein 1 precursor
IKENK	O60476	MA1A2	Mannosyl-oligosaccharide 1,2-alpha-mannosidase IB

NNRLL	Q16644	MAPK3	MAP kinase-activated protein kinase 3
AKVKL	P27448	MARK3	MAP/microtubule affinity-regulating kinase 3
AKVKL	Q96L34	MARK4	MAP/microtubule affinity-regulating kinase 4
PLVFP	P57077	M3KCL	MAP3K7 C-terminal-like protein
NNAKK	Q8IWC1	MA7D3	MAP7 domain-containing protein 3
KLHTS	Q8WU39	MZB1	Marginal zone B- and B1-cell-specific protein precursor
ITTII	Q8N4S9	MALD2	MARVEL domain-containing protein 2
TTIII	Q8N4S9	MALD2	MARVEL domain-containing protein 2
ITSLG	Q96A59	MALD3	MARVEL domain-containing protein 3
DVSSS	P10721	KIT	Mast/stem cell growth factor receptor Kit precursor
VSSSV	P10721	KIT	Mast/stem cell growth factor receptor Kit precursor
QEGKS	P10721	KIT	Mast/stem cell growth factor receptor Kit precursor
AKVKL	Q14680	MELK	Maternal embryonic leucine zipper kinase
NIAFS	O00339	MATN2	Matrilin-2 precursor
VGLLL	Q9NQ76	MEPE	Matrix extracellular phosphoglycoprotein precursor
LLYCK	P51511	MMP15	Matrix metalloproteinase-15 precursor
LGFLL	Q99542	MMP19	Matrix metalloproteinase-19 precursor
VGLLL	Q9H239	MMP28	Matrix metalloproteinase-28 precursor
LHLEG	Q9NR99	MXRA5	Matrix-remodeling-associated protein 5 precursor
CTASN	Q9NR99	MXRA5	Matrix-remodeling-associated protein 5 precursor
LLGVG	Q9Y6X3	SCC4	MAU2 chromatid cohesion factor homolog
VQLPL	Q8IWI9	MGAP	MAX gene-associated protein
SDEFD	Q8IWI9	MGAP	MAX gene-associated protein

LSGIN	Q8IWI9	MGAP	MAX gene-associated protein
QLPLY	Q99583	MNT	Max-binding protein MNT
TELQL	Q9NPJ1	MKKS	McKusick-Kaufman/Bardet-Biedl syndromes putative chaperonin
SALLS	Q9NPJ1	MKKS	McKusick-Kaufman/Bardet-Biedl syndromes putative chaperonin
IIKEE	Q14676	MDC1	Mediator of DNA damage checkpoint protein 1
SSSVI	Q14676	MDC1	Mediator of DNA damage checkpoint protein 1
STPPT	Q15648	MED1	Mediator of RNA polymerase II transcription subunit 1
LSLIA	Q15648	MED1	Mediator of RNA polymerase II transcription subunit 1
AVVSL	Q93074	MED12	Mediator of RNA polymerase II transcription subunit 12
LCNVD	Q93074	MED12	Mediator of RNA polymerase II transcription subunit 12
NRARR	Q9UHV7	MED13	Mediator of RNA polymerase II transcription subunit 13
SAIAS	Q9UHV7	MED13	Mediator of RNA polymerase II transcription subunit 13
SDELL	Q9UHV7	MED13	Mediator of RNA polymerase II transcription subunit 13
DELLH	Q9UHV7	MED13	Mediator of RNA polymerase II transcription subunit 13
TPVTL	Q71F56	MD13L	Mediator of RNA polymerase II transcription subunit 13-like
NSELL	Q9NVC6	MED17	Mediator of RNA polymerase II transcription subunit 17
LLPIV	Q9ULK4	MED23	Mediator of RNA polymerase II transcription subunit 23
DKYKN	P11310	ACADM	Medium-chain specific acyl-CoA dehydrogenase, mitochondrial precursor
QSTPP	Q5TIA1	MEI1	Meiosis inhibitor protein 1
GVAVS	Q5TIA1	MEI1	Meiosis inhibitor protein 1
IIKEE	Q8NEH6	MNS1	Meiosis-specific nuclear structural protein 1
LLSLI	Q8N635	MEIOB	Meiosis-specific with OB domain-containing protein

RRELP	O95072	REC8	Meiotic recombination protein REC8 homolog
ELLSL	O95072	REC8	Meiotic recombination protein REC8 homolog
SALLS	O60732	MAGC1	Melanoma-associated antigen C1
VVSLS	Q13585	MTR1L	Melatonin-related receptor
GSAIA	Q9NXK6	PAQR5	Membrane progestin receptor gamma
SAIAS	Q15049	MLC1	Membrane protein MLC1
VKLIK	Q86UL8	MAGI2	Membrane-associated guanylate kinase, WW and PDZ domain-containing protein 2
LGFLL	Q9UMX9	S45A2	Membrane-associated transporter protein
SLGAI	Q9GZW8	MS4A7	Membrane-spanning 4-domains subfamily A member 7
VNAGV	O00255	MEN1	Menin
SEINL	Q16819	MEP1A	Meprin A subunit alpha precursor
ELLSL	Q8N108	MIER1	Mesoderm induction early response protein 1
LTLPS	Q7Z3K6	MIER3	Mesoderm induction early response protein 3
VGLLL	Q13255	GRM1	Metabotropic glutamate receptor 1 precursor
ITIEL	Q14416	GRM2	Metabotropic glutamate receptor 2 precursor
VLHLE	Q14833	GRM4	Metabotropic glutamate receptor 4 precursor
ILLSL	Q13228	SBP1	Methanethiol oxidase
YGKTK	Q9NZL9	MAT2B	Methionine adenosyltransferase 2 subunit beta
FNPKY	P50579	MAP2	Methionine aminopeptidase 2
NPKYD	P50579	MAP2	Methionine aminopeptidase 2
NSELL	P56192	SYMC	Methionine--tRNA ligase, cytoplasmic
SLSNG	Q96DP5	FMT	Methionyl-tRNA formyltransferase, mitochondrial precursor

SKKRK	Q9UIS9	MBD1	Methyl-CpG-binding domain protein 1
SAIAS	O95983	MBD3	Methyl-CpG-binding domain protein 3
KQEGK	Q8WWY6	MB3L1	Methyl-CpG-binding domain protein 3-like 1
IKENK	Q8NFU7	TET1	Methylcytosine dioxygenase TET1
ARREL	Q8NFU7	TET1	Methylcytosine dioxygenase TET1
KKRKR	Q8NFU7	TET1	Methylcytosine dioxygenase TET1
QEGKS	Q6N021	TET2	Methylcytosine dioxygenase TET2
ARREL	O43151	TET3	Methylcytosine dioxygenase TET3
KKRKR	O43151	TET3	Methylcytosine dioxygenase TET3
AGKST	Q8IVH4	MMAA	Methylmalonic aciduria type A protein, mitochondrial precursor
VTELQ	P22670	RFX1	MHC class II regulatory factor RFX1
GKSTT	Q8IY33	MILK2	MICAL-like protein 2
GSVSF	P55083	MFAP4	Microfibril-associated glycoprotein 4 precursor
SVSFF	P55083	MFAP4	Microfibril-associated glycoprotein 4 precursor
TNKAV	P55081	MFAP1	Microfibrillar-associated protein 1
SPLCT	Q9Y4B5	MTCL1	Microtubule cross-linking factor 1
GVGSA	Q7Z591	AKNA	Microtubule organization protein AKNA
ELLSL	Q9UPN3	MACF1	Microtubule-actin cross-linking factor 1, isoforms 1/2/3/5
IKEEV	Q9UPN3	MACF1	Microtubule-actin cross-linking factor 1, isoforms 1/2/3/5
KEEVL	Q9UPN3	MACF1	Microtubule-actin cross-linking factor 1, isoforms 1/2/3/5
LLHNV	Q9UPN3	MACF1	Microtubule-actin cross-linking factor 1, isoforms 1/2/3/5
SVGNT	P46821	MAP1B	Microtubule-associated protein 1B
KQEGK	P46821	MAP1B	Microtubule-associated protein 1B

QEGKS	P46821	MAP1B	Microtubule-associated protein 1B
MTSKT	P11137	MTAP2	Microtubule-associated protein 2
TDVSS	P27816	MAP4	Microtubule-associated protein 4
LVFPS	P27816	MAP4	Microtubule-associated protein 4
TNNRA	Q49MG5	MAP9	Microtubule-associated protein 9
KQLLP	Q6P0Q8	MAST2	Microtubule-associated serine/threonine-protein kinase 2
ITSLG	O60307	MAST3	Microtubule-associated serine/threonine-protein kinase 3
IINFY	Q9NU22	MDN1	Midasin
NKIKS	P20774	MIME	Mimecan precursor
LSALR	Q8N4C8	MINK1	Misshapen-like kinase 1
ARSTP	Q7Z434	MAVS	Mitochondrial antiviral-signaling protein
KQEGK	Q7L5Y1	ENOF1	Mitochondrial enolase superfamily member 1
AGSVS	Q9Y3D6	FIS1	Mitochondrial fission 1 protein
LGVGS	Q96DW6	S2538	Mitochondrial glycine transporter
YTLNN	O96008	TOM40	Mitochondrial import receptor subunit TOM40 homolog
ELQLL	O95900	TRUB2	Mitochondrial mRNA pseudouridine synthase TRUB2 precursor
GVAVS	Q8IXI1	MIRO2	Mitochondrial Rho GTPase 2
HLEGE	Q9BT17	MTG1	Mitochondrial ribosome-associated GTPase 1 precursor
VLHLE	Q9H4K7	MTG2	Mitochondrial ribosome-associated GTPase 2
NAGKS	Q9H4K7	MTG2	Mitochondrial ribosome-associated GTPase 2
FLGFL	Q6J4K2	NCLX	Mitochondrial sodium/calcium exchanger protein precursor
REFSV	Q9HC21	TPC	Mitochondrial thiamine pyrophosphate carrier
AGSVS	Q9HC21	TPC	Mitochondrial thiamine pyrophosphate carrier

KGYLS	O95140	MFN2	Mitofusin-2
IDKQL	O95140	MFN2	Mitofusin-2
KGYLS	P31152	MK04	Mitogen-activated protein kinase 4
NRLLE	Q6ZN16	M3K15	Mitogen-activated protein kinase kinase kinase 15
LGFLL	Q56UN5	M3K19	Mitogen-activated protein kinase kinase kinase 19
CSAVS	Q9Y6R4	M3K4	Mitogen-activated protein kinase kinase kinase 4
NRLLE	Q99683	M3K5	Mitogen-activated protein kinase kinase kinase 5
STPVT	O95382	M3K6	Mitogen-activated protein kinase kinase kinase 6
SLIAV	O60566	BUB1B	Mitotic checkpoint serine/threonine-protein kinase BUB1 beta
ISNIE	Q13257	MD2L1	Mitotic spindle assembly checkpoint protein MAD2A
PIINF	Q96EN8	MOCOS	Molybdenum cofactor sulfurase
GFLLG	Q8TF71	MOT10	Monocarboxylate transporter 10
SVSFF	O15374	MOT5	Monocarboxylate transporter 5
ILTAV	O15375	MOT6	Monocarboxylate transporter 6
GIIKT	O15403	MOT7	Monocarboxylate transporter 7
IIKTF	O15403	MOT7	Monocarboxylate transporter 7
LPSEI	Q15797	SMAD1	Mothers against decapentaplegic homolog 1
PSEIN	Q15797	SMAD1	Mothers against decapentaplegic homolog 1
YGVID	Q9UJG1	MSPD1	Motile sperm domain-containing protein 1
SKGYL	P30304	MPIP1	M-phase inducer phosphatase 1
AKVKL	Q99549	MPP8	M-phase phosphoprotein 8
LDKYK	Q8N594	MPND	MPN domain-containing protein
SKKRK	O43148	MCES	mRNA cap guanine-N7 methyltransferase

KKRKR	O43148	MCES	mRNA cap guanine-N7 methyltransferase
SVITS	Q8IZD4	DCP1B	mRNA-decapping enzyme 1B
RKRRF	Q96T58	MINT	Msx2-interacting protein
SISNI	Q96T58	MINT	Msx2-interacting protein
IEFQQ	Q96T58	MINT	Msx2-interacting protein
STPVT	Q96T58	MINT	Msx2-interacting protein
LSNIK	P15941	MUC1	Mucin-1 precursor
TTILT	Q8WXI7	MUC16	Mucin-16
QSTPP	Q8WXI7	MUC16	Mucin-16
ALLST	Q8WXI7	MUC16	Mucin-16
SVLTS	Q8WXI7	MUC16	Mucin-16
VIDTP	Q8WXI7	MUC16	Mucin-16
LHTSP	Q8WXI7	MUC16	Mucin-16
HTSPL	Q8WXI7	MUC16	Mucin-16
LTRTD	Q8WXI7	MUC16	Mucin-16
SLTLP	Q8WXI7	MUC16	Mucin-16
DVSSS	Q8WXI7	MUC16	Mucin-16
TSLGA	Q8WXI7	MUC16	Mucin-16
STPVT	Q8WXI7	MUC16	Mucin-16
STPPT	Q685J3	MUC17	Mucin-17 precursor
RSTPV	Q685J3	MUC17	Mucin-17 precursor
STPVT	Q685J3	MUC17	Mucin-17 precursor
AGVTT	Q7Z5P9	MUC19	Mucin-19 precursor

GVTTP	Q7Z5P9	MUC19	Mucin-19 precursor
FDASI	Q02817	MUC2	Mucin-2 precursor
SNGVS	Q5SSG8	MUC21	Mucin-21 precursor
SISQV	Q02505	MUC3A	Mucin-3A precursor
STPVT	Q02505	MUC3A	Mucin-3A precursor
ARSTP	Q9H195	MUC3B	Mucin-3B precursor (Fragments)
LLGVG	Q99102	MUC4	Mucin-4 precursor
MTSKT	Q99102	MUC4	Mucin-4 precursor
TDVSS	Q99102	MUC4	Mucin-4 precursor
STPVT	Q99102	MUC4	Mucin-4 precursor
TTPVS	P98088	MUC5A	Mucin-5AC precursor
SELLS	P98088	MUC5A	Mucin-5AC precursor
STPVT	P98088	MUC5A	Mucin-5AC precursor
PQAET	Q9HC84	MUC5B	Mucin-5B precursor
VSSSV	Q9HC84	MUC5B	Mucin-5B precursor
TTPVS	Q6W4X9	MUC6	Mucin-6 precursor
TPVST	Q6W4X9	MUC6	Mucin-6 precursor
STTNI	Q6W4X9	MUC6	Mucin-6 precursor
FIRKS	Q8TAX7	MUC7	Mucin-7 precursor
VSVGN	Q8TDD5	MCLN3	Mucolipin-3
TSLGA	Q13477	MADCA	Mucosal addressin cell adhesion molecule 1 precursor
VLHLE	P03971	MIS	Muellerian-inhibiting factor precursor
LGFLL	P33527	MRP1	Multidrug resistance-associated protein 1

IVILL	P33527	MRP1	Multidrug resistance-associated protein 1
QIVRQ	O60568	PLOD3	Multifunctional procollagen lysine hydroxylase and glycosyltransferase LH3 precursor
LLGVG	Q7Z7M0	MEGF8	Multiple epidermal growth factor-like domains protein 8 precursor
SIIKE	O75970	MPDZ	Multiple PDZ domain protein
SLTLP	Q9H7P6	MB12B	Multivesicular body subunit 12B
AVVSL	P08912	ACM5	Muscarinic acetylcholine receptor M5
TLPSE	O15146	MUSK	Muscle, skeletal receptor tyrosine-protein kinase precursor
SKKRK	Q9BQG0	MBB1A	Myb-binding protein 1A
NGTDA	O60487	MPZL2	Myelin protein zero-like protein 2 precursor
IIIVI	O60487	MPZL2	Myelin protein zero-like protein 2 precursor
DASIS	Q6UWV2	MPZL3	Myelin protein zero-like protein 3 precursor
SKKRK	Q9Y2G1	MYRF	Myelin regulatory factor
AGVTT	P20138	CD33	Myeloid cell surface antigen CD33 precursor
TDVSS	P58340	MLF1	Myeloid leukemia factor 1
VSLSN	Q14814	MEF2D	Myocyte-specific enhancer factor 2D
LVFPS	Q5VU43	MYOME	Myomegalin
NQSLA	Q5VU43	MYOME	Myomegalin
HLEGE	P54296	MYOM2	Myomesin-2
CTASN	Q86TC9	MYPN	Myopalladin
TASNK	Q86TC9	MYPN	Myopalladin
EFDAS	P08590	MYL3	Myosin light chain 3
KSDEL	P14649	MYL6B	Myosin light chain 6B

QSYSI	Q15746	MYLK	Myosin light chain kinase, smooth muscle
CTASN	Q15746	MYLK	Myosin light chain kinase, smooth muscle
LEGEV	P12882	MYH1	Myosin-1
LHLEG	P35580	MYH10	Myosin-10
KSALL	P35580	MYH10	Myosin-10
NSELL	Q7Z406	MYH14	Myosin-14
LEGEV	Q9UKX2	MYH2	Myosin-2
KLMSN	P11055	MYH3	Myosin-3
AKVKL	P12883	MYH7	Myosin-7
LEGEV	P13535	MYH8	Myosin-8
DVSSS	Q13203	MYBPH	Myosin-binding protein H
VTLSK	Q8WXR4	MYO3B	Myosin-IIIb
TLSKD	Q8WXR4	MYO3B	Myosin-IIIb
LSKDQ	Q8WXR4	MYO3B	Myosin-IIIb
AGVTT	Q9UBF9	MYOTI	Myotilin
KSALL	Q13615	MTMR3	Myotubularin-related protein 3
NAKKT	O95248	MTMR5	Myotubularin-related protein 5
AVVSL	Q9Y217	MTMR6	Myotubularin-related protein 6
ITSLG	Q96EF0	MTMR8	Myotubularin-related protein 8
KKRKR	Q8N3J2	METL4	N(6)-adenine-specific methyltransferase METTL4
SLTLP	O95865	DDAH2	N(G),N(G)-dimethylarginine dimethylaminohydrolase 2
NGVSV	Q86UT5	NHRF4	Na(+)/H(+) exchange regulatory cofactor NHE-RF4
SGVAV	Q9Y3Q0	NALD2	N-acetylated-alpha-linked acidic dipeptidase 2

KSALL	Q3T906	GNPTA	N-acetylglucosamine-1-phosphotransferase subunits alpha/beta precursor
LHLEG	Q9Y303	NAGA	N-acetylglucosamine-6-phosphate deacetylase
NRLLE	Q9Y2A9	B3GN3	N-acetyllactosaminide beta-1,3-N-acetylglucosaminyltransferase 3
TCKVQ	Q9Y2A9	B3GN3	N-acetyllactosaminide beta-1,3-N-acetylglucosaminyltransferase 3
FLLGV	Q9C0J1	B3GN4	N-acetyllactosaminide beta-1,3-N-acetylglucosaminyltransferase 4
LGFLL	Q96PB1	CASD1	N-acetylneuraminate 9-O-acetyltransferase
ASNKN	Q5FWF5	ESCO1	N-acetyltransferase ESCO1
LILKA	Q86W25	NAL13	NACHT, LRR and PYD domains-containing protein 13
GLLLY	Q86W24	NAL14	NACHT, LRR and PYD domains-containing protein 14
IKQEL	Q9NX02	NALP2	NACHT, LRR and PYD domains-containing protein 2
CLTRT	Q9NX02	NALP2	NACHT, LRR and PYD domains-containing protein 2
IKQEL	Q8WX94	NALP7	NACHT, LRR and PYD domains-containing protein 7
CLTRT	Q8WX94	NALP7	NACHT, LRR and PYD domains-containing protein 7
GFLLG	Q4G0N4	NAKD2	NAD kinase 2, mitochondrial precursor
SKVLD	Q4G0N4	NAKD2	NAD kinase 2, mitochondrial precursor
VTELQ	Q9NRC8	SIR7	NAD-dependent protein deacetylase sirtuin-7
RKSDE	O75438	NDUB1	NADH dehydrogenase [ubiquinone] 1 beta subcomplex subunit 1
LTNSE	P28331	NDUS1	NADH-ubiquinone oxidoreductase 75 kDa subunit, mitochondrial precursor
MSNNV	P03891	NU2M	NADH-ubiquinone oxidoreductase chain 2
SPLCT	P03915	NU5M	NADH-ubiquinone oxidoreductase chain 5
VSKVL	Q6N069	NAA16	N-alpha-acetyltransferase 16, NatA auxiliary subunit

LEGEV	Q14CX7	NAA25	N-alpha-acetyltransferase 25, NatB auxiliary subunit
PQAET	Q13765	NACA	Nascent polypeptide-associated complex subunit alpha
ISNIE	E9PAV3	NACAM	Nascent polypeptide-associated complex subunit alpha, muscle-specific form
PQAET	E9PAV3	NACAM	Nascent polypeptide-associated complex subunit alpha, muscle-specific form
GAIVS	Q68D85	NR3L1	Natural cytotoxicity triggering receptor 3 ligand 1 precursor
VIIVI	Q9BZW8	CD244	Natural killer cell receptor 2B4 precursor
IIVIL	Q9BZW8	CD244	Natural killer cell receptor 2B4 precursor
GSAIA	P49281	NRAM2	Natural resistance-associated macrophage protein 2
VGLLL	P49281	NRAM2	Natural resistance-associated macrophage protein 2
LLSLI	Q9Y2A7	NCKP1	Nck-associated protein 1
YTSVI	P55160	NCKPL	Nck-associated protein 1-like
LLSLI	P55160	NCKPL	Nck-associated protein 1-like
KANAI	P20929	NEBU	Nebulin
VSKVL	P20929	NEBU	Nebulin
ITEEF	Q99608	NECD	Necdin
GVSVL	Q15223	NECT1	Nectin-1 precursor
PVTLS	Q92692	NECT2	Nectin-2 precursor
LGFLL	Q9BT67	NFIP1	NEDD4 family-interacting protein 1
VRQQS	Q9H0M0	WWP1	NEDD4-like E3 ubiquitin-protein ligase WWP1
SAIAS	Q8WX92	NELFB	Negative elongation factor B
GNTLY	P18615	NELFE	Negative elongation factor E

NAITT	Q92859	NEO1	Neogenin precursor
KYKNA	Q92859	NEO1	Neogenin precursor
NVTLS	O60500	NPHN	Nephrin precursor
LTLPS	Q7Z494	NPHP3	Nephrocystin-3
IIIVI	Q7Z494	NPHP3	Nephrocystin-3
TTILT	Q8NF91	SYNE1	Nesprin-1
SAVSK	Q8NF91	SYNE1	Nesprin-1
AVSKV	Q8NF91	SYNE1	Nesprin-1
VSKVL	Q8NF91	SYNE1	Nesprin-1
DVSSS	Q8NF91	SYNE1	Nesprin-1
LLSLI	Q6ZMZ3	SYNE3	Nesprin-3
LLHNV	Q6ZMZ3	SYNE3	Nesprin-3
NQSLA	O95185	UNC5C	Netrin receptor UNC5C precursor
LYCKA	O95185	UNC5C	Netrin receptor UNC5C precursor
ISNIE	Q96CW9	NTNG2	Netrin-G2 precursor
CTASN	P13591	NCAM1	Neural cell adhesion molecule 1 precursor
YVVQL	P32004	L1CAM	Neural cell adhesion molecule L1 precursor
VVQLP	P32004	L1CAM	Neural cell adhesion molecule L1 precursor
CTASN	O00533	NCHL1	Neural cell adhesion molecule L1-like protein precursor
STPPT	O00401	WASL	Neural Wiskott-Aldrich syndrome protein
LGVGS	Q96JN8	NEUL4	Neuralized-like protein 4
PVTLS	Q8IZ57	NRSN1	Neurensin-1
NAKKT	Q9ULB1	NRX1A	Neurexin-1 precursor

GLLLY	Q9Y4C0	NRX3A	Neurexin-3 precursor
TAVTF	Q8NFP9	NBEA	Neurobeachin
SELLS	Q8NFP9	NBEA	Neurobeachin
NRVFC	Q8NFP9	NBEA	Neurobeachin
LIAVG	Q8NFP9	NBEA	Neurobeachin
TTPVS	Q6ZNJ1	NBEL2	Neurobeachin-like protein 2
DAKVK	Q09666	AHNK	Neuroblast differentiation-associated protein AHNAK
ELLSL	A2RRP1	NBAS	Neuroblastoma-amplified sequence
VLTSK	P16519	NEC2	Neuroendocrine convertase 2 precursor
TFSNG	P16519	NEC2	Neuroendocrine convertase 2 precursor
KRKRR	Q7Z628	ARHG8	Neuroepithelial cell-transforming gene 1 protein
VVSLS	Q7Z628	ARHG8	Neuroepithelial cell-transforming gene 1 protein
IASGV	O94856	NFASC	Neurofascin precursor
AVVSL	P21359	NF1	Neurofibromin
KQLLP	P21359	NF1	Neurofibromin
SQVNE	Q04721	NOTC2	Neurogenic locus notch homolog protein 2 precursor
RARRE	Q9UM47	NOTC3	Neurogenic locus notch homolog protein 3 precursor
ARREL	Q9UM47	NOTC3	Neurogenic locus notch homolog protein 3 precursor
SSSVI	Q8NEJ9	NGDN	Neuroguidin
LPSEI	Q9NZ94	NLGN3	Neuroligin-3 precursor
LPSEI	Q8NFZ3	NLGNY	Neuroligin-4, Y-linked precursor
IIVII	P29371	NK3R	Neuromedin-K receptor
FLLGV	Q9HB89	NMUR1	Neuromedin-U receptor 1

LLGVG	Q9HB89	NMUR1	Neuromedin-U receptor 1
SAVSK	Q8IVL1	NAV2	Neuron navigator 2
VDIFN	Q9UGM1	ACHA9	Neuronal acetylcholine receptor subunit alpha-9 precursor
TNVTL	Q7Z3B1	NEGR1	Neuronal growth regulator 1 precursor
LTLPS	Q8IUM7	NPAS4	Neuronal PAS domain-containing protein 4
MELLI	O95502	NPTXR	Neuronal pentraxin receptor
VIDTP	Q9UH03	SEPT3	Neuronal-specific septin-3
VGNTL	Q9GZQ6	NPFF1	Neuropeptide FF receptor 1
RARRE	Q9Y5X5	NPFF2	Neuropeptide FF receptor 2
VLDLK	P49146	NPY2R	Neuropeptide Y receptor type 2
SALLS	Q15761	NPY5R	Neuropeptide Y receptor type 5
VITSL	P48146	NPBW2	Neuropeptides B/W receptor type 2
SVLTS	O14786	NRP1	Neuropilin-1 precursor
VLTSK	O14786	NRP1	Neuropilin-1 precursor
GLLLY	O60462	NRP2	Neuropilin-2 precursor
LLLYC	O60462	NRP2	Neuropilin-2 precursor
SNGVS	Q9Y639	NPTN	Neuroplastin precursor
NGVSV	Q9Y639	NPTN	Neuroplastin precursor
GVSVL	Q9Y639	NPTN	Neuroplastin precursor
PVTLS	Q14697	GANAB	Neutral alpha-glucosidase AB precursor
SKKRK	Q8TET4	GANC	Neutral alpha-glucosidase C
VLTSK	Q8TET4	GANC	Neutral alpha-glucosidase C
VNEKI	Q6GTS8	P20D1	N-fatty-acyl-amino acid synthase/hydrolase PM20D1 precursor

TYMLT	O15226	NKRF	NF-kappa-B-repressing factor
LGVGS	Q9P2E3	ZNFX1	NFX1-type zinc finger-containing protein 1
QEGKS	Q9P2E3	ZNFX1	NFX1-type zinc finger-containing protein 1
ILLSL	Q9P2E3	ZNFX1	NFX1-type zinc finger-containing protein 1
GVSVL	Q969V3	NCLN	Nicalin precursor
TSVIT	P14543	NID1	Nidogen-1 precursor
SVITS	P14543	NID1	Nidogen-1 precursor
DELLH	Q7Z2Y5	NRK	Nik-related protein kinase
CKVQS	Q8N4C6	NIN	Ninein
LGVGS	Q9Y2I6	NINL	Ninein-like protein
KDQLS	Q9Y2I6	NINL	Ninein-like protein
NAGKS	Q8NC60	NOA1	Nitric oxide-associated protein 1
AGKST	Q8NC60	NOA1	Nitric oxide-associated protein 1
GKSLY	Q9NPP4	NLRC4	NLR family CARD domain-containing protein 4
QSTCS	O95897	NOE2	Noelin-2 precursor
NRLLE	Q0D2I5	IFFO1	Non-homologous end joining factor IFFO1
LMSNN	Q0D2I5	IFFO1	Non-homologous end joining factor IFFO1
VSLSN	Q9HCG7	GBA2	Non-lysosomal glucosylceramidase
LQLLM	Q8WV22	NSE1	Non-structural maintenance of chromosomes element 1 homolog
ALRTG	Q9NVX2	NLE1	Notchless protein homolog 1
STCSA	Q7Z6K4	NRARP	Notch-regulated ankyrin repeat-containing protein
FSVNA	O15118	NPC1	NPC intracellular cholesterol transporter 1 precursor
SLGAI	O15118	NPC1	NPC intracellular cholesterol transporter 1 precursor

NIAFS	O15118	NPC1	NPC intracellular cholesterol transporter 1 precursor
IAVGL	Q16288	NTRK3	NT-3 growth factor receptor precursor
RRELP	Q69YI7	NAIF1	Nuclear apoptosis-inducing factor 1
RKRRF	P23497	SP100	Nuclear autoantigen Sp-100
KRKRR	Q13342	SP140	Nuclear body protein SP140
KRKRR	Q9H930	SP14L	Nuclear body protein SP140-like protein
VGLLL	Q96HA1	P121A	Nuclear envelope pore membrane protein POM 121
GLLLY	Q96HA1	P121A	Nuclear envelope pore membrane protein POM 121
VGLLL	A8CG34	P121C	Nuclear envelope pore membrane protein POM 121C
GLLLY	A8CG34	P121C	Nuclear envelope pore membrane protein POM 121C
ELLSL	Q16649	NFIL3	Nuclear factor interleukin-3-regulated protein
VVSLS	Q6P4R8	NFRKB	Nuclear factor related to kappa-B-binding protein
SALLS	Q14980	NUMA1	Nuclear mitotic apparatus protein 1
KQLLP	P57740	NU107	Nuclear pore complex protein Nup107
KGYLS	Q12769	NU160	Nuclear pore complex protein Nup160
SSVIT	Q12769	NU160	Nuclear pore complex protein Nup160
TVSVG	Q8TEM1	PO210	Nuclear pore membrane glycoprotein 210 precursor
TSVIT	Q9NZP6	NPAP1	Nuclear pore-associated protein 1
SVITS	Q9NZP6	NPAP1	Nuclear pore-associated protein 1
KVKLI	Q8NI08	NCOA7	Nuclear receptor coactivator 7
KIMTS	Q8NI08	NCOA7	Nuclear receptor coactivator 7
TSVIT	O75376	NCOR1	Nuclear receptor corepressor 1
STPPT	O75376	NCOR1	Nuclear receptor corepressor 1

HLEGE	Q9Y618	NCOR2	Nuclear receptor corepressor 2
NITEE	P49116	NR2C2	Nuclear receptor subfamily 2 group C member 2
ELLIL	O00482	NR5A2	Nuclear receptor subfamily 5 group A member 2
IKENK	Q9UHY1	NRBP	Nuclear receptor-binding protein
TTPVS	P48552	NRIP1	Nuclear receptor-interacting protein 1
TPVST	P48552	NRIP1	Nuclear receptor-interacting protein 1
KKRKR	Q16656	NRF1	Nuclear respiratory factor 1
ELLSL	Q9UBU9	NXF1	Nuclear RNA export factor 1
ELLSL	Q9GZY0	NXF2	Nuclear RNA export factor 2
ELLSL	Q9H1B4	NXF5	Nuclear RNA export factor 5
SKDQL	P80303	NUCB2	Nucleobindin-2 precursor
KAVVS	Q14978	NOLC1	Nucleolar and coiled-body phosphoprotein 1
PQAET	Q9Y3T9	NOC2L	Nucleolar complex protein 2 homolog
KDQLS	Q9Y3T9	NOC2L	Nucleolar complex protein 2 homolog
SIIKE	Q13823	NOG2	Nucleolar GTP-binding protein 2
IIKEE	Q13823	NOG2	Nucleolar GTP-binding protein 2
LSLIN	O60287	NPA1P	Nucleolar pre-ribosomal-associated protein 1
INFYD	O60287	NPA1P	Nucleolar pre-ribosomal-associated protein 1
KAVVS	Q9H8H0	NOL11	Nucleolar protein 11
IETVI	Q9H8H0	NOL11	Nucleolar protein 11
NSELL	P78316	NOP14	Nucleolar protein 14
SDEFD	O94818	NOL4	Nucleolar protein 4
LLGVG	O00567	NOP56	Nucleolar protein 56

VVSLS	O00567	NOP56	Nucleolar protein 56
SKKRK	Q9Y2X3	NOP58	Nucleolar protein 58
KRKRR	Q9UMY1	NOL7	Nucleolar protein 7
LIKQE	Q7Z3B4	NUP54	Nucleoporin p54
GEVNK	Q9H1M0	N62CL	Nucleoporin-62 C-terminal-like protein
TELQL	P12270	TPR	Nucleoprotein TPR
TCSAV	Q12830	BPTF	Nucleosome-remodeling factor subunit BPTF
KKRKR	Q12830	BPTF	Nucleosome-remodeling factor subunit BPTF
CTASN	Q5VST9	OBSCN	Obscurin
TDVSS	O75147	OBSL1	Obscurin-like protein 1
STCSA	Q9H209	O10A4	Olfactory receptor 10A4
IIIVI	Q8NH74	O10A6	Olfactory receptor 10A6
GFLLG	Q8NH81	O10G6	Olfactory receptor 10G6
IETVI	Q8NGN5	O10G8	Olfactory receptor 10G8
LVFPS	Q8NGN5	O10G8	Olfactory receptor 10G8
LLSLI	Q8NHC4	O10J5	Olfactory receptor 10J5
YVSNK	Q8NGX6	O10R2	Olfactory receptor 10R2
ELQLL	Q8NGX3	O10T2	Olfactory receptor 10T2
LGFLL	Q8NGI7	O10V1	Olfactory receptor 10V1
ELQLL	Q8NGY1	O10Z1	Olfactory receptor 10Z1
AVVSL	Q8NGC1	O11G2	Olfactory receptor 11G2
AVVSL	Q8NG94	O11H1	Olfactory receptor 11H1
AVVSL	B2RN74	O11HC	Olfactory receptor 11H12

AVVSL	Q8NH07	O11H2	Olfactory receptor 11H2
STCSA	Q8NGS9	O13C2	Olfactory receptor 13C2
VVQLP	Q8NGS9	O13C2	Olfactory receptor 13C2
STCSA	Q8NGS6	O13C3	Olfactory receptor 13C3
STCSA	Q8NGS5	O13C4	Olfactory receptor 13C4
STCSA	Q8NGS8	O13C5	Olfactory receptor 13C5
VVQLP	Q8NGS8	O13C5	Olfactory receptor 13C5
STCSA	P0DN81	O13C7	Olfactory receptor 13C7
STCSA	Q8NGS7	O13C8	Olfactory receptor 13C8
STCSA	Q8NGT0	O13C9	Olfactory receptor 13C9
VVQLP	Q8NGT0	O13C9	Olfactory receptor 13C9
STCSA	Q8NGV5	O13D1	Olfactory receptor 13D1
IMITT	Q8NHC5	O14AG	Olfactory receptor 14A16
IKSAL	Q8NGZ2	O14K1	Olfactory receptor 14K1
VVSLS	O60431	OR1I1	Olfactory receptor 1I1
AVVSL	Q8NGR3	OR1K1	Olfactory receptor 1K1
NKIKS	Q8NH93	OR1L3	Olfactory receptor 1L3
SCSIS	Q8NGR5	OR1L4	Olfactory receptor 1L4
SCSIS	Q8NGR2	OR1L6	Olfactory receptor 1L6
LGFLL	Q8NGT9	OR2A1	Olfactory receptor 2A1/2A42
GFLLG	Q8NGT9	OR2A1	Olfactory receptor 2A1/2A42
LLSLI	O95047	OR2A4	Olfactory receptor 2A4
LLSLI	Q96R45	OR2A7	Olfactory receptor 2A7

VITIE	Q9GZK3	OR2B2	Olfactory receptor 2B2
AVVSL	Q8N628	OR2C3	Olfactory receptor 2C3
VQLPL	Q5TZ20	OR2G6	Olfactory receptor 2G6
VGNTL	Q9GZK4	OR2H1	Olfactory receptor 2H1
VGNTL	O95918	OR2H2	Olfactory receptor 2H2
ITTIL	Q9NQN1	OR2S1	Olfactory receptor 2S2
STCSA	Q9NQN1	OR2S1	Olfactory receptor 2S2
LTAVT	Q8NHB1	OR2V1	Olfactory receptor 2V1
LTAVT	Q96R30	OR2V2	Olfactory receptor 2V2
LSLIA	Q8NG97	OR2Z1	Olfactory receptor 2Z1
GVSVL	Q8NH83	OR4A5	Olfactory receptor 4A5
FLGFL	Q8NGM1	OR4CF	Olfactory receptor 4C15
SSVIT	Q8NGM1	OR4CF	Olfactory receptor 4C15
VILLS	Q8NGM1	OR4CF	Olfactory receptor 4C15
ILLSL	Q8NGM1	OR4CF	Olfactory receptor 4C15
ILTAV	Q8NGL9	OR4CG	Olfactory receptor 4C16
QNITE	Q8NH37	OR4C3	Olfactory receptor 4C3
STCSA	Q8NGC2	OR4E2	Olfactory receptor 4E2
IIVII	Q8NGD4	OR4K1	Olfactory receptor 4K1
STCSA	Q8NGD5	OR4KE	Olfactory receptor 4K14
SISQV	Q8NGD5	OR4KE	Olfactory receptor 4K14
KARST	Q8NH41	OR4KF	Olfactory receptor 4K15
KARST	Q8NGC6	OR4KH	Olfactory receptor 4K17

STCSA	Q96R72	OR4K3	Olfactory receptor 4K3
TIIIV	Q8NGL7	OR4P4	Olfactory receptor 4P4
VVQLP	Q8NH73	OR4S2	Olfactory receptor 4S2
LLILK	Q8TCB6	O51E1	Olfactory receptor 51E1
TTILT	A6NGY5	O51F1	Olfactory receptor 51F1
IDTPC	A6NGY5	O51F1	Olfactory receptor 51F1
TTILT	Q8NH61	O51F2	Olfactory receptor 51F2
ALLST	Q8NH61	O51F2	Olfactory receptor 51F2
TTILT	Q8NH59	O51Q1	Olfactory receptor 51Q1
SISNI	Q9UKL2	O52A1	Olfactory receptor 52A1
TTILT	Q8NGK2	O52B4	Olfactory receptor 52B4
ALLST	Q8NGF0	O52B6	Olfactory receptor 52B6
LTSKV	Q8NGF0	O52B6	Olfactory receptor 52B6
IVIIV	Q8NH55	O52E5	Olfactory receptor 52E5
TTILT	Q8NGJ2	O52H1	Olfactory receptor 52H1
LILKA	Q8NH67	O52I2	Olfactory receptor 52I2
AVVSL	Q8NH53	O52N1	Olfactory receptor 52N1
VVSLS	Q8NH53	O52N1	Olfactory receptor 52N1
AVVSL	Q8NGI2	O52N4	Olfactory receptor 52N4
VVSLS	Q8NGI2	O52N4	Olfactory receptor 52N4
LILKA	Q8NH56	O52N5	Olfactory receptor 52N5
ALLST	P0C646	O52Z1	Olfactory receptor 52Z1
LILKA	Q8NGI3	O56B1	Olfactory receptor 56B1

YLSAL	Q8NGI3	O56B1	Olfactory receptor 56B1
LTAVT	Q8NGI9	OR5A2	Olfactory receptor 5A2
LSLIA	Q8NGI9	OR5A2	Olfactory receptor 5A2
STCSA	P0C628	O5AC1	Olfactory receptor 5AC1
LTAVT	Q8NH90	O5AK2	Olfactory receptor 5AK2
LTAVT	Q8NH18	OR5J2	Olfactory receptor 5J2
VGLLL	A6NMS3	OR5K4	Olfactory receptor 5K4
LTAVT	Q8NGP6	OR5M8	Olfactory receptor 5M8
LTAVT	Q8WZ94	OR5P3	Olfactory receptor 5P3
RVFCD	Q8NGG2	OR5T2	Olfactory receptor 5T2
SSVIT	Q8NGG2	OR5T2	Olfactory receptor 5T2
ALLST	Q9UGF6	OR5V1	Olfactory receptor 5V1
GVSVL	Q9NZP2	OR6C2	Olfactory receptor 6C2
VSVLT	Q9NZP2	OR6C2	Olfactory receptor 6C2
GVSVL	A6NDL8	O6C68	Olfactory receptor 6C68
ALLST	A6NCV1	O6C74	Olfactory receptor 6C74
VTLSK	A6NL08	O6C75	Olfactory receptor 6C75
IIIVI	Q8NGW6	OR6K6	Olfactory receptor 6K6
IIVIL	Q8NGW6	OR6K6	Olfactory receptor 6K6
WYTSV	Q8NGQ2	OR6Q1	Olfactory receptor 6Q1
TIIIV	Q8N148	OR6V1	Olfactory receptor 6V1
IMTSK	Q8NH79	OR6X1	Olfactory receptor 6X1
ILLSL	Q8NG99	OR7G2	Olfactory receptor 7G2

SSVIT	Q8NGG7	OR8A1	Olfactory receptor 8A1
SSVIT	Q15620	OR8B8	Olfactory receptor 8B8
FLGFL	Q8WZ84	OR8D1	Olfactory receptor 8D1
SSVIT	Q8WZ84	OR8D1	Olfactory receptor 8D1
SSVIT	Q9GZM6	OR8D2	Olfactory receptor 8D2
SSVIT	Q8NGM9	OR8D4	Olfactory receptor 8D4
ELLIL	Q8NG78	OR8G5	Olfactory receptor 8G5
INLCN	Q8NGG1	OR8J2	Olfactory receptor 8J2
LTAVT	Q8NH09	OR8S1	Olfactory receptor 8S1
STCSA	Q8NH09	OR8S1	Olfactory receptor 8S1
SSVIT	Q8NH10	OR8U1	Olfactory receptor 8U1
SSVIT	P0C7N1	OR8U8	Olfactory receptor 8U8
SSVIT	P0C7N5	OR8U9	Olfactory receptor 8U9
VSVLT	Q8NGT5	OR9A2	Olfactory receptor 9A2
VIIVI	Q8NGT5	OR9A2	Olfactory receptor 9A2
SKGYL	Q8NH87	OR9G1	Olfactory receptor 9G1
WYTSV	Q8NGQ1	OR9G4	Olfactory receptor 9G4
YTSVI	Q8NGQ6	OR9I1	Olfactory receptor 9I1
TSVIT	Q8NGQ6	OR9I1	Olfactory receptor 9I1
IIVII	Q8NGE7	OR9K2	Olfactory receptor 9K2
IVIIV	Q8NGE7	OR9K2	Olfactory receptor 9K2
MLTNS	Q13516	OLIG2	Oligodendrocyte transcription factor 2
TLSKD	P23515	OMGP	Oligodendrocyte-myelin glycoprotein precursor

VSKVL	Q99650	OSMR	Oncostatin-M-specific receptor subunit beta precursor
DPLVF	A6NGQ2	OOEP	Oocyte-expressed protein homolog
TPVST	Q86WS3	OOSP2	Oocyte-secreted protein 2 precursor
RRELP	Q9NZT2	OGFR	Opioid growth factor receptor
SKKRK	Q9NZT2	OGFR	Opioid growth factor receptor
LLGVG	Q9H1Y3	OPN3	Opsin-3
NVTLS	Q5W0B1	OBI1	ORC ubiquitin ligase 1
SLTLP	Q5W0B1	OBI1	ORC ubiquitin ligase 1
VSVLT	O43614	OX2R	Orexin receptor type 2
VGNTL	O43613	OX1R	Orexin/Hypocretin receptor type 1
LQLLM	Q86UW1	OSTA	Organic solute transporter subunit alpha
LSKKR	Q9UBD5	ORC3	Origin recognition complex subunit 3
SKKRK	Q9UBD5	ORC3	Origin recognition complex subunit 3
QEGKS	Q9UMX2	OAZ3	Ornithine decarboxylase antizyme 3
NKIKS	Q99983	OMD	Osteomodulin precursor
SLTLP	Q3ZCN5	OTOGL	Otogelin-like protein precursor
VSLSN	Q01804	OTUD4	OTU domain-containing protein 4
KTDVS	Q01804	OTUD4	OTU domain-containing protein 4
FSNGC	Q5T2S8	ODAD2	Outer dynein arm-docking complex subunit 2
TSPLC	A5D8V7	ODAD3	Outer dynein arm-docking complex subunit 3
TDVSS	Q9BZK8	OCR1	Ovarian cancer-related protein 1
IETVI	Q7RTZ1	OVCH2	Ovochymase-2 precursor
TLSKK	Q8N573	OXR1	Oxidation resistance protein 1

DQLSG	Q8N573	OXR1	Oxidation resistance protein 1
ARREL	P36639	8ODP	Oxidized purine nucleoside triphosphate hydrolase
MELLI	P36551	HEM6	Oxygen-dependent coproporphyrinogen-III oxidase, mitochondrial precursor
ELLIL	P36551	HEM6	Oxygen-dependent coproporphyrinogen-III oxidase, mitochondrial precursor
GAIVS	Q9BXB5	OSB10	Oxysterol-binding protein-related protein 10
EEVLA	Q9H0X9	OSBL5	Oxysterol-binding protein-related protein 5
SLSNG	Q96SU4	OSBL9	Oxysterol-binding protein-related protein 9
SNGVS	Q99571	P2RX4	P2X purinoceptor 4
NGTDA	P47900	P2RY1	P2Y purinoceptor 1
ASGVA	Q96G91	P2Y11	P2Y purinoceptor 11
EEVLA	Q9NUG6	PDRG1	p53 and DNA damage-regulated protein 1
VQLPL	Q9HB75	PIDD1	p53-induced death domain-containing protein 1
AVGLL	Q9HB75	PIDD1	p53-induced death domain-containing protein 1
VNKIK	Q96ST3	SIN3A	Paired amphipathic helix protein Sin3a
VNKIK	O75182	SIN3B	Paired amphipathic helix protein Sin3b
RARRE	P15863	PAX1	Paired box protein Pax-1
TTPVS	P26367	PAX6	Paired box protein Pax-6
PVTLS	Q9ULE6	PALD	Paladin
SALLS	Q8WX93	PALLD	Palladin
RSTPV	Q8WX93	PALLD	Palladin
NLCNV	Q8WTX9	ZDHC1	Palmitoyltransferase ZDHHC1

LCNVD	Q8WTX9	ZDHC1	Palmitoyltransferase ZDHHC1
FLLGV	Q8IYP9	ZDH23	Palmitoyltransferase ZDHHC23
SALRT	Q58A45	PAN3	PAN2-PAN3 deadenylation complex subunit PAN3
LIAVG	Q96RD7	PANX1	Pannexin-1
IAVGL	Q96RD7	PANX1	Pannexin-1
VNEKI	Q9H999	PANK3	Pantothenate kinase 3
NEKIN	Q9H999	PANK3	Pantothenate kinase 3
PVTLS	Q13219	PAPP1	Pappalysin-1 precursor
VIEFQ	A0A0J9YXQ4	PMA6E	Paraneoplastic antigen Ma6E
TDVSS	Q3KNS1	PTHD3	Patched domain-containing protein 3
DVSSS	Q3KNS1	PTHD3	Patched domain-containing protein 3
VILLS	Q6ZW05	PTHD4	Patched domain-containing protein 4
PLYGV	P49023	PAXI	Paxillin
GFLLG	Q6ZW49	PAXI1	PAX-interacting protein 1
DKQLL	Q6ZW49	PAXI1	PAX-interacting protein 1
AVSKV	Q96HC4	PDLI5	PDZ and LIM domain protein 5
CKARS	O15018	PDZD2	PDZ domain-containing protein 2
VTLSK	Q9H5P4	PDZD7	PDZ domain-containing protein 7
LSNGV	Q9H5P4	PDZD7	PDZ domain-containing protein 7
KLIKQ	Q8NEN9	PDZD8	PDZ domain-containing protein 8
ELQLL	Q6ZMN7	PZRN4	PDZ domain-containing RING finger protein 4
SSSVI	Q96RV3	PCX1	Pecanex-like protein 1
LTLPS	Q9H6A9	PCX3	Pecanex-like protein 3

SLGAI	Q9H6A9	PCX3	Pecanex-like protein 3
EFQQK	Q8WV60	PTCD2	Pentatricopeptide repeat-containing protein 2, mitochondrial
EEVLA	Q8WV60	PTCD2	Pentatricopeptide repeat-containing protein 2, mitochondrial
PPTNN	O43692	PI15	Peptidase inhibitor 15 precursor
LVFPS	P19021	AMD	Peptidyl-glycine alpha-amidating monooxygenase precursor
DKYKN	Q13427	PPIG	Peptidyl-prolyl cis-trans isomerase G
SKKRK	Q13427	PPIG	Peptidyl-prolyl cis-trans isomerase G
ALRTG	Q13526	PIN1	Peptidyl-prolyl cis-trans isomerase NIMA-interacting 1
KAVVS	Q96BP3	PPWD1	Peptidylprolyl isomerase domain and WD repeat-containing protein 1
KLHTS	Q96BP3	PPWD1	Peptidylprolyl isomerase domain and WD repeat-containing protein 1
LHTSP	Q96BP3	PPWD1	Peptidylprolyl isomerase domain and WD repeat-containing protein 1
HTSPL	Q96BP3	PPWD1	Peptidylprolyl isomerase domain and WD repeat-containing protein 1
SLTLP	Q9BXM0	PRAX	Periaxin
LTLPS	Q9BXM0	PRAX	Periaxin
ELQLL	O95613	PCNT	Pericentrin
LNNAK	O95613	PCNT	Pericentrin
SALLS	O95613	PCNT	Pericentrin
VLDLK	O95613	PCNT	Pericentrin
ELQLL	Q15154	PCM1	Pericentriolar material 1 protein

TMNSL	Q00G26	PLIN5	Perilipin-5
VNKIK	P56645	PER3	Period circadian protein homolog 3
GEPII	Q15063	POSTN	Periostin precursor
LSKKR	O95153	RIMB1	Peripheral-type benzodiazepine receptor-associated protein 1
SKKRK	O95153	RIMB1	Peripheral-type benzodiazepine receptor-associated protein 1
SKVLD	Q8NEY8	PPHLN	Periphilin-1
NVNAG	A1KZ92	PXDNL	Peroxidasin-like protein precursor
LSLIA	A1KZ92	PXDNL	Peroxidasin-like protein precursor
IAFSN	P32119	PRDX2	Peroxiredoxin-2
EFSVN	O15254	ACOX3	Peroxisomal acyl-coenzyme A oxidase 3
SGVAV	Q2T9J0	TYSD1	Peroxisomal leader peptide-processing protease
AVVSL	Q2T9J0	TYSD1	Peroxisomal leader peptide-processing protease
IIKEE	O43808	PM34	Peroxisomal membrane protein PMP34
LGAIV	P51659	DHB4	Peroxisomal multifunctional enzyme type 2
AVTEL	Q13608	PEX6	Peroxisome assembly factor 2
ELLSL	Q13608	PEX6	Peroxisome assembly factor 2
ITREF	Q07869	PPARA	Peroxisome proliferator-activated receptor alpha
SELLS	Q86YN6	PRGC2	Peroxisome proliferator-activated receptor gamma coactivator 1-beta
LQLLM	Q5VV67	PPRC1	Peroxisome proliferator-activated receptor gamma coactivator-related protein 1
EGEVN	Q8WY91	THAP4	Peroxynitrite isomerase THAP4
PIVNK	O00541	PESC	Pescadillo homolog

SALLS	Q8IYB4	PEX5R	PEX5-related protein
SGVAV	A5PKW4	PSD1	PH and SEC7 domain-containing protein 1
ELLIL	Q6ZVD8	PHLP2	PH domain leucine-rich repeat-containing protein phosphatase 2
LPSEI	Q9P1Y6	PHRF1	PHD and RING finger domain-containing protein 1
RQQSY	O43189	PHF1	PHD finger protein 1
KKRKR	Q86YI8	PHF13	PHD finger protein 13
KRKRR	Q86YI8	PHF13	PHD finger protein 13
STPVT	Q5T6S3	PHF19	PHD finger protein 19
EEVLA	A8MW92	P20L1	PHD finger protein 20-like protein 1
SSSVI	A8MW92	P20L1	PHD finger protein 20-like protein 1
KKTNV	Q9BUL5	PHF23	PHD finger protein 23
SKKRK	Q9BUL5	PHF23	PHD finger protein 23
NIETV	Q92576	PHF3	PHD finger protein 3
AFIRK	P30039	PBLD	Phenazine biosynthesis-like domain-containing protein
GINNI	Q9Y285	SYFA	Phenylalanine--tRNA ligase alpha subunit
FYDPL	P21439	MDR3	Phosphatidylcholine translocator ABCB4
GKSTT	P21439	MDR3	Phosphatidylcholine translocator ABCB4
AVSKV	Q9Y2H2	SAC2	Phosphatidylinositide phosphatase SAC2
TELQL	Q8TCU6	PREX1	Phosphatidylinositol 3,4,5-trisphosphate-dependent Rac exchanger 1 protein
NKAVV	Q8TCU6	PREX1	Phosphatidylinositol 3,4,5-trisphosphate-dependent Rac exchanger 1 protein
EGSNI	Q70Z35	PREX2	Phosphatidylinositol 3,4,5-trisphosphate-dependent Rac exchanger 2

			protein
KARST	O00329	PK3CD	Phosphatidylinositol 4,5-bisphosphate 3-kinase catalytic subunit delta isoform
LYYVN	P48736	PK3CG	Phosphatidylinositol 4,5-bisphosphate 3-kinase catalytic subunit gamma isoform
NIMIT	P48736	PK3CG	Phosphatidylinositol 4,5-bisphosphate 3-kinase catalytic subunit gamma isoform
AVVSL	P42356	PI4KA	Phosphatidylinositol 4-kinase alpha
SISQV	P42356	PI4KA	Phosphatidylinositol 4-kinase alpha
GVAVS	Q9UBF8	PI4KB	Phosphatidylinositol 4-kinase beta
VTELQ	O75747	P3C2G	Phosphatidylinositol 4-phosphate 3-kinase C2 domain-containing subunit gamma
SLTLP	O75747	P3C2G	Phosphatidylinositol 4-phosphate 3-kinase C2 domain-containing subunit gamma
DTVSV	Q99755	PI51A	Phosphatidylinositol 4-phosphate 5-kinase type-1 alpha
IELSN	O14986	PI51B	Phosphatidylinositol 4-phosphate 5-kinase type-1 beta
DTVSV	O14986	PI51B	Phosphatidylinositol 4-phosphate 5-kinase type-1 beta
IELSN	O60331	PI51C	Phosphatidylinositol 4-phosphate 5-kinase type-1 gamma
DTVSV	O60331	PI51C	Phosphatidylinositol 4-phosphate 5-kinase type-1 gamma
TNTKE	Q92535	PIGC	Phosphatidylinositol N-acetylglucosaminyltransferase subunit C
LTAVT	Q14442	PIGH	Phosphatidylinositol N-acetylglucosaminyltransferase subunit H
PVSTY	Q9UMZ3	PTPRQ	Phosphatidylinositol phosphatase PTPRQ precursor
NIAFS	Q9UMZ3	PTPRQ	Phosphatidylinositol phosphatase PTPRQ precursor

SAVSK	Q13492	PICAL	Phosphatidylinositol-binding clathrin assembly protein
KGYLS	O95870	ABHGA	Phosphatidylserine lipase ABHD16A
TLSKD	P48651	PTSS1	Phosphatidylserine synthase 1
TELQL	Q6VY07	PACS1	Phosphofurin acidic cluster sorting protein 1
NSLTL	Q96G03	PGM2	Phosphoglucomutase-2
ILTAV	Q99570	PI3R4	Phosphoinositide 3-kinase regulatory subunit 4
SVITS	Q99570	PI3R4	Phosphoinositide 3-kinase regulatory subunit 4
LILKA	Q5UE93	PI3R6	Phosphoinositide 3-kinase regulatory subunit 6
KLIKQ	Q96KN8	PLAT5	Phospholipase A and acyltransferase 5
KAVVS	Q96KN8	PLAT5	Phospholipase A and acyltransferase 5
AVVSL	Q96KN8	PLAT5	Phospholipase A and acyltransferase 5
VLDLK	Q6P4A8	PLBL1	Phospholipase B-like 1 precursor
KEEVL	O94830	DDHD2	Phospholipase DDHD2
IIVII	O14494	PLPP1	Phospholipid phosphatase 1
IVILL	Q96GM1	PLPR2	Phospholipid phosphatase-related protein type 2
VTELQ	P55058	PLTP	Phospholipid transfer protein precursor
TELQL	P55058	PLTP	Phospholipid transfer protein precursor
VGSAI	Q9Y2Q0	AT8A1	Phospholipid-transporting ATPase IA
VKLIK	P98196	AT11A	Phospholipid-transporting ATPase IH
ILLSL	O60423	AT8B3	Phospholipid-transporting ATPase IK
KARST	O60423	AT8B3	Phospholipid-transporting ATPase IK
SAIAS	O60312	AT10A	Phospholipid-transporting ATPase VA
LSALR	Q9HAB8	PPCS	Phosphopantothenate--cysteine ligase

RELPR	Q9NWQ8	PHAG1	Phosphoprotein associated with glycosphingolipid-enriched microdomains 1
MELLI	Q14558	KPRA	Phosphoribosyl pyrophosphate synthase-associated protein 1
MELLI	O60256	KPRB	Phosphoribosyl pyrophosphate synthase-associated protein 2
LEGEV	Q93100	KPBB	Phosphorylase b kinase regulatory subunit beta
GLLLY	Q63HQ2	EGFLA	Pikachurin precursor
LLLYC	Q63HQ2	EGFLA	Pikachurin precursor
IIVIL	P41586	PACR	Pituitary adenylate cyclase-activating polypeptide type I receptor precursor
KRKRR	P28069	PIT1	Pituitary-specific positive transcription factor 1
NFYDP	Q8TC59	PIWL2	Piwi-like protein 2
YTLNN	Q99959	PKP2	Plakophilin-2
LSLIA	Q99959	PKP2	Plakophilin-2
TPVST	Q99569	PKP4	Plakophilin-4
KSTTN	Q99569	PKP4	Plakophilin-4
VLTSK	Q9BTY2	FUCO2	Plasma alpha-L-fucosidase precursor
MELLI	Q01814	AT2B2	Plasma membrane calcium-transporting ATPase 2
VLDLK	Q01814	AT2B2	Plasma membrane calcium-transporting ATPase 2
VLDLK	Q16720	AT2B3	Plasma membrane calcium-transporting ATPase 3
KVLDL	P23634	AT2B4	Plasma membrane calcium-transporting ATPase 4
LNNAK	Q14651	PLSI	Plastin-1
LSGIN	Q14651	PLSI	Plastin-1
LNNAK	P13796	PLSL	Plastin-2

AGKST	P13797	PLST	Plastin-3
TSKVL	P16284	PECA1	Platelet endothelial cell adhesion molecule precursor
NGTDA	P16671	CD36	Platelet glycoprotein 4
LLSTN	P25105	PTAFR	Platelet-activating factor receptor
VVQLP	P25105	PTAFR	Platelet-activating factor receptor
NKIKS	P16234	PGFRA	Platelet-derived growth factor receptor alpha precursor
TPVTL	P16234	PGFRA	Platelet-derived growth factor receptor alpha precursor
KKRKR	P04085	PDGFA	Platelet-derived growth factor subunit A precursor
RLLEI	P04085	PDGFA	Platelet-derived growth factor subunit A precursor
ASGVA	Q96S99	PKHF1	Pleckstrin homology domain-containing family F member 1
NSELL	Q9ULL1	PKHG1	Pleckstrin homology domain-containing family G member 1
VTTPV	Q9H7P9	PKHG2	Pleckstrin homology domain-containing family G member 2
SLTLP	Q9ULM0	PKHH1	Pleckstrin homology domain-containing family H member 1
TLPSE	Q9ULM0	PKHH1	Pleckstrin homology domain-containing family H member 1
TLPSE	Q8IVE3	PKHH2	Pleckstrin homology domain-containing family H member 2
AVSKG	Q8IWE5	PKHM2	Pleckstrin homology domain-containing family M member 2
TNDQK	Q5SXH7	PKHS1	Pleckstrin homology domain-containing family S member 1
CKIMT	Q5SXH7	PKHS1	Pleckstrin homology domain-containing family S member 1
KSALL	Q86UU1	PHLB1	Pleckstrin homology-like domain family B member 1
ASISQ	O75051	PLXA2	Plexin-A2 precursor
IIVII	O75051	PLXA2	Plexin-A2 precursor
IVIIV	O75051	PLXA2	Plexin-A2 precursor
AVTEL	O43157	PLXB1	Plexin-B1 precursor

LTAVT	O15031	PLXB2	Plexin-B2 precursor
GQNIT	O60486	PLXC1	Plexin-C1 precursor
SSSVI	O60486	PLXC1	Plexin-C1 precursor
LPSEI	Q9Y4D7	PLXD1	Plexin-D1 precursor
MTSKT	P54277	PMS1	PMS1 protein homolog 1
KTDVS	P54277	PMS1	PMS1 protein homolog 1
EEVLA	Q7Z3K3	POGZ	Pogo transposable element with ZNF domain
LEGEV	Q9UHX1	PUF60	Poly(U)-binding-splicing factor PUF60
NSELL	P11940	PABP1	Polyadenylate-binding protein 1
NSELL	Q4VXU2	PAP1L	Polyadenylate-binding protein 1-like
NSELL	Q9H361	PABP3	Polyadenylate-binding protein 3
NSELL	Q13310	PABP4	Polyadenylate-binding protein 4
AVSKG	Q9NQ11	AT132	Polyamine-transporting ATPase 13A2
KQLLP	Q9H7F0	AT133	Polyamine-transporting ATPase 13A3
LKANA	Q8IXJ9	ASXL1	Polycomb group protein ASXL1
RARRE	Q8IXJ9	ASXL1	Polycomb group protein ASXL1
EFYQS	Q9BSM1	PCGF1	Polycomb group RING finger protein 1
DPLVF	O75530	EED	Polycomb protein EED
SIMSI	Q15022	SUZ12	Polycomb protein SUZ12
VILLS	Q9P0L9	PK2L1	Polycystic kidney disease 2-like 1 protein
LGFLL	Q9NTG1	PKDRE	Polycystic kidney disease and receptor for egg jelly-related protein precursor
LLGVG	Q9NTG1	PKDRE	Polycystic kidney disease and receptor for egg jelly-related protein

			precursor
LAYVV	Q9NTG1	PKDRE	Polycystic kidney disease and receptor for egg jelly-related protein precursor
LTSKV	Q8TDX9	PK1L1	Polycystic kidney disease protein 1-like 1
LSKDQ	Q7Z443	PK1L3	Polycystic kidney disease protein 1-like 3 precursor
IKQEL	P98161	PKD1	Polycystin-1 precursor
GSVSF	P98161	PKD1	Polycystin-1 precursor
SEINL	P98161	PKD1	Polycystin-1 precursor
AVSKG	Q10472	GALT1	Polypeptide N-acetylgalactosaminyltransferase 1
KNNRL	Q8NCW6	GLT11	Polypeptide N-acetylgalactosaminyltransferase 11
RSTPV	Q92562	FIG4	Polyphosphoinositide phosphatase
YGKTK	Q9H8P0	PORED	Polyprenol reductase
GKTKC	Q9H8P0	PORED	Polyprenol reductase
VTLSK	Q9UKA9	PTBP2	Polypyrimidine tract-binding protein 2
SDELL	Q9UKA9	PTBP2	Polypyrimidine tract-binding protein 2
SDELL	O95758	PTBP3	Polypyrimidine tract-binding protein 3
SCSIS	Q6ICG8	WBP2L	Postacrosomal sheath WW domain-binding protein
KRRFL	O00180	KCNK1	Potassium channel subfamily K member 1
GVSVL	O00180	KCNK1	Potassium channel subfamily K member 1
TASNK	Q9HB15	KCNKC	Potassium channel subfamily K member 12
PIVNK	P48547	KCNC1	Potassium voltage-gated channel subfamily C member 1
NRLLE	Q96PR1	KCNC2	Potassium voltage-gated channel subfamily C member 2
LGFLL	Q9NSA2	KCND1	Potassium voltage-gated channel subfamily D member 1

LGFLL	Q9NZV8	KCND2	Potassium voltage-gated channel subfamily D member 2
LGFLL	Q9UK17	KCND3	Potassium voltage-gated channel subfamily D member 3
AVTEL	Q9ULD8	KCNH3	Potassium voltage-gated channel subfamily H member 3
TLNNA	Q96L42	KCNH8	Potassium voltage-gated channel subfamily H member 8
VGLLL	Q9ULS6	KCNS2	Potassium voltage-gated channel subfamily S member 2
VGLLL	Q9BQ31	KCNS3	Potassium voltage-gated channel subfamily S member 3
VGLLL	Q6PIU1	KCNV1	Potassium voltage-gated channel subfamily V member 1
ALLST	Q8TDN2	KCNV2	Potassium voltage-gated channel subfamily V member 2
VSVLT	Q9UL51	HCN2	Potassium/sodium hyperpolarization-activated cyclic nucleotide-gated channel 2
VSVLT	Q9Y3Q4	HCN4	Potassium/sodium hyperpolarization-activated cyclic nucleotide-gated channel 4
LGAIV	P20648	ATP4A	Potassium-transporting ATPase alpha chain 1
GVAVS	P54707	AT12A	Potassium-transporting ATPase alpha chain 2
LSNGV	P0CG38	POTEI	POTE ankyrin domain family member I
LSNGV	P0CG39	POTEJ	POTE ankyrin domain family member J
KKRKR	Q01851	PO4F1	POU domain, class 4, transcription factor 1
KKRKR	Q12837	PO4F2	POU domain, class 4, transcription factor 2
SKKRK	Q14863	PO6F1	POU domain, class 6, transcription factor 1
KKRKR	Q14863	PO6F1	POU domain, class 6, transcription factor 1
KRKRR	Q14863	PO6F1	POU domain, class 6, transcription factor 1
SKKRK	P78424	PO6F2	POU domain, class 6, transcription factor 2
KKRKR	P78424	PO6F2	POU domain, class 6, transcription factor 2

KRKRR	P78424	PO6F2	POU domain, class 6, transcription factor 2
FIRKS	Q9NQV6	PRD10	PR domain zinc finger protein 10
QEGKS	Q9GZV8	PRD14	PR domain zinc finger protein 14
KLIKQ	P57071	PRD15	PR domain zinc finger protein 15
ELQLL	P57071	PRD15	PR domain zinc finger protein 15
TCSAV	Q13029	PRDM2	PR domain zinc finger protein 2
AVGLL	O60831	PRAF2	PRA1 family protein 2
SNIKE	O60809	PRA10	PRAME family member 10
SNIKE	Q5VTA0	PRA17	PRAME family member 17
SNIKE	A0A0G2JMD5	PRA33	PRAME family member 33
LYCKA	P61758	PFD3	Prefoldin subunit 3
FLLGV	P11464	PSG1	Pregnancy-specific beta-1-glycoprotein 1 precursor
FLLGV	Q9UQ74	PSG8	Pregnancy-specific beta-1-glycoprotein 8 precursor
CSISN	Q9UMS4	PRP19	Pre-mRNA-processing factor 19
LILKA	Q6P2Q9	PRP8	Pre-mRNA-processing-splicing factor 8
RFLGF	Q6P2Q9	PRP8	Pre-mRNA-processing-splicing factor 8
FLLGV	Q99633	PRP18	Pre-mRNA-splicing factor 18
DELLH	Q8NAV1	PR38A	Pre-mRNA-splicing factor 38A
TLSKK	Q2NL82	TSR1	Pre-rRNA-processing protein TSR1 homolog
NQSLA	Q96MT3	PRIC1	Prickle-like protein 1 precursor
SDELL	Q7Z3G6	PRIC2	Prickle-like protein 2 precursor
DELLH	Q7Z3G6	PRIC2	Prickle-like protein 2 precursor
VLAYV	Q96BN6	F149B	Primary cilium assembly protein FAM149B1

IVILL	Q96HY7	DHTK1	Probable 2-oxoglutarate dehydrogenase E1 component DHKTD1, mitochondrial precursor
KSLYV	P26196	DDX6	Probable ATP-dependent RNA helicase DDX6
NITEE	Q8IY21	DDX60	Probable ATP-dependent RNA helicase DDX60
EFYQS	Q8IY21	DDX60	Probable ATP-dependent RNA helicase DDX60
ELLSL	Q8IY21	DDX60	Probable ATP-dependent RNA helicase DDX60
ELLSL	Q5H9U9	DDX6L	Probable ATP-dependent RNA helicase DDX60-like
SDELL	Q5H9U9	DDX6L	Probable ATP-dependent RNA helicase DDX60-like
DELLH	Q5H9U9	DDX6L	Probable ATP-dependent RNA helicase DDX60-like
FLLGV	Q14147	DHX34	Probable ATP-dependent RNA helicase DHX34
VGLLL	Q14147	DHX34	Probable ATP-dependent RNA helicase DHX34
VQLPL	Q9H903	MTD2L	Probable bifunctional methylenetetrahydrofolate dehydrogenase/cyclohydrolase 2
LGVGS	Q8TBB6	S7A14	Probable cationic amino acid transporter
FASGQ	Q4VNC1	AT134	Probable cation-transporting ATPase 13A4
YLSAL	Q4VNC1	AT134	Probable cation-transporting ATPase 13A4
IMSII	Q4VNC1	AT134	Probable cation-transporting ATPase 13A4
ILLSL	Q4VNC1	AT134	Probable cation-transporting ATPase 13A4
FASGQ	Q4VNC0	AT135	Probable cation-transporting ATPase 13A5
LSALR	Q2PZI1	D19L1	Probable C-mannosyltransferase DPY19L1
SNIKE	Q6ZPD9	D19L3	Probable C-mannosyltransferase DPY19L3
SAIAS	Q9HA77	SYCM	Probable cysteine--tRNA ligase, mitochondrial precursor
LKNYI	Q9HA77	SYCM	Probable cysteine--tRNA ligase, mitochondrial precursor

SELLS	Q5U5R9	HECD2	Probable E3 ubiquitin-protein ligase HECTD2
DELLH	Q9Y4D8	HECD4	Probable E3 ubiquitin-protein ligase HECTD4
LQLLM	Q15751	HERC1	Probable E3 ubiquitin-protein ligase HERC1
SKVLD	Q15034	HERC3	Probable E3 ubiquitin-protein ligase HERC3
SLIND	Q15034	HERC3	Probable E3 ubiquitin-protein ligase HERC3
ASGVA	Q9H1B7	I2BPL	Probable E3 ubiquitin-protein ligase IRF2BPL
ITTIL	P28370	SMCA1	Probable global transcription activator SNF2L1
KKRKR	P51531	SMCA2	Probable global transcription activator SNF2L2
KRKRR	P51531	SMCA2	Probable global transcription activator SNF2L2
QSTPP	Q96P66	GP101	Probable G-protein coupled receptor 101
VVSLS	Q86SP6	GP149	Probable G-protein coupled receptor 149
VLAYV	Q9UJ42	GP160	Probable G-protein coupled receptor 160
SALLS	Q6PRD1	GP179	Probable G-protein coupled receptor 179 precursor
AVGLL	O00155	GPR25	Probable G-protein coupled receptor 25
LGFLL	Q9NS67	GPR27	Probable G-protein coupled receptor 27
LLGVG	Q9NS67	GPR27	Probable G-protein coupled receptor 27
LGFLL	Q49SQ1	GPR33	Probable G-protein coupled receptor 33
SKKRK	Q7Z333	SETX	Probable helicase senataxin
RARRE	Q96NU7	HUTI	Probable imidazolonepropionase
ARREL	Q96NU7	HUTI	Probable imidazolonepropionase
LLEIT	Q4AC99	1A1L2	Probable inactive 1-aminocyclopropane-1-carboxylate synthase-like protein 2
SSSVI	Q8IXY8	PPIL6	Probable inactive peptidyl-prolyl cis-trans isomerase-like 6

KGYLS	Q8N6Q8	MET25	Probable methyltransferase-like protein 25
EEVLA	Q8N8M0	NAT16	Probable N-acetyltransferase 16
NAGVT	A0A0A0MS04	TVB67	Probable non-functional T cell receptor beta variable 6-7 precursor
ELLSL	O75110	ATP9A	Probable phospholipid-transporting ATPase IIA
IIKEE	Q8TAA1	RNS11	Probable ribonuclease 11 precursor
SLIND	Q5HYM0	ZC12B	Probable ribonuclease ZC3H12B
SLSNG	Q9C0D7	ZC12C	Probable ribonuclease ZC3H12C
INLCN	Q9C0D7	ZC12C	Probable ribonuclease ZC3H12C
SCSIS	Q9Y4C8	RBM19	Probable RNA-binding protein 19
NSELL	Q8IYL2	TRM44	Probable tRNA (uracil-O(2)-)-methyltransferase
VSSSV	Q93008	USP9X	Probable ubiquitin carboxyl-terminal hydrolase FAF-X
KVLHL	Q4G0A6	MINY4	Probable ubiquitin carboxyl-terminal hydrolase MINDY-4
VLHLE	Q4G0A6	MINY4	Probable ubiquitin carboxyl-terminal hydrolase MINDY-4
KSALL	Q8WXW3	PIBF1	Progesterone-induced-blocking factor 1
LSTNK	Q8WXW3	PIBF1	Progesterone-induced-blocking factor 1
ELLHN	Q8WXW3	PIBF1	Progesterone-induced-blocking factor 1
KDQLS	Q9NZQ7	PD1L1	Programmed cell death 1 ligand 1 precursor
LGVGS	Q9HCJ1	ANKH	Progressive ankylosis protein homolog
VILLS	P49683	PRLHR	Prolactin-releasing peptide receptor
LAFIR	P51888	PRELP	Prolargin precursor
KEEVL	P46013	KI67	Proliferation marker protein Ki-67
GVSVL	Q9UQ80	PA2G4	Proliferation-associated protein 2G4
VSLSN	Q8IZL8	PELP1	Proline-, glutamic acid- and leucine-rich protein 1

RKRRF	Q6MZM9	PRR27	Proline-rich protein 27 precursor
DQLSG	Q92733	PRCC	Proline-rich protein PRCC
GSAIA	C9JH25	PRRT4	Proline-rich transmembrane protein 4 precursor
VNKQS	Q07954	LRP1	Prolow-density lipoprotein receptor-related protein 1 precursor
LLSLI	Q9H6Z9	EGLN3	Prolyl hydroxylase EGLN3
QSCSI	Q92824	PCSK5	Proprotein convertase subtilisin/kexin type 5 precursor
SCSIS	Q92824	PCSK5	Proprotein convertase subtilisin/kexin type 5 precursor
FIRKS	Q8NBP7	PCSK9	Proprotein convertase subtilisin/kexin type 9 precursor
VTELQ	Q16651	PRSS8	Prostasin precursor
TRTDR	B3GLJ2	PATE3	Prostate and testis expressed protein 3 precursor
AVGLL	O43653	PSCA	Prostate stem cell antigen precursor
KKRKR	Q14997	PSME4	Proteasome activator complex subunit 4
SALLS	Q5VYK3	ECM29	Proteasome adapter and scaffold protein ECM29
VFPSD	Q5VYK3	ECM29	Proteasome adapter and scaffold protein ECM29
NKIKS	Q969U7	PSMG2	Proteasome assembly chaperone 2
VSVGN	Q969U7	PSMG2	Proteasome assembly chaperone 2
KANAI	O14818	PSA7	Proteasome subunit alpha type-7
KANAI	Q8TAA3	PSMA8	Proteasome subunit alpha-type 8
LSTNK	Q0P651	ABD18	Protein ABHD18 precursor
LHLEG	P42568	AF9	Protein AF-9
VAVSK	Q9BXJ7	AMNLS	Protein amnionless precursor
AVSKV	Q9BXJ7	AMNLS	Protein amnionless precursor
LLILK	A6NFN9	ANKUB	Protein ANKUB1

LILKA	A6NFN9	ANKUB	Protein ANKUB1
IIVIL	Q9UL18	ago-01	Protein argonaute-1
GINNI	Q9UL18	ago-01	Protein argonaute-1
ELQLL	Q9H9G7	ago-03	Protein argonaute-3
IIVIL	Q9H9G7	ago-03	Protein argonaute-3
GINNI	Q9H9G7	ago-03	Protein argonaute-3
ELQLL	Q9HCK5	ago-04	Protein argonaute-4
CTASN	Q3KR37	ASTRB	Protein Aster-B
TDVSS	Q6PGQ7	BORA	Protein aurora borealis
TSPLC	Q9UPA5	BSN	Protein bassoon
LSALR	Q8TD16	BICD2	Protein bicaudal D homolog 2
NRARR	P0C671	BNIP5	Protein BNIP5
VSKGY	Q8N9W6	BOLL	Protein boule-like
SELLS	Q96RK0	CIC	Protein capicua homolog
VSSSV	Q06455	MTG8	Protein CBFA2T1
KQLLP	Q6DHV5	C2D2B	Protein CC2D2B
ELLSL	P14921	ETS1	Protein C-ets-1
NKGMD	Q2KHT3	CL16A	Protein CLEC16A
FLGFL	Q9UBY8	CLN8	Protein CLN8
TVSVG	O75128	COBL	Protein cordon-bleu
SALLS	Q8IUH2	CREG2	Protein CREG2 precursor
VKLIK	Q9P219	DAPLE	Protein Daple
RARRE	Q5TDH0	DDI2	Protein DDI1 homolog 2

IETVI	Q8NEG7	DEN6B	Protein DENND6B
LLSLI	Q8NEG7	DEN6B	Protein DENND6B
LSLIA	Q8NEG7	DEN6B	Protein DENND6B
LLEIT	O60610	DIAP1	Protein diaphanous homolog 1
NRLLE	O60879	DIAP2	Protein diaphanous homolog 2
FPQAE	O60879	DIAP2	Protein diaphanous homolog 2
RLLEI	Q6UXH1	CREL2	Protein disulfide isomerase CRELD2 precursor
LSALR	Q15084	PDIA6	Protein disulfide-isomerase A6 precursor
SNKNR	Q15084	PDIA6	Protein disulfide-isomerase A6 precursor
VNAGV	Q8N807	PDILT	Protein disulfide-isomerase-like protein of the testis precursor
EEVLA	Q8N807	PDILT	Protein disulfide-isomerase-like protein of the testis precursor
NEKIN	Q9C005	DPY30	Protein dpy-30 homolog
SVLTS	Q9H8V3	ECT2	Protein ECT2
ITIEL	Q14156	EFR3A	Protein EFR3 homolog A
EPIIN	Q8WYP5	ELYS	Protein ELYS
QELDK	P49257	LMAN1	Protein ERGIC-53 precursor
DVSSS	Q5T1H1	EYS	Protein eyes shut homolog precursor
AVGLL	Q5T1H1	EYS	Protein eyes shut homolog precursor
GLLLY	Q5T1H1	EYS	Protein eyes shut homolog precursor
TSKVL	Q9NRY5	F1142	Protein FAM114A2
LSKKR	Q8N9E0	F133A	Protein FAM133A
SKKRK	Q8N9E0	F133A	Protein FAM133A
LSKKR	Q5BKY9	F133B	Protein FAM133B

SKKRK	Q5BKY9	F133B	Protein FAM133B
KLIKQ	Q9P2D6	F135A	Protein FAM135A
LDLKN	Q9NYF5	FA13B	Protein FAM13B
DVSSS	Q96A26	F162A	Protein FAM162A
GVAVS	A1A519	F170A	Protein FAM170A
ELQLL	Q8IXR5	F178B	Protein FAM178B
TSLGA	Q8IXR5	F178B	Protein FAM178B
ALLST	A6NEQ2	F181B	Protein FAM181B
LSLIA	Q8IYM0	F186B	Protein FAM186B
DVSSS	Q6PEV8	F199X	Protein FAM199X
RLLEI	Q8TCP9	F200A	Protein FAM200A
LTAVT	Q9NTX9	F217B	Protein FAM217B
ELDKY	P0C875	F228B	Protein FAM228B
LPSEI	P58499	FAM3B	Protein FAM3B precursor
LKANA	Q5T0W9	FA83B	Protein FAM83B
KNRGI	Q5T0W9	FA83B	Protein FAM83B
KAVVS	Q6ZRV2	FA83H	Protein FAM83H
TKEGS	Q8IZU1	FAM9A	Protein FAM9A
AFIRK	Q92636	FAN	Protein FAN
SKKRK	Q70Z53	F10C1	Protein FRA10AC1
KKRKR	Q14331	FRG1	Protein FRG1
NAKKT	Q5TBA9	FRY	Protein furry homolog
GLLLY	Q4ZG55	GREB1	Protein GREB1

ELLSL	Q86T90	K1328	Protein hinderin
DASIS	Q86T90	K1328	Protein hinderin
KVQSN	A6NMK8	INY2B	Protein INSYN2B
QSTCS	P28290	ITPI2	Protein ITPRID2
KNNRL	Q92613	JADE3	Protein Jade-3
NNRLL	Q92613	JADE3	Protein Jade-3
KTKCT	P78504	JAG1	Protein jagged-1 precursor
LSALR	Q9Y219	JAG2	Protein jagged-2 precursor
DVSSS	Q92833	JARD2	Protein Jumonji
AGKST	Q92833	JARD2	Protein Jumonji
ARREL	Q8N6L0	KASH5	Protein KASH5
GVSVL	O15037	KHNYN	Protein KHNYN
ELLSL	O15037	KHNYN	Protein KHNYN
TLSKK	Q8IX03	KIBRA	Protein KIBRA
RTDRG	Q8IX03	KIBRA	Protein KIBRA
GVSVL	Q05655	KPCD	Protein kinase C delta type
LEGEV	Q92832	NELL1	Protein kinase C-binding protein NELL1 precursor
VITIE	Q9NVR5	KTU	Protein kintoun
ITIEL	Q9NVR5	KTU	Protein kintoun
AFIRK	Q7Z429	LFG1	Protein lifeguard 1
NGVSV	O14910	LIN7A	Protein lin-7 homolog A
NGVSV	Q9HAP6	LIN7B	Protein lin-7 homolog B
NGVSV	Q9NUP9	LIN7C	Protein lin-7 homolog C

GYLSA	Q9Y586	MB212	Protein mab-21-like 2
LGVGS	Q8IYB1	M21D2	Protein MB21D2
GLLLY	Q6ZRQ5	MMS22	Protein MMS22-like
QSLAF	Q53GL7	PAR10	Protein mono-ADP-ribosyltransferase PARP10
GVAVS	Q460N5	PAR14	Protein mono-ADP-ribosyltransferase PARP14
ELLSL	Q9UKK3	PARP4	Protein mono-ADP-ribosyltransferase PARP4
TVSVG	Q9UKK3	PARP4	Protein mono-ADP-ribosyltransferase PARP4
VSVGN	Q9UKK3	PARP4	Protein mono-ADP-ribosyltransferase PARP4
DASIS	Q9UKK3	PARP4	Protein mono-ADP-ribosyltransferase PARP4
STPVT	A6PVC2	TTLL8	Protein monoglycylase TTLL8
SALLS	Q9BZQ8	NIBA1	Protein Niban 1
ELPRF	Q96TA1	NIBA2	Protein Niban 2
ELDKY	Q9HAS0	NJMU	Protein Njmu-R1
VILLS	Q86WI3	NLRC5	Protein NLRC5
ILLSL	Q86WI3	NLRC5	Protein NLRC5
LPSEI	Q14207	NPAT	Protein NPAT
YGKTK	Q14207	NPAT	Protein NPAT
ILLSL	Q6UW63	PLGT2	Protein O-glucosyltransferase 2 precursor
LLLYC	Q6UW63	PLGT2	Protein O-glucosyltransferase 2 precursor
ILLSL	Q7Z4H8	PLGT3	Protein O-glucosyltransferase 3 precursor
AVGLL	Q9H5K3	SG196	Protein O-mannose kinase
VGLLL	Q9H5K3	SG196	Protein O-mannose kinase
ELLSL	Q5T4D3	TMTC4	Protein O-mannosyl-transferase TMTC4

SALLS	Q96SN7	ORAI2	Protein orai-2
SALLS	Q9BRQ5	ORAI3	Protein orai-3
KQELD	Q8N3R9	PALS1	Protein PALS1
TELQL	Q96T49	PP16B	Protein phosphatase 1 regulatory inhibitor subunit 16B precursor
ELQLL	Q96T49	PP16B	Protein phosphatase 1 regulatory inhibitor subunit 16B precursor
RARRE	O60237	MYPT2	Protein phosphatase 1 regulatory subunit 12B
SELLS	Q9NXH3	PP14D	Protein phosphatase 1 regulatory subunit 14D
KRKRR	Q5T8A7	PPR26	Protein phosphatase 1 regulatory subunit 26
VLHLE	O75864	PPR37	Protein phosphatase 1 regulatory subunit 37
FPSDE	O75864	PPR37	Protein phosphatase 1 regulatory subunit 37
ELDKY	P35813	PPM1A	Protein phosphatase 1A
TPPTN	O15297	PPM1D	Protein phosphatase 1D
STPPT	Q9ULR3	PPM1H	Protein phosphatase 1H
NAGKS	Q9ULR3	PPM1H	Protein phosphatase 1H
AGKST	Q9ULR3	PPM1H	Protein phosphatase 1H
NAGKS	Q5JR12	PPM1J	Protein phosphatase 1J
SAVSK	Q9Y6V0	PCLO	Protein piccolo
KQELD	Q9Y6V0	PCLO	Protein piccolo
TLPSE	Q9Y6V0	PCLO	Protein piccolo
TNSEL	Q6ZMV5	P4R3C	Protein PPP4R3C
SAVSK	Q5THK1	PR14L	Protein PRR14L
LLMQS	Q5THK1	PR14L	Protein PRR14L
KIMTS	Q5THK1	PR14L	Protein PRR14L

SALLS	P48634	PRC2A	Protein PRRC2A
RTDRG	P48634	PRC2A	Protein PRRC2A
SLTLP	P48634	PRC2A	Protein PRRC2A
IKQEL	Q5JSZ5	PRC2B	Protein PRRC2B
NRLLE	Q9Y520	PRC2C	Protein PRRC2C
IASGV	Q8WUY3	PRUN2	Protein prune homolog 2
PSEIN	Q8WUY3	PRUN2	Protein prune homolog 2
KKLMS	Q96PU8	QKI	Protein quaking
VVSLS	Q14690	RRP5	Protein RRP5 homolog
SLIAV	P31949	S10AB	Protein S100-A11
KLMSN	P26447	S10A4	Protein S100-A4
TLSKK	P06703	S10A6	Protein S100-A6
QELDK	P29377	S100G	Protein S100-G
KKRKR	Q9NVU7	SDA1	Protein SDA1 homolog
ELLSL	Q9NVU7	SDA1	Protein SDA1 homolog
LSGIN	Q5TEA6	SE1L2	Protein sel-1 homolog 2 precursor
RRELP	A8K8P3	SFI1	Protein SFI1 homolog
KRRFL	A8K8P3	SFI1	Protein SFI1 homolog
QQSYS	Q6UWV7	SHL2A	Protein shisa-like-2A
QEGKS	A6NKW6	SHL2B	Protein shisa-like-2B
SALLS	Q13796	SHRM2	Protein Shroom2
RELPR	Q7Z5N4	SDK1	Protein sidekick-1 precursor
IVILL	Q7Z5N4	SDK1	Protein sidekick-1 precursor

IKENK	Q8N1H7	S6OS1	Protein SIX6OS1
VVSLS	P0C7P3	SLN14	Protein SLFN14
QLLMQ	Q92540	SMG7	Protein SMG7
TVSVG	O94964	SOGA1	Protein SOGA1
VTTPV	P18583	SON	Protein SON
VVSLS	Q96EA4	SPDLY	Protein Spindly
KIKSA	Q08AE8	SPIR1	Protein spire homolog 1
TTIII	Q5VVC0	SPO16	Protein SPO16 homolog
KKRKR	Q8N9Q2	SR1IP	Protein SREK1IP1
EITRE	A3KN83	SBNO1	Protein strawberry notch homolog 1
KRKRR	Q9Y2G9	SBNO2	Protein strawberry notch homolog 2
DVSSS	Q9Y2G9	SBNO2	Protein strawberry notch homolog 2
LGFLL	Q9C0D5	TANC1	Protein TANC1
GFLLG	Q9C0D5	TANC1	Protein TANC1
LGFLL	Q9HCD6	TANC2	Protein TANC2
GFLLG	Q9HCD6	TANC2	Protein TANC2
SISNI	Q9HCD6	TANC2	Protein TANC2
SALLS	Q5VWN6	TASO2	Protein TASOR 2
EVLAY	Q6UWH6	TX261	Protein TEX261
ITSLG	Q8N9V7	TOPZ1	Protein TOPAZ1
VSLSN	O15027	SC16A	Protein transport protein Sec16A
IAVGL	Q15436	SC23A	Protein transport protein Sec23A
AVGLL	Q15436	SC23A	Protein transport protein Sec23A

IAVGL	Q15437	SC23B	Protein transport protein Sec23B
AVGLL	Q15437	SC23B	Protein transport protein Sec23B
VITSL	P53992	SC24C	Protein transport protein Sec24C
QLLPI	O94855	SC24D	Protein transport protein Sec24D
TDAKV	Q9H3U1	UN45A	Protein unc-45 homolog A
NSLTL	Q9H3U1	UN45A	Protein unc-45 homolog A
VGNTL	Q53HI1	UNC50	Protein unc-50 homolog
GVAVS	Q8N2C7	UNC80	Protein unc-80 homolog
LLEIT	Q8N2C7	UNC80	Protein unc-80 homolog
GVGSA	Q9H1C4	UN93B	Protein unc-93 homolog B1
ILTAV	Q08AM6	VAC14	Protein VAC14 homolog
SALLS	Q9NRH1	YAE1	Protein YAE1 homolog
ILLSL	Q5BJH7	YIF1B	Protein YIF1B
SKKRK	Q8IZ13	ZBED8	Protein ZBED8
LDKYK	Q9UK55	ZPI	Protein Z-dependent protease inhibitor precursor
LTNSE	Q7Z7L7	ZER1	Protein zer-1 homolog
LPSEI	Q7Z7L7	ZER1	Protein zer-1 homolog
KIKSA	Q86YA3	ZGRF1	Protein ZGRF1
ELLSL	Q86YA3	ZGRF1	Protein ZGRF1
SSSVI	Q9H900	ZWILC	Protein zwilch homolog
MDTVS	Q9H900	ZWILC	Protein zwilch homolog
NSLTL	Q9C0D3	ZY11B	Protein zyg-11 homolog B
KKRKR	P55085	PAR2	Proteinase-activated receptor 2 precursor

ASGQN	P22735	TGM1	Protein-glutamine gamma-glutamyltransferase K
AGVTT	P22735	TGM1	Protein-glutamine gamma-glutamyltransferase K
LPSEI	Q96PF1	TGM7	Protein-glutamine gamma-glutamyltransferase Z
GIIKT	P22061	PIMT	Protein-L-isoaspartate(D-aspartate) O-methyltransferase
SLAFI	Q9H237	PORCN	Protein-serine O-palmitoleoyltransferase porcupine
TSKVL	Q92954	PRG4	Proteoglycan 4 precursor
EFYQS	Q9Y5I0	PCDAD	Protocadherin alpha-13 precursor
TNSEL	Q9Y5F1	PCDBC	Protocadherin beta-12 precursor
SNNVQ	Q9Y5E8	PCDBF	Protocadherin beta-15 precursor
SNNVQ	Q9NRJ7	PCDBG	Protocadherin beta-16 precursor
TTILT	Q14517	FAT1	Protocadherin Fat 1 precursor
KVLDL	Q8TDW7	FAT3	Protocadherin Fat 3 precursor
SSSVI	Q6V0I7	FAT4	Protocadherin Fat 4 precursor
SQVNE	Q6V0I7	FAT4	Protocadherin Fat 4 precursor
LQLLM	Q9Y5G9	PCDG4	Protocadherin gamma-A4 precursor
FSVNA	Q9Y5G7	PCDG6	Protocadherin gamma-A6 precursor
QNITE	Q9Y5G1	PCDGF	Protocadherin gamma-B3 precursor
ASISQ	Q9UN71	PCDGG	Protocadherin gamma-B4 precursor
SISQV	Q9UN71	PCDGG	Protocadherin gamma-B4 precursor
SYSIM	Q9Y5G0	PCDGH	Protocadherin gamma-B5 precursor
LLSLI	Q9UN70	PCDGK	Protocadherin gamma-C3 precursor
VLHLE	Q9P2E7	PCD10	Protocadherin-10 precursor
GSVSF	Q9P2E7	PCD10	Protocadherin-10 precursor

VNEKI	Q9P2E7	PCD10	Protocadherin-10 precursor
TIELS	Q96QU1	PCD15	Protocadherin-15 precursor
GINNI	Q96QU1	PCD15	Protocadherin-15 precursor
PVTLS	Q96JQ0	PCD16	Protocadherin-16 precursor
SLGAI	Q9HCL0	PCD18	Protocadherin-18 precursor
IVIIV	O95206	PCDH8	Protocadherin-8 precursor
AVGLL	Q96QE2	MYCT	Proton myo-inositol cotransporter
QLSGI	Q96QE2	MYCT	Proton myo-inositol cotransporter
LSGIN	Q96QE2	MYCT	Proton myo-inositol cotransporter
LGFLL	Q9Y2W3	S45A1	Proton-associated sugar transporter A
GFLLG	Q6YBV0	S36A4	Proton-coupled amino acid transporter 4
DVSSS	Q96NT5	PCFT	Proton-coupled folate transporter
VIIVI	Q9P0T7	TMEM9	Proton-transporting V-type ATPase complex assembly regulator TMEM9 precursor
LGVGS	P08922	ROS1	Proto-oncogene tyrosine-protein kinase ROS precursor
GAIVS	P08922	ROS1	Proto-oncogene tyrosine-protein kinase ROS precursor
GVSVL	P50336	PPOX	Protoporphyrinogen oxidase
AVTEL	Q14242	SELPL	P-selectin glycoprotein ligand 1 precursor
LGFLL	O14603	PRY	PTPN13-like protein, Y-linked
LSALR	Q58EX7	PKHG4	Puratrophin-1
EEVLA	P00491	PNPH	Purine nucleoside phosphorylase
FQQKN	P55786	PSA	Puromycin-sensitive aminopeptidase
DTVSV	P55786	PSA	Puromycin-sensitive aminopeptidase

FQQKN	A6NEC2	PSAL	Puromycin-sensitive aminopeptidase-like protein
IKENK	Q6A1A2	PDPK2	Putative 3-phosphoinositide-dependent protein kinase 2
TELQL	Q719I0	AHSA2	Putative activator of 90 kDa heat shock protein ATPase homolog 2
GKSLY	A6NMY6	AXA2L	Putative annexin A2-like protein
LSKKR	Q8N888	BCOR2	Putative BCoR-like protein 2
ELQLL	Q9GZL8	BPEC1	Putative BPES syndrome breakpoint region protein
IEFQQ	P59074	CHM4P	Putative charged multivesicular body protein 4B-like protein CHMP4BP1
VGLLL	A6NM45	CLD24	Putative claudin-24
KLMSN	A0A1B0GTZ2	CC196	Putative coiled-coil domain-containing protein 196
LPSEI	A0A1B0GTZ2	CC196	Putative coiled-coil domain-containing protein 196
KNAVT	P0C7P4	UCRIL	Putative cytochrome b-c1 complex subunit Rieske-like protein 1
VLDLK	P0C7P4	UCRIL	Putative cytochrome b-c1 complex subunit Rieske-like protein 1
VSSSV	Q9H9P5	UNKL	Putative E3 ubiquitin-protein ligase UNKL
QEGKS	Q0D2K5	EGFEM	Putative EGF-like and EMI domain-containing protein 1
GKSTT	Q5VTE0	EF1A3	Putative elongation factor 1-alpha-like 3
LTLPS	Q6ZTU2	E400N	Putative EP400-like protein
SVGNT	Q6ZTU2	E400N	Putative EP400-like protein
INQSL	P0C7X4	FHL19	Putative ferritin heavy polypeptide-like 19
RRELP	Q9BZA5	TXNG2	Putative gamma-taxilin 2
KKRKR	Q6DN03	H2B2C	Putative histone H2B type 2-C
KKRKR	Q6DRA6	H2B2D	Putative histone H2B type 2-D
ELSNI	Q8N7R0	NANG2	Putative homeobox protein NANOG2

LSLIA	Q5VSP4	LC1L1	Putative lipocalin 1-like protein 1 precursor
QSCSI	Q2TV78	MST1L	Putative macrophage stimulating 1-like protein precursor
EGKSL	Q9HBA9	FOH1B	Putative N-acetylated-alpha-linked acidic dipeptidase
GKSLY	Q9HBA9	FOH1B	Putative N-acetylated-alpha-linked acidic dipeptidase
PQAET	Q9BZK3	NACP4	Putative nascent polypeptide-associated complex subunit alpha-like protein
GYLSA	Q13394	MB211	Putative nucleotidyltransferase MAB21L1
STCSA	Q8NH80	O10D3	Putative olfactory receptor 10D3
STCSA	Q8NGN7	O10D4	Putative olfactory receptor 10D4
LLSLI	Q8NGN7	O10D4	Putative olfactory receptor 10D4
VITIE	Q8NHC6	O14L1	Putative olfactory receptor 14L1
VIIVI	Q8NGI1	O56B2	Putative olfactory receptor 56B2
LTAVT	Q8NH89	O5AK3	Putative olfactory receptor 5AK3
LGFLL	Q8NHP8	PLBL2	Putative phospholipase B-like 2 precursor
DTVSV	A2A3N6	PIPSL	Putative PIP5K1A and PSMD4-like protein
DKQLL	Q9C0F0	ASXL3	Putative Polycomb group protein ASXL3
IKSAL	Q3SYA9	P12L1	Putative POM121-like protein 1
EEVLA	Q7L7V1	DHX32	Putative pre-mRNA-splicing factor ATP-dependent RNA helicase DHX32
LGVGS	A6NEY8	PRXD1	Putative prolyl-tRNA synthetase associated domain-containing protein 1
KRRFL	Q9NVL1	F86C1	Putative protein FAM86C1P
VTLSK	Q8N7N1	F86B1	Putative protein N-methyltransferase FAM86B1

VTLSK	P0C5J1	F86B2	Putative protein N-methyltransferase FAM86B2
NVTLS	Q7RTT6	SSX6	Putative protein SSX6
VSSSV	Q6P474	PDXD2	Putative pyridoxal-dependent decarboxylase domain-containing protein 2
CSAVS	Q8N0V3	RBFA	Putative ribosome-binding factor A, mitochondrial precursor
NITEE	Q8IXW5	RPAP2	Putative RNA polymerase II subunit B1 CTD phosphatase RPAP2
ITEEF	Q8IXW5	RPAP2	Putative RNA polymerase II subunit B1 CTD phosphatase RPAP2
VSSSV	Q8IXW5	RPAP2	Putative RNA polymerase II subunit B1 CTD phosphatase RPAP2
SALRT	Q8NDT2	RB15B	Putative RNA-binding protein 15B
GVSVL	Q9HBR0	S38AA	Putative sodium-coupled neutral amino acid transporter 10
VSVLT	Q9HBR0	S38AA	Putative sodium-coupled neutral amino acid transporter 10
SVLTS	Q9NVC3	S38A7	Putative sodium-coupled neutral amino acid transporter 7
FLLGV	G3V0H7	SO1B7	Putative solute carrier organic anion transporter family member 1B7
NRLLE	P0DTA3	SPD11	Putative speedy protein E11
NRLLE	A0A494C0Z2	SPD13	Putative speedy protein E13
NRLLE	P0DUD3	SPD14	Putative speedy protein E14
NRLLE	P0DUD4	SPD15	Putative speedy protein E15
NRLLE	A6NNV3	SPD16	Putative speedy protein E16
NRLLE	P0DUD2	SPD17	Putative speedy protein E17
RKRRF	A0A494C086	SPD21	Putative speedy protein E21
NRLLE	A0A494C086	SPD21	Putative speedy protein E21
RKRRF	Q495Y7	SPDE7	Putative speedy protein E7
NRLLE	Q495Y7	SPDE7	Putative speedy protein E7

NRLLE	P0DUD1	SPD8	Putative speedy protein E8
NRLLE	A0A494C191	SPD9	Putative speedy protein E9
NRLLE	A6NJR5	SPDL3	Putative speedy protein-like protein 3
KKRKR	B4DYI2	S31C2	Putative spermatogenesis-associated protein 31C2
LGFLL	Q8IY50	S35F3	Putative thiamine transporter SLC35F3
SKKRK	Q5EBN2	TRI61	Putative tripartite motif-containing protein 61
KKRKR	Q5EBN2	TRI61	Putative tripartite motif-containing protein 61
GSVSF	A6NI03	TR64B	Putative tripartite motif-containing protein 64B
SVSFF	A6NI03	TR64B	Putative tripartite motif-containing protein 64B
ASGQN	P0C5K6	CT18	Putative tumor antigen NA88-A
ALLST	O75061	AUXI	Putative tyrosine-protein phosphatase auxilin
TNSEL	O75061	AUXI	Putative tyrosine-protein phosphatase auxilin
NSELL	O75061	AUXI	Putative tyrosine-protein phosphatase auxilin
SELLS	O75061	AUXI	Putative tyrosine-protein phosphatase auxilin
IIVIL	P56180	TPTE	Putative tyrosine-protein phosphatase TPTE
IVILL	P56180	TPTE	Putative tyrosine-protein phosphatase TPTE
SNICL	Q3LFD5	UBP41	Putative ubiquitin carboxyl-terminal hydrolase 41
SVLTS	A8MUL3	ADAS1	Putative uncharacterized protein ADARB2-AS1
NSELL	Q499Y3	YJ016	Putative uncharacterized protein C10orf88-like
TLPSE	Q8WZ69	CK040	Putative uncharacterized protein C11orf40
VKGEP	Q96LS8	CB048	Putative uncharacterized protein C2orf48
SVITI	A6NIL9	CRAS1	Putative uncharacterized protein CRYM-AS1
ELQLL	Q8NCQ3	NC301	Putative uncharacterized protein encoded by LINC00301

LLSLI	Q7Z4B0	CR020	Putative uncharacterized protein encoded by LINC00305 precursor
ALRTG	Q9NZY2	FA30A	Putative uncharacterized protein FAM30A
TLPSE	Q8NBF4	YG006	Putative uncharacterized protein FLJ33307
RGIIK	Q8N8P6	YX004	Putative uncharacterized protein FLJ39060
KSALL	Q6ZS92	YD022	Putative uncharacterized protein FLJ45721
REFSV	Q6ZRM9	YG024	Putative uncharacterized protein FLJ46235
DVSSS	Q6ZR54	YN009	Putative uncharacterized protein FLJ46641
TYMLT	P0DP75	M14OS	Putative uncharacterized protein MED14OS
STPPT	Q9BRP9	YK016	Putative uncharacterized protein MGC13053
AVVSL	Q9H6N6	MYH16	Putative uncharacterized protein MYH16
SALLS	Q6XCG6	YA011	Putative uncharacterized protein PP632
TFSNG	Q6UY13	YB003	Putative uncharacterized protein UNQ5830/PRO19650/PRO19816 precursor
GSVSF	B1APH4	ZN487	Putative zinc finger protein 487
ELPRF	Q5H9M0	PWP3B	PWWP domain-containing DNA repair factor 3B
VAVSK	O94903	PLPHP	Pyridoxal phosphate homeostasis protein
VSSSV	Q6P996	PDXD1	Pyridoxal-dependent decarboxylase domain-containing protein 1
NYIDK	Q08257	QOR	Quinone oxidoreductase
LLGVG	O95825	QORL1	Quinone oxidoreductase-like protein 1
TREFS	Q9Y3T6	R3HC1	R3H and coiled-coil domain-containing protein 1
REFSV	Q9Y3T6	R3HC1	R3H and coiled-coil domain-containing protein 1
TLPSE	P24386	RAE1	Rab proteins geranylgeranyltransferase component A 1
ELPRF	Q6WKZ4	RFIP1	Rab11 family-interacting protein 1

NSELL	Q86YS3	RFIP4	Rab11 family-interacting protein 4
YLSAL	Q9P260	RELCH	RAB11-binding protein RELCH
RELPR	Q9P260	RELCH	RAB11-binding protein RELCH
IIKTF	Q9P260	RELCH	RAB11-binding protein RELCH
SKVLH	Q15042	RB3GP	Rab3 GTPase-activating protein catalytic subunit
NSLTL	Q7Z6M1	RABEK	Rab9 effector protein with kelch motifs
VAVSK	P31751	AKT2	RAC-beta serine/threonine-protein kinase
SDELL	Q6NUQ1	RINT1	RAD50-interacting protein 1
DVSSS	P0C881	R10B1	Radial spoke head 10 homolog B
DVSSS	B2RC85	R10B2	Radial spoke head 10 homolog B2
EEVLA	Q9HA92	RSAD1	Radical S-adenosyl methionine domain-containing protein 1, mitochondrial precursor
GAIVS	Q2PPJ7	RGPA2	Ral GTPase-activating protein subunit alpha-2
ILLSL	Q86X10	RLGPB	Ral GTPase-activating protein subunit beta
ETCKV	Q12967	GNDS	Ral guanine nucleotide dissociation stimulator
SLTLP	Q9NZL6	RGL1	Ral guanine nucleotide dissociation stimulator-like 1
LGFLL	Q3MIN7	RGL3	Ral guanine nucleotide dissociation stimulator-like 3
LPRFM	Q8NFH8	REPS2	RalBP1-associated Eps domain-containing protein 2
LSKDQ	Q9H2T7	RBP17	Ran-binding protein 17
FLLGV	P0DJD0	RGPD1	RANBP2-like and GRIP domain-containing protein 1
KAVVS	P0DJD0	RGPD1	RANBP2-like and GRIP domain-containing protein 1
FLLGV	P0DJD1	RGPD2	RANBP2-like and GRIP domain-containing protein 2
KAVVS	P0DJD1	RGPD2	RANBP2-like and GRIP domain-containing protein 2

FLLGV	A6NKT7	RGPD3	RanBP2-like and GRIP domain-containing protein 3
KAVVS	A6NKT7	RGPD3	RanBP2-like and GRIP domain-containing protein 3
FLLGV	Q7Z3J3	RGPD4	RanBP2-like and GRIP domain-containing protein 4
KAVVS	Q7Z3J3	RGPD4	RanBP2-like and GRIP domain-containing protein 4
FLLGV	Q99666	RGPD5	RANBP2-like and GRIP domain-containing protein 5/6
KAVVS	Q99666	RGPD5	RANBP2-like and GRIP domain-containing protein 5/6
FLLGV	O14715	RGPD8	RANBP2-like and GRIP domain-containing protein 8
KAVVS	O14715	RGPD8	RANBP2-like and GRIP domain-containing protein 8
EITRE	O14715	RGPD8	RANBP2-like and GRIP domain-containing protein 8
DQKKL	Q13905	RPGF1	Rap guanine nucleotide exchange factor 1
LTNSE	O95398	RPGF3	Rap guanine nucleotide exchange factor 3
KVLHL	Q92565	RPGF5	Rap guanine nucleotide exchange factor 5
VSSSV	Q8TEU7	RPGF6	Rap guanine nucleotide exchange factor 6
LSALR	P52306	GDS1	Rap1 GTPase-GDP dissociation stimulator 1
ITTIL	Q6R327	RICTR	Rapamycin-insensitive companion of mTOR
NSLTL	Q6R327	RICTR	Rapamycin-insensitive companion of mTOR
SLTLP	Q6R327	RICTR	Rapamycin-insensitive companion of mTOR
SLIAV	Q6R327	RICTR	Rapamycin-insensitive companion of mTOR
LIAVG	Q6R327	RICTR	Rapamycin-insensitive companion of mTOR
LHTSP	Q8WYP3	RIN2	Ras and Rab interactor 2
NDQKK	Q15283	RASA2	Ras GTPase-activating protein 2
SAIAS	Q14644	RASA3	Ras GTPase-activating protein 3
LPIVN	Q14644	RASA3	Ras GTPase-activating protein 3

LSALR	O43374	RASL2	Ras GTPase-activating protein 4
LSALR	C9J798	RAS4B	Ras GTPase-activating protein 4B
LSALR	P46940	IQGA1	Ras GTPase-activating-like protein IQGAP1
LYGVI	P46940	IQGA1	Ras GTPase-activating-like protein IQGAP1
VSKVL	Q13576	IQGA2	Ras GTPase-activating-like protein IQGAP2
VLTSK	Q13576	IQGA2	Ras GTPase-activating-like protein IQGAP2
SKKRK	O95267	GRP1	RAS guanyl-releasing protein 1
KCTAS	Q96PV0	SYGP1	Ras/Rap GTPase-activating protein SynGAP
LSALR	O95294	RASL1	RasGAP-activating-like protein 1
FPQAE	O95294	RASL1	RasGAP-activating-like protein 1
EEVLA	Q0VAM2	RGF1B	Ras-GEF domain-containing family member 1B
ARREL	Q9NYN1	RASLC	Ras-like protein family member 12
KKRKR	P63000	RAC1	Ras-related C3 botulinum toxin substrate 1 precursor
SVGNT	Q5VZM2	RRAGB	Ras-related GTP-binding protein B
RLLEI	Q14964	RB39A	Ras-related protein Rab-39A
AIVSC	Q96S21	RB40C	Ras-related protein Rab-40C
SALLS	Q5JT25	RAB41	Ras-related protein Rab-41
ALLST	Q5JT25	RAB41	Ras-related protein Rab-41
SALLS	Q92766	RREB1	Ras-responsive element-binding protein 1
FASGQ	Q13972	RGRF1	Ras-specific guanine nucleotide-releasing factor 1
ASGQN	Q13972	RGRF1	Ras-specific guanine nucleotide-releasing factor 1
KGYLS	Q13972	RGRF1	Ras-specific guanine nucleotide-releasing factor 1
ELLSL	Q8TDY2	RBCC1	RB1-inducible coiled-coil protein 1

AVGLL	O95199	RCBT2	RCC1 and BTB domain-containing protein 2
AGVTT	Q9BX79	STRA6	Receptor for retinol uptake STRA6
DKQLL	P21860	ERBB3	Receptor tyrosine-protein kinase erbB-3 precursor
LLILK	Q15303	ERBB4	Receptor tyrosine-protein kinase erbB-4 precursor
SELLS	P57078	RIPK4	Receptor-interacting serine/threonine-protein kinase 4
KAVVS	P23469	PTPRE	Receptor-type tyrosine-protein phosphatase epsilon precursor
NKIKS	P23470	PTPRG	Receptor-type tyrosine-protein phosphatase gamma precursor
NQSLA	P23470	PTPRG	Receptor-type tyrosine-protein phosphatase gamma precursor
LLGVG	Q15262	PTPRK	Receptor-type tyrosine-protein phosphatase kappa precursor
VDIFN	O14522	PTPRT	Receptor-type tyrosine-protein phosphatase T precursor
LAFIR	Q92729	PTPRU	Receptor-type tyrosine-protein phosphatase U precursor
IIVII	Q92729	PTPRU	Receptor-type tyrosine-protein phosphatase U precursor
STPPT	P23471	PTPRZ	Receptor-type tyrosine-protein phosphatase zeta precursor
IIKEE	P23471	PTPRZ	Receptor-type tyrosine-protein phosphatase zeta precursor
CSAVS	Q16849	PTPRN	Receptor-type tyrosine-protein phosphatase-like N precursor
AVTFC	P78509	RELN	Reelin precursor
GKSLY	P78509	RELN	Reelin precursor
VQLPL	Q8N5W9	RFLB	Refilin-B
VSLSN	Q5VT52	RPRD2	Regulation of nuclear pre-mRNA domain-containing protein 2
SLTLP	Q5VT52	RPRD2	Regulation of nuclear pre-mRNA domain-containing protein 2
LTLPS	Q5VT52	RPRD2	Regulation of nuclear pre-mRNA domain-containing protein 2
EEVLA	O43566	RGS14	Regulator of G-protein signaling 14
PRFMN	O76081	RGS20	Regulator of G-protein signaling 20

ITTII	Q8NE09	RGS22	Regulator of G-protein signaling 22
LGFLL	P49798	RGS4	Regulator of G-protein signaling 4
RKRRF	Q6MZT1	R7BP	Regulator of G-protein signaling 7-binding protein
AVVSL	Q6ZWK4	RHEX	Regulator of hemoglobinization and erythroid cell expansion protein
KIMTS	Q96LZ7	RMD2	Regulator of microtubule dynamics protein 2
VILLS	Q96DM3	RMC1	Regulator of MON1-CCZ1 complex
IIKEE	Q96T23	RSF1	Remodeling and spacing factor 1
SVITS	Q96T23	RSF1	Remodeling and spacing factor 1
LKNYI	Q5VYX0	RNLS	Renalase precursor
ELLHN	P40938	RFC3	Replication factor C subunit 3
QEGKS	Q13156	RFA4	Replication protein A 30 kDa subunit
TCKVQ	Q96B86	RGMA	Repulsive guidance molecule A precursor
SIIKE	A6NLU0	RFPLA	Ret finger protein-like 4A
SIIKE	F8VTS6	RFAL1	Ret finger protein-like 4A-like protein 1
VSVLT	Q6ZWI9	RFPLB	Ret finger protein-like 4B
SCSIS	Q6ZWI9	RFPLB	Ret finger protein-like 4B
EVNKI	Q16799	RTN1	Reticulon-1
SALLS	O95197	RTN3	Reticulon-3
TLSKK	Q9NQC3	RTN4	Reticulon-4
TSKVL	Q9NQC3	RTN4	Reticulon-4
RRELP	Q02846	GUC2D	Retinal guanylyl cyclase 1 precursor
FLGFL	Q02846	GUC2D	Retinal guanylyl cyclase 1 precursor
LGFLL	Q02846	GUC2D	Retinal guanylyl cyclase 1 precursor

LEGEV	P28749	RBL1	Retinoblastoma-like protein 1
KRKRR	Q08999	RBL2	Retinoblastoma-like protein 2
TPVST	Q08999	RBL2	Retinoblastoma-like protein 2
FLLGV	Q8NFJ5	RAI3	Retinoic acid-induced protein 3
VLTSK	Q9HCM1	RESF1	Retroelement silencing factor 1
DQKKL	Q9HCM1	RESF1	Retroelement silencing factor 1
LTLPS	Q9HCM1	RESF1	Retroelement silencing factor 1
VSSSV	O95980	RECK	Reversion-inducing cysteine-rich protein with Kazal motifs precursor
QELDK	P52565	GDIR1	Rho GDP-dissociation inhibitor 1
SKKRK	Q6P4F7	RHGBA	Rho GTPase-activating protein 11A
KRKRR	Q6P4F7	RHGBA	Rho GTPase-activating protein 11A
SGINN	Q6P4F7	RHGBA	Rho GTPase-activating protein 11A
DASIS	Q8IWW6	RHG12	Rho GTPase-activating protein 12
ASGQN	Q68EM7	RHG17	Rho GTPase-activating protein 17
INQSL	Q9P2F6	RHG20	Rho GTPase-activating protein 20
LQLLM	Q9UNA1	RHG26	Rho GTPase-activating protein 26
RELPR	Q9P2N2	RHG28	Rho GTPase-activating protein 28
ALLST	Q9P2N2	RHG28	Rho GTPase-activating protein 28
TSKVL	Q9P2N2	RHG28	Rho GTPase-activating protein 28
LLGVG	Q7Z6I6	RHG30	Rho GTPase-activating protein 30
SLTLP	Q2M1Z3	RHG31	Rho GTPase-activating protein 31
GKSLY	A7KAX9	RHG32	Rho GTPase-activating protein 32

GFLLG	Q9NRY4	RHG35	Rho GTPase-activating protein 35
STPVT	Q9NRY4	RHG35	Rho GTPase-activating protein 35
EEVLA	Q9C0H5	RHG39	Rho GTPase-activating protein 39
SELLS	Q92619	HMHA1	Rho GTPase-activating protein 45
MSIIK	Q9HCE6	ARGAL	Rho guanine nucleotide exchange factor 10-like protein
KYDCK	Q9HCE6	ARGAL	Rho guanine nucleotide exchange factor 10-like protein
VKLIK	O15085	ARHGB	Rho guanine nucleotide exchange factor 11
LPLYG	O15085	ARHGB	Rho guanine nucleotide exchange factor 11
VKLIK	Q9NZN5	ARHGC	Rho guanine nucleotide exchange factor 12
LHLEG	Q6ZSZ5	ARHGI	Rho guanine nucleotide exchange factor 18
STTNI	Q92974	ARHG2	Rho guanine nucleotide exchange factor 2
SDELL	Q8N1W1	ARG28	Rho guanine nucleotide exchange factor 28
TLSKK	A1IGU5	ARH37	Rho guanine nucleotide exchange factor 37
SALLS	Q9NXL2	ARH38	Rho guanine nucleotide exchange factor 38
LSGIN	Q13009	TIAM1	Rho guanine nucleotide exchange factor TIAM1
REFSV	Q8IVF5	TIAM2	Rho guanine nucleotide exchange factor TIAM2
DELLH	Q8IVF5	TIAM2	Rho guanine nucleotide exchange factor TIAM2
GINNI	Q8TEB9	RHBL4	Rhomboid-related protein 4
NRARR	O94844	RHBT1	Rho-related BTB domain-containing protein 1
NRARR	Q9BYZ6	RHBT2	Rho-related BTB domain-containing protein 2
QNITE	Q96L33	RHOV	Rho-related GTP-binding protein RhoV
LPLYG	Q9BST9	RTKN	Rhotekin
LPLYG	Q8IZC4	RTKN2	Rhotekin-2

STTNI	P34096	RNAS4	Ribonuclease 4 precursor
SGINN	Q93091	RNAS6	Ribonuclease K6 precursor
TNVTL	Q99575	POP1	Ribonucleases P/MRP protein subunit POP1
NVTLS	Q99575	POP1	Ribonucleases P/MRP protein subunit POP1
KKRKR	Q99575	POP1	Ribonucleases P/MRP protein subunit POP1
GVAVS	P23921	RIR1	Ribonucleoside-diphosphate reductase large subunit
MELLI	P60891	PRPS1	Ribose-phosphate pyrophosphokinase 1
MELLI	P11908	PRPS2	Ribose-phosphate pyrophosphokinase 2
MELLI	P21108	PRPS3	Ribose-phosphate pyrophosphokinase 3
TLPSE	Q9H6W3	RIOX1	Ribosomal oxygenase 1
LILKA	Q15418	KS6A1	Ribosomal protein S6 kinase alpha-1
LILKA	Q15349	KS6A2	Ribosomal protein S6 kinase alpha-2
ETVIE	Q96S38	KS6C1	Ribosomal protein S6 kinase delta-1
KRKRR	Q8TDN6	BRX1	Ribosome biogenesis protein BRX1 homolog
VLTSK	Q9P2E9	RRBP1	Ribosome-binding protein 1
TPVST	O15034	RIMB2	RIMS-binding protein 2
GNTLY	Q96EX2	RNFT2	RING finger and transmembrane domain-containing protein 2
VNKIK	Q96MT1	RN145	RING finger protein 145
NKIKS	Q96MT1	RN145	RING finger protein 145
SVLTS	Q9BXT8	RNF17	RING finger protein 17
SVITS	Q8N4F7	RN175	RING finger protein 175
STPPT	Q8ND24	RN214	RING finger protein 214
VNAGV	Q9H2S5	RNF39	RING finger protein 39

VSKVL	Q9Y2P8	RCL1	RNA 3'-terminal phosphate cyclase-like protein
NQSLA	Q9Y2P8	RCL1	RNA 3'-terminal phosphate cyclase-like protein
TMNSL	Q9H0A0	NAT10	RNA cytidine acetyltransferase
LYYVN	Q9H0A0	NAT10	RNA cytidine acetyltransferase
SVSFF	Q6P6C2	ALKB5	RNA demethylase ALKBH5
ILLSL	O60306	AQR	RNA helicase aquarius
FLGFL	Q9BXT6	M10L1	RNA helicase Mov10l1
SLGAI	O00472	ELL2	RNA polymerase II elongation factor ELL2
KKRKR	Q8N7H5	PAF1	RNA polymerase II-associated factor 1 homolog
AVTEL	Q9H6T3	RPAP3	RNA polymerase II-associated protein 3
ELLSL	Q92541	RTF1	RNA polymerase-associated protein RTF1 homolog
VSKVL	Q9UJJ7	RUSD1	RNA pseudouridylate synthase domain-containing protein 1
KVKLI	Q9Y388	RBMX2	RNA-binding motif protein, X-linked 2
PVSTY	Q6XE24	RBMS3	RNA-binding motif, single-stranded-interacting protein 3
RRFLG	Q8IXT5	RB12B	RNA-binding protein 12B
TELQL	Q5T8P6	RBM26	RNA-binding protein 26
IKEEV	Q9P2N5	RBM27	RNA-binding protein 27
TLSKK	P42696	RBM34	RNA-binding protein 34
SKKRK	Q14498	RBM39	RNA-binding protein 39
ELLSL	Q9BTD8	RBM42	RNA-binding protein 42
SALLS	Q6ZP01	RBM44	RNA-binding protein 44
SNGCD	Q6ZP01	RBM44	RNA-binding protein 44
VFPSD	Q6ZP01	RBM44	RNA-binding protein 44

QQSYS	P35637	FUS	RNA-binding protein FUS
GIIKT	P35637	FUS	RNA-binding protein FUS
ALRTG	Q6ZN04	MEX3B	RNA-binding protein MEX3B
KSDEL	Q86SE5	RALYL	RNA-binding Raly-like protein
PIVNK	P16499	PDE6A	Rod cGMP-specific 3',5'-cyclic phosphodiesterase subunit alpha precursor
PIVNK	P35913	PDE6B	Rod cGMP-specific 3',5'-cyclic phosphodiesterase subunit beta precursor
ARREL	Q5TZA2	CROCC	Rootletin
KEEVL	Q86VV8	RTTN	Rotatin
DAKVK	Q6NSI4	RADX	RPA-related protein RADX
ALLST	Q6IN84	MRM1	rRNA methyltransferase 1, mitochondrial precursor
KKRKR	Q9BRU9	UTP23	rRNA-processing protein UTP23 homolog
TLSKK	Q13950	RUNX2	Runt-related transcription factor 2
SISNI	Q6NW29	RWDD4	RWD domain-containing protein 4
IVILL	P21817	RYR1	Ryanodine receptor 1
ELLIL	Q92736	RYR2	Ryanodine receptor 2
VSKGY	Q92736	RYR2	Ryanodine receptor 2
NVTLS	Q92736	RYR2	Ryanodine receptor 2
NGVSV	Q92736	RYR2	Ryanodine receptor 2
IVILL	Q92736	RYR2	Ryanodine receptor 2
ELLIL	Q15413	RYR3	Ryanodine receptor 3
IVILL	Q15413	RYR3	Ryanodine receptor 3

GSVSF	Q9BY12	SCAPE	S phase cyclin A-associated protein in the endoplasmic reticulum
LLSLI	Q9NZJ4	SACS	Sacsin
ILLSL	Q9NZJ4	SACS	Sacsin
LGAIV	O43865	SAHH2	S-adenosylhomocysteine hydrolase-like protein 1
TNVTL	Q9BXA9	SALL3	Sal-like protein 3
ALLST	Q9BXA9	SALL3	Sal-like protein 3
TNVTL	Q9UJQ4	SALL4	Sal-like protein 4
CSAVS	O94885	SASH1	SAM and SH3 domain-containing protein 1
KKRKR	P82979	SARNP	SAP domain-containing ribonucleoprotein
EGKSL	Q93084	AT2A3	Sarcoplasmic/endoplasmic reticulum calcium ATPase 3
ARSTP	P57086	SCND1	SCAN domain-containing protein 1
ELSNI	Q6R2W3	SCND3	SCAN domain-containing protein 3
VLTSK	Q6R2W3	SCND3	SCAN domain-containing protein 3
NAVTE	Q6ZMJ2	SCAR5	Scavenger receptor class A member 5
VSCYG	Q9NR16	C163B	Scavenger receptor cysteine-rich type 1 protein M160 precursor
LHLEG	Q5VUG0	SMBT2	Scm-like with four MBT domains protein 2
STCSA	A2VEC9	SSPO	SCO-spondin precursor
SALLS	A2VEC9	SSPO	SCO-spondin precursor
NGVSV	A2VEC9	SSPO	SCO-spondin precursor
ELLIL	Q8WU76	SCFD2	Sec1 family domain-containing protein 2
SSVIT	Q92503	S14L1	SEC14-like protein 1
GVSVL	Q12765	SCRN1	Secernin-1
SKDQL	P13521	SCG2	Secretogranin-2 precursor

KDQLS	P13521	SCG2	Secretogranin-2 precursor
KEGSN	Q13018	PLA2R	Secretory phospholipase A2 receptor precursor
KSALL	Q9BYH1	SE6L1	Seizure 6-like protein precursor
NAITT	Q93073	SBP2L	Selenocysteine insertion sequence-binding protein 2-like
SKVLD	Q93073	SBP2L	Selenocysteine insertion sequence-binding protein 2-like
AGKST	Q93073	SBP2L	Selenocysteine insertion sequence-binding protein 2-like
QEGKS	Q9BQE4	SELS	Selenoprotein S
VLHLE	Q9NS98	SEM3G	Semaphorin-3G precursor
KAVVS	Q9H3S1	SEM4A	Semaphorin-4A precursor
GNTLY	Q9H3S1	SEM4A	Semaphorin-4A precursor
IAVGL	Q13591	SEM5A	Semaphorin-5A precursor
SALRT	Q8NFY4	SEM6D	Semaphorin-6D precursor
NKQEG	P04279	SEMG1	Semenogelin-1 precursor
LHLEG	A6NMZ2	SNTAN	Sentan
IINFY	Q9P0U3	SENP1	Sentrin-specific protease 1
NVDIF	Q9H4L4	SENP3	Sentrin-specific protease 3
VDIFN	Q9H4L4	SENP3	Sentrin-specific protease 3
SIIKE	Q14674	ESPL1	Separin
VGNTL	Q14674	ESPL1	Separin
LYYVN	Q14674	ESPL1	Separin
RELPR	P35270	SPRE	Sepiapterin reductase
VIDTP	Q9UHD8	SEPT9	Septin-9
SSVIT	Q9NRX5	SERC1	Serine incorporator 1

GLLLY	Q96SA4	SERC2	Serine incorporator 2
TASNK	Q86VE9	SERC5	Serine incorporator 5
AVGLL	Q6UWY2	PRS57	Serine protease 57 precursor
LTAVT	O43464	HTRA2	Serine protease HTRA2, mitochondrial precursor
RGIIK	Q6IE38	ISK14	Serine protease inhibitor Kazal-type 14 precursor
SKKRK	Q9UQ35	SRRM2	Serine/arginine repetitive matrix protein 2
KKRKR	Q9UQ35	SRRM2	Serine/arginine repetitive matrix protein 2
KRKRR	Q9UQ35	SRRM2	Serine/arginine repetitive matrix protein 2
MQSTP	A6NNA2	SRRM3	Serine/arginine repetitive matrix protein 3
GYLSA	A7MD48	SRRM4	Serine/arginine repetitive matrix protein 4
TSKTD	O75494	SRS10	Serine/arginine-rich splicing factor 10
RLLEI	Q5VZP5	STYL2	Serine/threonine/tyrosine-interacting-like protein 2
LSALR	Q8N1F8	S11IP	Serine/threonine-protein kinase 11-interacting protein
ISQVN	O94768	ST17B	Serine/threonine-protein kinase 17B
VNAGV	P49842	STK19	Serine/threonine-protein kinase 19
NAKKT	Q9Y6E0	STK24	Serine/threonine-protein kinase 24
AGSVS	Q13188	STK3	Serine/threonine-protein kinase 3
AGSVS	Q13043	STK4	Serine/threonine-protein kinase 4
GKSTT	Q13535	ATR	Serine/threonine-protein kinase ATR
ARSTP	Q8TDC3	BRSK1	Serine/threonine-protein kinase BRSK1
SVLTS	Q15139	KPCD1	Serine/threonine-protein kinase D1
TSKVL	P11801	KPSH1	Serine/threonine-protein kinase H1
VLDLK	Q9UPZ9	CILK1	Serine/threonine-protein kinase ICK

EEVLA	Q96Q04	LMTK3	Serine/threonine-protein kinase LMTK3 precursor
AKVKL	Q9P0L2	MARK1	Serine/threonine-protein kinase MARK1
AKVKL	Q7KZI7	MARK2	Serine/threonine-protein kinase MARK2
IVILL	Q9Y5S2	MRCKB	Serine/threonine-protein kinase MRCK beta
IAVGL	Q9Y5S2	MRCKB	Serine/threonine-protein kinase MRCK beta
ASGQN	Q96PY6	NEK1	Serine/threonine-protein kinase Nek1
SISQV	P51957	NEK4	Serine/threonine-protein kinase Nek4
MELLI	Q8N165	PDK1L	Serine/threonine-protein kinase PDIK1L
ASGQN	Q8N165	PDK1L	Serine/threonine-protein kinase PDIK1L
KAVVS	O00444	PLK4	Serine/threonine-protein kinase PLK4
LILKA	P37023	ACVL1	Serine/threonine-protein kinase receptor R3 precursor
TKEGS	Q9BVS4	RIOK2	Serine/threonine-protein kinase RIO2
DELLH	Q9BVS4	RIOK2	Serine/threonine-protein kinase RIO2
LEGEV	Q96Q15	SMG1	Serine/threonine-protein kinase SMG1
YDPLV	Q96Q15	SMG1	Serine/threonine-protein kinase SMG1
TCKVQ	Q9H2K8	TAOK3	Serine/threonine-protein kinase TAO3
VITIE	Q59H18	TNI3K	Serine/threonine-protein kinase TNNI3K
LYCKA	Q6PHR2	ULK3	Serine/threonine-protein kinase ULK3
TPVST	Q9H4A3	WNK1	Serine/threonine-protein kinase WNK1
TLPSE	Q9H4A3	WNK1	Serine/threonine-protein kinase WNK1
VSNKG	Q9H4A3	WNK1	Serine/threonine-protein kinase WNK1
KDQLS	Q9Y3S1	WNK2	Serine/threonine-protein kinase WNK2
TDVSS	Q9BYP7	WNK3	Serine/threonine-protein kinase WNK3

ELLSL	Q76MJ5	ERN2	Serine/threonine-protein kinase/endoribonuclease IRE2 precursor
VNKIK	Q96QC0	PP1RA	Serine/threonine-protein phosphatase 1 regulatory subunit 10
LLSTN	P63151	2ABA	Serine/threonine-protein phosphatase 2A 55 kDa regulatory subunit B alpha isoform
LLSTN	Q00005	2ABB	Serine/threonine-protein phosphatase 2A 55 kDa regulatory subunit B beta isoform
LLSTN	Q66LE6	2ABD	Serine/threonine-protein phosphatase 2A 55 kDa regulatory subunit B delta isoform
LLSTN	Q9Y2T4	2ABG	Serine/threonine-protein phosphatase 2A 55 kDa regulatory subunit B gamma isoform
AFIRK	Q15172	2A5A	Serine/threonine-protein phosphatase 2A 56 kDa regulatory subunit alpha isoform
AFIRK	Q16537	2A5E	Serine/threonine-protein phosphatase 2A 56 kDa regulatory subunit epsilon isoform
LSALR	Q9Y5P8	P2R3B	Serine/threonine-protein phosphatase 2A regulatory subunit B" subunit beta
LLPIV	Q8TF05	PP4R1	Serine/threonine-protein phosphatase 4 regulatory subunit 1
DQKKL	P53041	PPP5	Serine/threonine-protein phosphatase 5
STPVT	O00743	PPP6	Serine/threonine-protein phosphatase 6 catalytic subunit
ELLIL	O15084	ANR28	Serine/threonine-protein phosphatase 6 regulatory ankyrin repeat subunit A
ELLIL	Q8N8A2	ANR44	Serine/threonine-protein phosphatase 6 regulatory ankyrin repeat subunit B

VSSSV	O75170	PP6R2	Serine/threonine-protein phosphatase 6 regulatory subunit 2
LSLIN	Q96HS1	PGAM5	Serine/threonine-protein phosphatase PGAM5, mitochondrial
EEFYQ	P49591	SYSC	Serine--tRNA ligase, cytoplasmic
EFYQS	P49591	SYSC	Serine--tRNA ligase, cytoplasmic
VDIFN	P29508	SPB3	Serpin B3
VSKVL	P48594	SPB4	Serpin B4
SKVLH	P48594	SPB4	Serpin B4
AKVKL	P36952	SPB5	Serpin B5
SVLTS	P02743	SAMP	Serum amyloid P-component precursor
AVGLL	Q15165	PON2	Serum paraoxonase/arylesterase 2
LLGVG	Q15166	PON3	Serum paraoxonase/lactonase 3
SKGYL	Q8IYR2	SMYD4	SET and MYND domain-containing protein 4
KKRKR	Q9Y6X0	SETBP	SET-binding protein
KRKRR	Q9Y6X0	SETBP	SET-binding protein
NGTDA	P04278	SHBG	Sex hormone-binding globulin precursor
ITSLG	Q9H788	SH24A	SH2 domain-containing protein 4A
ARREL	Q9Y566	SHAN1	SH3 and multiple ankyrin repeat domains protein 1
TLYYV	Q8TE82	S3TC1	SH3 domain and tetratricopeptide repeat-containing protein 1
VSLSN	Q9Y3L3	3BP1	SH3 domain-binding protein 1
RLLEI	Q9Y3L3	3BP1	SH3 domain-binding protein 1
LEGEV	P78314	3BP2	SH3 domain-binding protein 2
SCSIS	Q9P0V3	SH3B4	SH3 domain-binding protein 4
KSTTN	Q92529	SHC3	SHC-transforming protein 3

SEINL	Q9UL62	TRPC5	Short transient receptor potential channel 5
SKKRK	Q562F6	SGO2	Shugoshin 2
KQLLP	Q9NR45	SIAS	Sialic acid synthase
AGVTT	Q9Y286	SIGL7	Sialic acid-binding Ig-like lectin 7 precursor
VGLLL	Q9BZZ2	SN	Sialoadhesin precursor
YGKTK	Q5T5P2	SKT	Sickle tail protein homolog
IAVGL	Q8TD22	SFXN5	Sideroflexin-5
VGLLL	Q8TD22	SFXN5	Sideroflexin-5
LGFLL	Q8IUH8	SPP2C	Signal peptide peptidase-like 2C precursor
AVGLL	Q8IUH8	SPP2C	Signal peptide peptidase-like 2C precursor
LVFPS	Q8TCT6	SPPL3	Signal peptide peptidase-like 3
NSLTL	Q9Y5M8	SRPRB	Signal recognition particle receptor subunit beta
VQSNR	P42229	STA5A	Signal transducer and activator of transcription 5A
VQSNR	P51692	STA5B	Signal transducer and activator of transcription 5B
SALLS	P42226	STAT6	Signal transducer and activator of transcription 6
VVSLS	P25063	CD24	Signal transducer CD24 precursor
AVSKV	O43166	SI1L1	Signal-induced proliferation-associated 1-like protein 1
VSKVL	O43166	SI1L1	Signal-induced proliferation-associated 1-like protein 1
GVSVL	Q9P2F8	SI1L2	Signal-induced proliferation-associated 1-like protein 2
LYCKA	Q9P2F8	SI1L2	Signal-induced proliferation-associated 1-like protein 2
LYCKA	O60292	SI1L3	Signal-induced proliferation-associated 1-like protein 3
SISNI	O00241	SIRB1	Signal-regulatory protein beta-1 precursor
AFIRK	Q9NTI5	PDS5B	Sister chromatid cohesion protein PDS5 homolog B

DASIS	P12757	SKIL	Ski-like protein
SLAFI	Q96DU3	SLAF6	SLAM family member 6 precursor
NKIKS	Q96PX8	SLIK1	SLIT and NTRK-like protein 1 precursor
NRGII	O94991	SLIK5	SLIT and NTRK-like protein 5 precursor
LSALR	O75094	SLIT3	Slit homolog 3 protein precursor
VLHLE	O75094	SLIT3	Slit homolog 3 protein precursor
VGLLL	A0A1B0GVV1	SIM35	Small integral membrane protein 35
SNKNR	A0A5F9ZH02	SIM42	Small integral membrane protein 42
IKSAL	A6NGZ8	SMIM9	Small integral membrane protein 9 precursor
KAVVS	O75691	UTP20	Small subunit processome component 20 homolog
MSIIK	O75691	UTP20	Small subunit processome component 20 homolog
SKVLH	Q9BQI6	SLF1	SMC5-SMC6 complex localization factor protein 1
KVLHL	Q9BQI6	SLF1	SMC5-SMC6 complex localization factor protein 1
RARRE	Q9H2M3	BHMT2	S-methylmethionine--homocysteine S-methyltransferase BHMT2
AVVSL	Q5SXM2	SNPC4	snRNA-activating protein complex subunit 4
VSSSV	Q5SXM2	SNPC4	snRNA-activating protein complex subunit 4
SSSVI	Q5SXM2	SNPC4	snRNA-activating protein complex subunit 4
ELLIL	P48065	S6A12	Sodium- and chloride-dependent betaine transporter
IVILL	Q9NSD5	S6A13	Sodium- and chloride-dependent GABA transporter 2
ELLIL	P48066	S6A11	Sodium- and chloride-dependent GABA transporter 3
GFLLG	P48067	SC6A9	Sodium- and chloride-dependent glycine transporter 1
SALRT	P35498	SCN1A	Sodium channel protein type 1 subunit alpha
VILLS	Q9UI33	SCNBA	Sodium channel protein type 11 subunit alpha

SALRT	Q99250	SCN2A	Sodium channel protein type 2 subunit alpha
SALRT	Q9NY46	SCN3A	Sodium channel protein type 3 subunit alpha
SALRT	P35499	SCN4A	Sodium channel protein type 4 subunit alpha
SALRT	Q14524	SCN5A	Sodium channel protein type 5 subunit alpha
VSVLT	Q14524	SCN5A	Sodium channel protein type 5 subunit alpha
SVLTS	Q14524	SCN5A	Sodium channel protein type 5 subunit alpha
IIVIL	Q14524	SCN5A	Sodium channel protein type 5 subunit alpha
SALRT	Q9UQD0	SCN8A	Sodium channel protein type 8 subunit alpha
SALRT	Q15858	SCN9A	Sodium channel protein type 9 subunit alpha
PVTLS	Q8IWT1	SCN4B	Sodium channel subunit beta-4 precursor
QSCSI	Q8IZF0	NALCN	Sodium leak channel non-selective protein
ILLSL	Q8IZF0	NALCN	Sodium leak channel non-selective protein
GFLLG	Q14973	NTCP	Sodium/bile acid cotransporter
LLSLI	P57103	NAC3	Sodium/calcium exchanger 3 precursor
ILLSL	P57103	NAC3	Sodium/calcium exchanger 3 precursor
FLGFL	P19634	SL9A1	Sodium/hydrogen exchanger 1
IASGV	P19634	SL9A1	Sodium/hydrogen exchanger 1
VGLLL	Q4G0N8	SL9C1	Sodium/hydrogen exchanger 10
VSTYM	Q5TAH2	SL9C2	Sodium/hydrogen exchanger 11
STPPT	Q9UBY0	SL9A2	Sodium/hydrogen exchanger 2
VGLLL	Q9UBY0	SL9A2	Sodium/hydrogen exchanger 2
LSKDQ	Q9UBY0	SL9A2	Sodium/hydrogen exchanger 2
SLAFI	P48764	SL9A3	Sodium/hydrogen exchanger 3

LAFIR	P48764	SL9A3	Sodium/hydrogen exchanger 3
LTLPS	Q14940	SL9A5	Sodium/hydrogen exchanger 5
NVTLS	Q92581	SL9A6	Sodium/hydrogen exchanger 6
GAIVS	Q92581	SL9A6	Sodium/hydrogen exchanger 6
AVVSL	Q9Y2E8	SL9A8	Sodium/hydrogen exchanger 8
DQKKL	Q4ZJI4	SL9B1	Sodium/hydrogen exchanger 9B1
LLPIV	O60721	NCKX1	Sodium/potassium/calcium exchanger 1
VSSSV	O60721	NCKX1	Sodium/potassium/calcium exchanger 1
VSSSV	Q9UI40	NCKX2	Sodium/potassium/calcium exchanger 2
VGLLL	Q9HC58	NCKX3	Sodium/potassium/calcium exchanger 3 precursor
IIIVI	Q5VXU1	NKAI2	Sodium/potassium-transporting ATPase subunit beta-1-interacting protein 2
IIVIL	Q5VXU1	NKAI2	Sodium/potassium-transporting ATPase subunit beta-1-interacting protein 2
ILLSL	Q8N8D7	NKAI3	Sodium/potassium-transporting ATPase subunit beta-1-interacting protein 3
IIIVI	Q8IVV8	NKAI4	Sodium/potassium-transporting ATPase subunit beta-1-interacting protein 4
IIVIL	Q8IVV8	NKAI4	Sodium/potassium-transporting ATPase subunit beta-1-interacting protein 4
NQSLA	P54709	AT1B3	Sodium/potassium-transporting ATPase subunit beta-3
LTAVT	Q99624	S38A3	Sodium-coupled neutral amino acid transporter 3
LGFLL	Q99624	S38A3	Sodium-coupled neutral amino acid transporter 3

LIAVG	Q99624	S38A3	Sodium-coupled neutral amino acid transporter 3
IAVGL	Q99624	S38A3	Sodium-coupled neutral amino acid transporter 3
AVGLL	Q99624	S38A3	Sodium-coupled neutral amino acid transporter 3
ARSTP	Q99624	S38A3	Sodium-coupled neutral amino acid transporter 3
TSVIT	Q969I6	S38A4	Sodium-coupled neutral amino acid transporter 4
DELLH	Q969I6	S38A4	Sodium-coupled neutral amino acid transporter 4
VGLLL	Q8NBW4	S38A9	Sodium-coupled neutral amino acid transporter 9
TSLGA	Q5TF39	MFS4B	Sodium-dependent glucose transporter 1
NIKEN	Q14916	NPT1	Sodium-dependent phosphate transport protein 1
GFLLG	Q14916	NPT1	Sodium-dependent phosphate transport protein 1
SVITS	Q06495	NPT2A	Sodium-dependent phosphate transport protein 2A
VITIE	O95436	NPT2B	Sodium-dependent phosphate transport protein 2B
LGFLL	Q8N130	NPT2C	Sodium-dependent phosphate transport protein 2C
AGSVS	Q8WUM9	S20A1	Sodium-dependent phosphate transporter 1
VQLPL	Q6U841	S4A10	Sodium-driven chloride bicarbonate exchanger
LGFLL	Q86WA9	S2611	Sodium-independent sulfate anion transporter
IAVGL	Q3KNW5	SOAT	Solute carrier family 10 member 6
AVGLL	Q3KNW5	SOAT	Solute carrier family 10 member 6
LRTGW	A0AV02	S12A8	Solute carrier family 12 member 8
LLPIV	Q8WWT9	S13A3	Solute carrier family 13 member 3
SFFPQ	Q8WWT9	S13A3	Solute carrier family 13 member 3
FLGFL	Q9UKG4	S13A4	Solute carrier family 13 member 4
LGFLL	Q9UKG4	S13A4	Solute carrier family 13 member 4

LSLIA	Q16348	S15A2	Solute carrier family 15 member 2
ILLSL	Q8N697	S15A4	Solute carrier family 15 member 4
VILLS	Q9BYT1	S17A9	Solute carrier family 17 member 9
QLSGI	P11166	GTR1	Solute carrier family 2, facilitated glucose transporter member 1
LSGIN	P11166	GTR1	Solute carrier family 2, facilitated glucose transporter member 1
VNAGV	Q9BYW1	GTR11	Solute carrier family 2, facilitated glucose transporter member 11
INQSL	Q8TD20	GTR12	Solute carrier family 2, facilitated glucose transporter member 12
QLSGI	Q8TDB8	GTR14	Solute carrier family 2, facilitated glucose transporter member 14
LSGIN	Q8TDB8	GTR14	Solute carrier family 2, facilitated glucose transporter member 14
QLSGI	P11169	GTR3	Solute carrier family 2, facilitated glucose transporter member 3
LSGIN	P11169	GTR3	Solute carrier family 2, facilitated glucose transporter member 3
QLSGI	P14672	GLUT4	Solute carrier family 2, facilitated glucose transporter member 4
LSGIN	P14672	GLUT4	Solute carrier family 2, facilitated glucose transporter member 4
QLSGI	Q6PXP3	GTR7	Solute carrier family 2, facilitated glucose transporter member 7
LSGIN	Q6PXP3	GTR7	Solute carrier family 2, facilitated glucose transporter member 7
EEVLA	Q9NRM0	GTR9	Solute carrier family 2, facilitated glucose transporter member 9
AVGLL	Q9NRM0	GTR9	Solute carrier family 2, facilitated glucose transporter member 9
LYGVI	Q9NSA0	S22AB	Solute carrier family 22 member 11
ILLSL	Q9Y267	S22AE	Solute carrier family 22 member 14
FLLGV	Q86VW1	S22AG	Solute carrier family 22 member 16
SELLS	Q86VW1	S22AG	Solute carrier family 22 member 16
ELLSL	Q86VW1	S22AG	Solute carrier family 22 member 16
LGFLL	Q8WUG5	S22AH	Solute carrier family 22 member 17

AGVTT	A1A5C7	S22AN	Solute carrier family 22 member 23
TILTA	O76082	S22A5	Solute carrier family 22 member 5
AVSKV	Q9Y694	S22A7	Solute carrier family 22 member 7
GKSLY	Q96H78	S2544	Solute carrier family 25 member 44
LLILK	Q96AG3	S2546	Solute carrier family 25 member 46
LLSLI	Q96AG3	S2546	Solute carrier family 25 member 46
FDASI	Q9BXS9	S26A6	Solute carrier family 26 member 6
VILLS	Q5T1Q4	S35F1	Solute carrier family 35 member F1
LLGVG	Q8IXU6	S35F2	Solute carrier family 35 member F2
IVILL	Q8WV83	S35F5	Solute carrier family 35 member F5
LGFLL	Q8NBS3	S4A11	Solute carrier family 4 member 11
TSLGA	Q8NBS3	S4A11	Solute carrier family 4 member 11
AVVSL	Q8NBI5	S43A3	Solute carrier family 43 member 3
ILLSL	Q96JT2	S45A3	Solute carrier family 45 member 3
FLGFL	Q5BKX6	S45A4	Solute carrier family 45 member 4
KRRFL	Q7Z3Q1	S46A3	Solute carrier family 46 member 3 precursor
ASGVA	Q9NY91	SC5A4	Solute carrier family 5 member 4
LLGVG	Q9NQ40	S52A3	Solute carrier family 52, riboflavin transporter, member 3
GFLLG	Q9NYB5	SO1C1	Solute carrier organic anion transporter family member 1C1
SVITS	Q9NYB5	SO1C1	Solute carrier organic anion transporter family member 1C1
LLGVG	O94956	SO2B1	Solute carrier organic anion transporter family member 2B1
VSVLT	Q9UIG8	SO3A1	Solute carrier organic anion transporter family member 3A1
VITSL	Q96BD0	SO4A1	Solute carrier organic anion transporter family member 4A1

SSVIT	Q9H2Y9	SO5A1	Solute carrier organic anion transporter family member 5A1
YVVQL	P30872	SSR1	Somatostatin receptor type 1
LGFLL	P31391	SSR4	Somatostatin receptor type 4
YVVQL	P31391	SSR4	Somatostatin receptor type 4
LILKA	Q9NQZ2	SAS10	Something about silencing protein 10
EEVLA	Q9NQZ2	SAS10	Something about silencing protein 10
KRKRR	Q07889	SOS1	Son of sevenless homolog 1
ELLSL	Q07889	SOS1	Son of sevenless homolog 1
LLSLI	Q07889	SOS1	Son of sevenless homolog 1
KRKRR	Q07890	SOS2	Son of sevenless homolog 2
ELLSL	Q07890	SOS2	Son of sevenless homolog 2
RTDRG	Q15465	SHH	Sonic hedgehog protein precursor
DVSSS	Q92673	SORL	Sortilin-related receptor precursor
VSSSV	Q92673	SORL	Sortilin-related receptor precursor
ILLSL	Q92673	SORL	Sortilin-related receptor precursor
DIFNP	Q9UMY4	SNX12	Sorting nexin-12
SELLS	O60749	SNX2	Sorting nexin-2
IIKEE	Q5VWJ9	SNX30	Sorting nexin-30
KRKRR	Q8WV41	SNX33	Sorting nexin-33
KKRKR	Q9HB58	SP110	Sp110 nuclear body protein
ELLIL	Q96JI7	SPTCS	Spatacsin
VLTSK	Q96JI7	SPTCS	Spatacsin
KYDCK	Q96JI7	SPTCS	Spatacsin

IKEEV	Q9NUQ6	SPS2L	SPATS2-like protein
VSLSN	Q9H6E5	STPAP	Speckle targeted PIP5K1A-regulated poly(A) polymerase
AGSVS	Q13813	SPTN1	Spectrin alpha chain, non-erythrocytic 1
QEGKS	Q01082	SPTB2	Spectrin beta chain, non-erythrocytic 1
ALLST	Q9NRC6	SPTN5	Spectrin beta chain, non-erythrocytic 5
RKRRF	Q8NFV5	SPDE1	Speedy protein E1
NRLLE	Q8NFV5	SPDE1	Speedy protein E1
NRLLE	P0DUX0	SPD10	Speedy protein E10
NRLLE	P0DUX1	SPD12	Speedy protein E12
RKRRF	Q495Y8	SPDE2	Speedy protein E2
NRLLE	Q495Y8	SPDE2	Speedy protein E2
RKRRF	A6NHP3	SPE2B	Speedy protein E2B
NRLLE	A6NHP3	SPE2B	Speedy protein E2B
NRLLE	A6NKU9	SPDE3	Speedy protein E3
RKRRF	A6NIY4	SPDE5	Speedy protein E5
NRLLE	A6NIY4	SPDE5	Speedy protein E5
RKRRF	P0CI01	SPDE6	Speedy protein E6
NRLLE	P0CI01	SPDE6	Speedy protein E6
HTSPL	Q9HBV2	SACA1	Sperm acrosome membrane-associated protein 1 precursor
IETVI	Q6UW49	SPESP	Sperm equatorial segment protein 1 precursor
SKVLD	Q07617	SPAG1	Sperm-associated antigen 1
LLPIV	Q8N0X2	SPG16	Sperm-associated antigen 16 protein
TSVIT	Q6Q759	SPG17	Sperm-associated antigen 17

ILTAV	Q96R06	SPAG5	Sperm-associated antigen 5
VSSSV	Q8TB22	SPT20	Spermatogenesis-associated protein 20 precursor
RELPR	Q8NHS9	SPT22	Spermatogenesis-associated protein 22
IKTFS	Q8IUW3	SPA2L	Spermatogenesis-associated protein 2-like protein
LPSEI	Q6ZQQ2	S31D1	Spermatogenesis-associated protein 31D1
PSEIN	Q6ZQQ2	S31D1	Spermatogenesis-associated protein 31D1
PSEIN	P0C874	S31D3	Spermatogenesis-associated protein 31D3
PSEIN	Q6ZUB0	S31D4	Spermatogenesis-associated protein 31D4
KNRGI	Q6ZUB1	S31E1	Spermatogenesis-associated protein 31E1
SELLS	Q8NEY3	SPAT4	Spermatogenesis-associated protein 4
TSVIT	Q9NWH7	SPAT6	Spermatogenesis-associated protein 6 precursor
ASISQ	Q86VE3	SATL1	Spermidine/spermine N(1)-acetyltransferase-like protein 1
SELLS	Q13309	SKP2	S-phase kinase-associated protein 2
AVGLL	O60906	NSMA	Sphingomyelin phosphodiesterase 2
FLGFL	Q9NY59	NSMA2	Sphingomyelin phosphodiesterase 3
AKVKL	O95136	S1PR2	Sphingosine 1-phosphate receptor 2
VQLPL	Q8N0Z3	SPICE	Spindle and centriole-associated protein 1
VNEKI	Q96BD8	SKA1	Spindle and kinetochore-associated protein 1
KQELD	Q6UVJ0	SAS6	Spindle assembly abnormal protein 6 homolog
QELDK	Q6UVJ0	SAS6	Spindle assembly abnormal protein 6 homolog
SKKRK	Q15637	SF01	Splicing factor 1
KKRKR	Q15637	SF01	Splicing factor 1
KSALL	Q12874	SF3A3	Splicing factor 3A subunit 3

KRKRR	O75533	SF3B1	Splicing factor 3B subunit 1
RTDRG	O75533	SF3B1	Splicing factor 3B subunit 1
DTVSV	Q13435	SF3B2	Splicing factor 3B subunit 2
DEFDA	Q15393	SF3B3	Splicing factor 3B subunit 3
TPVTL	P26368	U2AF2	Splicing factor U2AF 65 kDa subunit
STPPT	Q9H7N4	SFR19	Splicing factor, arginine/serine-rich 19
GVGSA	P23246	SFPQ	Splicing factor, proline- and glutamine-rich
SLGAI	Q8WXA9	SREK1	Splicing regulatory glutamine/lysine-rich protein 1
IAVGL	Q8NCJ5	SPRY3	SPRY domain-containing protein 3
VGLLL	Q8WW59	SPRY4	SPRY domain-containing protein 4
NRLLE	Q9C0H9	SRCN1	SRC kinase signaling inhibitor 1
EVLAY	Q8WWQ8	STAB2	Stabilin-2 precursor
TPVTL	Q8WWQ8	STAB2	Stabilin-2 precursor
VVQLP	P52823	STC1	Stanniocalcin-1 precursor
RGIIK	Q7KZF4	SND1	Staphylococcal nuclease domain-containing protein 1
LLPIV	Q14849	STAR3	StAR-related lipid transfer protein 3
SELLS	Q14849	STAR3	StAR-related lipid transfer protein 3
NGVSV	Q9NSY2	STAR5	StAR-related lipid transfer protein 5
NITEE	Q9P2P6	STAR9	StAR-related lipid transfer protein 9
PTNNR	Q9P2P6	STAR9	StAR-related lipid transfer protein 9
NIMIT	Q9H2G2	SLK	STE20-like serine/threonine-protein kinase
LTRTD	Q8N6K7	SAMD3	Sterile alpha motif domain-containing protein 3
LKNYI	Q5K651	SAMD9	Sterile alpha motif domain-containing protein 9

SSSVI	Q8IVG5	SAM9L	Sterile alpha motif domain-containing protein 9-like
SELLS	P05093	CP17A	Steroid 17-alpha-hydroxylase/17,20 lyase
TLSKD	Q8N4F4	S22AO	Steroid transmembrane transporter SLC22A24
GTDAK	Q12772	SRBP2	Sterol regulatory element-binding protein 2
DAKVK	Q12772	SRBP2	Sterol regulatory element-binding protein 2
FLGFL	P08842	STS	Steryl-sulfatase precursor
SPLCT	P08842	STS	Steryl-sulfatase precursor
FLGFL	Q9UBI4	STML1	Stomatin-like protein 1
LGFLL	Q9UBI4	STML1	Stomatin-like protein 1
KSALL	Q15772	SPEG	Striated muscle preferentially expressed protein kinase
VKGEP	Q15772	SPEG	Striated muscle preferentially expressed protein kinase
KGEPI	Q15772	SPEG	Striated muscle preferentially expressed protein kinase
KQELD	A6NHR9	SMHD1	Structural maintenance of chromosomes flexible hinge domain-containing protein 1
KDQLS	A6NHR9	SMHD1	Structural maintenance of chromosomes flexible hinge domain-containing protein 1
VSVLT	Q96SB8	SMC6	Structural maintenance of chromosomes protein 6
ILLSL	Q9NX18	SDHF2	Succinate dehydrogenase assembly factor 2, mitochondrial precursor
KKLMS	Q96I99	SUCB2	Succinate--CoA ligase [GDP-forming] subunit beta, mitochondrial precursor
AVSKG	P51649	SSDH	Succinate-semialdehyde dehydrogenase, mitochondrial precursor
KTFSN	Q8NCC5	SPX3	Sugar phosphate exchanger 3

SALLS	P50443	S26A2	Sulfate transporter
ALLST	P50443	S26A2	Sulfate transporter
TSLGA	Q9Y6N5	SQOR	Sulfide:quinone oxidoreductase, mitochondrial precursor
SLGAI	Q9Y6N5	SQOR	Sulfide:quinone oxidoreductase, mitochondrial precursor
TSLGA	Q6IMI4	ST6B1	Sulfotransferase 6B1
TPVST	Q5W0Q7	USPL1	SUMO-specific isopeptidase USPL1
TPVST	Q9UBS9	SUCO	SUN domain-containing ossification factor precursor
LSALR	Q9UH99	SUN2	SUN domain-containing protein 2
SKKRK	O95425	SVIL	Supervillin
TTPVS	O95425	SVIL	Supervillin
RELPR	Q86UD0	SAPC2	Suppressor APC domain-containing protein 2
IASGV	Q86UD0	SAPC2	Suppressor APC domain-containing protein 2
SCYGK	O14544	SOCS6	Suppressor of cytokine signaling 6
KKRKR	O75683	SURF6	Surfeit locus protein 6
LDLKN	Q8IX01	SUGP2	SURP and G-patch domain-containing protein 2
ITIEL	Q9UGT4	SUSD2	Sushi domain-containing protein 2 precursor
AGVTT	Q96L08	SUSD3	Sushi domain-containing protein 3
GVTTP	Q96L08	SUSD3	Sushi domain-containing protein 3
KKRKR	Q8TAQ2	SMRC2	SWI/SNF complex subunit SMARCC2
NNRLL	Q9H4L7	SMRCD	SWI/SNF-related matrix-associated actin-dependent regulator of chromatin subfamily A containing DEAD/H box 1
KEEVL	Q9H4L7	SMRCD	SWI/SNF-related matrix-associated actin-dependent regulator of chromatin subfamily A containing DEAD/H box 1

SGQNI	Q9NZC9	SMAL1	SWI/SNF-related matrix-associated actin-dependent regulator of chromatin subfamily A-like protein 1
VILLS	Q9NZC9	SMAL1	SWI/SNF-related matrix-associated actin-dependent regulator of chromatin subfamily A-like protein 1
NDQKK	Q96GM5	SMRD1	SWI/SNF-related matrix-associated actin-dependent regulator of chromatin subfamily D member 1
NDQKK	Q92925	SMRD2	SWI/SNF-related matrix-associated actin-dependent regulator of chromatin subfamily D member 2
TNKAV	Q9H7V2	SYNG1	Synapse differentiation-inducing gene protein 1
SNKGM	Q7L0J3	SV2A	Synaptic vesicle glycoprotein 2A
VNKIK	Q496J9	SV2C	Synaptic vesicle glycoprotein 2C
SKVLD	O43426	SYNJ1	Synaptojanin-1
DASIS	O15056	SYNJ2	Synaptojanin-2
IELSN	Q15431	SYCP1	Synaptonemal complex protein 1
KKRKR	Q5T4T6	SYC2L	Synaptonemal complex protein 2-like
ELLSL	Q9H987	SYP2L	Synaptopodin 2-like protein
FFPQA	Q9H987	SYP2L	Synaptopodin 2-like protein
ELLSL	Q9UMS6	SYNP2	Synaptopodin-2
ILLSL	Q8IV01	SYT12	Synaptotagmin-12
TTIII	O43581	SYT7	Synaptotagmin-7
LGFLL	Q4VX76	SYTL3	Synaptotagmin-like protein 3
EGKSL	Q9Y6H5	SNCAP	Synphilin-1
LLPIV	Q9NX95	SYBU	Syntabulin

QNITE	Q5T5C0	STXB5	Syntaxin-binding protein 5
GAIVS	A0A0B4J271	TVAL3	T cell receptor alpha variable 12-3 precursor
NTKEG	A0A0B4J272	TVA24	T cell receptor alpha variable 24 precursor
PVTLS	A0A087WT02	TVA92	T cell receptor alpha variable 9-2 precursor
TPVTL	A0A0K0K1G6	TVBJ3	T cell receptor beta variable 10-3 precursor
NVTLS	A0A075B6N1	TVB19	T cell receptor beta variable 19 precursor
VNAGV	A0A0K0K1D8	TVB61	T cell receptor beta variable 6-1 precursor
NAGVT	A0A0K0K1D8	TVB61	T cell receptor beta variable 6-1 precursor
VNAGV	A0A0J9YXY3	TVB62	T cell receptor beta variable 6-2 precursor
NAGVT	A0A0J9YXY3	TVB62	T cell receptor beta variable 6-2 precursor
VNAGV	P0DPF7	TVB63	T cell receptor beta variable 6-3 precursor
NAGVT	P0DPF7	TVB63	T cell receptor beta variable 6-3 precursor
VNAGV	A0A0K0K1A5	TVB65	T cell receptor beta variable 6-5 precursor
NAGVT	A0A0K0K1A5	TVB65	T cell receptor beta variable 6-5 precursor
VNAGV	A0A0A6YYG2	TVB66	T cell receptor beta variable 6-6 precursor
NAGVT	A0A0A6YYG2	TVB66	T cell receptor beta variable 6-6 precursor
VNAGV	A0A0A6YYG3	TVB68	T cell receptor beta variable 6-8 precursor
NAGVT	A0A0A6YYG3	TVB68	T cell receptor beta variable 6-8 precursor
VNAGV	A0A0J9YX75	TVB69	T cell receptor beta variable 6-9 precursor
NAGVT	A0A0J9YX75	TVB69	T cell receptor beta variable 6-9 precursor
ILLSL	Q9Y6J9	TAF6L	TAF6-like RNA polymerase II p300/CBP-associated factor-associated factor 65 kDa subunit 6L
AITTI	Q9Y4G6	TLN2	Talin-2

IKQEL	Q9Y4G6	TLN2	Talin-2
AVTEL	Q9Y4G6	TLN2	Talin-2
KEGSN	Q8N9U0	TAC2N	Tandem C2 domains nuclear protein
LSALR	O15533	TPSN	Tapasin precursor
SLTLP	Q9BX59	TPSNR	Tapasin-related protein precursor
SNNVQ	Q8TE23	TS1R2	Taste receptor type 1 member 2 precursor
SALLS	Q9NYW7	TA2R1	Taste receptor type 2 member 1
ITSLG	P59551	T2R60	Taste receptor type 2 member 60
LSKKR	Q9H5J8	TAF1D	TATA box-binding protein-associated factor RNA polymerase I subunit D
AVVSL	O14981	BTAF1	TATA-binding protein-associated factor 172
QQSYS	Q92804	RBP56	TATA-binding protein-associated factor 2N
GIIKT	Q92804	RBP56	TATA-binding protein-associated factor 2N
ALLST	Q5TCY1	TTBK1	Tau-tubulin kinase 1
TNVTL	Q9P2M4	TBC14	TBC1 domain family member 14
NVTLS	Q9P2M4	TBC14	TBC1 domain family member 14
IAFSN	Q9P2M4	TBC14	TBC1 domain family member 14
VLHLE	Q9BYX2	TBD2A	TBC1 domain family member 2A
LSALR	Q9UPU7	TBD2B	TBC1 domain family member 2B
LILKA	Q92609	TBCD5	TBC1 domain family member 5
SNIKE	Q92609	TBCD5	TBC1 domain family member 5
VNKQS	Q66K14	TBC9B	TBC1 domain family member 9B
LTLPS	Q13207	TBX2	T-box transcription factor TBX2

SAIAS	Q9UMR3	TBX20	T-box transcription factor TBX20
LLGVG	Q8TB96	TIP	T-cell immunomodulatory protein precursor
GAIVS	P29016	CD1B	T-cell surface glycoprotein CD1b precursor
CNGTD	P04234	CD3D	T-cell surface glycoprotein CD3 delta chain precursor
VNKIK	Q99832	TCPH	T-complex protein 1 subunit eta
CDNAG	Q99832	TCPH	T-complex protein 1 subunit eta
EITRE	P49368	TCPG	T-complex protein 1 subunit gamma
ILLSL	Q8WWU5	TCP11	T-complex protein 11 homolog
LIKQE	Q9NUJ3	T11L1	T-complex protein 11-like protein 1
VSKVL	Q6ZSZ6	TSH1	Teashirt homolog 1
TAVTF	Q2MV58	TECT1	Tectonic-1 precursor
TKEGS	O15040	TCPR2	Tectonin beta-propeller repeat-containing protein 2
LLSTN	Q9UIF3	TEKT2	Tektin-2
KSALL	Q6NXR4	TTI2	TELO2-interacting protein 2
VLTSK	Q9BQ61	TRIR	Telomerase RNA component interacting RNase
ILTAV	Q86US8	EST1A	Telomerase-binding protein EST1A
NRLLE	Q86US8	EST1A	Telomerase-binding protein EST1A
TELQL	P10074	TZAP	Telomere zinc finger-associated protein
STTNI	Q15554	TERF2	Telomeric repeat-binding factor 2
NRLLE	P24821	TENA	Tenascin precursor
LHLEG	P22105	TENX	Tenascin-X precursor
VITSL	P22105	TENX	Tenascin-X precursor
TAVTF	Q9NT68	TEN2	Teneurin-2

LDLKN	Q9NT68	TEN2	Teneurin-2
DLKNY	Q9NT68	TEN2	Teneurin-2
GVSVL	Q9P273	TEN3	Teneurin-3
FLGFL	Q6N022	TEN4	Teneurin-4
SVITI	Q9BSI4	TINF2	TERF1-interacting nuclear factor 2
SSVIT	Q9BSI4	TINF2	TERF1-interacting nuclear factor 2
TCKVQ	Q5VYS8	TUT7	Terminal uridylyltransferase 7
CFASG	Q9Y4I5	MTL5	Tesmin
PSEIN	Q08629	TICN1	Testican-1 precursor
ELSNI	Q9BQ16	TICN3	Testican-3 precursor
SNIKE	Q9BZQ2	SHP1L	Testicular spindle-associated protein SHCBP1L
PTNNR	Q9NXF1	TEX10	Testis-expressed protein 10
KKRKR	Q9NXF1	TEX10	Testis-expressed protein 10
LGAIV	Q9BXU2	TX13B	Testis-expressed protein 13B
HLEGE	Q9BXT5	TEX15	Testis-expressed protein 15
ETCKV	Q9BXT5	TEX15	Testis-expressed protein 15
IKTFS	Q9BXT5	TEX15	Testis-expressed protein 15
TLSKD	Q9BXT5	TEX15	Testis-expressed protein 15
DKQLL	Q8IWB9	TEX2	Testis-expressed protein 2
KQLLP	Q8IWB9	TEX2	Testis-expressed protein 2
SCSIS	Q9Y6I9	TX264	Testis-expressed protein 264
SALRT	Q53QW1	TEX44	Testis-expressed protein 44
AGSVS	Q53QW1	TEX44	Testis-expressed protein 44

VSSSV	Q53QW1	TEX44	Testis-expressed protein 44
KRKRR	A6NCN8	TEX52	Testis-expressed protein 52
KDQLS	Q96M34	TEX55	Testis-specific expressed protein 55
VVSLS	Q9H0U9	TSYL1	Testis-specific Y-encoded-like protein 1
TCSAV	Q9H2G4	TSYL2	Testis-specific Y-encoded-like protein 2
VVSLS	Q9UJ04	TSYL4	Testis-specific Y-encoded-like protein 4
IKENK	O14817	TSN4	Tetraspanin-4
KENKC	O14817	TSN4	Tetraspanin-4
LIAVG	P19075	TSN8	Tetraspanin-8
IKENK	O75954	TSN9	Tetraspanin-9
KENKC	O75954	TSN9	Tetraspanin-9
LLGVG	O75954	TSN9	Tetraspanin-9
GLLLY	O75954	TSN9	Tetraspanin-9
TAVTF	Q8NEE8	TTC16	Tetratricopeptide repeat protein 16
SALLS	Q96AE7	TTC17	Tetratricopeptide repeat protein 17
LAFIR	Q6DKK2	TTC19	Tetratricopeptide repeat protein 19, mitochondrial precursor
ELQLL	Q5TAA0	TTC22	Tetratricopeptide repeat protein 22
IIKEE	Q5W5X9	TTC23	Tetratricopeptide repeat protein 23
VTELQ	Q6P3X3	TTC27	Tetratricopeptide repeat protein 27
FLLGV	Q6P3X3	TTC27	Tetratricopeptide repeat protein 27
SVGNT	Q86WT1	TT30A	Tetratricopeptide repeat protein 30A
VGNTL	Q86WT1	TT30A	Tetratricopeptide repeat protein 30A
LLLYC	Q86WT1	TT30A	Tetratricopeptide repeat protein 30A

LLYCK	Q86WT1	TT30A	Tetratricopeptide repeat protein 30A
SVGNT	Q8N4P2	TT30B	Tetratricopeptide repeat protein 30B
VGNTL	Q8N4P2	TT30B	Tetratricopeptide repeat protein 30B
LLLYC	Q8N4P2	TT30B	Tetratricopeptide repeat protein 30B
LLYCK	Q8N4P2	TT30B	Tetratricopeptide repeat protein 30B
KVLDL	Q5I0X7	TTC32	Tetratricopeptide repeat protein 32
GVGSA	Q15750	TAB1	TGF-beta-activated kinase 1 and MAP3K7-binding protein 1
RRFLG	P52888	THOP1	Thimet oligopeptidase
SALLS	Q9Y320	TMX2	Thioredoxin-related transmembrane protein 2 precursor
EEVLA	Q96FV9	THOC1	THO complex subunit 1
LLILK	Q8NI27	THOC2	THO complex subunit 2
AVVSL	Q8NI27	THOC2	THO complex subunit 2
SKKRK	Q13769	THOC5	THO complex subunit 5 homolog
EGEVN	Q13769	THOC5	THO complex subunit 5 homolog
LDLKN	P40238	TPOR	Thrombopoietin receptor precursor
SVITS	Q9NS62	THSD1	Thrombospondin type-1 domain-containing protein 1 precursor
QSTPP	Q9C0I4	THS7B	Thrombospondin type-1 domain-containing protein 7B precursor
LDLKN	Q9C0I4	THS7B	Thrombospondin type-1 domain-containing protein 7B precursor
SISNI	P35442	TSP2	Thrombospondin-2 precursor
SALLS	Q8WU66	TSEAR	Thrombospondin-type laminin G domain and EAR repeat-containing protein precursor
LLGVG	P21731	TA2R	Thromboxane A2 receptor
KEEVL	Q9BV44	THUM3	THUMP domain-containing protein 3

LGFLL	Q9BY10	TSCOT	Thymic stromal cotransporter homolog
AGKST	P23919	KTHY	Thymidylate kinase
ELDKY	Q15643	TRIPB	Thyroid receptor-interacting protein 11
QKKLM	Q15643	TRIPB	Thyroid receptor-interacting protein 11
KKLMS	Q15643	TRIPB	Thyroid receptor-interacting protein 11
KDQLS	Q15643	TRIPB	Thyroid receptor-interacting protein 11
RRFLG	Q9UKU6	TRHDE	Thyrotropin-releasing hormone-degrading ectoenzyme
IIKEE	Q6NT04	TIGD7	Tigger transposable element-derived protein 7
QSLAF	Q9UDY2	ZO2	Tight junction protein ZO-2
TLSKD	O95049	ZO3	Tight junction protein ZO-3
NAITT	Q8WZ42	TITIN	Titin
TCSAV	Q8WZ42	TITIN	Titin
RRELP	Q8WZ42	TITIN	Titin
GSAIA	Q8WZ42	TITIN	Titin
SALLS	Q8WZ42	TITIN	Titin
VLDLK	Q8WZ42	TITIN	Titin
ETVIE	Q8WZ42	TITIN	Titin
LTNSE	Q8WZ42	TITIN	Titin
PLYGV	Q8WZ42	TITIN	Titin
KLHTS	Q8WZ42	TITIN	Titin
AGSVS	Q8WZ42	TITIN	Titin
CTASN	Q8WZ42	TITIN	Titin
EGKSL	Q8WZ42	TITIN	Titin

DASIS	Q8WZ42	TITIN	Titin
ISQVN	Q8WZ42	TITIN	Titin
DELLH	Q8WZ42	TITIN	Titin
LSGIN	Q8WZ42	TITIN	Titin
IMSII	A6NGC4	TLCD2	TLC domain-containing protein 2
AIASG	Q96MV1	TLCD4	TLC domain-containing protein 4
CTASN	Q9HBG7	LY9	T-lymphocyte surface antigen Ly-9 precursor
AVVSL	Q9Y4F4	TGRM1	TOG array regulator of axonemal microtubules protein 1
HTSPL	Q9Y4F4	TGRM1	TOG array regulator of axonemal microtubules protein 1
SVGNT	Q9Y4F4	TGRM1	TOG array regulator of axonemal microtubules protein 1
SLGAI	Q6ZUX3	TGRM2	TOG array regulator of axonemal microtubules protein 2
LGAIV	Q6ZUX3	TGRM2	TOG array regulator of axonemal microtubules protein 2
GAIVS	P58753	TIRAP	Toll/interleukin-1 receptor domain-containing adapter protein
ELSNI	Q15399	TLR1	Toll-like receptor 1 precursor
LSNIK	Q15399	TLR1	Toll-like receptor 1 precursor
NKIKS	Q15399	TLR1	Toll-like receptor 1 precursor
KVLDL	Q15399	TLR1	Toll-like receptor 1 precursor
NAITT	O60603	TLR2	Toll-like receptor 2 precursor
GVSVL	O00206	TLR4	Toll-like receptor 4 precursor
IMTSK	O00206	TLR4	Toll-like receptor 4 precursor
NKIKS	Q9Y2C9	TLR6	Toll-like receptor 6 precursor
KVLDL	Q9Y2C9	TLR6	Toll-like receptor 6 precursor
KVLDL	Q9NR97	TLR8	Toll-like receptor 8 precursor

ELLSL	Q9NR97	TLR8	Toll-like receptor 8 precursor
GVGSA	Q96HA7	TONSL	Tonsoku-like protein
LTRTD	Q9H496	IFG15	Torsin-1A-interacting protein 2, isoform IFRG15
KRKRR	Q12888	TP53B	TP53-binding protein 1
SISQV	Q12888	TP53B	TP53-binding protein 1
ILLSL	O15050	TRNK1	TPR and ankyrin repeat-containing protein 1
QLSGI	O15050	TRNK1	TPR and ankyrin repeat-containing protein 1
SLYVK	O14804	TAAR5	Trace amine-associated receptor 5
FCFAS	Q96RI9	TAAR9	Trace amine-associated receptor 9
LSALR	Q9UKE5	TNIK	TRAF2 and NCK-interacting protein kinase
SGINN	Q8TDR0	MIPT3	TRAF3-interacting protein 1
KSDEL	O60296	TRAK2	Trafficking kinesin-binding protein 2
ELLHN	P48553	TPC10	Trafficking protein particle complex subunit 10
ALRTG	Q8WVR3	TPC14	Trafficking protein particle complex subunit 14
KSALL	Q8IUR0	TPPC5	Trafficking protein particle complex subunit 5
KYKNA	Q96Q05	TPPC9	Trafficking protein particle complex subunit 9
TSKVL	Q96Q05	TPPC9	Trafficking protein particle complex subunit 9
VGLLL	P20061	TCO1	Transcobalamin-1 precursor
FLLGV	P20062	TCO2	Transcobalamin-2 precursor
LSNIK	Q99990	VGLL1	Transcription cofactor vestigial-like protein 1
ALRTG	P23193	TCEA1	Transcription elongation factor A protein 1
KKRKR	Q7KZ85	SPT6H	Transcription elongation factor SPT6
KKRKR	O14776	TCRG1	Transcription elongation regulator 1

NVNAG	Q9UGU0	TCF20	Transcription factor 20
ALLST	Q7RTU1	TCF23	Transcription factor 23
KKRKR	Q9HCS4	TF7L1	Transcription factor 7-like 1
KKRKR	Q9NQB0	TF7L2	Transcription factor 7-like 2
TTILT	Q9NZI6	TF2L1	Transcription factor CP2-like protein 1
KQLLP	Q5H9I0	TFDP3	Transcription factor Dp family member 3
KQLLP	Q14186	TFDP1	Transcription factor Dp-1
CSISN	Q14186	TFDP1	Transcription factor Dp-1
VLDLK	Q16254	E2F4	Transcription factor E2F4
VLDLK	Q15329	E2F5	Transcription factor E2F5
INQSL	Q9Y543	HES2	Transcription factor HES-2
SAVSK	P31629	ZEP2	Transcription factor HIVEP2
VSSSV	Q5T1R4	ZEP3	Transcription factor HIVEP3
TSPLC	P52954	LBX1	Transcription factor LBX1
TSPLC	Q6XYB7	LBX2	Transcription factor LBX2
AGSVS	Q9Y5Q3	MAFB	Transcription factor MafB
SVITI	Q9ULX9	MAFF	Transcription factor MafF
TSVIT	O15525	MAFG	Transcription factor MafG
SVITI	O15525	MAFG	Transcription factor MafG
TSVIT	O60675	MAFK	Transcription factor MafK
SVITI	O60675	MAFK	Transcription factor MafK
SALLS	Q04206	TF65	Transcription factor p65
KKRKR	Q01201	RELB	Transcription factor RelB

ELQLL	Q9UN79	SOX13	Transcription factor SOX-13
ISNIE	P48436	SOX9	Transcription factor SOX-9
SNIET	P48436	SOX9	Transcription factor SOX-9
ASGQN	Q02447	SP3	Transcription factor Sp3
SGQNI	Q02447	SP3	Transcription factor Sp3
QNITE	Q8N5J4	SPIC	Transcription factor Spi-C
KEEVL	A6H8Y1	BDP1	Transcription factor TFIIIB component B" homolog
QEGKS	A6H8Y1	BDP1	Transcription factor TFIIIB component B" homolog
LILKA	Q00403	TF2B	Transcription initiation factor IIB
TNSEL	O00268	TAF4	Transcription initiation factor TFIID subunit 4
TNSEL	Q92750	TAF4B	Transcription initiation factor TFIID subunit 4B
SALLS	Q15542	TAF5	Transcription initiation factor TFIID subunit 5
RKRRF	Q15545	TAF7	Transcription initiation factor TFIID subunit 7
SKKRK	Q15361	TTF1	Transcription termination factor 1
KLIKQ	Q9UNY4	TTF2	Transcription termination factor 2
NSELL	P10071	GLI3	Transcriptional activator GLI3
SELLS	P10071	GLI3	Transcriptional activator GLI3
KSALL	O75478	TAD2A	Transcriptional adapter 2-alpha
SAVSK	P46100	ATRX	Transcriptional regulator ATRX
VSLSN	P46100	ATRX	Transcriptional regulator ATRX
DMPIT	Q2TAL8	QRIC1	Transcriptional regulator QRICH1
KGYLS	Q9BWW7	SCRT1	Transcriptional repressor scratch 1
VSSSV	Q04725	TLE2	Transducin-like enhancer protein 2

SALRT	Q04726	TLE3	Transducin-like enhancer protein 3
TTPVS	Q04727	TLE4	Transducin-like enhancer protein 4
TNSEL	Q9Y4A5	TRRAP	Transformation/transcription domain-associated protein
MITTI	Q9Y4A5	TRRAP	Transformation/transcription domain-associated protein
VKGEP	O75410	TACC1	Transforming acidic coiled-coil-containing protein 1
FLLGV	Q8TD43	TRPM4	Transient receptor potential cation channel subfamily M member 4
LLGVG	Q8TD43	TRPM4	Transient receptor potential cation channel subfamily M member 4
LLEIT	Q9BX84	TRPM6	Transient receptor potential cation channel subfamily M member 6
VGLLL	Q9BX84	TRPM6	Transient receptor potential cation channel subfamily M member 6
QNITE	Q96QT4	TRPM7	Transient receptor potential cation channel subfamily M member 7
NITEE	Q96QT4	TRPM7	Transient receptor potential cation channel subfamily M member 7
EITRE	Q96QT4	TRPM7	Transient receptor potential cation channel subfamily M member 7
DQKKL	Q96QT4	TRPM7	Transient receptor potential cation channel subfamily M member 7
SVGNT	Q8NER1	TRPV1	Transient receptor potential cation channel subfamily V member 1
FNPKY	Q8NET8	TRPV3	Transient receptor potential cation channel subfamily V member 3
ILKAN	P55072	TERA	Transitional endoplasmic reticulum ATPase
VVSLS	P55072	TERA	Transitional endoplasmic reticulum ATPase
DTMNS	Q9H0I9	TKTL2	Transketolase-like protein 2
VSKVL	Q9UI10	EI2BD	Translation initiation factor eIF-2B subunit delta
SVLTS	Q13144	EI2BE	Translation initiation factor eIF-2B subunit epsilon
EGSNI	Q9NR50	EI2BG	Translation initiation factor eIF-2B subunit gamma
NVNAG	P46199	IF2M	Translation initiation factor IF-2, mitochondrial precursor
VSVLT	Q8N609	TR1L1	Translocating chain-associated membrane protein 1-like 1

ALLST	Q9UNL2	SSRG	Translocon-associated protein subunit gamma
ILTAV	Q9NS93	TM7S3	Transmembrane 7 superfamily member 3 precursor
LTAVT	Q9NS93	TM7S3	Transmembrane 7 superfamily member 3 precursor
IVIIV	Q9HD45	TM9S3	Transmembrane 9 superfamily member 3 precursor
LLGVG	Q96BF3	TMIG2	Transmembrane and immunoglobulin domain-containing protein 2 precursor
LGVGS	Q96BF3	TMIG2	Transmembrane and immunoglobulin domain-containing protein 2 precursor
LGAIV	Q71RG4	TMUB2	Transmembrane and ubiquitin-like domain-containing protein 2
GAIVS	Q71RG4	TMUB2	Transmembrane and ubiquitin-like domain-containing protein 2
RFMNY	Q8TDI7	TMC2	Transmembrane channel-like protein 2
ITSLG	Q6UXY8	TMC5	Transmembrane channel-like protein 5
VGNTL	P0DMS9	TMIG3	Transmembrane domain-containing protein TMIGD3
ILLSL	P0DMS9	TMIG3	Transmembrane domain-containing protein TMIGD3
TELQL	Q7Z7H5	TMED4	Transmembrane emp24 domain-containing protein 4 precursor
FLGFL	Q14956	GPNMB	Transmembrane glycoprotein NMB precursor
LGFLL	Q14956	GPNMB	Transmembrane glycoprotein NMB precursor
IASGV	Q6ZMR5	TM11A	Transmembrane protease serine 11A
KSDEL	Q9BYE2	TMPSD	Transmembrane protease serine 13
KAVVS	O15393	TMPS2	Transmembrane protease serine 2 precursor
AVVSL	O15393	TMPS2	Transmembrane protease serine 2 precursor
TELQL	Q8N9M5	TM102	Transmembrane protein 102
ALLST	Q96AQ2	TM125	Transmembrane protein 125

GLLLY	Q96AQ2	TM125	Transmembrane protein 125
TKEGS	A2VDJ0	T131L	Transmembrane protein 131-like precursor
TLPSE	Q14DG7	T132B	Transmembrane protein 132B
LLSTN	Q14C87	T132D	Transmembrane protein 132D precursor
AVTEL	Q6IEE7	T132E	Transmembrane protein 132E precursor
TLPSE	Q6IEE7	T132E	Transmembrane protein 132E precursor
ILTAV	Q9NPI0	TM138	Transmembrane protein 138
NSELL	Q96AN5	TM143	Transmembrane protein 143
SELLS	Q96AN5	TM143	Transmembrane protein 143
SDELL	Q8IW70	T151B	Transmembrane protein 151B
TSKVL	Q8N614	TM156	Transmembrane protein 156
ALLST	Q5U3C3	TM164	Transmembrane protein 164
GFLLG	Q9H0V1	TM168	Transmembrane protein 168
PLYGV	Q7Z7N9	T179B	Transmembrane protein 179B
SNRVF	Q14CX5	MF13A	Transmembrane protein 180
LIAVG	Q8NFB2	T185A	Transmembrane protein 185A
LIAVG	Q9H7F4	T185B	Transmembrane protein 185B
VGLLL	Q66K66	TM198	Transmembrane protein 198
LQLLM	Q969S6	TM203	Transmembrane protein 203
QELDK	Q6NUQ4	TM214	Transmembrane protein 214
LLSLI	Q5W0B7	TM236	Transmembrane protein 236
VILLS	Q8N6L7	TM252	Transmembrane protein 252
LGFLL	Q8NCS4	TM35B	Transmembrane protein 35B precursor

SAIAS	Q69YG0	TMM42	Transmembrane protein 42
VSVLT	Q9BQJ4	TMM47	Transmembrane protein 47
ALLST	Q8N0U2	TMM61	Transmembrane protein 61
SVITS	Q6P5X7	TMM71	Transmembrane protein 71
SLAFI	Q8NBN3	TM87A	Transmembrane protein 87A precursor
FLLGV	Q6ZNR0	TMM91	Transmembrane protein 91
ELLIL	Q9C0B7	TNG6	Transport and Golgi organization protein 6 homolog
KQLLP	Q9C0B7	TNG6	Transport and Golgi organization protein 6 homolog
AVGLL	Q9Y5L0	TNPO3	Transportin-3
TSPLC	Q7Z2Z1	TICRR	Treslin
RARRE	Q9BT92	TCHP	Trichoplein keratin filament-binding protein
SEINL	Q9NP99	TREM1	Triggering receptor expressed on myeloid cells 1 precursor
IVILL	Q9NP99	TREM1	Triggering receptor expressed on myeloid cells 1 precursor
SLGAI	Q9UPQ9	TNR6B	Trinucleotide repeat-containing gene 6B protein
LKANA	Q14142	TRI14	Tripartite motif-containing protein 14
EGKSL	Q14134	TRI29	Tripartite motif-containing protein 29
ALRTG	Q8IWZ5	TRI42	Tripartite motif-containing protein 42
GSVSF	Q96BQ3	TRI43	Tripartite motif-containing protein 43
GSVSF	A6NCK2	TR43B	Tripartite motif-containing protein 43B
LTNSE	Q9C035	TRIM5	Tripartite motif-containing protein 5
GSVSF	A6NGJ6	TRI64	Tripartite motif-containing protein 64
SVSFF	A6NGJ6	TRI64	Tripartite motif-containing protein 64
GSVSF	A6NLI5	TR64C	Tripartite motif-containing protein 64C

SVSFF	A6NLI5	TR64C	Tripartite motif-containing protein 64C
SISQV	O15016	TRI66	Tripartite motif-containing protein 66
NRLLE	Q6ZTA4	TRI67	Tripartite motif-containing protein 67
TPVST	Q86UV7	TRI73	Tripartite motif-containing protein 73
TPVST	Q86UV6	TRI74	Tripartite motif-containing protein 74
LSLIN	O14773	TPP1	Tripeptidyl-peptidase 1 precursor
KTDVS	Q7Z2T5	TRM1L	TRMT1-like protein
VNKQE	Q7Z2T5	TRM1L	TRMT1-like protein
KVLDL	Q9H649	NSUN3	tRNA (cytosine(34)-C(5))-methyltransferase, mitochondrial
LLILK	O14717	TRDMT	tRNA (cytosine(38)-C(5))-methyltransferase
NGVSV	Q96GJ1	TRM2	tRNA (uracil(54)-C(5))-methyltransferase homolog
ELLSL	Q96GJ1	TRM2	tRNA (uracil(54)-C(5))-methyltransferase homolog
RRFLG	Q8IZ69	TRM2A	tRNA (uracil-5-)-methyltransferase homolog A
KLIKQ	Q8TBZ6	TM10A	tRNA methyltransferase 10 homolog A
NAGKS	Q969Y2	GTPB3	tRNA modification GTPase GTPBP3, mitochondrial precursor
VSKGY	Q9NX07	TSAP1	tRNA selenocysteine 1-associated protein 1
LVFPS	O60294	TYW4	tRNA wybutosine-synthesizing protein 4
KRKRR	Q8NCE0	SEN2	tRNA-splicing endonuclease subunit Sen2
LSALR	P0DKB5	TPBGL	Trophoblast glycoprotein-like precursor
ANAIT	Q9NYL9	TMOD3	Tropomodulin-3
ELDKY	P09493	TPM1	Tropomyosin alpha-1 chain
ELDKY	P06753	TPM3	Tropomyosin alpha-3 chain
RARRE	P45379	TNNT2	Troponin T, cardiac muscle

PVTLS	Q9NRR2	TRYG1	Tryptase gamma precursor
VSSSV	Q15714	T22D1	TSC22 domain family protein 1
TNVTL	O75157	T22D2	TSC22 domain family protein 2
LSLIA	O75157	T22D2	TSC22 domain family protein 2
VSSSV	O75386	TULP3	Tubby-related protein 3
SEINL	Q9NRJ4	TULP4	Tubby-related protein 4
PCWKL	P49815	TSC2	Tuberin
ASGVA	Q96A98	TIP39	Tuberoinfundibular peptide of 39 residues precursor
SFFPQ	Q14679	TTLL4	Tubulin monoglutamylase TTLL4
YDPLV	Q6EMB2	TTLL5	Tubulin polyglutamylase TTLL5
DAKVK	Q9BW30	TPPP3	Tubulin polymerization-promoting protein family member 3
VKLIK	Q8NG68	TTL	Tubulin--tyrosine ligase
VSKVL	Q9BXT4	TDRD1	Tudor domain-containing protein 1
LSNIK	Q9H7E2	TDRD3	Tudor domain-containing protein 3
IKSAL	O60522	TDRD6	Tudor domain-containing protein 6
SELLS	O60522	TDRD6	Tudor domain-containing protein 6
TFSNG	O14788	TNF11	Tumor necrosis factor ligand superfamily member 11
AVVSL	O43508	TNF12	Tumor necrosis factor ligand superfamily member 12
TVSVG	Q9H3D4	P63	Tumor protein 63
VGSAI	O43399	TPD54	Tumor protein D54
RLLEI	Q12792	TWF1	Twinfilin-1
DVSSS	Q15672	TWST1	Twist-related protein 1
VGLLL	Q8NHX9	TPC2	Two pore channel protein 2

SIMSI	Q8N6T0	TO6BL	Type 2 DNA topoisomerase 6 subunit B-like
AKVKL	P32019	I5P2	Type II inositol 1,4,5-trisphosphate 5-phosphatase precursor
KVKLI	P32019	I5P2	Type II inositol 1,4,5-trisphosphate 5-phosphatase precursor
KQEGK	Q06187	BTK	Tyrosine-protein kinase BTK
KAVVS	Q08881	ITK	Tyrosine-protein kinase ITK/TSK
KIKSA	Q05209	PTN12	Tyrosine-protein phosphatase non-receptor type 12
GSVSF	Q12923	PTN13	Tyrosine-protein phosphatase non-receptor type 13
NIKEN	Q99952	PTN18	Tyrosine-protein phosphatase non-receptor type 18
TNSEL	Q9H3S7	PTN23	Tyrosine-protein phosphatase non-receptor type 23
ASISQ	Q9H3S7	PTN23	Tyrosine-protein phosphatase non-receptor type 23
KANAI	Q9Y2Z4	SYYM	Tyrosine--tRNA ligase, mitochondrial precursor
RARRE	P08621	RU17	U1 small nuclear ribonucleoprotein 70 kDa
VLDLK	Q9BV90	SNR25	U11/U12 small nuclear ribonucleoprotein 25 kDa protein
TLSKK	O15042	SR140	U2 snRNP-associated SURP motif-containing protein
AVSKV	Q8TED0	UTP15	U3 small nucleolar RNA-associated protein 15 homolog
SIIKE	Q8TED0	UTP15	U3 small nucleolar RNA-associated protein 15 homolog
IKTFS	Q8TED0	UTP15	U3 small nucleolar RNA-associated protein 15 homolog
EEVLA	Q9Y5J1	UTP18	U3 small nucleolar RNA-associated protein 18 homolog
ILKAN	Q68CQ4	UTP25	U3 small nucleolar RNA-associated protein 25 homolog
LKANA	Q68CQ4	UTP25	U3 small nucleolar RNA-associated protein 25 homolog
ARREL	Q9NYH9	UTP6	U3 small nucleolar RNA-associated protein 6 homolog
LSKKR	O43818	U3IP2	U3 small nucleolar RNA-interacting protein 2
TPVTL	O43395	PRPF3	U4/U6 small nuclear ribonucleoprotein Prp3

AKVKL	O75643	U520	U5 small nuclear ribonucleoprotein 200 kDa helicase
KKRKR	Q6ZU65	UBN2	Ubinuclein-2
AGVTT	Q6ZU65	UBN2	Ubinuclein-2
PVTLS	Q6ZU65	UBN2	Ubinuclein-2
KVLHL	Q9Y5T5	UBP16	Ubiquitin carboxyl-terminal hydrolase 16
VITIE	Q9UPU5	UBP24	Ubiquitin carboxyl-terminal hydrolase 24
FLLGV	Q9UPU5	UBP24	Ubiquitin carboxyl-terminal hydrolase 24
LLSLI	Q9UPU5	UBP24	Ubiquitin carboxyl-terminal hydrolase 24
VRQQS	Q9UPU5	UBP24	Ubiquitin carboxyl-terminal hydrolase 24
KKRKR	Q9BXU7	UBP26	Ubiquitin carboxyl-terminal hydrolase 26
VLHLE	Q70CQ4	UBP31	Ubiquitin carboxyl-terminal hydrolase 31
KIKSA	Q70CQ2	UBP34	Ubiquitin carboxyl-terminal hydrolase 34
QIVRQ	Q70CQ2	UBP34	Ubiquitin carboxyl-terminal hydrolase 34
LVFPS	Q70EL4	UBP43	Ubiquitin carboxyl-terminal hydrolase 43
KLIKQ	Q9H0E7	UBP44	Ubiquitin carboxyl-terminal hydrolase 44
IAVGL	Q70EL2	UBP45	Ubiquitin carboxyl-terminal hydrolase 45
RLLEI	Q93009	UBP7	Ubiquitin carboxyl-terminal hydrolase 7
STPPT	P40818	UBP8	Ubiquitin carboxyl-terminal hydrolase 8
ASGVA	O95155	UBE4B	Ubiquitin conjugation factor E4 B
EEVLA	Q9NZ09	UBAP1	Ubiquitin-associated protein 1
TSVIT	Q5T6F2	UBAP2	Ubiquitin-associated protein 2
SVITS	Q5T6F2	UBAP2	Ubiquitin-associated protein 2
VSKVL	P51668	UB2D1	Ubiquitin-conjugating enzyme E2 D1

VSKVL	Q9Y2X8	UB2D4	Ubiquitin-conjugating enzyme E2 D4
QSLAF	Q8N2K1	UB2J2	Ubiquitin-conjugating enzyme E2 J2
VLDLK	Q8WVY7	UBCP1	Ubiquitin-like domain-containing CTD phosphatase 1
LSKKR	P22314	UBA1	Ubiquitin-like modifier-activating enzyme 1
LLGVG	A0AVT1	UBA6	Ubiquitin-like modifier-activating enzyme 6
SKVLD	O95352	ATG7	Ubiquitin-like modifier-activating enzyme ATG7
PITND	Q96S82	UBL7	Ubiquitin-like protein 7
SNICL	Q9UMW8	UBP18	Ubl carboxyl-terminal hydrolase 18
LSKDQ	Q5T124	UBX11	UBX domain-containing protein 11
AGSVS	Q92575	UBXN4	UBX domain-containing protein 4
VITSL	Q8NCR0	B3GL2	UDP-GalNAc:beta-1,3-N-acetylgalactosaminyltransferase 2
ITSLG	Q8NCR0	B3GL2	UDP-GalNAc:beta-1,3-N-acetylgalactosaminyltransferase 2
FLLGV	Q6UX72	B3GN9	UDP-GlcNAc:betaGal beta-1,3-N-acetylglucosaminyltransferase 9
GLLLY	Q6UX72	B3GN9	UDP-GlcNAc:betaGal beta-1,3-N-acetylglucosaminyltransferase 9
ELLHN	P35503	UD13	UDP-glucuronosyltransferase 1A3 precursor
ELLHN	P22310	UD14	UDP-glucuronosyltransferase 1A4 precursor
ELLHN	P35504	UD15	UDP-glucuronosyltransferase 1A5 precursor
LSALR	P0DTE4	UD2A1	UDP-glucuronosyltransferase 2A1 precursor
SALRT	P0DTE4	UD2A1	UDP-glucuronosyltransferase 2A1 precursor
LSALR	P0DTE5	UD2A2	UDP-glucuronosyltransferase 2A2
SALRT	P0DTE5	UD2A2	UDP-glucuronosyltransferase 2A2
FLGFL	Q76EJ3	S35D2	UDP-N-acetylglucosamine/UDP-glucose/GDP-mannose transporter
LGFLL	Q76EJ3	S35D2	UDP-N-acetylglucosamine/UDP-glucose/GDP-mannose transporter

SVNAG	A0JNW5	UH1BL	UHRF1-binding protein 1-like
RELPR	Q3MIX3	ADCK5	Uncharacterized aarF domain-containing protein kinase 5
ELQLL	Q5SQS8	CJ120	Uncharacterized protein C10orf120
LIKQE	Q86WS4	CL040	Uncharacterized protein C12orf40
CTTNT	Q8IXR9	CL056	Uncharacterized protein C12orf56
TTNTK	Q8IXR9	CL056	Uncharacterized protein C12orf56
TNTKE	Q8IXR9	CL056	Uncharacterized protein C12orf56
GVAVS	A8MV24	CQ098	Uncharacterized protein C17orf98
TLPSE	Q8N9H9	CA127	Uncharacterized protein C1orf127
NIKEN	Q5JVX7	CA141	Uncharacterized protein C1orf141
IKENK	Q5JVX7	CA141	Uncharacterized protein C1orf141
QSTPP	Q8N0U7	CA087	Uncharacterized protein C1orf87
SLTLP	Q6P1W5	CA094	Uncharacterized protein C1orf94
LTSKV	B9A014	F243A	Uncharacterized protein C21orf140
ELLSL	B9A014	F243A	Uncharacterized protein C21orf140
LEITR	Q9NWW7	CB042	Uncharacterized protein C2orf42
KVKLI	A6NCI8	CB078	Uncharacterized protein C2orf78
DVSSS	A6NCI8	CB078	Uncharacterized protein C2orf78
IASGV	A6NN90	CB081	Uncharacterized protein C2orf81
VQLPL	Q8N5N4	CC022	Uncharacterized protein C3orf22
SELLS	Q6ZRC1	CD050	Uncharacterized protein C4orf50
LSLIA	D6RIA3	CD054	Uncharacterized protein C4orf54
SLTLP	Q5T0Z8	CF132	Uncharacterized protein C6orf132

LTLPS	Q5T0Z8	CF132	Uncharacterized protein C6orf132
IKENK	Q5TEZ5	CF163	Uncharacterized protein C6orf163
ELQLL	Q96N11	CG026	Uncharacterized protein C7orf26
STPPT	Q5VYM1	CI131	Uncharacterized protein C9orf131
FYQST	Q9ULG3	CFA92	Uncharacterized protein CFAP92
LLSLI	Q8TB03	CX038	Uncharacterized protein CXorf38
LSLIN	Q8TB03	CX038	Uncharacterized protein CXorf38
SQVNE	Q8TB03	CX038	Uncharacterized protein CXorf38
LGVGS	A2RU37	CI170	Uncharacterized protein encoded by LINC02872
AGSVS	Q6ZSR9	YJ005	Uncharacterized protein FLJ45252
ILLSL	Q8IV33	K0825	Uncharacterized protein KIAA0825
STPPT	Q6ICG6	K0930	Uncharacterized protein KIAA0930
FASGQ	Q5HYC2	K2026	Uncharacterized protein KIAA2026
KIMTS	Q5HYC2	K2026	Uncharacterized protein KIAA2026
CDYVS	Q5HYC2	K2026	Uncharacterized protein KIAA2026
LLGVG	Q8N816	TMM99	Uncharacterized protein KRT10-AS1 precursor
LGVGS	Q8N816	TMM99	Uncharacterized protein KRT10-AS1 precursor
RARRE	Q8N2C9	UMAS1	Uncharacterized protein UMODL1-AS1
AKVKL	Q86XI8	ZSWM9	Uncharacterized protein ZSWIM9
ARREL	O43795	MYO1B	Unconventional myosin-Ib
KNNRL	O43795	MYO1B	Unconventional myosin-Ib
NNRLL	O43795	MYO1B	Unconventional myosin-Ib
NRLLE	O43795	MYO1B	Unconventional myosin-Ib

DAKVK	O00159	MYO1C	Unconventional myosin-Ic
YGKTK	O94832	MYO1D	Unconventional myosin-Id
MDTVS	O94832	MYO1D	Unconventional myosin-Id
TSLGA	O00160	MYO1F	Unconventional myosin-If
TVSVG	O00160	MYO1F	Unconventional myosin-If
LPLYG	B0I1T2	MYO1G	Unconventional myosin-Ig
NRLLE	Q9Y4I1	MYO5A	Unconventional myosin-Va
KEEVL	Q9Y4I1	MYO5A	Unconventional myosin-Va
ARREL	Q9ULV0	MYO5B	Unconventional myosin-Vb
SCSIS	Q9ULV0	MYO5B	Unconventional myosin-Vb
KEEVL	Q9ULV0	MYO5B	Unconventional myosin-Vb
DKQLL	Q6PIF6	MYO7B	Unconventional myosin-VIIb
ILLSL	Q6PIF6	MYO7B	Unconventional myosin-VIIb
IKEEV	Q9HD67	MYO10	Unconventional myosin-X
SALLS	Q9UKN7	MYO15	Unconventional myosin-XV
SLTLP	Q96JP2	MY15B	Unconventional myosin-XVB
KSALL	Q9Y6X6	MYO16	Unconventional myosin-XVI
SALLS	Q9Y6X6	MYO16	Unconventional myosin-XVI
VNAGK	Q9Y6X6	MYO16	Unconventional myosin-XVI
LHLEG	Q92614	MY18A	Unconventional myosin-XVIIIa
SALRT	Q8IUG5	MY18B	Unconventional myosin-XVIIIb
AVVSL	Q8IUG5	MY18B	Unconventional myosin-XVIIIb
KKLMS	Q8IUG5	MY18B	Unconventional myosin-XVIIIb

TDVSS	Q9HCM3	K1549	UPF0606 protein KIAA1549
SVITS	Q9HCM3	K1549	UPF0606 protein KIAA1549
GEVNK	Q6ZVL6	K154L	UPF0606 protein KIAA1549L
SKKRK	Q8N6N3	CA052	UPF0690 protein C1orf52
TTILT	Q9NZI7	UBIP1	Upstream-binding protein 1
QSTCS	Q9NZI7	UBIP1	Upstream-binding protein 1
VGNTL	Q13304	GPR17	Uracil nucleotide/cysteinyl leukotriene receptor
ILLSL	Q96BW1	UPP	Uracil phosphoribosyltransferase homolog
SAIAS	Q15849	UT2	Urea transporter 2
AIASG	Q15849	UT2	Urea transporter 2
CDTMN	P07911	UROM	Uromodulin precursor
AGSVS	O75445	USH2A	Usherin precursor
KDQLS	Q9BZF9	UACA	Uveal autoantigen with coiled-coil domains and ankyrin repeats
LKANA	Q96RL7	VP13A	Vacuolar protein sorting-associated protein 13A
KVLDL	Q96RL7	VP13A	Vacuolar protein sorting-associated protein 13A
VLDLK	Q96RL7	VP13A	Vacuolar protein sorting-associated protein 13A
SKDQL	Q96RL7	VP13A	Vacuolar protein sorting-associated protein 13A
NDQKK	Q7Z7G8	VP13B	Vacuolar protein sorting-associated protein 13B
DQKKL	Q7Z7G8	VP13B	Vacuolar protein sorting-associated protein 13B
VLHLE	Q709C8	VP13C	Vacuolar protein sorting-associated protein 13C
KVLDL	Q709C8	VP13C	Vacuolar protein sorting-associated protein 13C
DASIS	Q709C8	VP13C	Vacuolar protein sorting-associated protein 13C
TSVIT	Q5THJ4	VP13D	Vacuolar protein sorting-associated protein 13D

LSLIN	Q5THJ4	VP13D	Vacuolar protein sorting-associated protein 13D
SVITS	Q5THJ4	VP13D	Vacuolar protein sorting-associated protein 13D
SAVSK	Q96QK1	VPS35	Vacuolar protein sorting-associated protein 35
LEGEV	A5D8V6	VP37C	Vacuolar protein sorting-associated protein 37C
LSNGV	P49754	VPS41	Vacuolar protein sorting-associated protein 41 homolog
EEVLA	Q9NRW7	VPS45	Vacuolar protein sorting-associated protein 45
VSVLT	Q9UID3	VPS51	Vacuolar protein sorting-associated protein 51 homolog
TPVST	Q9UID3	VPS51	Vacuolar protein sorting-associated protein 51 homolog
IETVI	Q8N3P4	VPS8	Vacuolar protein sorting-associated protein 8 homolog
SLIAV	Q8N3P4	VPS8	Vacuolar protein sorting-associated protein 8 homolog
LIAVG	Q8N3P4	VPS8	Vacuolar protein sorting-associated protein 8 homolog
AVGLL	Q5ST30	SYVM	Valine--tRNA ligase, mitochondrial precursor
NITEE	P35916	VGFR3	Vascular endothelial growth factor receptor 3 precursor
TPVTL	Q14119	VEZF1	Vascular endothelial zinc finger 1
IRKSD	P32241	VIPR1	Vasoactive intestinal polypeptide receptor 1 precursor
STYML	P47901	V1BR	Vasopressin V1b receptor
ITEEF	P13611	CSPG2	Versican core protein precursor
IKEEV	P13611	CSPG2	Versican core protein precursor
KEEVL	P13611	CSPG2	Versican core protein precursor
VSSSV	P13611	CSPG2	Versican core protein precursor
TLSKD	Q68DQ2	CRBG3	Very large A-kinase anchor protein
IAVGL	Q6Y1H2	HACD2	Very-long-chain (3R)-3-hydroxyacyl-CoA dehydratase 2
NKAVV	Q9P035	HACD3	Very-long-chain (3R)-3-hydroxyacyl-CoA dehydratase 3

VVSLS	Q8NDX2	VGLU3	Vesicular glutamate transporter 3
NYIDK	P08670	VIME	Vimentin
DVSSS	P58304	VSX2	Visual system homeobox 2
LEGEV	P38435	VKGC	Vitamin K-dependent gamma-carboxylase
LTNSE	P22891	PROZ	Vitamin K-dependent protein Z precursor
NVNAG	P21796	VDAC1	Voltage-dependent anion-selective channel protein 1
VNEKI	Q9Y277	VDAC3	Voltage-dependent anion-selective channel protein 3
LTLPS	Q9BXT2	CCG6	Voltage-dependent calcium channel gamma-6 subunit
ITTIL	Q7Z3S7	CA2D4	Voltage-dependent calcium channel subunit alpha-2/delta-4 precursor
SLSNG	Q00975	CAC1B	Voltage-dependent N-type calcium channel subunit alpha-1B
TPPTN	O00555	CAC1A	Voltage-dependent P/Q-type calcium channel subunit alpha-1A
LTNSE	Q15878	CAC1E	Voltage-dependent R-type calcium channel subunit alpha-1E
IIVII	Q9P0X4	CAC1I	Voltage-dependent T-type calcium channel subunit alpha-1I
CNVDI	Q8TDW0	LRC8C	Volume-regulated anion channel subunit LRRC8C
LHLEG	Q6NSJ5	LRC8E	Volume-regulated anion channel subunit LRRC8E
GYLSA	Q7Z5H4	VN1R5	Vomeronasal type-1 receptor 5
LLGVG	Q5GFL6	VWA2	von Willebrand factor A domain-containing protein 2 precursor
LGVGS	Q5GFL6	VWA2	von Willebrand factor A domain-containing protein 2 precursor
GVGSA	Q5GFL6	VWA2	von Willebrand factor A domain-containing protein 2 precursor
LSALR	A6NCI4	VWA3A	von Willebrand factor A domain-containing protein 3A precursor
KVQSN	O00534	VMA5A	von Willebrand factor A domain-containing protein 5A
SLGAI	Q8N398	VW5B2	von Willebrand factor A domain-containing protein 5B2

RARRE	Q9Y334	VWA7	von Willebrand factor A domain-containing protein 7 precursor
VQLPL	P04275	VWF	von Willebrand factor precursor
GFLLG	Q16864	VATF	V-type proton ATPase subunit F
SGVAV	Q9UI12	VATH	V-type proton ATPase subunit H
STPPT	A6NGB9	WIPF3	WAS/WASL-interacting protein family member 3
AKKTN	Q641Q2	WAC2A	WASH complex subunit 2A
AKKTN	Q9Y4E1	WAC2C	WASH complex subunit 2C
IKEEV	Q12768	WASC5	WASH complex subunit 5
KEEVL	Q12768	WASC5	WASH complex subunit 5
SNGVS	Q8N5D0	WDTC1	WD and tetratricopeptide repeats protein 1
TELQL	Q8IZQ1	WDFY3	WD repeat and FYVE domain-containing protein 3
CDYVS	Q8IZQ1	WDFY3	WD repeat and FYVE domain-containing protein 3
SVLTS	Q6ZS81	WDFY4	WD repeat- and FYVE domain-containing protein 4
KKRKR	O75717	WDHD1	WD repeat and HMG-box DNA-binding protein 1
ELLIL	Q8IZU2	WDR17	WD repeat-containing protein 17
SEINL	Q8NEZ3	WDR19	WD repeat-containing protein 19
AIASG	Q8TBZ3	WDR20	WD repeat-containing protein 20
IASGV	Q8TBZ3	WDR20	WD repeat-containing protein 20
KKRKR	Q9UNX4	WDR3	WD repeat-containing protein 3
MDTVS	Q8NA23	WDR31	WD repeat-containing protein 31
DTVSV	Q8NA23	WDR31	WD repeat-containing protein 31
KSALL	Q8NI36	WDR36	WD repeat-containing protein 36
LSLIN	Q9HAD4	WDR41	WD repeat-containing protein 41

TEEFY	Q5JSH3	WDR44	WD repeat-containing protein 44
FPSDE	Q5JSH3	WDR44	WD repeat-containing protein 44
VVSLS	B1ANS9	WDR64	WD repeat-containing protein 64
LSLIA	Q9Y4E6	WDR7	WD repeat-containing protein 7
LHLEG	Q3MJ13	WDR72	WD repeat-containing protein 72
KVLDL	Q3MJ13	WDR72	WD repeat-containing protein 72
VQLPL	Q8IWA0	WDR75	WD repeat-containing protein 75
KKLMS	Q6ZQQ6	WDR87	WD repeat-containing protein 87
VQLPL	Q6ZQQ6	WDR87	WD repeat-containing protein 87
KQEGK	Q6ZQQ6	WDR87	WD repeat-containing protein 87
DASIS	Q7Z5K2	WAPL	Wings apart-like protein homolog
RQQSY	Q9GZV5	WWTR1	WW domain-containing transcription regulator protein 1
VITIE	Q9NQH7	XPP3	Xaa-Pro aminopeptidase 3 precursor
NIKEN	Q9NQH7	XPP3	Xaa-Pro aminopeptidase 3 precursor
SVLTS	P12955	PEPD	Xaa-Pro dipeptidase
YGVID	P12955	PEPD	Xaa-Pro dipeptidase
MDTVS	P47989	XDH	Xanthine dehydrogenase/oxidase
VNAGK	Q9UBH6	XPR1	Xenotropic and polytropic retrovirus receptor 1
SLSNG	A4UGR9	XIRP2	Xin actin-binding repeat-containing protein 2
LSNGV	A4UGR9	XIRP2	Xin actin-binding repeat-containing protein 2
VIDTP	A4UGR9	XIRP2	Xin actin-binding repeat-containing protein 2
TLSKD	A4UGR9	XIRP2	Xin actin-binding repeat-containing protein 2
SLIAV	Q5GH77	XKR3	XK-related protein 3

LSALR	Q6UX68	XKR5	XK-related protein 5
TKEGS	Q5GH72	XKR7	XK-related protein 7
LLSLI	Q9NP60	IRPL2	X-linked interleukin-1 receptor accessory protein-like 2 precursor
LDLKN	Q96KN7	RPGR1	X-linked retinitis pigmentosa GTPase regulator-interacting protein 1
LAFIR	Q9UM01	YLAT1	Y+L amino acid transporter 1
DVSSS	Q9BYJ9	YTHD1	YTH domain-containing family protein 1
RRELP	Q96MU7	YTDC1	YTH domain-containing protein 1
SNICL	Q96LD1	SGCZ	Zeta-sarcoglycan
SSSVI	O43829	ZBT14	Zinc finger and BTB domain-containing protein 14
SSVIT	Q05516	ZBT16	Zinc finger and BTB domain-containing protein 16
SVITS	Q05516	ZBT16	Zinc finger and BTB domain-containing protein 16
LVFPS	O15209	ZBT22	Zinc finger and BTB domain-containing protein 22
KVQSN	O15060	ZBT39	Zinc finger and BTB domain-containing protein 39
MLTNS	Q8NCP5	ZBT44	Zinc finger and BTB domain-containing protein 44
SAIAS	Q86UZ6	ZBT46	Zinc finger and BTB domain-containing protein 46
AKVKL	Q9UFB7	ZBT47	Zinc finger and BTB domain-containing protein 47
RLLEI	O95365	ZBT7A	Zinc finger and BTB domain-containing protein 7A
RLLEI	O15156	ZBT7B	Zinc finger and BTB domain-containing protein 7B
SELLS	O15156	ZBT7B	Zinc finger and BTB domain-containing protein 7B
LGFLL	Q96C00	ZBTB9	Zinc finger and BTB domain-containing protein 9
ALLST	Q96C00	ZBTB9	Zinc finger and BTB domain-containing protein 9
LTLPS	Q96C00	ZBTB9	Zinc finger and BTB domain-containing protein 9
NAGKS	Q96SZ4	ZSC10	Zinc finger and SCAN domain-containing protein 10

LSLIN	P17040	ZSC20	Zinc finger and SCAN domain-containing protein 20
VSSSV	Q3MJ62	ZSC23	Zinc finger and SCAN domain-containing protein 23
ARREL	Q96IU2	ZBED3	Zinc finger BED domain-containing protein 3
STPPT	O75132	ZBED4	Zinc finger BED domain-containing protein 4
NRLLE	O75132	ZBED4	Zinc finger BED domain-containing protein 4
IKTFS	O75152	ZC11A	Zinc finger CCCH domain-containing protein 11A
IKTFS	A0A1B0GTU1	ZC11B	Zinc finger CCCH domain-containing protein 11B
LILKA	Q6PJT7	ZC3HE	Zinc finger CCCH domain-containing protein 14
KKRKR	Q8WU90	ZC3HF	Zinc finger CCCH domain-containing protein 15
VTLSK	Q9UPT8	ZC3H4	Zinc finger CCCH domain-containing protein 4
NSELL	Q7Z2W4	ZCCHV	Zinc finger CCCH-type antiviral protein 1
NNVQI	Q8WYQ9	ZCH14	Zinc finger CCHC domain-containing protein 14
SDELL	O60315	ZEB2	Zinc finger E-box-binding homeobox 2
IAVGL	O60315	ZEB2	Zinc finger E-box-binding homeobox 2
VIDTP	O95405	ZFYV9	Zinc finger FYVE domain-containing protein 9
SNGCD	O95405	ZFYV9	Zinc finger FYVE domain-containing protein 9
DPLVF	O95405	ZFYV9	Zinc finger FYVE domain-containing protein 9
SQVNE	O95405	ZFYV9	Zinc finger FYVE domain-containing protein 9
IVILL	O95405	ZFYV9	Zinc finger FYVE domain-containing protein 9
SISQV	Q5H9K5	ZMAT1	Zinc finger matrin-type protein 1
RFLGF	Q5SVZ6	ZMYM1	Zinc finger MYM-type protein 1
AVTEL	O95789	ZMYM6	Zinc finger MYM-type protein 6
ELLSL	Q9H0C1	ZMY12	Zinc finger MYND domain-containing protein 12

LLSLI	Q9H0C1	ZMY12	Zinc finger MYND domain-containing protein 12
GSVSF	Q6P2D0	ZFP1	Zinc finger protein 1 homolog
AFIRK	P21506	ZNF10	Zinc finger protein 10
SALRT	Q9H2Y7	ZN106	Zinc finger protein 106
ILLSL	Q12901	ZN155	Zinc finger protein 155
GSVSF	P51786	ZN157	Zinc finger protein 157
VNEKI	P17022	ZNF18	Zinc finger protein 18
SLTLP	Q2M3W8	ZN181	Zinc finger protein 181
ARSTP	O15231	ZN185	Zinc finger protein 185
DVSSS	P98182	ZN200	Zinc finger protein 200
VVSLS	O95125	ZN202	Zinc finger protein 202
ISQVN	O95125	ZN202	Zinc finger protein 202
IKQEL	Q9UDV6	ZN212	Zinc finger protein 212
KSALL	Q8NDW4	ZN248	Zinc finger protein 248
KLHTS	Q9NSD4	ZN275	Zinc finger protein 275
YLSAL	Q9Y2X9	ZN281	Zinc finger protein 281
GVSVL	Q9Y2X9	ZN281	Zinc finger protein 281
NSELL	Q96NJ6	ZFP3	Zinc finger protein 3 homolog
SLTLP	Q9NR11	ZN302	Zinc finger protein 302
GFLLG	Q6U7Q0	ZN322	Zinc finger protein 322
GKTKC	Q9Y3S2	ZN330	Zinc finger protein 330
RRFLG	Q9H4Z2	ZN335	Zinc finger protein 335
GSVSF	Q06732	ZN33B	Zinc finger protein 33B

NVTLS	Q7RTV3	ZN367	Zinc finger protein 367
GSVSF	P17032	ZN37A	Zinc finger protein 37A
GSVSF	Q96SR6	ZN382	Zinc finger protein 382
LHLEG	Q8TD17	ZN398	Zinc finger protein 398
AVTEL	P15822	ZEP1	Zinc finger protein 40
GKSLY	P15822	ZEP1	Zinc finger protein 40
AVTEL	Q9C0G0	ZN407	Zinc finger protein 407
VSVLT	Q86VK4	ZN410	Zinc finger protein 410
GVAVS	Q8TF32	ZN431	Zinc finger protein 431
VAVSK	Q8TF32	ZN431	Zinc finger protein 431
VNEKI	Q8N8Z8	ZN441	Zinc finger protein 441
ELQLL	Q02386	ZNF45	Zinc finger protein 45
ARSTP	Q96JG9	ZN469	Zinc finger protein 469
AFIRK	Q5JVG2	ZN484	Zinc finger protein 484
FIRKS	Q5JVG2	ZN484	Zinc finger protein 484
SELLS	Q6ZNH5	ZN497	Zinc finger protein 497
SELLS	O60304	ZN500	Zinc finger protein 500
ICLTR	Q96CX3	ZN501	Zinc finger protein 501
LSALR	Q96F45	ZN503	Zinc finger protein 503
RARRE	Q92618	ZN516	Zinc finger protein 516
DQLSG	Q9C0D4	Z518B	Zinc finger protein 518B
KTFSN	Q8TF50	ZN526	Zinc finger protein 526
SLTLP	Q6P280	ZN529	Zinc finger protein 529

RQQSY	Q86UE3	ZN546	Zinc finger protein 546
AFIRK	Q8NEK5	ZN548	Zinc finger protein 548
FIRKS	Q8NEK5	ZN548	Zinc finger protein 548
KTFSN	Q8N988	ZN557	Zinc finger protein 557
GSVSF	Q8N184	ZN567	Zinc finger protein 567
AVGLL	Q96NI8	ZN570	Zinc finger protein 570
GSVSF	Q6P3V2	Z585A	Zinc finger protein 585A
AFIRK	Q6P3V2	Z585A	Zinc finger protein 585A
FIRKS	Q6P3V2	Z585A	Zinc finger protein 585A
GSVSF	Q52M93	Z585B	Zinc finger protein 585B
LAFIR	Q52M93	Z585B	Zinc finger protein 585B
AFIRK	Q52M93	Z585B	Zinc finger protein 585B
FIRKS	Q52M93	Z585B	Zinc finger protein 585B
GSAIA	Q92610	ZN592	Zinc finger protein 592
AFIRK	Q86T29	ZN605	Zinc finger protein 605
NKNRG	Q9ULD9	ZN608	Zinc finger protein 608
LLSTN	O15014	ZN609	Zinc finger protein 609
LSLIN	Q8N883	ZN614	Zinc finger protein 614
IELSN	Q5T7W0	ZN618	Zinc finger protein 618
KTFSN	Q8NB50	ZFP62	Zinc finger protein 62 homolog
TLPSE	Q14966	ZN638	Zinc finger protein 638
DVSSS	Q14966	ZN638	Zinc finger protein 638
KKRKR	Q9UID6	ZN639	Zinc finger protein 639

TLPSE	Q9NTW7	ZF64B	Zinc finger protein 64
KKRKR	Q9H582	ZN644	Zinc finger protein 644
TSKTD	P17097	ZNF7	Zinc finger protein 7
KKLMS	Q9NV72	ZN701	Zinc finger protein 701
STCSA	Q9H7S9	ZN703	Zinc finger protein 703
FIRKS	O43361	ZN749	Zinc finger protein 749
PQAET	Q32MQ0	ZN750	Zinc finger protein 750
SEINL	Q32MQ0	ZN750	Zinc finger protein 750
SKVLH	P36508	ZNF76	Zinc finger protein 76
ALLST	Q9H5H4	ZN768	Zinc finger protein 768
SALRT	Q3KP31	ZN791	Zinc finger protein 791
KSALL	A4D1E1	Z804B	Zinc finger protein 804B
KTFSN	Q8N4W9	ZN808	Zinc finger protein 808
AVSKG	Q3KNS6	ZN829	Zinc finger protein 829
PQAET	Q5JPB2	ZN831	Zinc finger protein 831
TLPSE	A8K0R7	ZN839	Zinc finger protein 839
SVLTS	A6NHJ4	ZN860	Zinc finger protein 860
KSALL	P0CJ79	ZN888	Zinc finger protein 888
LSALR	A8MXY4	ZNF99	Zinc finger protein 99
RLLEI	Q92784	DPF3	Zinc finger protein DPF3
QLLMQ	Q8IYY4	DZI1L	Zinc finger protein DZIP1L
NAGVT	P08151	GLI1	Zinc finger protein GLI1
LLMQS	P10070	GLI2	Zinc finger protein GLI2

NSLTL	P10070	GLI2	Zinc finger protein GLI2
SLTLP	P10070	GLI2	Zinc finger protein GLI2
LTLPS	P10070	GLI2	Zinc finger protein GLI2
SELLS	Q6DJT9	PLAG1	Zinc finger protein PLAG1
ELLSL	P14373	TRI27	Zinc finger protein RFP
ASNKN	Q13129	RLF	Zinc finger protein Rlf
TSLGA	Q3KNW1	SNAI3	Zinc finger protein SNAI3
GVGSA	Q92785	REQU	Zinc finger protein ubi-d4
FIRKS	Q63HK3	ZKSC2	Zinc finger protein with KRAB and SCAN domains 2
SALLS	Q15776	ZKSC8	Zinc finger protein with KRAB and SCAN domains 8
SELLS	Q9P243	ZFAT	Zinc finger protein ZFAT
IKQEL	Q15915	ZIC1	Zinc finger protein ZIC 1
STPPT	Q15915	ZIC1	Zinc finger protein ZIC 1
IKQEL	O95409	ZIC2	Zinc finger protein ZIC 2
IKQEL	O60481	ZIC3	Zinc finger protein ZIC 3
LGVGS	O60481	ZIC3	Zinc finger protein ZIC 3
IKQEL	Q8N9L1	ZIC4	Zinc finger protein ZIC 4
IKQEL	Q96T25	ZIC5	Zinc finger protein ZIC 5
ASGVA	Q96KR1	ZFR	Zinc finger RNA-binding protein
STPVT	Q96KR1	ZFR	Zinc finger RNA-binding protein
SEINL	Q9H7M6	ZSWM4	Zinc finger SWIM domain-containing protein 4
REFSV	O43149	ZZEF1	Zinc finger ZZ-type and EF-hand domain-containing protein 1
TTIII	Q9UKY1	ZHX1	Zinc fingers and homeoboxes protein 1

DQLSG	Q9Y6X8	ZHX2	Zinc fingers and homeoboxes protein 2
AVGLL	Q92504	S39A7	Zinc transporter SLC39A7
LLHNV	Q6P5W5	S39A4	Zinc transporter ZIP4 precursor
DVSSS	Q13433	S39A6	Zinc transporter ZIP6 precursor
LLGVG	P21754	ZP3	Zona pellucida sperm-binding protein 3 precursor
VSVLT	Q6X784	ZPBP2	Zona pellucida-binding protein 2 precursor
VGLLL	Q96DA0	ZG16B	Zymogen granule protein 16 homolog B precursor
VVQLP	Q8IYH5	ZZZ3	ZZ-type zinc finger-containing protein 3

Proteins given with AC, ID, and name entries according to UniProt.
